# Late-stage *meta*-C–H alkylation of pharmaceuticals to modulate biological properties and expedite molecular optimisation in a single step

**DOI:** 10.1038/s41467-024-46697-8

**Published:** 2024-04-18

**Authors:** Lucas Guillemard, Lutz Ackermann, Magnus J. Johansson

**Affiliations:** 1 Medicinal Chemistry, Research and Early Development, Cardiovascular, Renal and Metabolism (CVRM), BioPharmaceuticals R&D, AstraZeneca, Gothenburg, Sweden; 2 Institut für Organische und Biomolekulare Chemie and Wöhler Research Institute for Sustainable Chemistry (WISCh), Georg-August-Universität Göttingen, Göttingen, Germany; 3 German Centre for Cardiovascular Research (DZHK), Berlin, Germany

**Keywords:** Homogeneous catalysis, Drug discovery and development, Drug discovery and development

## Abstract

Catalysed C–H activation has emerged as a transformative platform for molecular synthesis and provides new opportunities in drug discovery by late-stage functionalisation (LSF) of complex molecules. Notably, small aliphatic motifs have gained significant interest in medicinal chemistry for their beneficial properties and applications as *sp*^3^-rich functional group bioisosteres. In this context, we disclose a versatile strategy with broad applicability for the ruthenium-catalysed late-stage *meta*-C(*sp*^2^)–H alkylation of pharmaceuticals. This general protocol leverages numerous directing groups inherently part of bioactive scaffolds to selectivity install a variety of medicinally relevant bifunctional alkyl units within drug compounds. Our strategy enables the direct modification of unprotected lead structures to quickly generate an array of pharmaceutically useful analogues without resorting to de novo syntheses. Moreover, productive late-stage modulation of key biological characteristics of drug candidates upon remote C–H alkylation proves viable, highlighting the major benefits of our approach to offer in drug development programmes.

## Abstract (web summary)

Installation of small aliphatic motifs within pharmaceuticals provides a medicinally relevant tool in drug discovery programmes. Here, the authors report a late-stage *meta*-C–H alkylation method facilitating the biological properties modulation of therapeutic agents.

## Introduction

Transition-metal catalysed C–H functionalisation has surfaced as a robust platform to enable entirely new synthetic disconnections and to significantly improve resource economy^1^. Specifically, proximity-induced C–H activation has matured to a powerful and predictable strategy that harnesses Lewis-basic motifs to control position-selectivity^2^. Beyond simple, privileged *ortho*-activations, approaches for remote *meta-* or *para-*C–H functionalisation have gained considerable momentum^3–5^. In recent years, major advances in the development of mild C–H activation protocols with broad functional group tolerance allowed the late-stage functionalisation (LSF) of increasingly complex molecules^6,7^. In particular, LSF methodologies have been successfully applied to the C–H diversification of pharmaceuticals^8,9^, offering new opportunities in medicinal chemistry and enabling the implementation of these transformations in modern drug discovery programmes^10,11^. Such approaches allow the direct modification of biologically active scaffolds to provide a range of relevant analogues, avoiding tedious de novo syntheses of each targeted compound^12^. From the perspective of drug development, the most synthetically useful LSF methods enable the selective C–H installation of small groups that have the capacity to positively influence key biological characteristics of a drug molecule without considerably altering its structural feature^13^. Notably, a variety of emerging small alkyl groups and aliphatic ring systems have been increasingly exploited in medicinal chemistry for their advantageous physicochemical properties and applications as bioisosteres^14–16^.

Additionally, benzylic C–H bonds are the preferred metabolic soft spots, easily oxidised by cytochromes P450, so the introduction of sterically hindered *gem*-disubstituted units at benzylic positions is highly valuable in order to block metabolically exposed sites in a biologically active compound^17,18^. However, despite significant progress in the field of LSF, challenges and limitations remain regarding the scope and selectivity of transition-metal catalysed C(*sp*^2^)–C(*sp*^3^) bond forming processes in elaborated molecular settings^19^. Especially, to the best of our knowledge, no approaches enabling the direct LSF of drug compounds with complete selectivity for the distal *meta*-position have been reported. Furthermore, LSF methodologies designed with the aim of providing a medicinally relevant tool to optimise drug-like molecular characteristics are still underdeveloped, resulting in a continued strong demand for such innovative C–H transformations^20^. In this context, we envisaged the unique capacity of ruthenium to direct remote C–H activation^21^ as an attractive strategy for diversifying the architecture of advanced therapeutic agents.

**Fig. 1:**
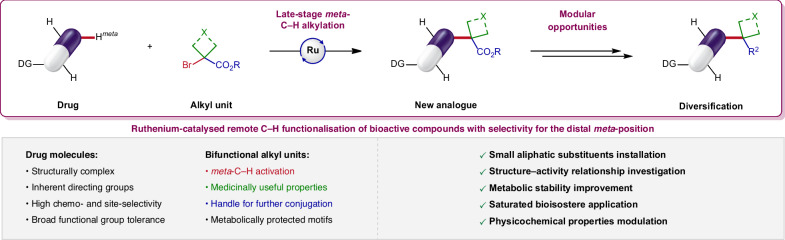
Ruthenium-catalysed late-stage *meta*-C(*sp*^2^)–H alkylation of pharmaceuticals. A versatile strategy enabling the late-stage installation of small aliphatic motifs within bioactive molecules to generate an array of useful analogues in a single step. Upon remote C–H alkylation selectively at the distal *meta*-position, this approach offering opportunities for further diversification provides a medicinally relevant tool to modulate biological properties and expedite molecular optimisation of drug compounds. DG directing group.

Here, we report a powerful method for the ruthenium-catalysed late-stage *meta*-C(*sp*^2^)–H alkylation of pharmaceuticals to produce an array of relevant bioactive analogues in a single step (Fig. 1). To this end, we develop a highly site-selective approach with excellent tolerance of unprotected functionalities by taking advantage of proximity-induced C–H activation with Lewis-basic sites inherently present in almost all structurally complex drugs, without the need for pre-installation and removal of artificial auxiliaries^22,23^. In order to expand the toolbox for medicinal chemists, we introduce a wide variety of diverse small aliphatic motifs finding useful applications in drug design, in a straightforward and predictable manner. In this work, we employ valuable bifunctional reagents^24^, coupling partners consisting of geminal C1-alkyl units bearing a dual synthetic handle, allowing C–H activation followed by sequential conjugation. This generally applicable *meta* LSF protocol for expediting the diversification of pharmaceuticals has the potential to profoundly impact medicinal chemistry and allow increased efficiency in the drug discovery process. Indeed, the direct incorporation of such saturated substituents within drug compounds provides productive entries into structure-activity relationships (SAR) investigation, metabolic stability improvement, biological properties modulation and bioisosteric replacements, as well as a versatile handle for further bioconjugations. In addition, this strategy offers flexibility in route development by giving access to new late-stage disconnections with high resource economy and substantial benefits in terms of sustainability through reduced waste generation. Moreover, it is noteworthy that accessing such *meta*-C–H alkylated drug derivatives via other synthetic means or by resorting to de novo syntheses with lengthy step counts would be greatly challenging. Finally, this approach offers complementarity in site-selectivity to proximal *ortho*-C–H activation strategies, enabling the late-stage alkylation remotely from polar directing groups, which can be a desired feature in medicinal chemistry, especially when aiming for proteolysis-targeting chimeras (PROTACs) and other drug conjugates.

## Results

### Multiparameter optimisation

**Fig. 2:**
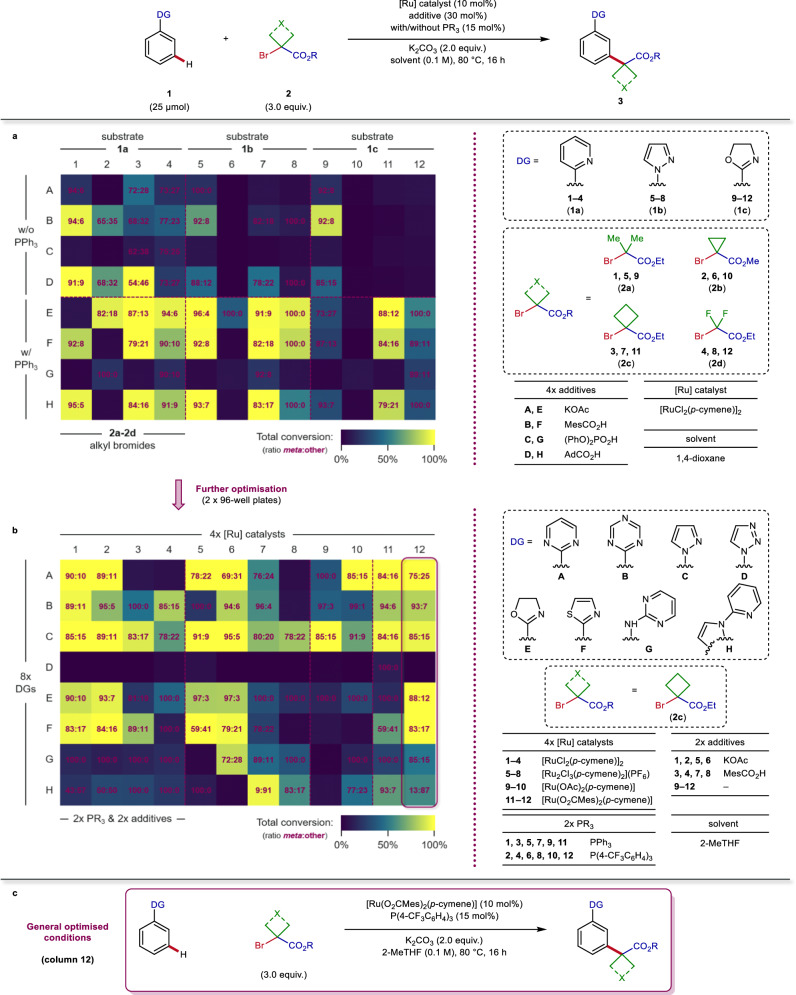
Optimisation of ruthenium-catalysed *meta*-C‒H alkylation via high-throughput experimentation (HTE). Reaction conditions: substrate **1** (25 μmol), alkyl bromide **2** (75 μmol), [Ru] catalyst (10 mol%), phosphine ligand (15 mol%), additive (30 mol%) and K_2_CO_3_ (50 μmol) in solvent (0.1 M) at 80 °C for 16 h. Heat maps: visualisation of total conversions and levels of *meta*-selectivity determined by LC-MS. **a** Initial multi-parameter evaluation of ruthenium catalytic systems against a combination of substrates with different properties. **b** Directing group informer library approach to assess the efficiency of this method towards motifs inherently present in drug molecules. **c** General reaction conditions selected for the *meta*-C‒H alkylation after optimisation studies (see Supplementary Section 2.1 for more details). Ac acetyl, Ad 1-adamantyl, DG directing group, Mes mesityl, 2-MeTHF 2-methyltetrahydrofuran, w/ with, w/o without.

A high-throughput experimentation (HTE) campaign involving automation technologies was initiated for the multi-parameter optimisation of this ruthenium-catalysed *meta*-C–H alkylation in a time-efficient manner^25^. We took advantage of a HTE approach to generate a large set of data points with minimised resource consumption in order to identify general reaction conditions applicable to a wide range of both coupling partners from the outset of our optimisation studies^26^. Initial optimisation with [Ru(*p*-cymene)Cl_2_]_2_ as the precatalyst and a selection of carboxylate additives was performed against the combination of 3 Lewis-basic sites **1a-1c** and 4 alkyl bromides **2a-2d** (Fig. 2a). These substrates were chosen to represent different levels of Lewis basicity and electronic properties, factors expected to have an impact both on the success and the site-selectivity of the C–H alkylation^27^. The presence of a phosphine ligand turned out to be crucial for higher reactivity as well as improved regioselectivity into the desired *meta*-functionalised products **3**^28,29^. Further HTE investigations involving other substrates revealed MesCO_2_H as a generally more effective additive in providing the C–H alkylated products, and indicated the importance of electron deficient substituents on the phosphine ligand to obtain better conversions. A valuable finding was the identification of 2-methyltetrahydrofuran (2-MeTHF)^30^ as a greener solvent giving similar reaction outcome than other organic media employed for this cross-coupling (see Supplementary Fig. 3 for details).

To assess the efficiency of this method more broadly towards motifs inherently present in drug molecules, we pursued the optimisation process by using a directing group informer library approach^31,32^. To this end, a set consisting of 8 different functional groups commonly found in bioactive compounds and able to direct the *meta*-C‒H activation was evaluated in this transformation, using alkyl bromide **2c** against 4 ruthenium catalytic systems (Fig. 2b). Interestingly, cationic complex [Ru_2_Cl_3_(*p*-cymene)_2_](PF_6_) performed equally well, and preformed dicarboxylate complexes were also competent ruthenium sources for this C–H alkylation protocol, allowing good to excellent conversions and levels of *meta*-selectivity for 7 out of the 8 substrates tested. Finally, optimisation studies led to a general set of mild reaction conditions, using [Ru(O_2_CMes)_2_(*p*-cymene)] as catalyst in combination with P(4-CF_3_C_6_H_4_)_3_ ligand, and K_2_CO_3_ as base in 2-MeTHF at 80 °C (Fig. 2c).

### Scope of directing groups

**Fig. 3:**
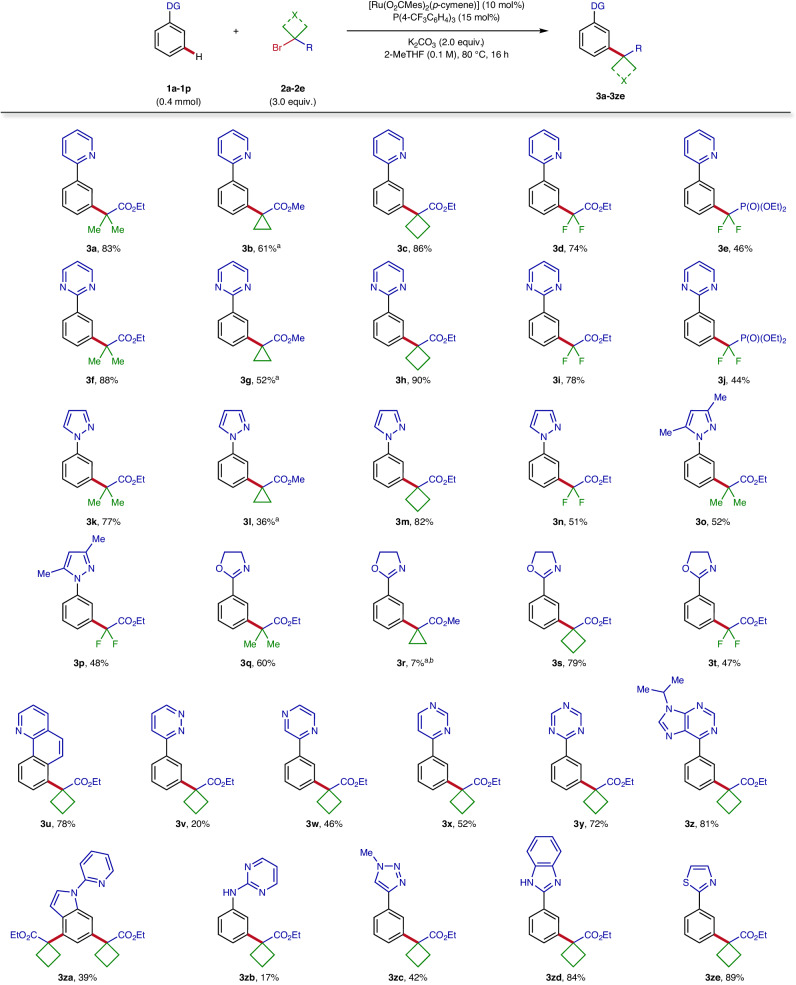
Scope of efficient directing groups for the ruthenium-catalysed *meta*-C‒H alkylation. General optimised reaction conditions: substrate **1** (0.40 mmol), alkyl bromide **2** (1.20 mmol), [Ru(O_2_CMes)_2_(*p*-cymene)] (22.5 mg, 0.04 mmol), P(4-CF_3_C_6_H_4_)_3_ (28.0 mg, 0.06 mmol) and K_2_CO_3_ (111 mg, 0.80 mmol) in 2-MeTHF (4.0 mL, 0.1 M) at 80 °C for 16 h. ^a^*tert*-amyl alcohol (*t*-AmOH) was used as solvent. ^b^Conversion determined by LC-MS, product not isolated. DG directing group, Mes mesityl, 2-MeTHF 2-methyltetrahydrofuran.

With the optimised reaction conditions in hand, the scope of this *meta*-C‒H activation was evaluated on a synthetically useful scale. To confirm the connectivity of C–H alkylated products obtained throughout the optimisation campaign, reactions were likewise scaled up 16 times from the HTE setting with identical performance (Fig. 3). Alkyl bromides with different electronic properties (nucleophilic **2a-2c** and electrophilic **2d,** **2e**) were efficiently tolerated in this C(*sp*^2^)–C(*sp*^3^) bond forming process, providing the corresponding coupling products **3** in moderate to excellent yields. An only slightly reduced efficacy was observed for phosphonate (**2e**) and cyclopropyl (**2b**) derivatives. For the latter, conversions could be improved by using *tert*-amyl alcohol (*t*-AmOH) as the solvent, showcasing the importance of our HTE approach to identify alternative reaction conditions. It is noteworthy that the catalytic system was broadly applicable to a wide range of Lewis-basic functionalities ubiquitous in natural products and pharmaceuticals, that can serve as productive directing groups for this *meta*-C–H activation. Indeed, numerous heterocycles comprising a variety of azines and azoles could be leveraged to guide the C–H alkylation in an effective manner. This includes for instance heterocyclic moieties such as pyrimidine (**3f-3j**), pyrazole (**3k-3p**), oxazoline (**3q-3t**), triazine (**3y**) and thiazole (**3ze**), all displaying good to excellent reactivity for this transformation. Interestingly, the reaction also proceeded with *N*-pyridyl indole (**3za**) and 2-aminopyrimidine (**3zb**), presumably via formation of 6-membered ruthenacycle intermediates, furnishing the *meta*-(bis)functionalised products although in moderate yields.

To our delight, further functional groups frequently encountered in drug-type molecules were identified to successfully direct this *meta*-C–H alkylation. Indeed, *N*-heterocycles such as quinoline (**3u**) as well as pyridazine (**3v**), pyrazine (**3w**) and 4-pyrimidine (**3x**) were also capable of delivering the desired alkylated products. Furthermore, synthetically useful moieties such as purine nucleobase derivative (**3z**) and triazole (**3zc**) were competent directing groups for this method, respectively offering opportunities for direct fluorescent labelling and click chemistry product diversification. Moreover, substrate containing a free *NH*-benzimidazole was exclusively converted to the desired C–H functionalised product **3zd** in very good yield, again highlighting the excellent levels of chemo- and *meta*-selectivity of this transformation. Interestingly, almost all the heteroarene motifs proved viable with our approach are among the most frequent heterocycles in approved pharmaceuticals by the United States Food and Drug Administration (U.S. FDA)^33^, attesting the usefulness of our strategy in drug discovery programmes.

### LSF screening

**Fig. 4:**
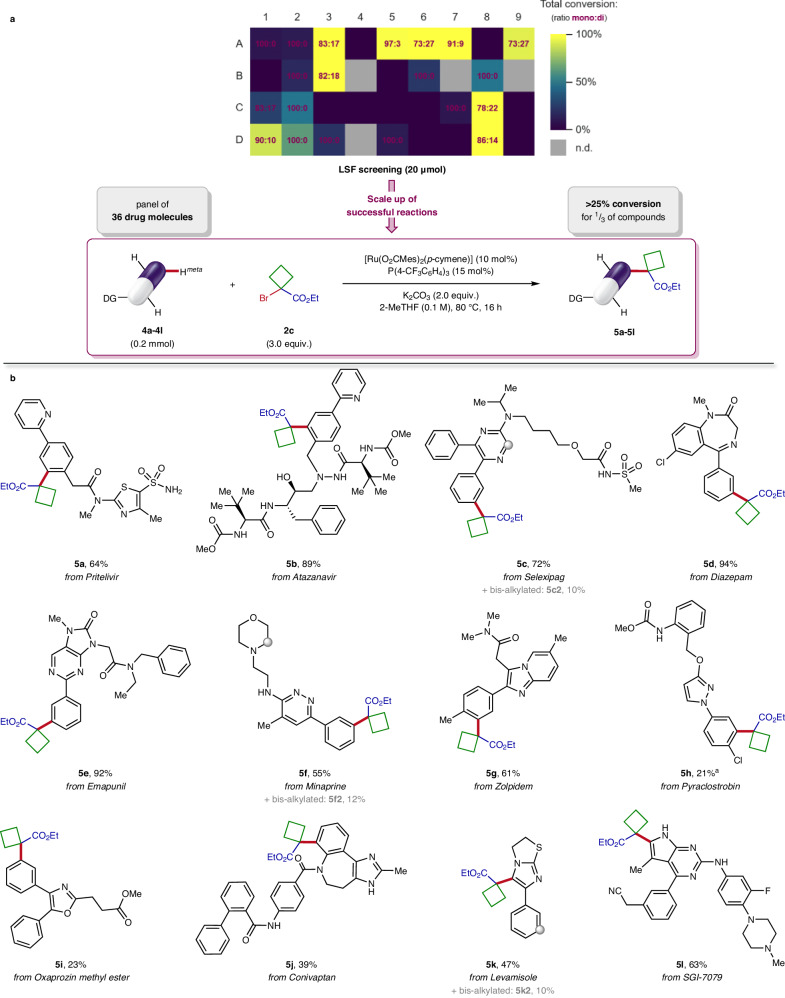
Late-stage functionalisation (LSF) of bioactive molecules via ruthenium-catalysed *meta*-C‒H alkylation. **a** High-throughput experimentation (HTE) screening of 36 commercial drug molecules against the general optimised reaction conditions with alkyl bromide **2c** on a 20 μmol LSF substrate scale. Heat map: visualisation of total conversions and levels of mono-functionalisation determined by LC-MS (see Supplementary Section 2.2 for more details). **b** Scope of successful LSF substrates after scale up. General optimised reaction conditions: LSF substrate **4** (0.20 mmol), alkyl bromide **2c** (97.1 μL, 0.60 mmol), [Ru(O_2_CMes)_2_(*p*-cymene)] (11.2 mg, 0.02 mmol), P(4-CF_3_C_6_H_4_)_3_ (14.0 mg, 0.03 mmol) and K_2_CO_3_ (55 mg, 0.40 mmol) in 2-MeTHF (2.0 mL, 0.1 M) at 80 °C for 16 h. ^a^In situ cleavage of N–OMe bond from the methoxylamine group of pyraclostrobin (**4h**) occurred during the transformation: product **5h** was isolated as the corresponding 2° carbamate. DG directing group, Mes mesityl, 2-MeTHF 2-methyltetrahydrofuran.

Having identified various efficient directing groups for this C‒H alkylation on unexplored building blocks, we were interested to investigate the LSF applicability of this transformation on significantly more complex molecular settings. To this end, a library of 36 commercially available drugs was evaluated in this late-stage *meta*-C‒H functionalisation, using alkyl bromide **2c** as coupling partner (Fig. 4a). This panel of bioactive compounds was selected based both on the available functional group(s) capable of successfully guide the C(*sp*^2^)‒H activation and the accessible *meta*-alkylation site(s) inherently present in the drug scaffolds (see Supplementary Section 2.2 for details). Furthermore, representative examples bearing unprotected groups typically found in pharmaceuticals were chosen in order to cover structural diversity and explore medicinally relevant chemical space with this catalytic system^34^. We foresaw this screening to promptly assess the robustness and generality of our reaction conditions towards real-world examples of complex molecules with industrial applications. It would also allow to evaluate the tolerance and compatibility of this protocol with sensitive functional groups, and provide information on the site-selectivity of the alkylation with scaffolds offering several suitable C–H bonds.

A HTE approach was deemed as the most appropriate strategy to rapidly conduct this preliminary LSF screening while using minimal amounts of valuable starting materials. Therefore, each drug molecule was probed against the optimised reaction conditions on a 20 μmol LSF substrate scale. Remarkably, for one third of the compounds (12 out of 36 drugs tested) a positive hit was obtained, defined as >25% conversion to the C–H alkylated product determined by LC-MS, thus corresponding to a success rate of 33% (Fig. 4a, see Supplementary Section 2.2 for details). As anticipated from the previous results, the screening of LSF substrates pleasingly revealed that a broad range of Lewis-basic functionalities inherently present in the pharmaceuticals architecture can be harnessed to efficiently direct the C–H activation. Indeed, after analysis of the successful reactions, an array of *N*-heterocycles ubiquitous in advanced molecules (namely azines, azoles and imines) was identified as competent directing groups for this late-stage *meta*-C–H alkylation (Fig. 4b). Gratifyingly, a variety of sensitive substituents including unprotected polar groups (basic amines, alcohols and nitriles), protic acids (acyl sulphonamides), hydrogen bond donors or acceptors (peptides backbone) and halogens (aryl chlorides) were compatible with this procedure. This showcases the high functional group tolerance of this methodology towards moieties typically present in bioactive molecules, as well as its robustness and applicability for LSF. It is noteworthy that good levels of mono-functionalisation are generally observed for most of the substrates, highlighting the strong chemo- and site-selectivity of the catalytic system.

### LSF scope

To validate the identity of C–H alkylated products obtained during the LSF screening (**5a-5l**), successful reactions were scaled up 10 times from the HTE conditions again without affecting the outcome (Fig. 4b). The scope of drug molecules revealed a set of 9 different innate functional groups able to promote this remote late-stage *meta*-C–H activation with moderate to excellent reactivity. The reaction proceeded smoothly with pyridine directing groups, while tolerating the unprotected thiazolylsulphonamide moiety of pritelivir (**4a**) and peptide backbone of atazanavir (**4b**), delivering the mono-functionalised products **5a** and **5b** respectively in 64% and 89% yield. Remarkably, high chemo- and regioselectivity was achieved when performing the transformation on selexipag (**4c**) bearing an acidic acyl sulphonamide group and displaying a 2,3-diphenylpyrazine scaffold with multiple reactive sites for C–H activation. Indeed, the C(*sp*^2^)–H bond at the *meta*-position of the least sterically hindered Lewis-basic *sp*^2^ nitrogen was selectively functionalised, providing the corresponding product **5c** in 72% yield. Both diazepam (**4d**) and emapunil (**4e**) proved to be excellent substrates to direct the C–H alkylation by exploiting imine or pyrimidine functionalities, furnishing the *meta*-substituted derivatives **5d** and **5e** in almost quantitative yields. Interestingly, pyridazine-containing minaprine (**4f**) efficiently afforded the desired C–H functionalised product **5f** in 55% yield, despite the presence of unprotected and strongly coordinating aniline or tertiary amine moieties in the bioactive compound. Fused imidazopyridine ring system found in the core structure of zolpidem (**4g**) was also a competent directing group in this C–H activation, giving the *meta*-alkylated analogue **5g** in good yield. Substrates with pyrazole (**4h**) and oxazole (**4i**) Lewis-basic groups proved viable, affording the desired pyraclostrobin and oxaprozin derivatives **5h** and **5i** in synthetically useful yields as the exclusive alkylated products. However, in situ cleavage of N–O bond from the methoxylamine group of pyraclostrobin delivered **5h** as the corresponding carbamate, with the alkyl group installed at the congested *ortho*-position of chlorine substituent that can serve as a handle for subsequent diversification. To our delight, free *NH* imidazole group innately part of conivaptan (**4j**) was productively leveraged to guide the C‒H alkylation, furnishing the *meta*-functionalised product **5j** in 39% yield. In few cases, unexpected reactivity beyond the anticipated *meta*-C(*sp*^2^)‒H activation selectivity was observed under the reaction conditions, still producing pharmaceutically relevant analogues. For instance, minor bis-alkylated compounds **5c2** and **5f2** could also be isolated after additions to the electron-deficient C(*sp*^2^)–H on the heterocycle of selexipag (**4c**) or the α-amino C(*sp*^3^)–H on the morpholine ring of minaprine (**4f**). Furthermore, the tetrahydroimidazothiazole core of levamisole (**4k**) was oxidised to the corresponding saturated imidazole ring during the transformation, providing the major C5-alkylated product **5k**, although minor bis-functionalised compound **5k2** obtained via *meta*-C–H activation could also be isolated. Finally, instead of reacting at the potential C(*sp*^2^)–H activation sites directed by the 2-aminopyrimidine group of SGI-7079 (**4l**), alkylation at the C5-position of the free *NH* pyrrolopyrimidine scaffold afforded product **5l** while being compatible with strongly chelating cyano and piperazine moieties.

It is worth noting that it would be very difficult to access these late-stage *meta*-C–H alkylated drug derivatives via other synthetic means or by resorting to de novo syntheses with lengthy step count as a consequence. Moreover, this method offers complementarity in site-selectivity to conventional *ortho*-C–H activation strategies, enabling the functionalisation of biologically active candidates remotely from polar directing groups, which can be a desired feature from a medicinal chemistry perspective.

### Pharmaceutical properties modulation

**Fig. 5:**
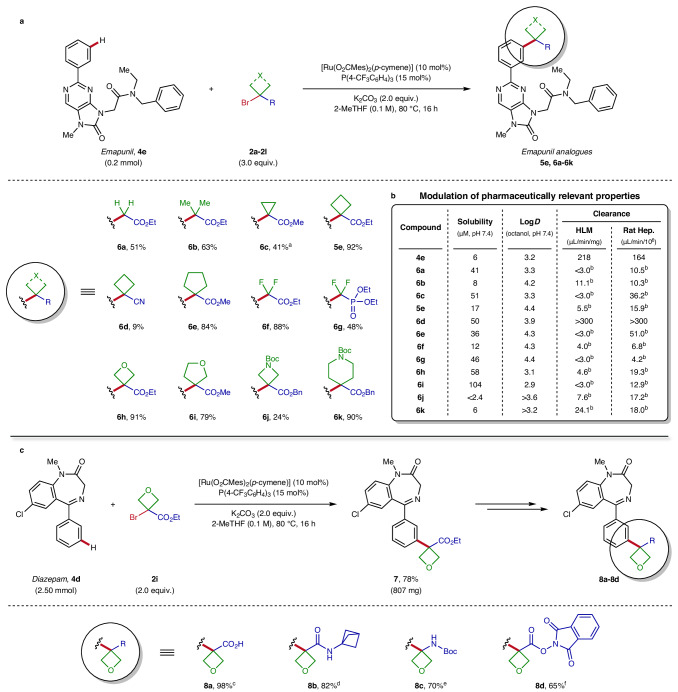
Late-stage pharmaceutical properties modulation and further functional handle diversification. **a** Preparation of emapunil analogues **6a-6k** using various alkyl bromides via ruthenium-catalysed late-stage *meta*-C‒H alkylation. General optimised reaction conditions: emapunil, **4e** (80.3 mg, 0.20 mmol), alkyl bromide **2** (0.60 mmol), [Ru(O_2_CMes)_2_(*p*-cymene)] (11.2 mg, 0.02 mmol), P(4-CF_3_C_6_H_4_)_3_ (14.0 mg, 0.03 mmol) and K_2_CO_3_ (55 mg, 0.40 mmol) in 2-MeTHF (2.0 mL, 0.1 M) at 80 °C for 16 h. ^a^*tert*-amyl alcohol (*t*-AmOH) was used as solvent. **b** Modulation of pharmaceutically relevant properties for *meta*-C–H alkylated emapunil analogues **5e,** **6a-6k** (*n* = 1), see Supplementary Section 5 for more details. ^b^Intrinsic clearance (CL_int_) in human liver microsomes (HLM) or rat hepatocytes (Rat Hep.) was measured on the corresponding free acid to prevent ester hydrolysis upon biological assays. **c** Late-stage diversification of the ester handle on diazepam derivative **7** after *meta*-C–H alkylation (see Supplementary Section 4.4 for more details). General optimised reaction conditions: diazepam, **4d** (712 mg, 2.50 mmol), alkyl bromide **2i** (1045 mg, 5.00 mmol), [Ru(O_2_CMes)_2_(*p*-cymene)] (140.4 mg, 0.250 mmol), P(4-CF_3_C_6_H_4_)_3_ (174.9 mg, 0.375 mmol) and K_2_CO_3_ (691 mg, 5.00 mmol) in 2-MeTHF (25 mL, 0.1 M) at 80 °C for 16 h. ^c^Saponification: NaOH (1.2 equiv.), THF:H_2_O (1:1), r.t., 1.5 h. ^d^Amide coupling: 1-BCP-NH_3_Cl (1.3 equiv.), IBCF (1.2 equiv.), Et_3_N (2.3 equiv.), CH_2_Cl_2_, 0 °C to r.t., 16 h. ^e^Curtius rearrangement: DPPA (1.1 equiv.), Et_3_N (1.4 equiv.), *t*-BuOH, 80 °C, 18 h. ^f^Redox-active ester: NHPI (1.05 equiv.), DIC (1.1 equiv.), DMAP (10 mol%), CH_2_Cl_2_, r.t., 16 h. BCP bicyclo[1.1.1]pentane, Bn benzyl, Boc *tert*-butyloxycarbonyl, DIC *N*,*N*′-diisopropylcarbodiimide, DMAP 4-dimethylaminopyridine, DPPA diphenylphosphoryl azide, IBCF isobutyl chloroformate, Mes mesityl, 2-MeTHF 2-methyltetrahydrofuran, NHPI *N*-hydroxyphthalimide, r.t. room temperature, THF tetrahydrofuran.

Pleased by the ample substrate scope and high functional group tolerance of this procedure, we next investigated its potential for the late-stage modulation of pharmaceutically relevant properties of drug candidates. For this purpose, the anxiolytic drug emapunil (**4e**) was efficiently converted to a wide range of *meta*-C–H alkylated analogues (**6a-6k**) under the optimised reaction conditions (Fig. 5a). Gratifyingly, a multitude of coupling partners (**2a-2l**) bearing small aliphatic groups finding useful applications in medicinal chemistry were selectively introduced in a single step, promptly generating an array of biologically pertinent derivatives in moderate to excellent yields. This includes a variety of acyclic (**6a,** **6b**), carbocyclic (**6c-6e**), fluorinated (**6f,** **6g**) or heterocyclic (**6h-6k**) important motifs in modern drug design, exhibiting favourable structural and physicochemical properties with proven benefits to molecular optimisation^15,35^. In regards to 3- to 6-membered aliphatic rings (cyclopropane **6c**, cyclobutane **6d**, cyclopentane **6e**, oxetane **6h**, tetrahydrofuran **6i**, azetidine **6j** and piperidine **6k**), such advantages usually result from their intrinsic small size and rigidity offering non-planar three-dimensional scaffolds with defined vectors and high fraction of *sp*^3^ carbons (F*sp*^3^). Furthermore, these small ring systems have been successfully applied to provide bioisosteric replacements for various functional groups, typically presenting improved pharmacokinetic properties^14^. Notably, all of the small fragments smoothly installed with our method – especially cyclopropane^36^, oxetane^37^, or fluorine substituents^38^ – have been frequently used in drug discovery for many years as valuable motifs enabling the fine-tuning of key characteristics of a bioactive compound. In particular, oxetanes are attractive rings featuring versatile applications, especially well known as robust units with significantly enhanced metabolic stability^39,40^. Interestingly, primary C–H alkylation showcasing high *meta*-selectivity^41^ was achieved with our approach, furnishing the analogue bearing a methylene bridge **6a** used as a reference for comparison of properties modulation with other aliphatic groups. Apart from ester-containing motifs, different synthetically useful handles offering alternative conjugation strategies can also be incorporated using this LSF approach, as demonstrated with phosphonate (**6g**) or cyano (**6d**) groups, albeit in low yield for the latter. Importantly, orthogonal deprotection conditions (basic, acidic or reductive) can be employed based on the chemical stability of the drug molecule, as illustrated with products **6j** and **6k** bearing simultaneously *tert*-butyloxycarbonyl (Boc) and benzyl (Bn) groups, allowing selective removal for further transformations.

With access to a collection of medicinally relevant analogues from the same chemical series, we then evaluated the capacity of our LSF method to influence key characteristics of a bioactive molecule. To this end, the derivatives **5e,** **6a-6k** obtained via *meta*-C–H alkylation of the benzodiazepine receptor agonist emapunil (**4e**) were subjected to a range of fundamental in vitro drug discovery assays (Fig. 5b). A selection of data including solubility, Log*D*, protein binding and clearance were measured in order to assess the modulation of these pharmaceutically relevant properties upon LSF (see Supplementary Section 5 for details). A general increase in aqueous solubility was observed for the synthesised ester analogues (**5e,** **6a-6k**) in comparison with the parent molecule (**4e**). Nevertheless, useful range of Log*D* values (2.9-4.4) was witnessed for all molecules, reflecting the interesting lipophilic nature of this chemical series. When comparing the reference compound bearing a methylene group (**6a**) with other derivatives, a decrease in hydrophilicity was observed for analogues with high F*sp*^3^ (**6b,** **5e,** **6e**) – although an exception was noticed for cyclopropane ring (**6c**) – or with the introduction of fluorine atoms (**6f**). Notably, a significant increase in solubility correlated with a decrease in Log*D* was observed upon installation of polar 4- and 5-membered heterocycles bearing an oxygen atom in comparison with their carbocyclic ring equivalents (**5e** vs. **6h** and **6e** vs. **6i**). In contrast, a decrease in solubility along with an increase in Log*D* was noticed for nitrogen-containing heterocycles azetidine (**6j**) and piperidine (**6k**) bearing lipophilic Boc and Bn groups. Additionally, very high human plasma protein binding was observed for most of the compounds and no clear trend could be extracted, but significant impact on unbound fractions upon C–H alkylation was determined in few cases (**6a,** **6c,** **6h,** **6i**). As expected, high intrinsic clearance (CL_int_) both in human liver microsomes (HLM) or rat hepatocytes (Rat Hep.) was obtained for the parent molecule (**4e**) and all ester-containing analogues **5e,** **6a-6k**, with values above the detection limit for the latter compounds. In order to compare the metabolic stability between these drug derivatives, their corresponding free acids were prepared and subjected to similar assays (see Supplementary Section 4.3 for details). Pleasingly, these analogues are much more stable and considerably lower clearance was found in all instances. Substantial changes were observed in some cases, notably low values for fluorinated derivatives **6f** and **6g**, but no general trend could be collected from the dataset.

It is worth noting that these findings only concern specific aliphatic substituents installed on this particular series based on emapunil scaffold and thus do not provide a general pattern. However, the overall outcome of this biological evaluation strongly suggests that this late-stage *meta*-C–H alkylation could be considered as a viable strategy to productively influence pharmaceutical properties. Consequently, this LSF approach should be envisaged upon molecular optimisation to improve various cross-parameters such as aqueous solubility, protein binding, lipophilicity, or metabolic stability.

### Functional handle diversification

Aiming to take advantage of the bifunctional alkyl units used as coupling partners bearing a synthetically useful ester group for subsequent transformations, we next turned our attention to the late-stage diversification of this functional handle (Fig. 5c). To this end, the catalytic *meta*-C‒H alkylation of diazepam (**4d**) using the optimised reaction conditions was achieved on a 2.5 mmol scale with reduced stoichiometry of bromooxetane reagent **2i**, providing gram quantities of the desired coupling product **7** in 78% yield. Thus, installation of this reactive ester handle in the drug molecule enabled downstream elaboration by either considering product-oriented (introduction of a specific substituent) or diversity-oriented (incorporation of a transient group for further conjugation) LSF approaches. This was exemplified with few classical synthetic manipulations generating pharmaceutically relevant new analogues **8a-8d** (see Supplementary Section 4.4 for details).

First, saponification of the oxetane ethyl ester compound **7** delivered the corresponding free carboxylic acid derivative **8a** in quantitative yield without the need for chromatography purification. The latter was smoothly converted via amide coupling into the product **8b** containing a bicyclo[1.1.1]pentane (BCP) moiety, ring system that has gained major interest in medicinal chemistry for its application as a saturated benzene bioisostere^42^. Notably, this bridged aliphatic ring is particularly attractive in drug design due to improved pharmacokinetic properties such as solubility and metabolic stability upon replacement of phenyl groups with BCPs. Furthermore, Curtius rearrangement performed on substrate **8a** furnished the useful Boc-protected amino-oxetane derivative **8c** in 70% yield. Interestingly, such aryl amino-oxetane scaffolds are difficult to access due to the lack of available synthetic methods, and provide valuable bioisosteres for ubiquitous benzamide pharmacophores^43^. Pleasingly, the *N*-hydroxyphthalimide (NHPI) ester **8d** was also synthesised in good yield, opening the door to an array of subsequent decarboxylative cross-coupling strategies under thermal, photochemical or electrochemical conditions. Indeed, NHPI redox-active esters have been identified as precursors to a wide variety of functional groups and provide a versatile handle for a multitude of synthetic transformations with LSF applicability^44^. Such a diversity-oriented LSF platform would be of great utility in the context of medicinal chemistry by allowing the expedient generation of bioactive analogues with a view to accelerate SAR studies.

## Discussion

In summary, we have reported a general ruthenium-catalysed *meta*-C(*sp*^2^)–H alkylation protocol with broad applicability in drug design. The use of HTE enabled to quickly access mild reaction conditions offering high levels of selectivity together with ample scope and functional group tolerance, suitable for the LSF of structurally complex molecules. Thus, this transformation was successfully achieved on a wide range of pharmaceuticals using numerous directing groups inherently present in the drug scaffold to productively guide the *meta*-functionalisation. A variety of medicinally relevant bifunctional alkyl units bearing a dual handle allowing geminal C–H activation and subsequent diversification were efficiently employed as coupling partners, including small aliphatic motifs offering beneficial biological properties and applications as saturated functional group bioisosteres. Moreover, we demonstrated the potential of this strategy for the direct modification of an unprotected lead structure to rapidly generate an array of related pharmaceutical analogues in a single step. This obviated the need for lengthy de novo synthesis of each targeted compound, which is of particular interest in terms of sustainability and from the perspective of drug development. Furthermore, this remote C–H alkylation enabled the late-stage modulation of key pharmacokinetic and physicochemical characteristics of bioactive molecules, highlighting the capacity of this powerful procedure to facilitate SAR exploration and expedite molecular optimisation. Consequently, we anticipate that our methodology will find further applications in medicinal chemistry and organic synthesis at large, and we expect this LSF approach to provide significant benefits in drug discovery programmes.

## Methods

### General experimental procedures for *meta*-C–H alkylation

#### General procedure A (standard conditions)

In a glovebox under N_2_ atmosphere, a microwave vial was charged with [Ru(O_2_CMes)_2_(*p*-cymene)] (22.5 mg, 0.04 mmol, 10 mol%), P(4-CF_3_C_6_H_4_)_3_ (28.0 mg, 0.06 mmol, 15 mol%), K_2_CO_3_ (111 mg, 0.80 mmol, 2.0 equiv.) and the appropriate substrate **1** (0.40 mmol, 1.0 equiv.). Then, alkyl bromide **2** (1.20 mmol, 3.0 equiv.) and 2-MeTHF (4.0 mL, 0.1 M) were sequentially added. The vial was sealed, taken out of the glovebox and the reaction mixture was heated to 80 °C. After stirring for 16 hours, the crude reaction mixture was cooled down to room temperature, diluted with EtOAc (10 mL), filtered and analysed by LC-MS. The volatiles were removed under reduced pressure and the residue was purified by automated flash column chromatography. The relevant fractions were collected and concentrated to yield the desired *meta*-alkylated product **3** (Fig. 3).

#### General procedure B (LSF conditions)

On the benchtop, a microwave vial was charged with the appropriate LSF substrate **4** (0.20 mmol, 1.0 equiv.). The vial was moved into a glovebox under N_2_ atmosphere, where [Ru(O_2_CMes)_2_(*p*-cymene)] (11.2 mg, 0.02 mmol, 10 mol%), P(4-CF_3_C_6_H_4_)_3_ (14.0 mg, 0.03 mmol, 15 mol%) and K_2_CO_3_ (55 mg, 0.40 mmol, 2.0 equiv.) were added. Then, alkyl bromide **2** (0.60 mmol, 3.0 equiv.) and 2-MeTHF (2.0 mL, 0.1 M) were sequentially added. The vial was sealed, taken out of the glovebox and the reaction mixture was heated to 80 °C. After stirring for 16 hours, the crude reaction mixture was cooled down to room temperature, diluted with EtOAc (5 mL) and MeOH (5 mL), filtered and analysed by LC-MS. The volatiles were removed under reduced pressure, the residue was dissolved in DMSO (3-5 mL) and purified by preparative reverse phase HPLC. The relevant fractions were collected and lyophilised to yield the desired *meta*-alkylated product **5** or **6 **(Figs. 4–5).

### Reporting summary

Further information on research design is available in the Nature Portfolio Reporting Summary linked to this article.

### Supplementary information

Supplementary Information

Peer Review File

Reporting Summary

## Data Availability

The data generated in this study are provided within the paper and its Supplementary Information files. This includes general information; extended HTE data for the reaction optimisation and LSF substrates screening; experimental procedures and analytical details with characterisation data for all products; further physicochemical and DMPK data with details on the pharmaceutically relevant properties modulation of emapunil analogues; NMR spectra for all compounds. These data are also available from the corresponding authors upon request.

## References

[1] Rogge T (2021). C–H activation. Nat. Rev. Methods Prim..

[2] Sambiagio C (2018). A comprehensive overview of directing groups applied in metal-catalysed C–H functionalisation chemistry. Chem. Soc. Rev..

[3] Meng G (2020). Achieving site-selectivity for C–H activation processes based on distance and geometry: a carpenter’s approach. J. Am. Chem. Soc..

[4] Dutta U, Maiti S, Bhattacharya T, Maiti D (2021). Arene diversification through distal C(sp^2^)−H functionalization. Science.

[5] Fan Z (2022). Molecular editing of aza-arene C–H bonds by distance, geometry and chirality. Nature.

[6] Wencel-Delord J, Glorius F (2013). C–H bond activation enables the rapid construction and late-stage diversification of functional molecules. Nat. Chem..

[7] Guillemard L, Kaplaneris N, Ackermann L, Johansson MJ (2021). Late-stage C–H functionalization offers new opportunities in drug discovery. Nat. Rev. Chem..

[8] Cernak T, Dykstra KD, Tyagarajan S, Vachal P, Krska SW (2016). The medicinal chemist’s toolbox for late stage functionalization of drug-like molecules. Chem. Soc. Rev..

[9] Börgel J, Ritter T (2020). Late-stage functionalization. Chem.

[10] Moir M, Danon JJ, Reekie TA, Kassiou M (2019). An overview of late-stage functionalization in today’s drug discovery. Expert Opin. Drug Discov..

[11] Jana R, Begam HM, Dinda E (2021). The emergence of the C–H functionalization strategy in medicinal chemistry and drug discovery. Chem. Commun..

[12] Dai H-X, Stepan AF, Plummer MS, Zhang Y-H, Yu J-Q (2011). Divergent C–H functionalizations directed by sulfonamide pharmacophores: late-stage diversification as a tool for drug discovery. J. Am. Chem. Soc..

[13] Friis SD, Johansson MJ, Ackermann L (2020). Cobalt-catalysed C–H methylation for late-stage drug diversification. Nat. Chem..

[14] Bauer MR (2021). Put a ring on it: application of small aliphatic rings in medicinal chemistry. RSC Med. Chem..

[15] Grygorenko OO, Volochnyuk DM, Vashchenko BV (2021). Emerging building blocks for medicinal chemistry: recent synthetic advances. Eur. J. Org. Chem..

[16] Meanwell NA (2023). Applications of bioisosteres in the design of biologically active compounds. J. Agric. Food Chem..

[17] Zhang Z, Tang W (2018). Drug metabolism in drug discovery and development. Acta Pharm. Sin. B.

[18] Burkhard JA, Wuitschik G, Rogers-Evans M, Müller K, Carreira EM (2010). Oxetanes as versatile elements in drug discovery and synthesis. Angew. Chem. Int. Ed..

[19] Docherty JH (2023). Transition-metal-catalyzed C–H bond activation for the formation of C–C bonds in complex molecules. Chem. Rev..

[20] Castellino NJ, Montgomery AP, Danon JJ, Kassiou M (2023). Late-stage functionalization for improving drug-like molecular properties. Chem. Rev..

[21] Korvorapun K, Samanta RC, Rogge T, Ackermann L (2021). Remote C–H functionalizations by ruthenium catalysis. Synthesis.

[22] Lam NYS (2022). Empirical guidelines for the development of remote directing templates through quantitative and experimental analyses. J. Am. Chem. Soc..

[23] Dutta U, Maiti D (2022). Emergence of pyrimidine-based *meta*-directing group: journey from weak to strong coordination in diversifying *meta*-C–H functionalization. Acc. Chem. Res..

[24] Huang H-M, Bellotti P, Ma J, Dalton T, Glorius F (2021). Bifunctional reagents in organic synthesis. Nat. Rev. Chem..

[25] Krska SW, DiRocco DA, Dreher SD, Shevlin M (2017). The evolution of chemical high-throughput experimentation to address challenging problems in pharmaceutical synthesis. Acc. Chem. Res..

[26] Mahjour B, Shen Y, Cernak T (2021). Ultrahigh-throughput experimentation for information-rich chemical synthesis. Acc. Chem. Res..

[27] Chen X (2023). Close-shell reductive elimination versus open-shell radical coupling for site-selective ruthenium-catalyzed C−H activations by computation and experiments. Angew. Chem. Int. Ed..

[28] Ruan Z (2017). Ruthenium(II)-catalyzed *meta* C−H mono- and difluoromethylations by phosphine/carboxylate cooperation. Angew. Chem. Int. Ed..

[29] Korvorapun K, Kuniyil R, Ackermann L (2020). Late-stage diversification by selectivity switch in *meta*-C–H activation: evidence for singlet stabilization. ACS Catal..

[30] Pace V, Hoyos P, Castoldi L, Domínguez de María P, Alcántara AR (2012). 2-Methyltetrahydrofuran (2-MeTHF): a biomass-derived solvent with broad application in organic chemistry. ChemSusChem.

[31] Dreher SD, Krska SW (2021). Chemistry informer libraries: conception, early experience, and role in the future of cheminformatics. Acc. Chem. Res..

[32] Weis E, Johansson M, Korsgren P, Martín-Matute B, Johansson MJ (2022). Merging directed C–H activations with high-throughput experimentation: development of iridium-catalyzed C–H aminations applicable to late-stage functionalization. JACS Au.

[33] Vitaku E, Smith DT, Njardarson JT (2014). Analysis of the structural diversity, substitution patterns, and frequency of nitrogen heterocycles among U.S. FDA approved pharmaceuticals. J. Med. Chem..

[34] Blakemore DC (2018). Organic synthesis provides opportunities to transform drug discovery. Nat. Chem..

[35] Wang Y, Haight I, Gupta R, Vasudevan A (2021). What is in our kit? An analysis of building blocks used in medicinal chemistry parallel libraries. J. Med. Chem..

[36] Talele TT (2016). The “Cyclopropyl Fragment” is a versatile player that frequently appears in preclinical/clinical drug molecules. J. Med. Chem..

[37] Bull JA, Croft RA, Davis OA, Doran R, Morgan KF (2016). Oxetanes: recent advances in synthesis, reactivity, and medicinal chemistry. Chem. Rev..

[38] Gillis EP, Eastman KJ, Hill MD, Donnelly DJ, Meanwell NA (2015). Applications of fluorine in medicinal chemistry. J. Med. Chem..

[39] Wuitschik G (2006). Oxetanes as promising modules in drug discovery. Angew. Chem. Int. Ed..

[40] Wuitschik G (2010). Oxetanes in drug discovery: structural and synthetic insights. J. Med. Chem..

[41] Paterson AJ (2017). α-Halo carbonyls enable *meta* selective primary, secondary and tertiary C–H alkylations by ruthenium catalysis. Org. Biomol. Chem..

[42] Subbaiah MAM, Meanwell NA (2021). Bioisosteres of the phenyl ring: recent strategic applications in lead optimization and drug design. J. Med. Chem..

[43] Rojas JJ (2022). Amino-oxetanes as amide isosteres by an alternative defluorosulfonylative coupling of sulfonyl fluorides. Nat. Chem..

[44] Murarka S (2018). *N*-(Acyloxy)phthalimides as redox-active esters in cross-coupling reactions. Adv. Synth. Catal..

