# Supplementary material for: Late-stage meta-C–H alkylation of pharmaceuticals to modulate biological properties and expedite molecular optimisation in a single step
Source: Nat Commun. 2024 Apr 18;15:3349. doi: 10.1038/s41467-024-46697-8 (PMC11026381; doi:10.1038/s41467-024-46697-8)
Supplement: Supplementary file 1 — Supplementary Information [file 41467_2024_46697_MOESM1_ESM.pdf]

Supplementary Information

Late-stage *meta*-C–H alkylation of pharmaceuticals to modulate biological properties and expedite molecular optimisation in a single step

Lucas Guillemard[1], Lutz Ackermann[2,3]* and Magnus J. Johansson[1]*

e-mail: magnus.j.johansson2@astrazeneca.com; lutz.ackermann@chemie.uni-goettingen.de

[1]Medicinal Chemistry, Research and Early Development, Cardiovascular, Renal and Metabolism (CVRM), BioPharmaceuticals R&D, AstraZeneca, Gothenburg, Sweden.

[2]Institut für Organische und Biomolekulare Chemie and Wöhler Research Institute for Sustainable Chemistry (WISCh), Georg-August-Universität Göttingen, Göttingen, Germany.

[3]German Centre for Cardiovascular Research (DZHK), Berlin, Germany.

Table of Contents

Supplementary Methods S3
1. General information S3
2. High-Throughput Experimentation (HTE) S4
2.1. Reaction optimisation S4
2.2. LSF substrates screening S9
3. General experimental procedures for *meta*-C–H alkylation S14
4. Experimental and analytical data for products S15
4.1. Building blocks functionalisation (**3a-3ze**) S15
4.2. Late-stage functionalisation (**5a-5l**) S34
4.3. Emapunil analogues preparation (**6a-6k**) S45
4.4. Synthetic handle diversification (**7**, **8a-8d**) S55
5. Pharmaceutically relevant properties modulation S59
6. NMR spectra (^{1}H, ^{13}C, ^{19}F and ^{31}P) S65
Supplementary References S137

Supplementary Methods

1. General information

General reagent information. Anhydrous solvents were purchased from Sigma Aldrich and sparged with N_2 prior to use. Unless otherwise noted, all reagents were obtained from commercial sources and used as received. The following catalysts and starting materials were synthesised according to previously described literature procedures: [Ru_2Cl_3(*p*-cymene)$_2$](PF_6)[1], [Ru(OAc)$_2$(*p*-cymene)] & [Ru(O_2CMes)$_2$(*p*-cymene)][2], azetidine bromide **2k** & piperidine bromide **2l**[3,4], purine derivative **1k**[5], ester drug derivative **4i**[6]. Reactions in sealed tubes were set up in glovebox under N_2 atmosphere and were run in Biotage microwave vials (2-5 mL) with aluminium caps equipped with septa. Solids were either weighed by hand or using a Mettler Toledo Quantos system for automated solid dispensing.

General purification information. Flash column chromatography purification was performed on a Biotage Isolera automated system with pre-packed silica gel columns (10 or 25 g SiO_2 Sfär HC Duo 20 µm). Unless otherwise stated, purification by preparative reverse phase HPLC was performed on a Kromasil C8 column (10 µm, 250×50 ID mm) with a flow rate of 100 mL/min over 30 or 45 minutes, using acidic mobile phase (A: H_2O/MeCN/HCO_2H = 95/5/0.2; B: MeCN), or alternatively on a XBridge C18 column (10 µm, 250×50 ID mm) with a flow rate of 100 mL/min over 30 or 45 minutes, using basic mobile phase (A: H_2O/MeCN = 95/5, 10 mM NH_4HCO_3; B: MeCN). UV detector Gilson UV/VIS-155 was used for UV-triggered collection of fractions at 254 nm wavelength.

General analytical information. Analytical thin-layer chromatography (TLC) was performed on precoated aluminium silica gel plates and the components were visualised by UV light absorbance at 254 nm. Analytical LC-MS was carried out on a Waters Acquity UPLC system with a HSS C18 column (1.8 µm, 50×2.1 ID mm) using an acidic mobile phase at pH 3 (A: H_2O, 10 mM HCO_2H, 1 mM NH_3; B: MeCN/H_2O = 95/5), or alternatively with a BEH C18 column (1.7 µm, 50×2.1 ID mm) using a basic mobile phase at pH 10 (A: H_2O, 5 mM NH_4HCO_3, 50 mM NH_3; B: MeCN/H_2O = 95/5). All new compounds were characterised by NMR spectroscopy and high-resolution mass spectrometry (HRMS). Nuclear magnetic resonance spectra (^{1}H, ^{13}C, ^{19}F, ^{31}P, COSY, NOESY, ROESY, HSQC and HMBC) were recorded on a Bruker Ultrashield 500 MHz spectrometer with a Bruker Cryo Platform. NMR data is reported as follows: chemical shift (multiplicity [s = singlet, d = doublet, t = triplet, q = quartet, p = pentet, h = heptet, m = multiplet and br = broad]), coupling constant (in Hz) and integration. Chemical shifts for ^{1}H & ^{13}C NMR spectra were reported in parts per million (ppm) with the residual solvent resonance as internal reference ($CDCl_3$: δ = 7.26 ppm & 77.16 ppm, DMSO-*d*$_6$: δ = 2.50 ppm & 39.52 ppm). ^{13}C, ^{19}F and ^{31}P NMR spectra were recorded with complete proton decoupling. High resolution mass spectrometry (HRMS) data was recorded on a Waters Acquity UPLC System equipped with Acquity PDA and XEVO-QTOF mass spectrometer using electrospray ionisation (ESI) in positive mode. A linear gradient (5-95%) was run with a flow of 0.8 mL/min for 3.2 min at 45 °C either using an acidic mobile phase at pH 3 (A: H_2O, 10 mM HCO_2H, 1 mM HCO_2NH_4; B: MeCN/H_2O = 95/5, 10 mM HCO_2H, 1 mM HCO_2NH_4) on a Waters Acquity UPLC HSS C18 column (1.8 µm, 50×2.1 ID mm); or alternatively using a basic mobile phase at pH 10 (A: H_2O, 5 mM NH_4HCO_3, 50 mM NH_3; B: MeCN/H_2O = 95/5, 5 mM NH_4HCO_3, 50 mM NH_3) on a Waters Acquity Premier BEH C18 column (1.7 µm, 50×2.1 ID mm). Relative absorbance was recorded at 230 nm (acidic, pH 3), or alternatively at 210 nm (basic, pH 10).

General software information. NMR data were collected using TopSpin v3 and IconNMR v5, and analysed using MestReNova v14. UPLC-MS data were collected and analysed using MassLynx v4. Heat maps were generated and data were visualised using TIBCO Spotfire v11.

2. High-Throughput Experimentation (HTE)

2.1. Reaction optimisation

Selected data obtained during the optimisation of reaction conditions for this ruthenium-catalysed *meta*-C–H alkylation is shown below in **Supplementary Figs. 1-4**. The reaction mixtures were analysed by LC-MS and the data shown is based on the ratio of relative intensities of product peak(s) *vs.* substrate peak in the UV chromatogram; *i.e.* total conversion to products [mono + bis + tri-functionalisation (with *ortho-* and/or *meta*-selectivity)]. When appropriate, levels of mono-functionalisation with *meta*-selectivity (desired product) are also indicated, based on the ratio of the relative intensity of desired product peak *vs.* other regioisomeric product peaks in the UV chromatogram.

*Note: To confirm the connectivity of products throughout the HTE optimisation campaign, reactions were performed using the general optimised conditions on 0.40 mmol substrate scale (16 times larger), and the desired products were characterised after purification (main text **Fig. 3**).*

General procedure for reaction optimisation

The reactions were set-up using 24- or 96-wells Para-dox® plates with 1 mL glass vials equipped with magnetic stirrer bars, performed on a 25 µmol substrate scale. In a glovebox under N_2 atmosphere, the appropriate vials were charged with all solid reagents (substrates **1**, bases, additives and catalysts) using a Mettler Toledo Quantos system for automated solid dispensing. Then, alkyl bromides **2** and any other liquid reagents were subsequently added to the vials as stock solutions in the required solvents (final concentration of 0.1 M). The plates were sealed with a Teflon film, taken out of the glovebox, and heated at 80 °C under stirring (600 rpm). After 16 hours, the reaction mixtures were allowed to cool down at room temperature, diluted with DMSO (500 µL) and a scoop of SiliaMetS® Thiol metal scavenger (Si-SH from SiliCycle, 40-63 µm, 1.34 mmol/g) was added in each vial. The reaction mixtures were stirred (900 rpm) at room temperature for 1 hour, then a 200 µL aliquot was extracted from each vial and transferred to a 96-well Greiner_V plastic microplate. The solids were centrifuged using Eppendorf Centrifuge 5810 R (room temperature, atmospheric pressure, 3500 rpm, 15 min), then a 20 µL aliquot was extracted from each well (upper part) and transferred to a new 96-well Greiner_V plastic microplate. Finally, the wells were diluted with further DMSO (180 µL) and the reaction mixtures were analysed by LC-MS both under acidic and basic and conditions [Waters Acquity UPLC system, HSS C18 column with a mobile phase at pH 3 (A: H_2O/MeCN/HCO_2H = 95/5/0.2, B: MeCN) and BEH C18 column with a mobile phase at pH 10 (A: H_2O/MeCN/NH_3 = 95/5/0.2, B: MeCN)].

Substrates, alkyl bromides and additives

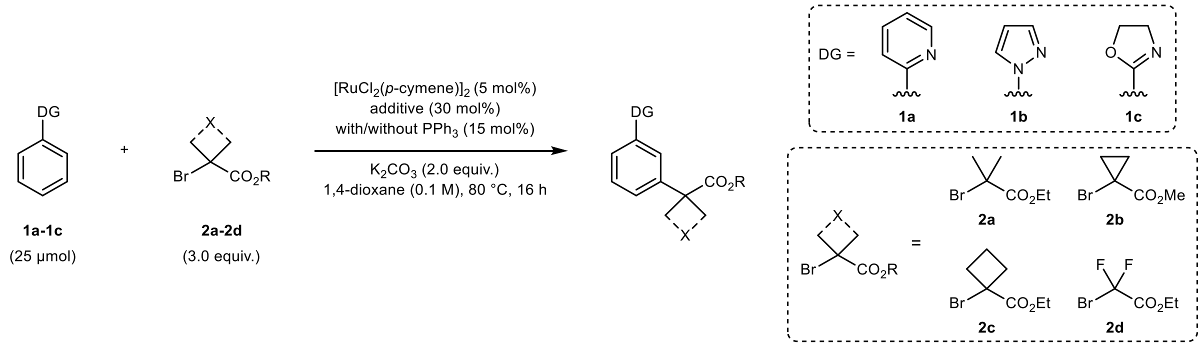

**Substrate**		**Alkyl bromide**		**Additive**		**PPh₃**	
**1–4**	1a	**1, 5, 9**	2a	**A, E**	KOAc	**A–D**	without
**5–8**	1b	**2, 6, 10**	2b	**B, F**	$MesCO_2H$	**E–H**	with
**9–12**	1c	**3, 7, 11**	2c	**C, G**	$(PhO)_2PO_2H$		
		**4, 8, 12**	2d	**D, H**	$AdCO_2H$		

a)

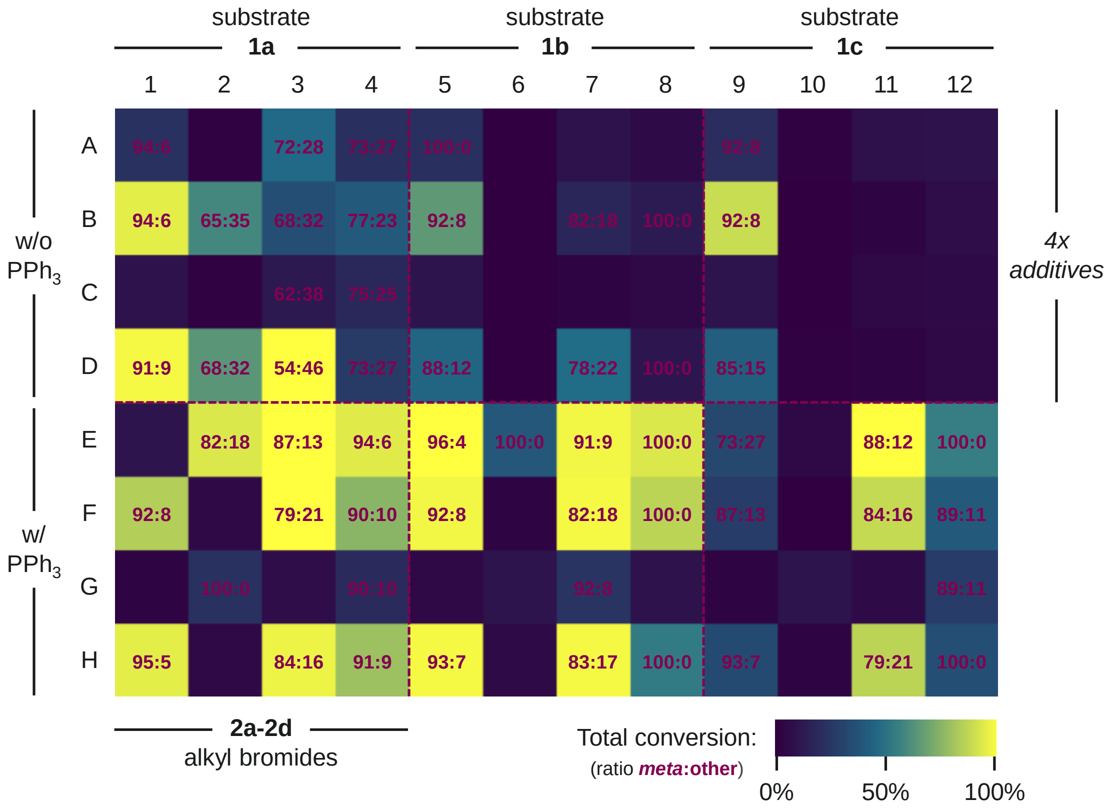

Supplementary Fig. 1. Evaluation of ruthenium catalytic systems for the *meta*-C–H alkylation against a combination of 3 directing groups and 4 coupling partners, using a selection of 4 additives in the presence/absence of phosphine ligand. **a)** Heat map visualising both total conversion (%) and *meta*-selectivity (level of desired mono-functionalised product). Conversion (%): determined by LC-MS based on relative intensities of product peak(s) *vs.* substrate peak in the UV chromatogram. Selectivity (*meta*:other): determined by LC-MS based on relative intensity of desired product peak *vs.* other product peak(s) in the UV chromatogram; this ratio is indicated on the relevant wells (not shown for conversion < 10%). Ac = acetyl, Ad = 1-adamantyl, DG = directing group, Mes = mesityl, w/ = with, w/o = without.

Substrates, alkyl bromides and additives

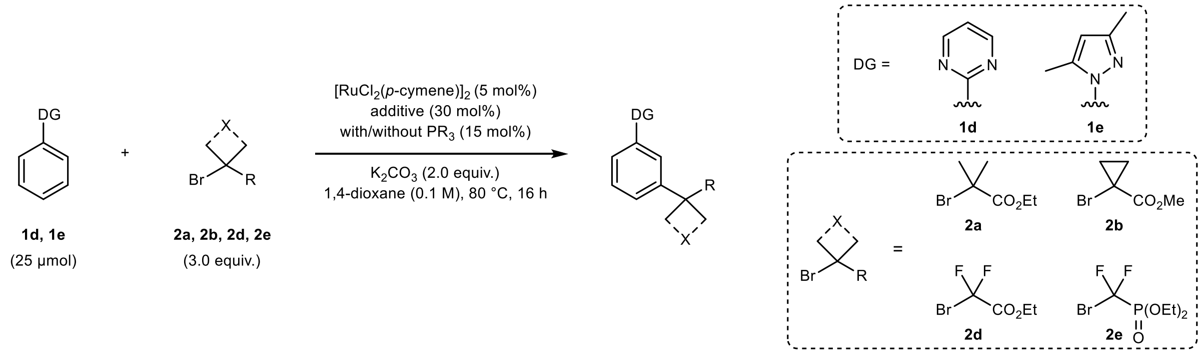

Substrate		Alkyl bromide		Additive		PR_3	
**A–D**	1d	**1–3**	2a	**1, 4, 7, 10**	KOAc	**A, E**	–
**E–H**	1e	**4–6**	2b	**2, 5, 8, 11**	$MesCO_2H$	**B, F**	PPh_3
		**7–9**	2d	**3, 6, 9, 12**	$AdCO_2H$	**C, G**	$P(4\text{-}CF_3C_6H_4)_3$
		**10–12**	2e			**D, H**	$P(4\text{-}MeOC_6H_4)_3$

a)

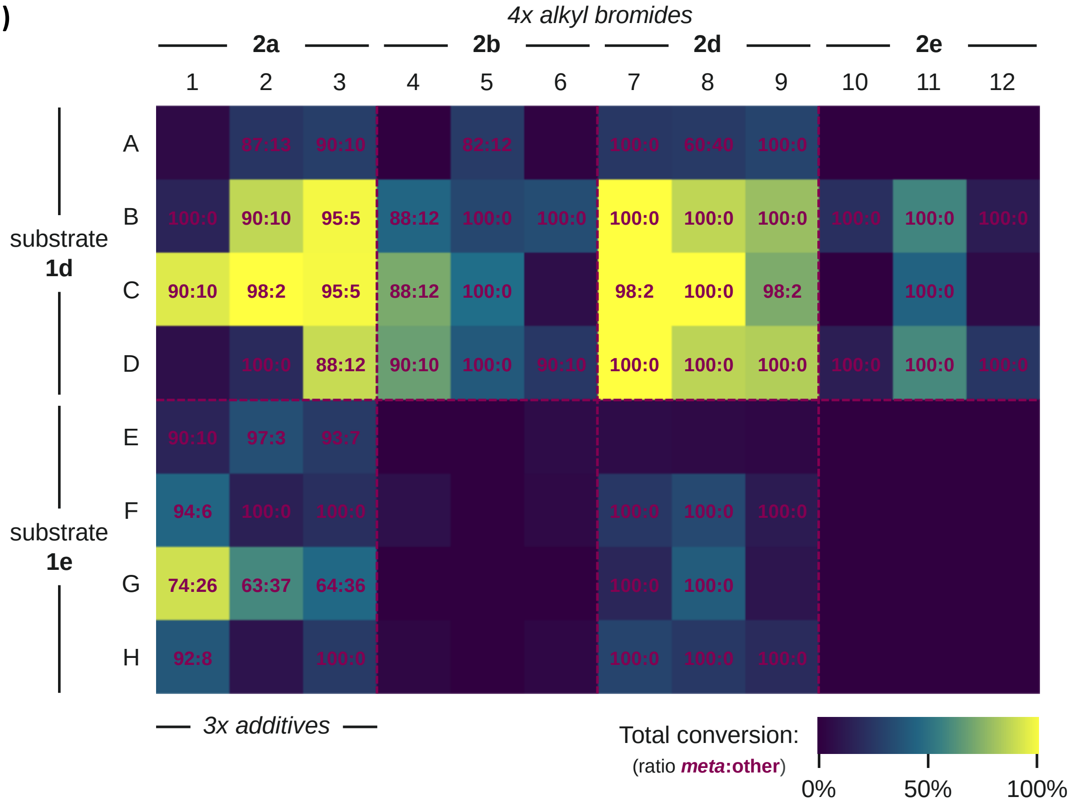

Supplementary Fig. 2. Evaluation of ruthenium catalytic systems for the *meta*-C–H alkylation against a combination of 2 directing groups and 4 coupling partners, using a selection of 3 additives in the presence/absence of 3 phosphine ligands. **a)** Heat map visualising both total conversion (%) and *meta*-selectivity (level of desired mono-functionalised product). Conversion (%): determined by LC-MS based on relative intensities of product peak(s) *vs.* substrate peak in the UV chromatogram. Selectivity (*meta*:other): determined by LC-MS based on relative intensity of desired product peak *vs.* other product peak(s) in the UV chromatogram; this ratio is indicated on the relevant wells (not shown for conversion < 10%). Ac = acetyl, Ad = 1-adamantyl, DG = directing group, Mes = mesityl.

Alkyl bromides, solvents and additives

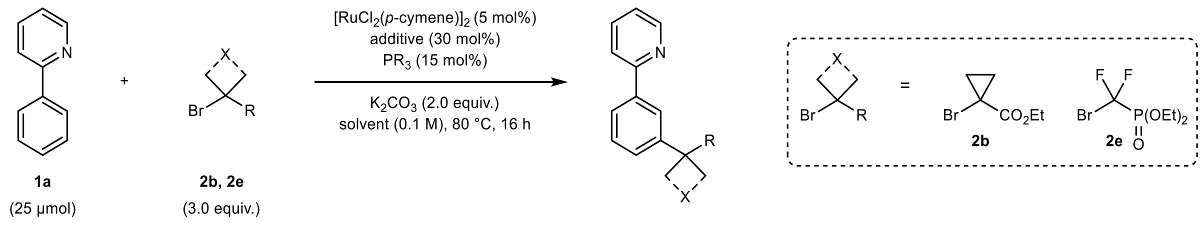

Alkyl bromide		Solvent		Additive		PR_3	
**A–D**	2b	**1–3**	1,4-dioxane	**A, E**	KOAc	**1, 4, 7, 10**	PPh_3
**E–H**	2e	**4–6**	2-MeTHF	**B, F**	$MesCO_2H$	**2, 5, 8, 11**	$P(4\text{-}CF_3C_6H_4)_3$
		**7–9**	toluene	**C, G**	$(PhO)_2PO_2H$	**3, 6, 9, 12**	$P(4\text{-}MeOC_6H_4)_3$
		**10–12**	*t*-AmOH	**D, H**	$AdCO_2H$		

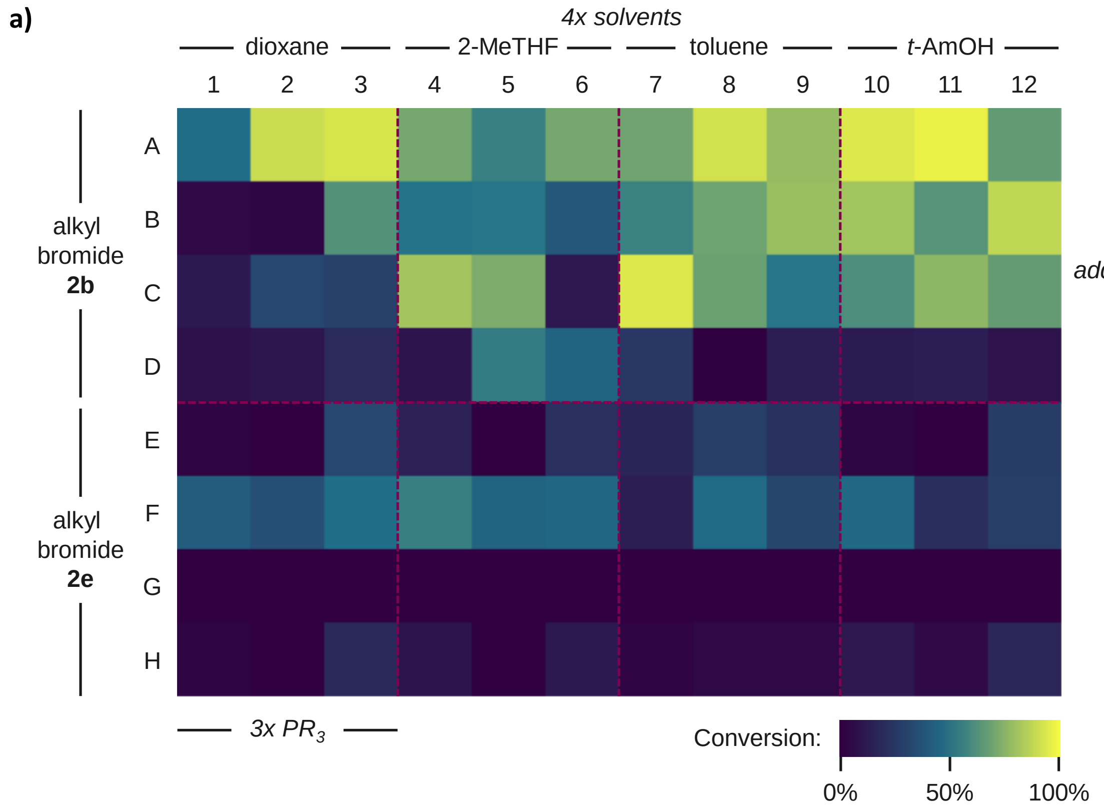

Supplementary Fig. 3. Evaluation of ruthenium catalytic systems for the *meta*-C–H alkylation against 2 coupling partners using a selection of 4 solvents and 4 additives in the presence of 3 phosphine ligands. **a)** Heat map visualising conversion (%): determined by LC-MS based on relative intensity of product peak *vs.* substrate peak in the UV chromatogram. Only *meta*-selectivity (desired mono-functionalised product) was obtained during this screen and the formation of other regioisomeric products was not observed. Ac = acetyl, Ad = 1-adamantyl, Mes = mesityl, 2-MeTHF = 2-methyltetrahydrofuran, *t*-AmOH = *tert*-amyl alcohol.

Substrates, catalysts and additives

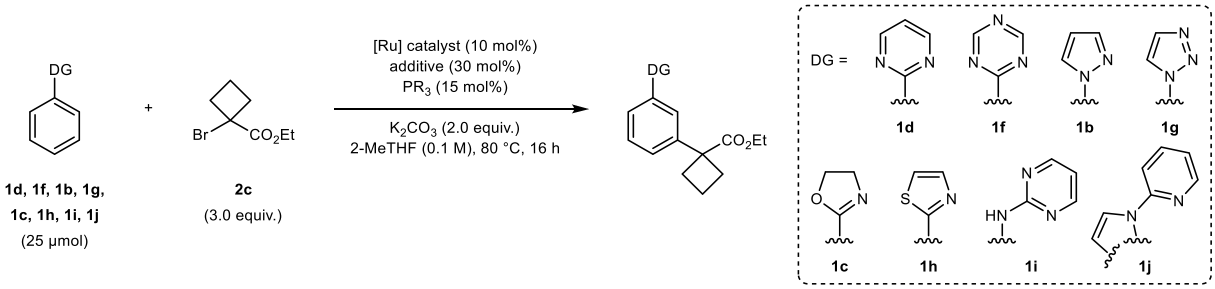

Substrate	
**A**	1d
**B**	1f
**C**	1b
**D**	1g
**E**	1c
**F**	1h
**G**	1i
**H**	1j

[Ru] catalyst	
**1–4**	$[RuCl_2(p\text{-cymene})]_2$
**5–8**	$[Ru_2Cl_3(p\text{-cymene})_2](PF_6)$
**9, 10**	$[Ru(OAc)_2(p\text{-cymene})]$
**11, 12**	$[Ru(O_2CMes)_2(p\text{-cymene})]$

Additive	
**1, 2, 5, 6**	KOAc
**3, 4, 7, 8**	$MesCO_2H$
**9–12**	–

PR_3	
**1, 3, 5, 7, 9, 11**	PPh_3
**2, 4, 6, 8, 10, 12**	$P(4\text{-}CF_3C_6H_4)_3$

a)

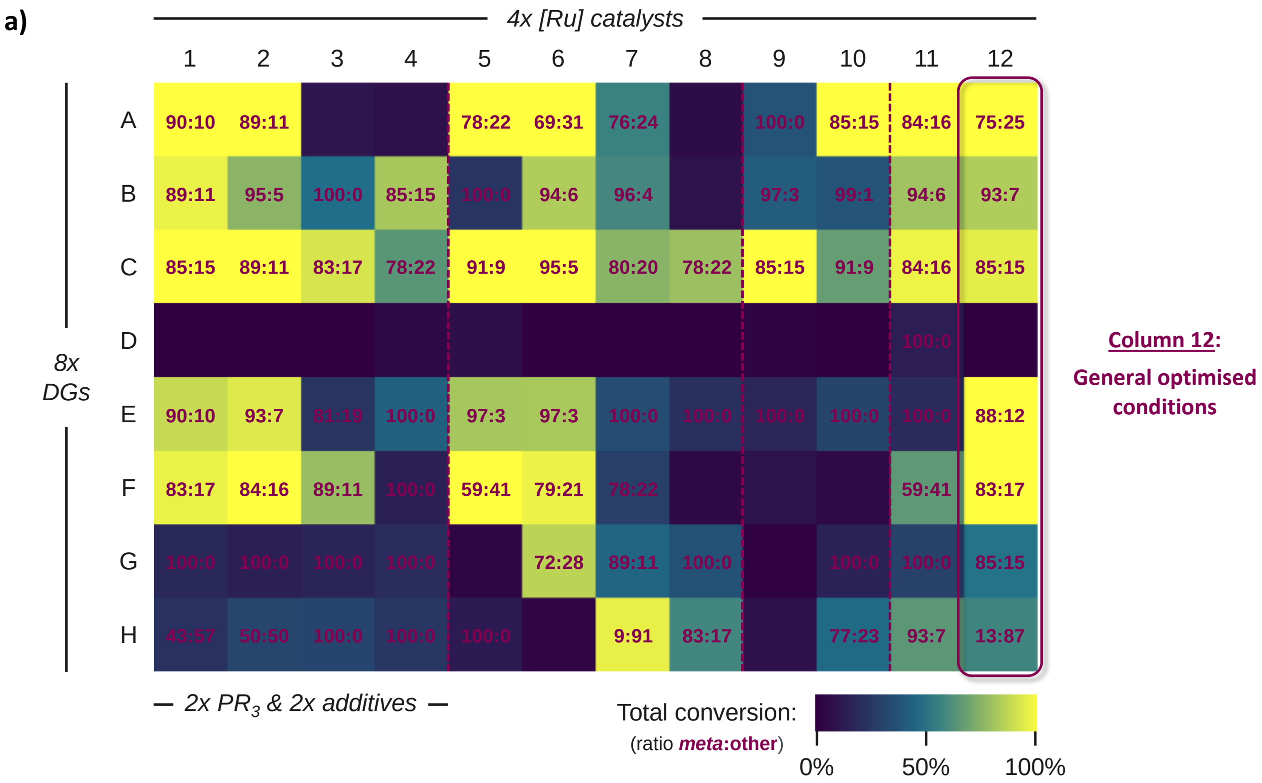

Supplementary Fig. 4. Evaluation of ruthenium catalytic systems for the *meta*-C–H alkylation against 8 directing groups using a selection of 4 catalysts and 2 additives in the presence of 2 phosphine ligands. **a)** Heat map visualising both total conversion (%) and *meta*-selectivity (level of desired mono-functionalised product). Conversion (%): determined by LC-MS based on relative intensities of product peak(s) *vs.* substrate peak in the UV chromatogram. Selectivity (*meta*:other): determined by LC-MS based on relative intensity of desired product peak *vs.* other product peak(s) in the UV chromatogram; this ratio is indicated on the relevant wells (not shown for conversion < 10%). HTE studies led to general optimised reaction conditions highlighted in column 12, using $[Ru(O_2CMes)_2(p\text{-cymene})]$ as catalyst in combination with $P(4\text{-}CF_3C_6H_4)_3$ ligand, and K_2CO_3 as base in 2-MeTHF at 80 °C. Ac = acetyl, DG = directing group, Mes = mesityl, 2-MeTHF = 2-methyltetrahydrofuran.

2.2. LSF substrates screening

A library of 36 commercially available drugs was evaluated in this *meta*-C–H alkylation, using alkyl bromide **2c** (ethyl 1-bromocyclobutane-1-carboxylate) as coupling partner. This panel of bioactive compounds was selected based on the available functional group(s) inherently present in their structure to direct the *meta*-C–H activation, identified to be successful throughout the HTE optimisation campaign (main text **Fig. 3**). Each drug molecule was screened in a 96-wells Para-dox® plate against the general optimised reaction conditions as shown below in **Supplementary Figs. 5-8**. The reaction mixtures were analysed by LC-MS and the data shown is based on the ratio of relative intensities of product peak(s) *vs.* LSF substrate peak (and other major by-products) in the UV chromatogram; *i.e.* relative product abundance. When appropriate, levels of mono-functionalisation are also indicated, based on the ratio of the relative intensity of the mono-functionalised product peak *vs.* bis-functionalisation peak in the UV chromatogram.

*Note: To determine the identity of products obtained with > 25% conversion during the LSF screening, reactions were performed using the general optimised conditions on 0.20 mmol LSF substrate scale (10 times larger), and the C–H alkylated products (**5a-5l**) were characterised after purification (main text **Fig. 4b**).*

Experimental procedure

The reactions were set-up using a 96-wells Para-dox® plate with 1 mL glass vials equipped with magnetic stirrer bars, performed on a 20 µmol substrate scale. On the benchtop, the vials were manually charged with the appropriate LSF substrates **4** (20 µmol, 1.0 equiv.). The plate was moved into a glovebox under N_2 atmosphere, where all solid reagents were added – [Ru(O_2CMes)$_2$(*p*-cymene)] (1.12 mg, 2.0 µmol, 10 mol%), P(4-$CF_3C_6H_4$)$_3$ (1.40 mg, 3.0 µmol, 15 mol%) and K_2CO_3 (5.53 mg, 40 µmol, 2.0 equiv.) – using a Mettler Toledo Quantos system for automated solid dispensing. Then, ethyl 1-bromocyclobutane-1-carboxylate **2c** (12.42 mg, 60 µmol, 3.0 equiv.) was subsequently added to the vials as a stock solution in 2-methyltetrahydrofuran (2-MeTHF, 200 µL, 0.1 M). The plate was sealed with a Teflon film, taken out of the glovebox and heated at 80 °C under stirring (600 rpm). After 16 hours, the reaction mixtures were allowed to cool down at room temperature, diluted with DMSO (400 µL) and a scoop of SiliaMetS® Thiol metal scavenger (Si-SH from SiliCycle, 40-63 µm, 1.34 mmol/g) was added in each vial. The reaction mixtures were stirred (900 rpm) at room temperature for 1 hour, then a 200 µL aliquot was extracted from each vial and transferred to a 96-well Greiner_V plastic microplate. The solids were centrifuged using Eppendorf Centrifuge 5810 R (room temperature, atmospheric pressure, 3500 rpm, 15 min), then a 20 µL aliquot was extracted from each well (upper part) and transferred to a new 96-well Greiner_V plastic microplate. Finally, the wells were diluted with further DMSO (180 µL) and the reaction mixtures were analysed by LC-MS both under acidic and basic and conditions [Waters Acquity UPLC system, HSS C18 column with a mobile phase at pH 3 (A: H_2O/MeCN/HCO_2H = 95/5/0.2, B: MeCN) and BEH C18 column with a mobile phase at pH 10 (A: H_2O/MeCN/NH_3 = 95/5/0.2, B: MeCN)].

LSF screen under general optimised conditions

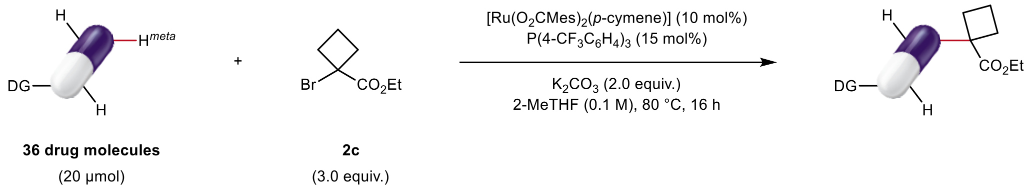

**LSF substrates**			
**A1**	Vismodegib	**C1**	GW6471
**A2**	Lumacaftor*	**C2**	Oxaprozin*
**A3**	Pritelivir	**C3**	Parecoxib
**A4**	GSK1292263	**C4**	Valdecoxib
**A5**	Atazanavir	**C5**	Ataluren*
**A6**	Selexipag	**C6**	Amenamevir
**A7**	Emapunil	**C7**	Suvorexant
**A8**	Sildenafil	**C8**	Levamisole
**A9**	Minaprine	**C9**	Febuxostat*
**B1**	Bemcentinib	**D1**	Zolpidem
**B2**	Clozapine	**D2**	Conivaptan
**B3**	Diazepam	**D3**	Capmatinib
**B4**	Sulfaphenazole	**D4**	Talniflumate
**B5**	Apixaban	**D5**	Rilpivirine
**B6**	Celecoxib	**D6**	Momelotinib
**B7**	Niraparib	**D7**	Pazopanib
**B8**	Pyraclostrobin	**D8**	SGI-7079
**B9**	Rimonabant	**D9**	Nilotinib

* corresponding methyl or ethyl ester

a)

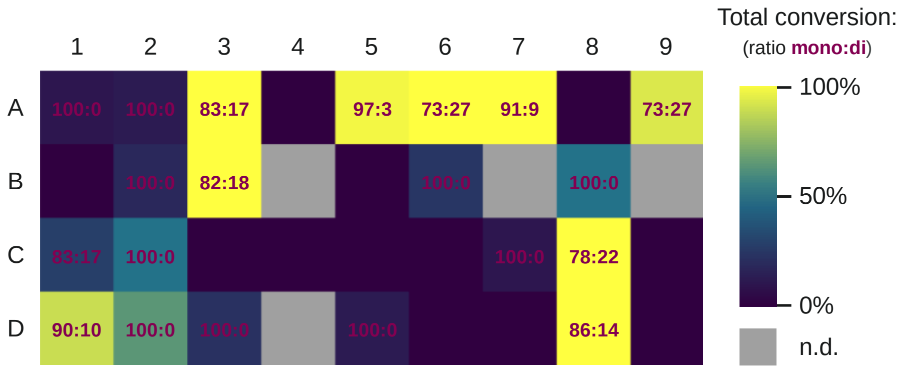

Supplementary Fig. 5. LSF substrates screen under the general optimised reaction conditions, using a selection of 36 drug molecules with inherent directing groups for the *meta*-C–H alkylation. **a)** Heat map visualising both total conversion and level of mono-functionalisation. Conversion (%): determined by LC-MS based on relative intensities of product peak(s) *vs.* LSF substrate peak in the UV chromatogram. Selectivity (mono:di): determined by LC-MS based on relative intensity of mono-functionalised product peak *vs.* bis-functionalised product peak in the UV chromatogram; this ratio is indicated on the relevant wells. n. d. = not determined due to degradation or significant by-product(s) formation. DG = directing group, Mes = mesityl, 2-MeTHF = 2-methyltetrahydrofuran. **Continue page S11**.

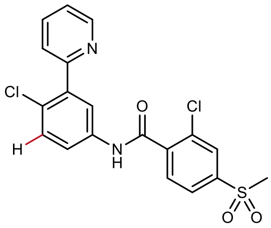

A1 - ***Vismodegib***
[5%] – (100:0)

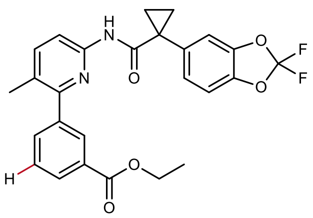

A2 - ***Lumacaftor****
[6%] – (100:0)

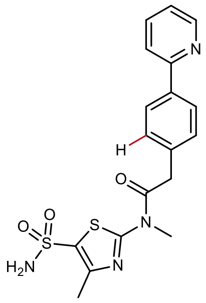

A3 - ***Pritelivir***
[**100%**] – (83:17)

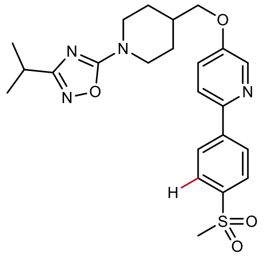

A4 - ***GSK1292263***
[n. r.]

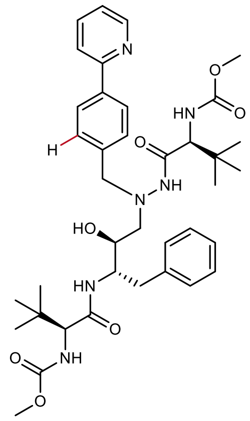

A5 - ***Atazanavir***
[**96%**] – (97:3)

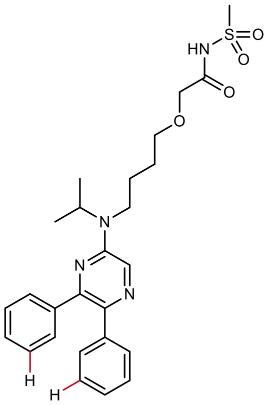

A6 - ***Selexipag***
[**100%**] – (73:27)

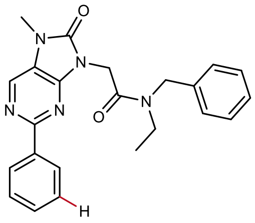

A7 - ***Emapunil***
[**100%**] – (91:9)

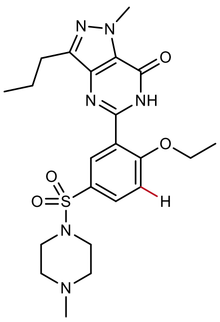

A8 - ***Sildenafil***
[traces]

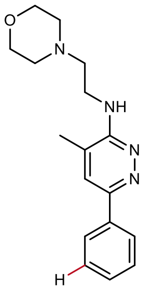

A9 - ***Minaprine***
[**88%**] – (73:27)

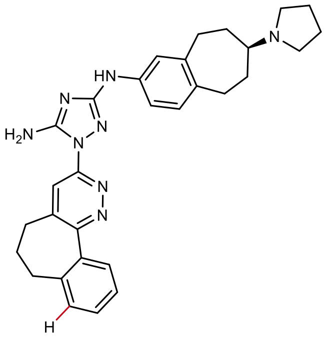

B1 - ***Bemcentinib***
[traces]

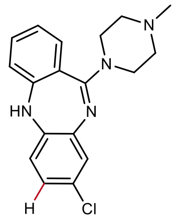

B2 - ***Clozapine***
[9%] – (100:0)

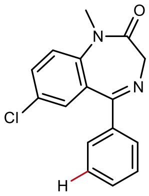

B3 - ***Diazepam***
[**100%**] – (82:18)

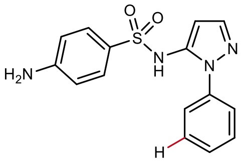

B4 - ***Sulfaphenazole***
[n. d., degradation]

Supplementary Fig. 6. LSF substrates screen under the general optimised reaction conditions, using a selection of 36 drug molecules with inherent directing groups for the *meta*-C–H alkylation. The reactions were analysed by LC-MS and the data shown in square brackets is based on relative intensities of product peak(s) *vs.* LSF substrate peak and other major by-products in the UV chromatogram; *i.e.* [conversion]. Level of mono-functionalisation is also indicated in round brackets when appropriate, determined by LC-MS based on relative intensity of mono-functionalised product peak *vs.* bis-functionalised product peak in the UV chromatogram; *i.e.* (mono:di ratio). For each LSF substrate, potential site(s) for the directed *meta*-C–H alkylation are depicted in red.*: corresponding ethyl ester derivative. n. r. = no reaction (only unreacted starting material remaining). n. d. = not determined due to degradation or significant by-product(s) formation. **Continue page S12**.

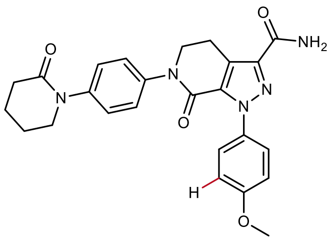

B5 - *Apixaban*

[n. r.]

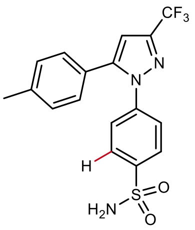

B6 - *Celecoxib*

[12%] – (100:0)

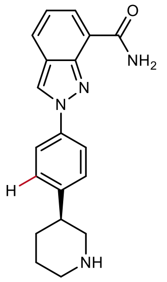

B7 - *Niraparib*

[n. d., degradation]

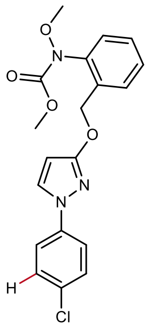

B8 - *Pyraclostrobin*

[26%][a] – (100:0)

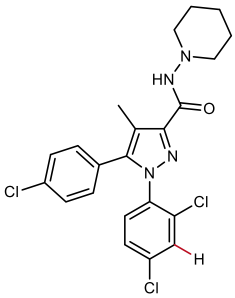

B9 - *Rimonabant*

[n. d., degradation][b]

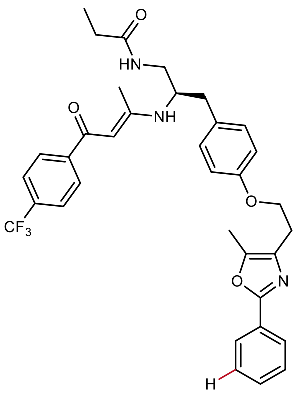

C1 - *GW6471*

[14%] – (83:17)

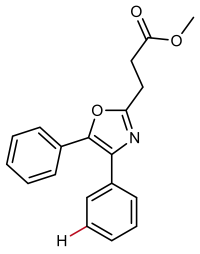

C2 - *Oxaprozin**

[**26%**] – (100:0)

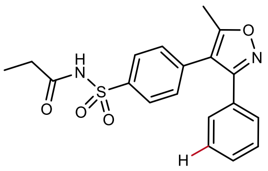

C3 - *Parecoxib*

[traces]

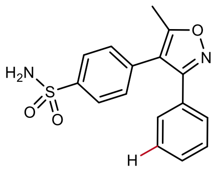

C4 - *Valdecoxib*

[n. r.]

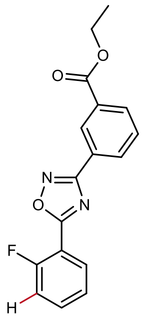

C5 - *Ataluren**

[n. r.]

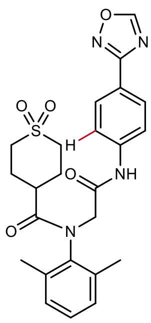

C6 - *Amenamevir*

[traces]

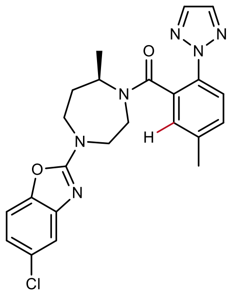

C7 - *Suvorexant*

[5%] – (100:0)

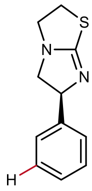

C8 - *Levamisole*

[**100%**][c] – (78:22)

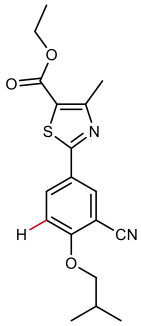

C9 - *Febuxostat**

[n. r.]

Supplementary Fig. 7. LSF substrates screen under the general optimised reaction conditions, using a selection of 36 drug molecules with inherent directing groups for the *meta*-C–H alkylation. The reactions were analysed by LC-MS and the data shown in square brackets is based on relative intensities of product peak(s) *vs.* LSF substrate peak and other major by-products in the UV chromatogram; *i.e.* [conversion]. Level of mono-functionalisation is also indicated in round brackets when appropriate, determined by LC-MS based on relative intensity of mono-functionalised product peak *vs.* bis-functionalised product peak in the UV chromatogram; *i.e.* (mono:di ratio). For each LSF substrate, potential site(s) for the directed *meta*-C–H alkylation are depicted in red.*: corresponding methyl or ethyl ester derivative. n. r. = no reaction (only unreacted starting material remaining). n. d. = not determined due to degradation or significant by-product(s) formation. [a] Cleavage of the N–OMe bond of both starting material and product occurred during the transformation. [b] Peak consistent with loss of piperidine after cleavage of N–N bond was observed by LC-MS. [c] Oxidation and formation of the corresponding imidazole core occurred during the transformation. **Continue page S13**.

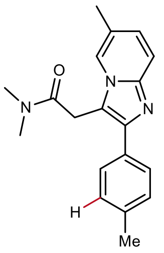

D1 - ***Zolpidem***

[**82%**] – (90:10)

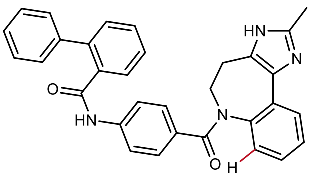

D2 - ***Conivaptan***

[**45%**] – (100:0)

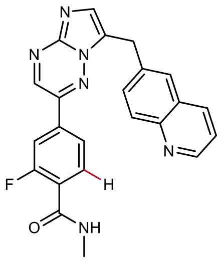

D3 - ***Capmatinib***

[11%] – (100:0)

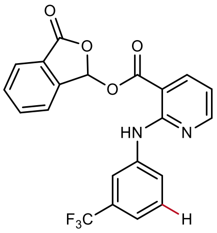

D4 - ***Talniflumate***

[n. d., degradation][d]

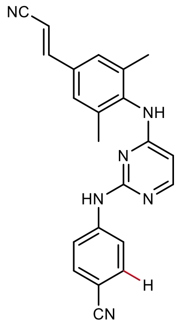

D5 - ***Rilpivirine***

[6%] – (100:0)

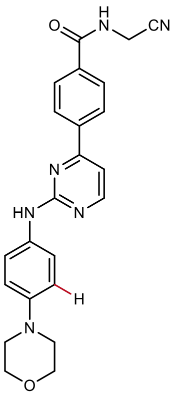

D6 - ***Momelotinib***

[n. r.]

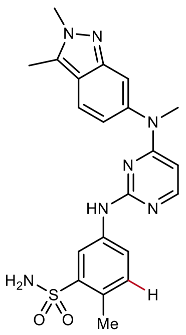

D7 - ***Pazopanib***

[traces]

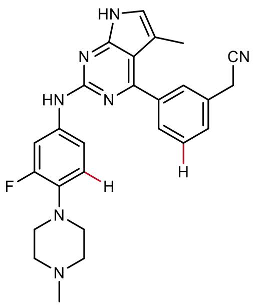

D8 - ***SGI-7079***

[**100%**] – (86:14)

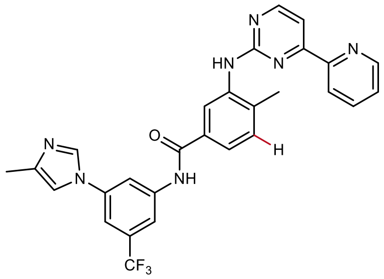

D9 - ***Nilotinib***

[traces]

Supplementary Fig. 8. LSF substrates screen under the general optimised reaction conditions, using a selection of 36 drug molecules with inherent directing groups for the *meta*-C–H alkylation. The reactions were analysed by LC-MS and the data shown in square brackets is based on relative intensities of product peak(s) *vs.* LSF substrate peak and other major by-products in the UV chromatogram; *i.e.* [conversion]. Level of mono-functionalisation is also indicated in round brackets when appropriate, determined by LC-MS based on relative intensity of mono-functionalised product peak *vs.* bis-functionalised product peak in the UV chromatogram; *i.e.* (mono:di ratio). For each LSF substrate, potential site(s) for the directed *meta*-C–H alkylation are depicted in red. n. r. = no reaction (only unreacted starting material remaining). n. d. = not determined due to degradation or significant by-product(s) formation. [d] Peaks consistent with acetal hydrolysis and formation of the corresponding carboxylic acids were observed by LC-MS.

3. General experimental procedures for *meta*-C–H alkylation

General Procedure A (standard conditions, main text **Fig. 3**)

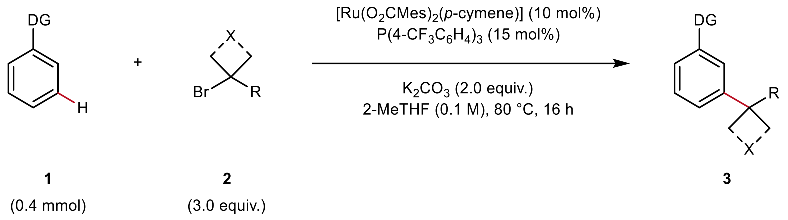

In a glovebox under N_2 atmosphere, a 5 mL crimp-cap microwave vial equipped with a magnetic stirrer bar was charged with [Ru(O_2CMes)$_2$(*p*-cymene)] (22.5 mg, 0.04 mmol, 10 mol%), P(4-$CF_3C_6H_4$)$_3$ (28.0 mg, 0.06 mmol, 15 mol%), K_2CO_3 (111 mg, 0.80 mmol, 2.0 equiv.) and the appropriate substrate **1** (0.40 mmol, 1.0 equiv.). Then, alky bromide **2** (1.20 mmol, 3.0 equiv.) and 2-methyltetrahydrofuran (2-MeTHF, 4.0 mL, 0.1 M) were sequentially added. The vial was sealed, taken out of the glovebox and the reaction mixture was heated to 80 °C. After stirring for 16 hours, the crude reaction mixture was cooled down to room temperature, diluted with EtOAc (10 mL), filtered and analysed by LC-MS. The volatiles were removed under reduced pressure and the residue was purified by automated flash column chromatography on silica gel. The relevant fractions were collected and concentrated *in vacuo* to yield the desired *meta*-alkylated product **3**.

General Procedure B (LSF conditions, main text **Figs. 4b-5a**)

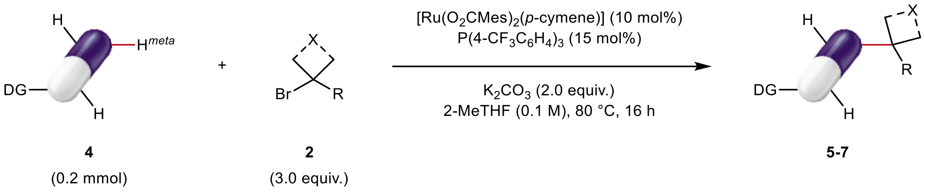

On the benchtop, a 2 mL crimp-cap microwave vial equipped with a magnetic stirrer bar was charged with the appropriate LSF substrate **4** (0.20 mmol, 1.0 equiv.). The vial was moved into a glovebox under N_2 atmosphere, where [Ru(O_2CMes)$_2$(*p*-cymene)] (11.2 mg, 0.02 mmol, 10 mol%), P(4-$CF_3C_6H_4$)$_3$ (14.0 mg, 0.03 mmol, 15 mol%) and K_2CO_3 (55 mg, 0.40 mmol, 2.0 equiv.) were added. Then, alky bromide **2** (0.60 mmol, 3.0 equiv.) and 2-methyltetrahydrofuran (2-MeTHF, 2.0 mL, 0.1 M) were sequentially added. The vial was sealed, taken out of the glovebox and the reaction mixture was heated to 80 °C. After stirring for 16 hours, the crude reaction mixture was cooled down to room temperature, diluted with EtOAc (5 mL) and MeOH (5 mL), filtered and analysed by LC-MS. The volatiles were removed under reduced pressure, the residue was dissolved in DMSO (3-5 mL) and purified by preparative reverse phase HPLC. The relevant fractions were collected and lyophilised to yield the desired *meta*-alkylated product **5** or **6**.

4. Experimental and analytical data for products

4.1. Building blocks functionalisation (3a-3ze)

Ethyl 2-methyl-2-(3-(pyridin-2-yl)phenyl)propanoate (3a)

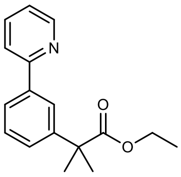

Prepared according to **General Procedure A**, using 2-phenylpyridine (57.2 µL, 0.40 mmol) as substrate and ethyl 2-bromo-2-methylpropanoate (176 µL, 1.20 mmol) as alkyl bromide. The crude reaction mixture was analysed by LC-MS using an acidic mobile phase at pH 3 and the UV chromatograms are shown below:

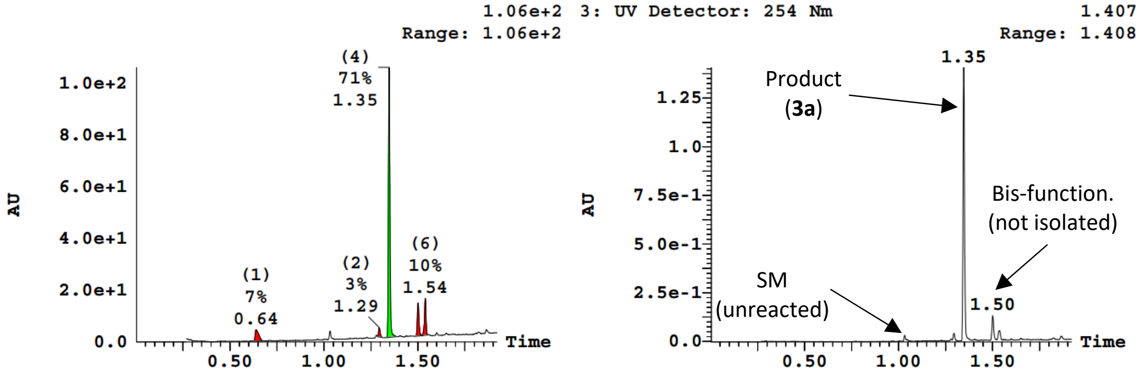

Supplementary Fig. 9. UV chromatograms (TIC & 254 nm) of the crude reaction mixture forming product **3a**.

Purification by automated flash column chromatography (0-15% EtOAc in *n*-heptane, 25 g SiO_2). **3a** was isolated as a colourless oil (89.4 mg, 83%). **^{1}H NMR** (500 MHz, $CDCl_3$) δ (ppm): 8.69 (ddd, *J* = 4.8, 1.8, 1.0 Hz, 1H), 7.99 (t, *J* = 2.0 Hz, 1H), 7.84 (dt, *J* = 7.5, 1.5 Hz, 1H), 7.74 (ddd, *J* = 8.0, 7.2, 1.8 Hz, 1H), 7.71 (dt, *J* = 8.0, 1.3 Hz, 1H), 7.43 (t, *J* = 7.5 Hz, 1H), 7.39 (ddd, *J* = 7.8, 2.0, 1.4 Hz, 1H), 7.23 (ddd, *J* = 7.2, 4.8, 1.4 Hz, 1H), 4.14 (q, *J* = 7.1 Hz, 2H), 1.65 (s, 6H), 1.19 (t, *J* = 7.1 Hz, 3H). **^{13}C NMR** (126 MHz, $CDCl_3$) δ (ppm): 176.8, 157.7, 149.8, 145.5, 139.7, 136.8, 128.8, 126.6, 125.4, 124.3, 122.2, 120.8, 61.0, 46.7, 26.7 (2C), 14.2. **HRMS** (ESI): *m/z* calcd. for $C_{17}H_{19}NO_2$ $[M+H]^+$: 270.1494, found: 270.1497. The analytical data are in accordance with previously reported literature values[7].

Methyl 1-(3-(pyridin-2-yl)phenyl)cyclopropane-1-carboxylate (3b)

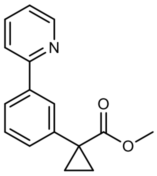

Prepared according to **General Procedure A**, using 2-phenylpyridine (57.2 µL, 0.40 mmol) as substrate, methyl 1-bromocyclopropane-1-carboxylate (124 µL, 1.20 mmol) as alkyl bromide and *tert*-amyl alcohol (*t*-AmOH, 4.0 mL, 0.1 M) as solvent. The crude reaction mixture was analysed by LC-MS using an acidic mobile phase at pH 3 and the UV chromatograms are shown below:

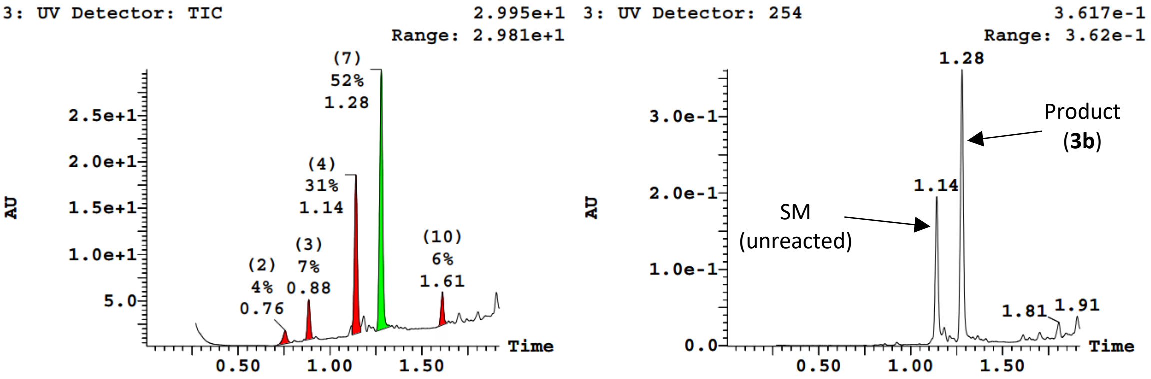

Supplementary Fig. 10. UV chromatograms (TIC & 254 nm) of the crude reaction mixture forming product **3b**.

Purification by automated flash column chromatography (0-20% EtOAc in *n*-heptane, 25 g SiO_2). **3b** was isolated as a colourless oil (62.1 mg, 61%). **^{1}H NMR** (500 MHz, $CDCl_3$) δ (ppm): 8.72 – 8.67 (m, 1H), 8.01 (t, *J* = 2.0 Hz, 1H), 7.86 (dt, *J* = 6.8, 2.1 Hz, 1H), 7.77 – 7.71 (m, 2H), 7.45 – 7.39 (m, 2H), 7.23 (ddd, *J* = 6.2, 4.8, 2.3 Hz, 1H), 3.62 (s, 3H), 1.65 (q, *J* = 4.0 Hz, 2H), 1.27 (q, *J* = 4.0 Hz, 2H). **^{13}C NMR** (126 MHz, $CDCl_3$) δ (ppm): 175.1, 157.3, 149.7, 140.2, 139.4, 136.9, 131.4, 129.2, 128.7, 125.9, 122.3, 120.8, 52.5, 29.2, 16.9 (2C). **HRMS** (ESI): *m/z* calcd. for $C_{16}H_{15}NO_2$ $[M+H]^+$: 254.1181, found: 254.1193.

Ethyl 1-(3-(pyridin-2-yl)phenyl)cyclobutane-1-carboxylate (3c)

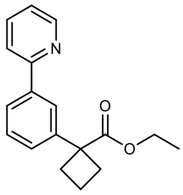

Prepared according to **General Procedure A**, using 2-phenylpyridine (57.2 µL, 0.40 mmol) as substrate and ethyl 1-bromocyclobutane-1-carboxylate (194 µL, 1.20 mmol) as alkyl bromide. The crude reaction mixture was analysed by LC-MS using an acidic mobile phase at pH 3 and the UV chromatograms are shown below:

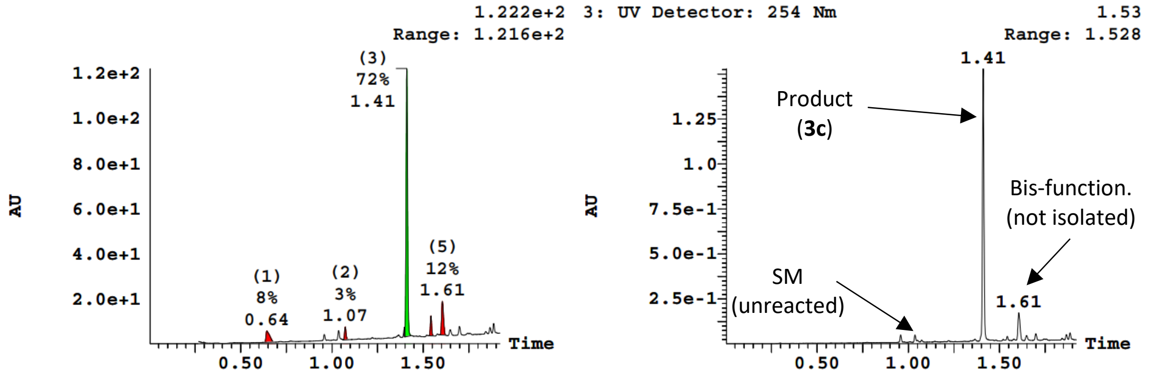

Supplementary Fig. 11. UV chromatograms (TIC & 254 nm) of the crude reaction mixture forming product **3c**.

Purification by automated flash column chromatography (0-15% EtOAc in *n*-heptane, 25 g SiO_2). **3c** was isolated as a colourless oil (97.3 mg, 86%). **^{1}H NMR** (500 MHz, $CDCl_3$) δ (ppm): 8.69 (ddd, *J* = 4.8, 1.7, 1.0 Hz, 1H), 7.92 (t, *J* = 1.8 Hz, 1H), 7.86 (ddd, *J* = 7.7, 1.8, 1.2 Hz, 1H), 7.77 – 7.69 (m, 2H), 7.43 (t, *J* = 7.7 Hz, 1H), 7.36 (ddd, *J* = 7.7, 1.9, 1.2 Hz, 1H), 7.22 (ddd, *J* = 6.6, 4.8, 1.8 Hz, 1H), 4.11 (q, *J* = 7.1 Hz, 2H), 2.93 – 2.84 (m, 2H), 2.63 – 2.55 (m, 2H), 2.12 – 2.01 (m, 1H), 1.95 – 1.85 (m, 1H), 1.17 (t, *J* = 7.1 Hz, 3H). **^{13}C NMR** (126 MHz, $CDCl_3$) δ (ppm): 176.0, 157.6, 149.8, 144.4, 139.5, 136.8, 128.7, 127.1, 125.3, 124.9, 122.2, 120.8, 61.0, 52.6, 32.5 (2C), 16.8, 14.2. **HRMS** (ESI): *m/z* calcd. for $C_{18}H_{19}NO_2$ $[M+H]^+$: 282.1494, found: 282.1484. The analytical data are in accordance with previously reported literature values[8].

Ethyl 2,2-difluoro-2-(3-(pyridin-2-yl)phenyl)acetate (3d)

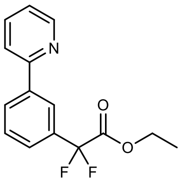

Prepared according to **General Procedure A**, using 2-phenylpyridine (57.2 µL, 0.40 mmol) as substrate and ethyl 2-bromo-2,2-difluoroacetate (154 µL, 1.20 mmol) as alkyl bromide. The crude reaction mixture was analysed by LC-MS using an acidic mobile phase at pH 3 and the UV chromatograms are shown below:

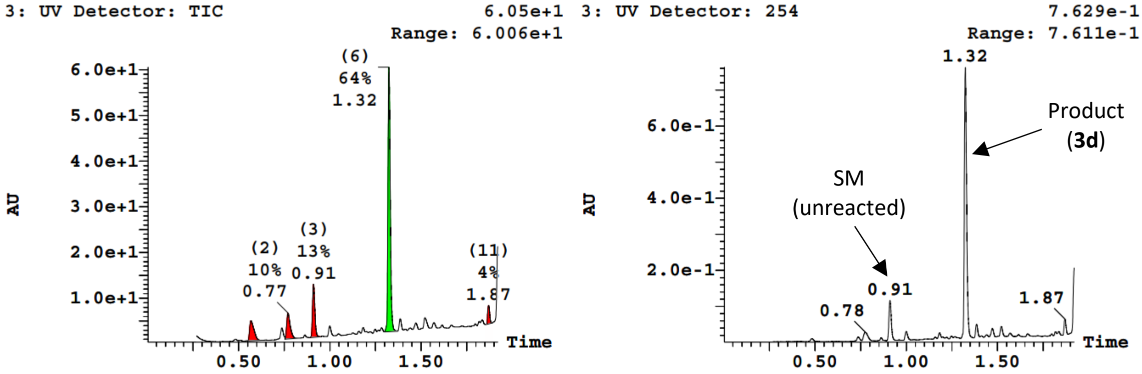

Supplementary Fig. 12. UV chromatograms (TIC & 254 nm) of the crude reaction mixture forming product **3d**.

Purification by automated flash column chromatography (0-20% EtOAc in *n*-heptane, 25 g SiO_2). **3d** was isolated as a colourless oil (82.2 mg, 74%). **^{1}H NMR** (500 MHz, $CDCl_3$) δ (ppm): 8.72 (ddd, *J* = 4.8, 1.8, 1.1 Hz, 1H), 8.23 (t, *J* = 2.1 Hz, 1H), 8.16 (ddt, *J* = 7.6, 1.7, 0.9 Hz, 1H), 7.81 – 7.74 (m, 2H), 7.66 (ddt, *J* = 7.8, 1.8, 1.0 Hz, 1H), 7.57 (tdd, *J* = 7.8, 1.5, 0.8 Hz, 1H), 7.28 (ddd, *J* = 6.8, 4.9, 1.7 Hz, 1H), 4.31 (q, *J* = 7.1 Hz, 2H), 1.31 (t, *J* = 7.1 Hz, 3H). **^{13}C NMR** (126 MHz, $CDCl_3$) δ (ppm): 164.3 (t, $^2J_{C-F}$ = 35.3 Hz), 156.3, 150.0, 140.1, 137.1, 133.6 (t, $^2J_{C-F}$ = 25.5 Hz), 129.6, (t, $^4J_{C-F}$ = 1.8 Hz), 129.3, 126.1 (t, $^3J_{C-F}$ = 6.1 Hz), 124.1 (t, $^3J_{C-F}$ = 6.3 Hz), 122.8, 120.8, 113.5 (t, $^1J_{C-F}$ = 252.4 Hz), 63.3, 14.0. **^{19}F NMR** (471 MHz, $CDCl_3$) δ (ppm): -103.7 (s, 2F). **HRMS** (ESI): *m/z* calcd. for $C_{15}H_{13}F_2NO_2$ $[M+H]^+$: 278.0992, found: 278.0996. The analytical data are in accordance with previously reported literature values[7].

Diethyl (difluoro(3-(pyridin-2-yl)phenyl)methyl)phosphonate (3e)

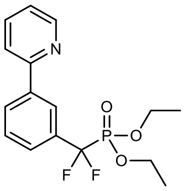

Prepared according to **General Procedure A**, using 2-phenylpyridine (57.2 µL, 0.40 mmol) as substrate and diethyl (bromodifluoromethyl)phosphonate (213 µL, 1.20 mmol) as alkyl bromide. The crude reaction mixture was analysed by LC-MS using an acidic mobile phase at pH 3 and the UV chromatograms are shown below:

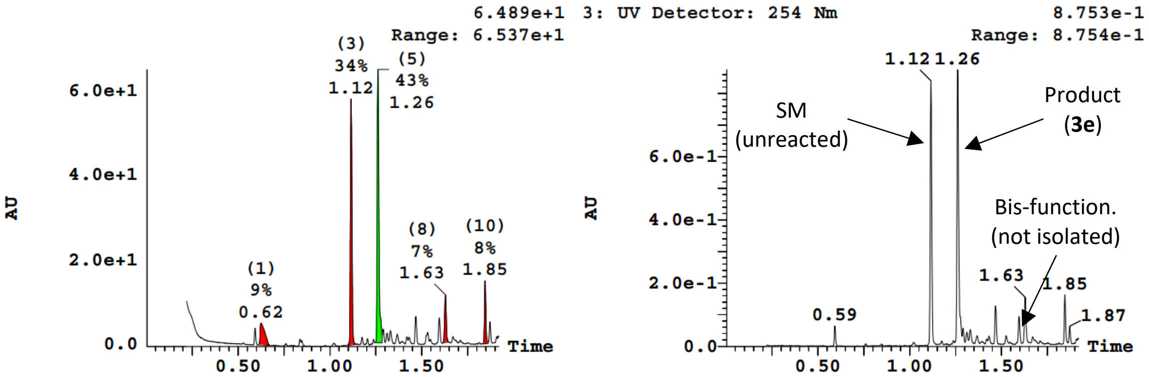

Supplementary Fig. 13. UV chromatograms (TIC & 254 nm) of the crude reaction mixture forming product **3e**.

Purification by automated flash column chromatography (0-50% EtOAc in *n*-heptane, 25 g SiO_2). **3e** was isolated as a colourless oil (63.6 mg, 46%). **^{1}H NMR** (500 MHz, $CDCl_3$) δ (ppm): 8.71 (dt, J = 4.8, 1.4 Hz, 1H), 8.24 (t, J = 1.9 Hz, 1H), 8.17 (ddt, J = 7.8, 2.1, 1.0 Hz, 1H), 7.80 – 7.75 (m, 2H), 7.68 (dd, J = 7.7, 1.7 Hz, 1H), 7.57 (t, J = 7.8 Hz, 1H), 7.28 – 7.24 (m, 1H), 4.28 – 4.12 (m, 4H), 1.32 (td, J = 7.0, 0.6 Hz, 6H). **^{13}C NMR** (126 MHz, $CDCl_3$) δ (ppm): 156.4, 149.9, 139.9, 137.0, 133.3 (td, $J_{C\text{-}F,C\text{-}P}$ = 21.9, 13.7 Hz), 129.4 (td, $J_{C\text{-}F,C\text{-}P}$ = 2.0, 1.9 Hz), 129.1, 126.8 (td, $J_{C\text{-}F,C\text{-}P}$ = 6.8, 2.3 Hz), 124.8 (td, $J_{C\text{-}F,C\text{-}P}$ = 7.0, 2.5 Hz), 122.7, 120.7, 118.2 (td, $J_{C\text{-}F,C\text{-}P}$ = 263.5, 217.8 Hz), 65.0 (d, $J_{C\text{-}P}$ = 6.8 Hz, 2C), 16.5 (d, $J_{C\text{-}P}$ = 5.5 Hz, 2C). **^{19}F NMR** (471 MHz, $CDCl_3$) δ (ppm): -108.3 (d, $J_{F\text{-}P}$ = 115.6 Hz, 2F). **^{31}P NMR** (203 MHz, $CDCl_3$) δ (ppm): 6.31 (t, $J_{P\text{-}F}$ = 115.9 Hz, 1P). **HRMS** (ESI): *m/z* calcd. for $C_{16}H_{18}F_2NO_3P$ $[M+H]^+$: 342.1071, found: 342.1073.

Ethyl 2-methyl-2-(3-(pyrimidin-2-yl)phenyl)propanoate (3f)

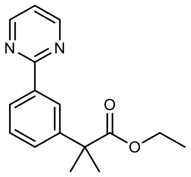

Prepared according to **General Procedure A**, using 2-phenylpyrimidine (62.5 mg, 0.40 mmol) as substrate and ethyl 2-bromo-2-methylpropanoate (176 µL, 1.20 mmol) as alkyl bromide. The crude reaction mixture was analysed by LC-MS using an acidic mobile phase at pH 3 and the UV chromatograms are shown below:

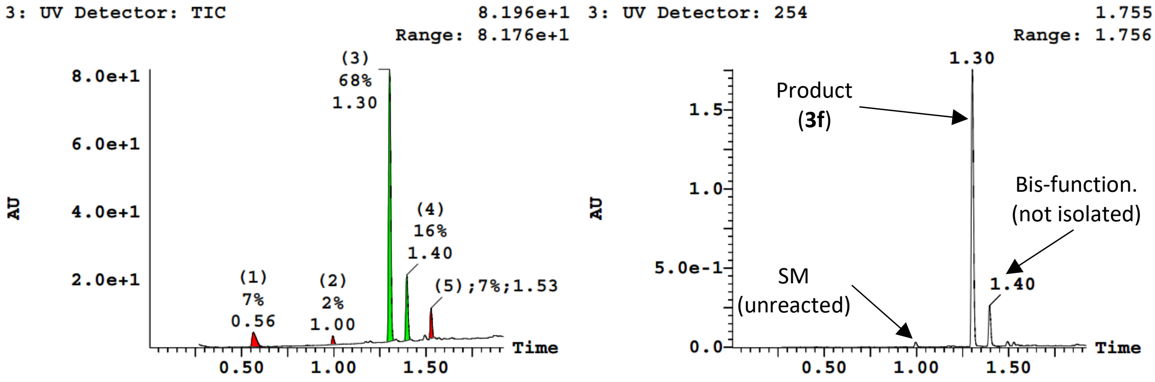

Supplementary Fig. 14. UV chromatograms (TIC & 254 nm) of the crude reaction mixture forming product **3f**.

Purification by automated flash column chromatography (0-20% EtOAc in *n*-heptane, 25 g SiO_2). **3f** was isolated as a colourless oil (94.8 mg, 88%). **^{1}H NMR** (500 MHz, $CDCl_3$) δ (ppm): 8.80 (d, J = 4.8 Hz, 2H), 8.48 (dt, J = 1.7, 1.1 Hz, 1H), 8.32 (ddd, J = 6.2, 2.5, 1.7 Hz, 1H), 7.48 – 7.42 (m, 2H), 7.18 (t, J = 4.8 Hz, 1H), 4.14 (q, J = 7.1 Hz, 2H), 1.66 (s, 6H), 1.18 (t, J = 7.1 Hz, 3H). **^{13}C NMR** (126 MHz, $CDCl_3$) δ (ppm): 176.8, 164.9, 157.3 (2C), 145.4, 137.8, 128.7, 128.5, 126.6, 125.5, 119.2, 61.0, 46.8, 26.7 (2C), 14.2. **HRMS** (ESI): *m/z* calcd. for $C_{16}H_{18}N_2O_2$ $[M+H]^+$: 271.1447, found: 271.1449. The analytical data are in accordance with previously reported literature values[9].

Methyl 1-(3-(pyrimidin-2-yl)phenyl)cyclopropane-1-carboxylate (3g)

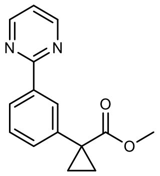

Prepared according to **General Procedure A**, using 2-phenylpyrimidine (62.5 mg, 0.40 mmol) as substrate, methyl 1-bromocyclopropane-1-carboxylate (124 µL, 1.20 mmol) as alkyl bromide and *tert*-amyl alcohol (*t*-AmOH, 4.0 mL, 0.1 M) as solvent. The crude reaction mixture was analysed by LC-MS using an acidic mobile phase at pH 3 and the UV chromatograms are shown below:

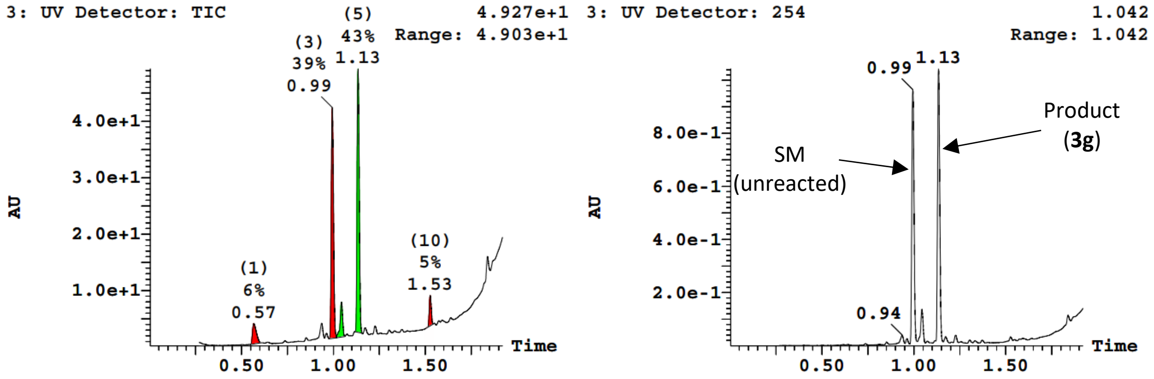

Supplementary Fig. 15. UV chromatograms (TIC & 254 nm) of the crude reaction mixture forming product **3g**.

Purification by automated flash column chromatography (0-20% EtOAc in *n*-heptane, 25 g SiO_2). **3g** was isolated as a white solid (53.4 mg, 52%). **^{1}H NMR** (500 MHz, $CDCl_3$) δ (ppm): 8.81 (d, *J* = 4.8 Hz, 2H), 8.43 (td, *J* = 1.8, 0.6 Hz, 1H), 8.36 (dt, *J* = 7.5, 1.6 Hz, 1H), 7.48 (dt, *J* = 7.6, 1.6 Hz, 1H), 7.44 (td, *J* = 7.6, 0.6 Hz, 1H), 7.19 (t, *J* = 4.8 Hz, 1H), 3.62 (s, 3H), 1.66 (q, *J* = 4.0 Hz, 2H), 1.28 (q, *J* = 4.0 Hz, 2H). **^{13}C NMR** (126 MHz, $CDCl_3$) δ (ppm): 175.1, 164.6, 157.3 (2C), 140.1, 137.6, 133.3, 130.3, 128.6, 127.2, 119.3, 52.5, 29.2, 17.0 (2C). **HRMS** (ESI): *m/z* calcd. for $C_{15}H_{14}N_2O_2$ $[M+H]^+$: 255.1133, found: 255.1131.

Ethyl 1-(3-(pyrimidin-2-yl)phenyl)cyclobutane-1-carboxylate (3h)

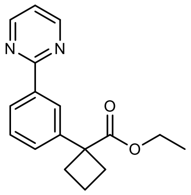

Prepared according to **General Procedure A**, using 2-phenylpyrimidine (62.5 mg, 0.40 mmol) as substrate and ethyl 1-bromocyclobutane-1-carboxylate (194 µL, 1.20 mmol) as alkyl bromide. The crude reaction mixture was analysed by LC-MS using an acidic mobile phase at pH 3 and the UV chromatograms are shown below:

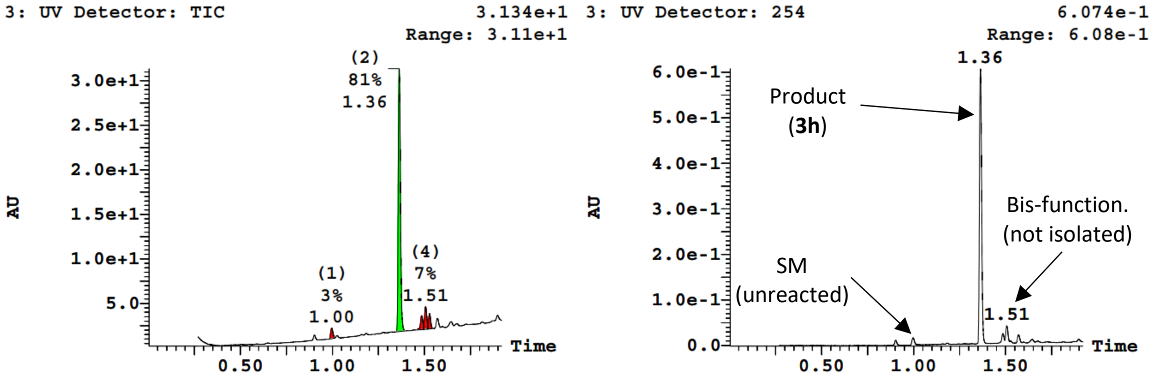

Supplementary Fig. 16. UV chromatograms (TIC & 254 nm) of the crude reaction mixture forming product **3h**.

Purification by automated flash column chromatography (0-20% EtOAc in *n*-heptane, 25 g SiO_2). **3h** was isolated as a colourless oil (101.9 mg, 90%). **^{1}H NMR** (500 MHz, $CDCl_3$) δ (ppm): 8.81 (d, *J* = 4.8 Hz, 2H), 8.41 (td, *J* = 1.8, 0.7 Hz, 1H), 8.32 (dt, *J* = 7.1, 1.8 Hz, 1H), 7.48 – 7.41 (m, 2H), 7.18 (t, *J* = 4.8 Hz, 1H), 4.11 (q, *J* = 7.1 Hz, 2H), 2.93 – 2.86 (m, 2H), 2.64 – 2.56 (m, 2H), 2.13 – 2.02 (m, 1H), 1.95 – 1.86 (m, 1H), 1.17 (t, *J* = 7.1 Hz, 3H). **^{13}C NMR** (126 MHz, $CDCl_3$) δ (ppm): 175.9, 164.8, 157.3 (2C), 144.4, 137.7, 129.0, 128.6, 126.6, 126.1, 119.2, 61.0, 52.6, 32.5 (2C), 16.8, 14.2. **HRMS** (ESI): *m/z* calcd. for $C_{17}H_{18}N_2O_2$ $[M+H]^+$: 283.1447, found: 283.1440.

Ethyl 2,2-difluoro-2-(3-(pyrimidin-2-yl)phenyl)acetate (3i)

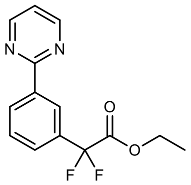

Prepared according to **General Procedure A**, using 2-phenylpyrimidine (62.5 mg, 0.40 mmol) as substrate and ethyl 2-bromo-2,2-difluoroacetate (154 µL, 1.20 mmol) as alkyl bromide. The crude reaction mixture was analysed by LC-MS using an acidic mobile phase at pH 3 and the UV chromatograms are shown below:

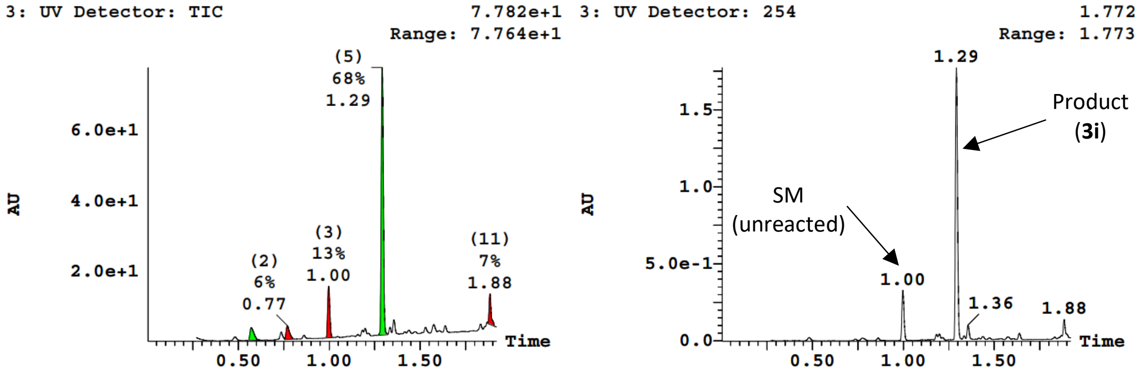

Supplementary Fig. 17. UV chromatograms (TIC & 254 nm) of the crude reaction mixture forming product **3i**.

Purification by automated flash column chromatography (0-20% EtOAc in *n*-heptane, 25 g SiO_2). **3i** was isolated as a white solid (87.0 mg, 78%). **^{1}H NMR** (500 MHz, $CDCl_3$) δ (ppm): 8.83 (d, *J* = 4.8 Hz, 2H), 8.74 (t, *J* = 2.1 Hz, 1H), 8.59 (ddt, *J* = 7.9, 1.9, 0.9 Hz, 1H), 7.73 (ddt, *J* = 7.8, 1.9, 0.9 Hz, 1H), 7.59 (td, *J* = 7.8, 0.8 Hz, 1H), 7.23 (t, *J* = 4.8 Hz, 1H), 4.32 (q, *J* = 7.1 Hz, 2H), 1.31 (t, *J* = 7.1 Hz, 3H). **^{13}C NMR** (126 MHz, $CDCl_3$) δ (ppm): 164.3 (t, $^{2}J_{C\text{-}F}$ = 35.2 Hz), 163.7, 157.5 (2C), 138.4, 133.5 (t, $^{2}J_{C\text{-}F}$ = 25.7 Hz), 130.7 (t, $^{4}J_{C\text{-}F}$ = 1.8 Hz), 129.1, 127.8 (t, $^{3}J_{C\text{-}F}$ = 6.0 Hz), 125.5 (t, $^{3}J_{C\text{-}F}$ = 6.3 Hz), 119.7, 113.5 (t, $^{1}J_{C\text{-}F}$ = 252.3 Hz), 63.3, 14.0. **^{19}F NMR** (471 MHz, $CDCl_3$) δ (ppm): -103.6 (s, 2F). **HRMS** (ESI): *m/z* calcd. for $C_{14}H_{12}F_2N_2O_2$ $[M+H]^+$: 279.0945, found: 279.0953. The analytical data are in accordance with previously reported literature values[7].

Diethyl (difluoro(3-(pyrimidin-2-yl)phenyl)methyl)phosphonate (3j)

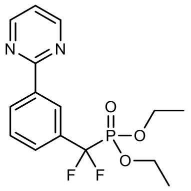

Prepared according to **General Procedure A**, using 2-phenylpyrimidine (62.5 mg, 0.40 mmol) as substrate and diethyl (bromodifluoromethyl)phosphonate (213 µL, 1.20 mmol) as alkyl bromide. The crude reaction mixture was analysed by LC-MS using an acidic mobile phase at pH 3 and the UV chromatograms are shown below:

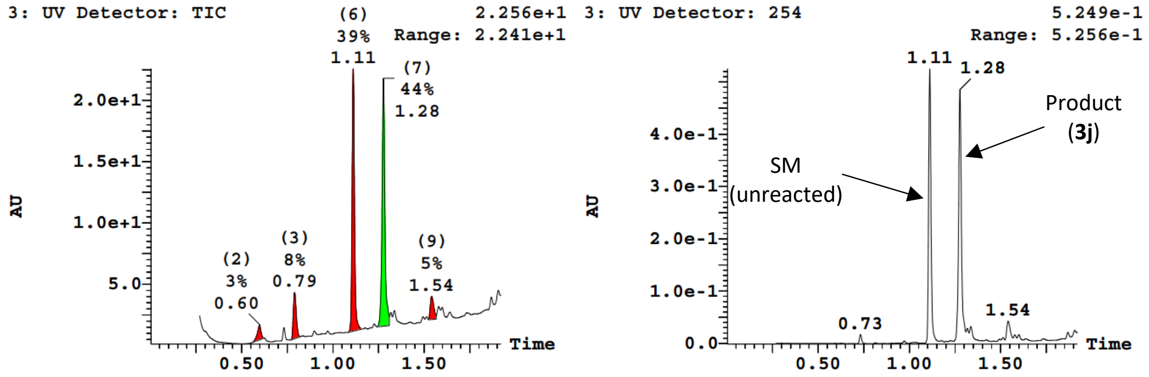

Supplementary Fig. 18. UV chromatograms (TIC & 254 nm) of the crude reaction mixture forming product **3j**.

Purification by automated flash column chromatography (0-50% EtOAc in *n*-heptane, 25 g SiO_2). **3j** was isolated as a colourless oil (60.5 mg, 44%). **^{1}H NMR** (500 MHz, $CDCl_3$) δ (ppm): 8.82 (d, *J* = 4.8 Hz, 2H), 8.74 (t, *J* = 2.0 Hz, 1H), 8.58 (ddt, *J* = 7.8, 2.1, 1.0 Hz, 1H), 7.75 (dd, *J* = 7.8, 1.8 Hz, 1H), 7.59 (t, *J* = 7.8 Hz, 1H), 7.22 (t, *J* = 4.8 Hz, 1H), 4.29 – 4.12 (m, 4H), 1.32 (td, *J* = 7.1, 0.7 Hz, 6H). **^{13}C NMR** (126 MHz, $CDCl_3$) δ (ppm): 163.8, 157.4 (2C), 138.1, 133.2 (td, $J_{C\text{-}F,C\text{-}P}$ = 22.1, 13.9 Hz), 130.5 (td, $J_{C\text{-}F,C\text{-}P}$ = 2.1, 1.9 Hz), 128.9, 128.6 (td, $J_{C\text{-}F,C\text{-}P}$ = 6.6, 2.2 Hz), 126.2 (td, $J_{C\text{-}F,C\text{-}P}$ = 7.1, 2.5 Hz), 119.6, 118.2 (td, $J_{C\text{-}F,C\text{-}P}$ = 263.4, 217.9 Hz), 65.0 (d, $J_{C\text{-}P}$ = 6.8 Hz, 2C), 16.4 (d, $J_{C\text{-}P}$ = 5.5 Hz, 2C). **^{19}F NMR** (471 MHz, $CDCl_3$) δ (ppm): -108.4 (d, $J_{F\text{-}P}$ = 115.6 Hz, 2F). **^{31}P NMR** (203 MHz, $CDCl_3$) δ (ppm): 6.25 (t, $J_{P\text{-}F}$ = 115.9 Hz, 1P). **HRMS** (ESI): *m/z* calcd. for $C_{15}H_{17}F_2N_2O_3P$ $[M+H]^+$: 343.1023, found: 343.1021.

Ethyl 2-(3-(1*H*-pyrazol-1-yl)phenyl)-2-methylpropanoate (3k)

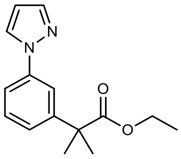

Prepared according to **General Procedure A**, using 1-phenyl-1*H*-pyrazole (52.9 µL, 0.40 mmol) as substrate and ethyl 2-bromo-2-methylpropanoate (176 µL, 1.20 mmol) as alkyl bromide. The crude reaction mixture was analysed by LC-MS using an acidic mobile phase at pH 3 and the UV chromatograms are shown below:

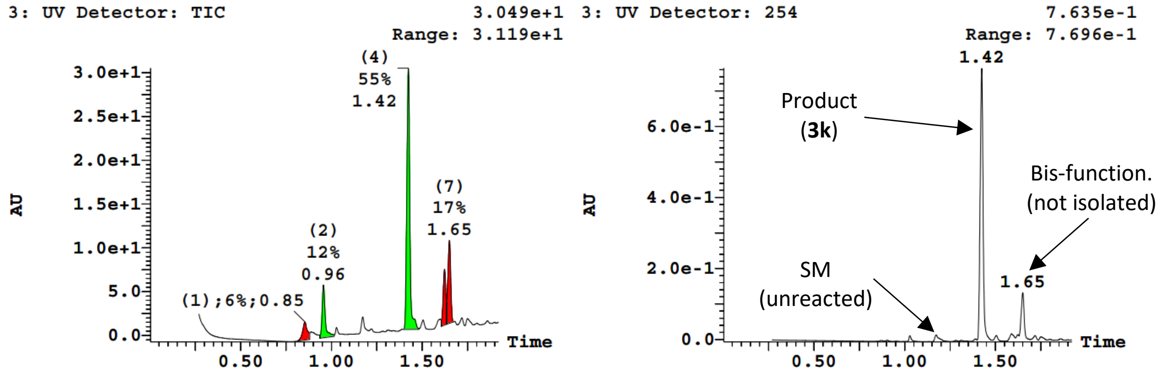

Supplementary Fig. 19. UV chromatograms (TIC & 254 nm) of the crude reaction mixture forming product **3k**.

Purification by automated flash column chromatography (0-15% EtOAc in *n*-heptane, 25 g SiO_2). **3k** was isolated as a colourless oil (79.8 mg, 77%). **^{1}H NMR** (500 MHz, $CDCl_3$) δ (ppm): 7.92 (dd, *J* = 2.4, 0.6 Hz, 1H), 7.74 – 7.70 (m, 2H), 7.53 (ddd, *J* = 8.0, 2.2, 1.0 Hz, 1H), 7.39 (t, *J* = 7.9 Hz, 1H), 7.26 (ddd, *J* = 7.8, 1.9, 1.0 Hz, 1H), 6.46 (dd, *J* = 2.5, 1.8 Hz, 1H), 4.13 (q, *J* = 7.1 Hz, 2H), 1.62 (s, 6H), 1.19 (t, *J* = 7.1 Hz, 3H). **^{13}C NMR** (126 MHz, $CDCl_3$) δ (ppm): 176.4, 146.6, 141.1, 140.4, 129.4, 126.9, 124.1, 117.6, 117.0, 107.6, 61.0, 46.7, 26.6 (2C), 14.1. **HRMS** (ESI): *m/z* calcd. for $C_{15}H_{18}N_2O_2$ $[M+H]^+$: 259.1447, found: 259.1447.

Ethyl 2-(3-(1*H*-pyrazol-1-yl)phenyl)-2-methylpropanoate (3l)

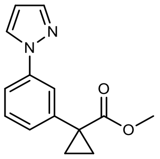

Prepared according to **General Procedure A**, using 1-phenyl-1*H*-pyrazole (52.9 µL, 0.40 mmol) as substrate, methyl 1-bromocyclopropane-1-carboxylate (124 µL, 1.20 mmol) as alkyl bromide and *tert*-amyl alcohol (*t*-AmOH, 4.0 mL, 0.1 M) as solvent. The crude reaction mixture was analysed by LC-MS using an acidic mobile phase at pH 3 and the UV chromatograms are shown below:

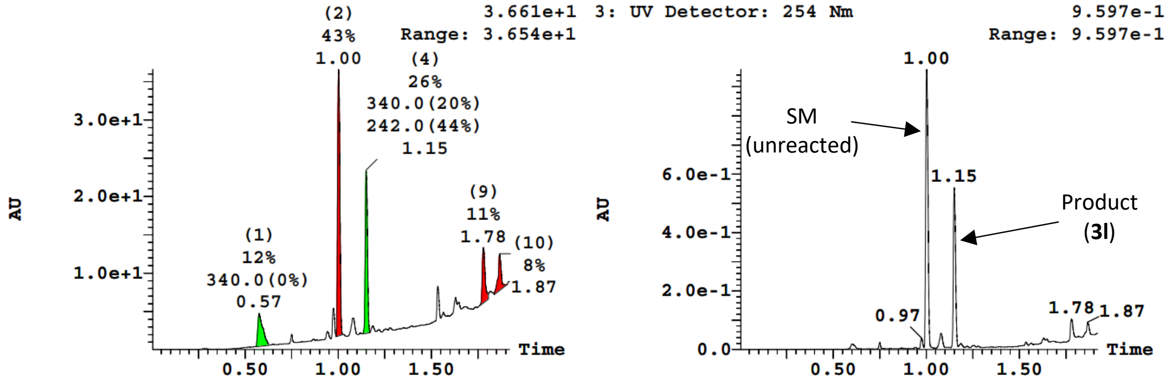

Supplementary Fig. 20. UV chromatograms (TIC & 254 nm) of the crude reaction mixture forming product **3l**.

Purification by automated flash column chromatography (0-15% EtOAc in *n*-heptane, 25 g SiO_2). **3l** was isolated as a colourless oil (35.3 mg, 36%). **^{1}H NMR** (500 MHz, $CDCl_3$) δ (ppm): 7.93 (dd, *J* = 2.5, 0.7 Hz, 1H), 7.73 (t, *J* = 2.0 Hz, 1H), 7.72 (dd, *J* = 1.8, 0.6 Hz, 1H), 7.56 (ddd, *J* = 8.1, 2.3, 1.1 Hz, 1H), 7.39 (t, *J* = 7.9 Hz, 1H), 7.27 (ddd, *J* = 7.7, 1.7, 1.1 Hz, 1H), 6.46 (dd, *J* = 2.5, 1.8 Hz, 1H), 3.63 (s, 3H), 1.66 – 1.63 (m, 2H), 1.27 – 1.24 (m, 2H). **^{13}C NMR** (126 MHz, $CDCl_3$) δ (ppm): 174.8, 141.3, 141.2, 140.1, 129.3, 128.8, 126.9, 121.7, 118.0, 107.7, 52.6, 29.1, 16.9 (2C). **HRMS** (ESI): *m/z* calcd. for $C_{14}H_{14}N_2O_2$ $[M+H]^+$: 243.1133, found: 243.1132.

Ethyl 1-(3-(1*H*-pyrazol-1-yl)phenyl)cyclobutane-1-carboxylate (3m)

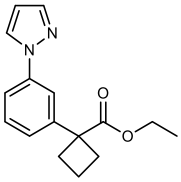

Prepared according to **General Procedure A**, using 1-phenyl-1*H*-pyrazole (52.9 µL, 0.40 mmol) as substrate and ethyl 1-bromocyclobutane-1-carboxylate (194 µL, 1.20 mmol) as alkyl bromide. The crude reaction mixture was analysed by LC-MS using an acidic mobile phase at pH 3 and the UV chromatograms are shown below:

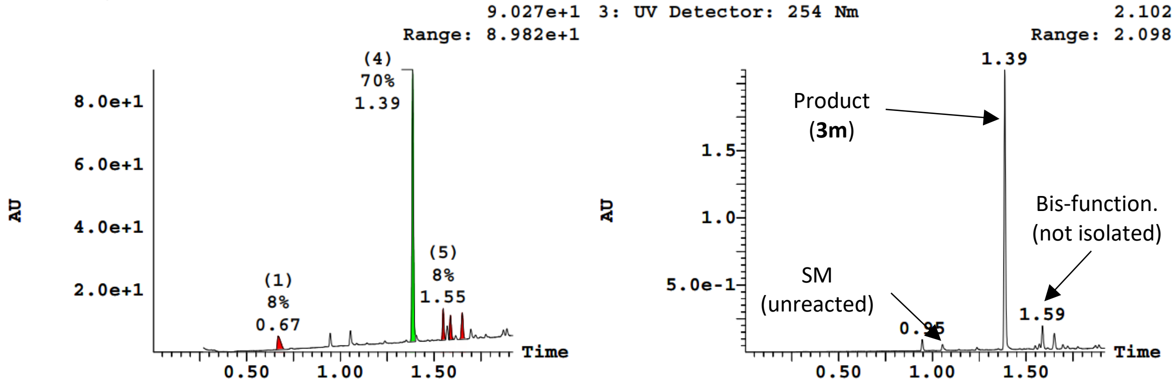

Supplementary Fig. 21. UV chromatograms (TIC & 254 nm) of the crude reaction mixture forming product **3m**.

Purification by automated flash column chromatography (0-15% EtOAc in *n*-heptane, 25 g SiO_2). **3m** was isolated as a colourless oil (88.8 mg, 82%). **^{1}H NMR** (500 MHz, $CDCl_3$) δ (ppm): 7.93 (d, *J* = 2.5 Hz, 1H), 7.72 (d, *J* = 1.8 Hz, 1H), 7.65 (t, *J* = 2.0 Hz, 1H), 7.54 (ddd, *J* = 8.0, 2.2, 1.0 Hz, 1H), 7.40 (t, *J* = 7.9 Hz, 1H), 7.22 (ddd, *J* = 7.7, 1.7, 1.0 Hz, 1H), 6.46 (t, *J* = 2.1 Hz, 1H), 4.11 (q, *J* = 7.1 Hz, 2H), 2.90 – 2.83 (m, 2H), 2.59 – 2.51 (m, 2H), 2.13 – 2.10 (m, 1H), 1.95 – 1.83 (m, 1H), 1.18 (t, *J* = 7.1 Hz, 3H). **^{13}C NMR** (126 MHz, $CDCl_3$) δ (ppm): 175.5, 145.6, 141.1, 140.2, 129.3, 126.9, 124.5, 117.5, 117.4, 107.6, 61.1, 52.5, 32.4 (2C), 16.7, 14.1. **HRMS** (ESI): *m/z* calcd. for $C_{16}H_{18}N_2O_2$ $[M+H]^+$: 271.1447, found: 271.1459.

Ethyl 2-(3-(1*H*-pyrazol-1-yl)phenyl)-2,2-difluoroacetate (3n)

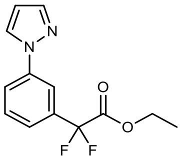

Prepared according to **General Procedure A**, using 1-phenyl-1*H*-pyrazole (52.9 µL, 0.40 mmol) as substrate and ethyl 2-bromo-2,2-difluoroacetate (154 µL, 1.20 mmol) as alkyl bromide. The crude reaction mixture was analysed by LC-MS using an acidic mobile phase at pH 3 and the UV chromatograms are shown below:

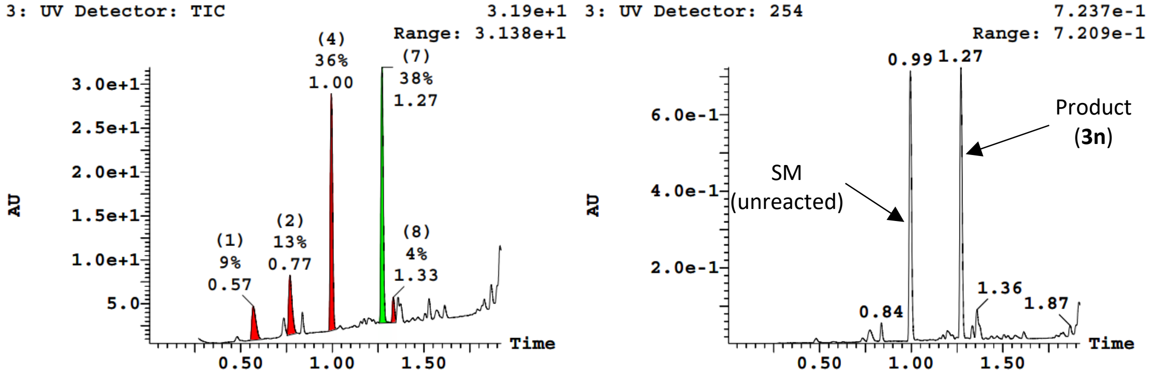

Supplementary Fig. 22. UV chromatograms (TIC & 254 nm) of the crude reaction mixture forming product **3n**.

Purification by automated flash column chromatography (0-15% EtOAc in *n*-heptane, 25 g SiO_2). **3n** was isolated as a colourless oil (54.5 mg, 51%). **^{1}H NMR** (500 MHz, $CDCl_3$) δ (ppm): 7.97 (d, *J* = 2.5 Hz, 1H), 7.95 (t, *J* = 2.1 Hz, 1H), 7.87 (dt, *J* = 7.6, 1.8 Hz, 1H), 7.75 (d, *J* = 1.8 Hz, 1H), 7.55 (t, *J* = 7.8 Hz, 1H), 7.52 (dt, *J* = 7.8, 1.6 Hz, 1H), 6.50 (t, *J* = 2.0 Hz, 1H), 4.32 (q, *J* = 7.1 Hz, 2H), 1.31 (t, *J* = 7.1 Hz, 3H). **^{13}C NMR** (126 MHz, $CDCl_3$) δ (ppm): 164.0 (t, $^2J_{C\text{-}F}$ = 35.0 Hz), 141.7, 140.6, 134.5 (t, $^2J_{C\text{-}F}$ = 25.9 Hz), 130.1, 126.9, 123.4 (t, $^3J_{C\text{-}F}$ = 6.1 Hz), 121.6 (t, $^4J_{C\text{-}F}$ = 1.8 Hz), 116.2 (t, $^3J_{C\text{-}F}$ = 6.5 Hz), 113.1 (t, $^1J_{C\text{-}F}$ = 252.9 Hz), 108.3, 63.5, 14.0. **^{19}F NMR** (471 MHz, $CDCl_3$) δ (ppm): -104.0 (s, 2F). **HRMS** (ESI): *m/z* calcd. for $C_{13}H_{12}F_2N_2O_2$ $[M+H]^+$: 267.0945, found: 267.0950. The analytical data are in accordance with previously reported literature values[10].

Ethyl 2-(3-(3,5-dimethyl-1*H*-pyrazol-1-yl)phenyl)-2-methylpropanoate (3o)

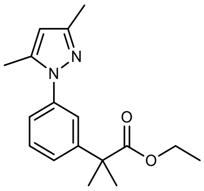

Prepared according to **General Procedure A**, using 3,5-dimethyl-1-phenyl-1*H*-pyrazole (65.0 µL, 0.40 mmol) as substrate and ethyl 2-bromo-2-methylpropanoate (176 µL, 1.20 mmol) as alkyl bromide. The crude reaction mixture was analysed by LC-MS using an acidic mobile phase at pH 3 and the UV chromatograms are shown below:

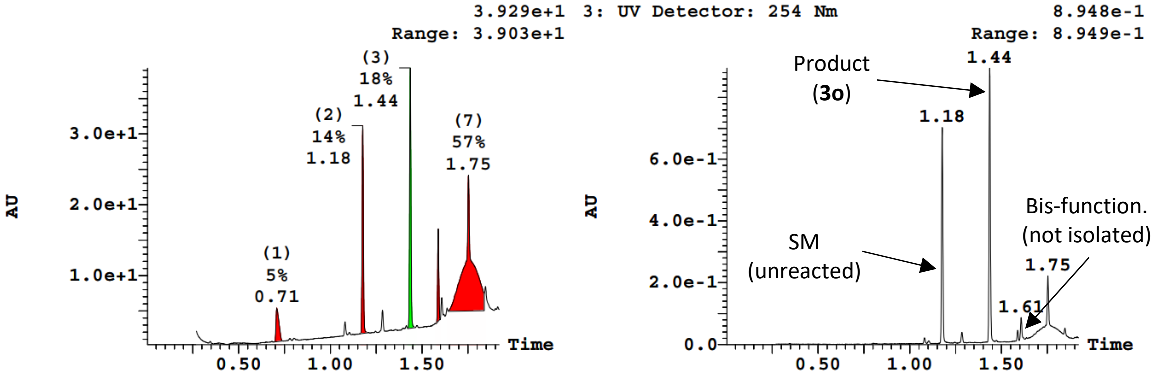

Supplementary Fig. 23. UV chromatograms (TIC & 254 nm) of the crude reaction mixture forming product **3o**.

Purification by automated flash column chromatography (0-20% EtOAc in *n*-heptane, 25 g SiO_2). **3o** was isolated as a colourless oil (60.2 mg, 52%). **^{1}H NMR** (500 MHz, $CDCl_3$) δ (ppm): 7.41 – 7.36 (m, 2H), 7.33 – 7.28 (m, 2H), 5.99 (s, 1H), 4.11 (q, *J* = 7.1 Hz, 2H), 2.29 (s, 3H), 2.28 (s, 3H), 1.59 (s, 6H), 1.18 (*t*, J = 7.1 Hz, 3H). **^{13}C NMR** (126 MHz, $CDCl_3$) δ (ppm): 176.4, 149.0, 145.9, 140.0, 139.5, 129.0, 124.8, 123.2, 122.4, 107.0, 61.0, 46.6, 26.5 (2C), 14.1, 13.6, 12.4. **HRMS** (ESI): *m/z* calcd. for $C_{17}H_{22}N_2O_2$ $[M+H]^+$: 287.1759, found: 287.1766.

Ethyl 2-(3-(3,5-dimethyl-1*H*-pyrazol-1-yl)phenyl)-2,2-difluoroacetate (3p)

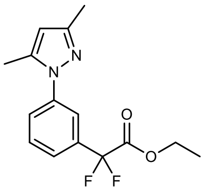

Prepared according to **General Procedure A**, using 3,5-dimethyl-1-phenyl-1*H*-pyrazole (65.0 µL, 0.40 mmol) as substrate and ethyl 2-bromo-2,2-difluoroacetate (154 µL, 1.20 mmol) as alkyl bromide. The crude reaction mixture was analysed by LC-MS using an acidic mobile phase at pH 3 and the UV chromatograms are shown below:

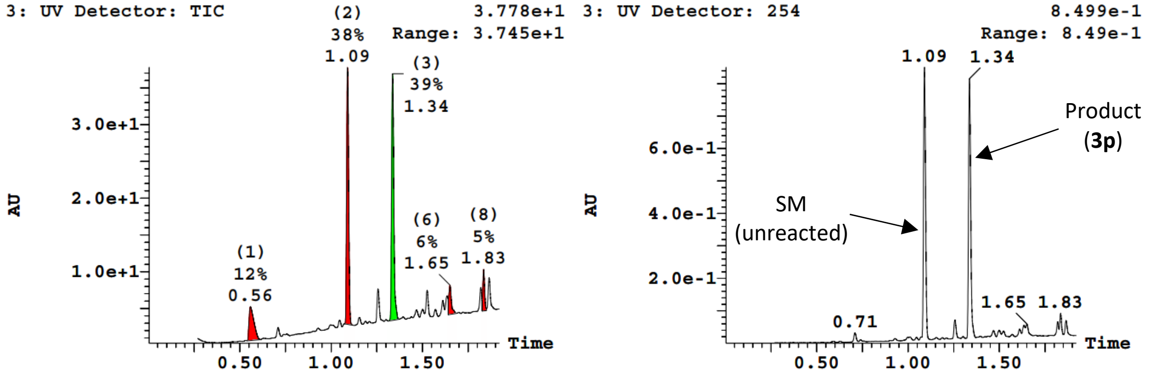

Supplementary Fig. 24. UV chromatograms (TIC & 254 nm) of the crude reaction mixture forming product **3p**.

Purification by automated flash column chromatography (0-20% EtOAc in *n*-heptane, 25 g SiO_2). **3p** was isolated as a colourless oil (56.5 mg, 48%). **^{1}H NMR** (500 MHz, $CDCl_3$) δ (ppm): 7.69 (t, *J* = 2.0 Hz, 1H), 7.61 (dt, *J* = 7.7, 1.8 Hz, 1H), 7.58 (dt, *J* = 7.9, 1.7 Hz, 1H), 7.54 (t, *J* = 7.7 Hz, 1H), 6.02 (s, 1H), 4.30 (q, *J* = 7.1 Hz, 2H), 2.33 (s, 3H), 2.29 (s, 3H), 1.31 (t, *J* = 7.1 Hz, 3H). **^{13}C NMR** (126 MHz, $CDCl_3$) δ (ppm): 164.0 (t, $^2J_{C\text{-}F}$ = 35.0 Hz), 149.7, 140.4, 139.6, 134.0 (t, $^2J_{C\text{-}F}$ = 25.9 Hz), 129.7, 127.2 (t, $^4J_{C\text{-}F}$ = 1.8 Hz), 124.2 (t, $^3J_{C\text{-}F}$ = 6.1 Hz), 121.7 (t, $^3J_{C\text{-}F}$ = 6.3 Hz), 113.0 (t, $^1J_{C\text{-}F}$ = 252.8 Hz), 107.8, 63.5, 14.0, 13.6, 12.6. **^{19}F NMR** (471 MHz, $CDCl_3$) δ (ppm): -103.8 (s, 2F). **HRMS** (ESI): *m/z* calcd. for $C_{15}H_{16}F_2N_2O_2$ $[M+H]^+$: 295.1258, found: 295.1250.

Ethyl 2-(3-(4,5-dihydrooxazol-2-yl)phenyl)-2-methylpropanoate (3q)

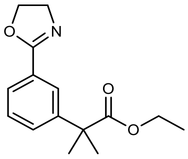

Prepared according to **General Procedure A**, using 2-phenyl-2-oxazoline (52.7 µL, 0.40 mmol) as substrate and ethyl 2-bromo-2-methylpropanoate (176 µL, 1.20 mmol) as alkyl bromide. The crude reaction mixture was analysed by LC-MS using an acidic mobile phase at pH 3 and the UV chromatograms are shown below:

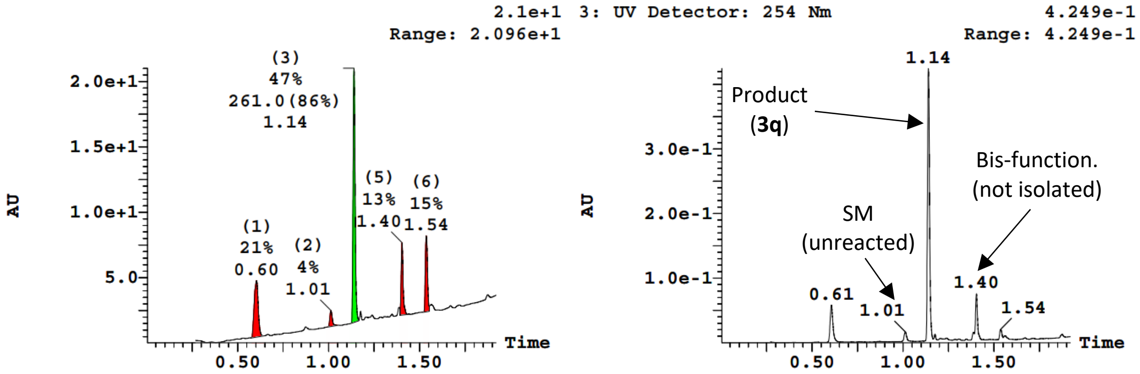

Supplementary Fig. 25. UV chromatograms (TIC & 254 nm) of the crude reaction mixture forming product **3q**.

Purification by automated flash column chromatography (0-40% EtOAc in *n*-heptane, 25 g SiO_2). **3q** was isolated as a colourless oil (63.4 mg, 60%). **^{1}H NMR** (500 MHz, $CDCl_3$) δ (ppm): 7.95 (t, *J* = 1.8 Hz, 1H), 7.81 (dt, *J* = 7.6, 1.4 Hz, 1H), 7.44 (ddd, *J* = 7.9, 2.1, 1.2 Hz, 1H), 7.36 (t, *J* = 7.8 Hz, 1H), 4.43 (t, *J* = 9.6 Hz, 2H), 4.11 (q, *J* = 7.1 Hz, 2H), 4.06 (t, *J* = 9.5 Hz, 2H), 1.59 (s, 6H), 1.17 (t, *J* = 7.1 Hz, 3H). **^{13}C NMR** (126 MHz, $CDCl_3$) δ (ppm): 176.5, 164.8, 145.2, 129.1, 128.5, 127.9, 126.6, 125.5, 67.7, 61.0, 55.1, 46.6, 26.6 (2C), 14.1. **HRMS** (ESI): *m/z* calcd. for $C_{15}H_{19}NO_3$ $[M+H]^+$: 262.1443, found: 262.1448.

Methyl 1-(3-(4,5-dihydrooxazol-2-yl)phenyl)cyclopropane-1-carboxylate (3r)

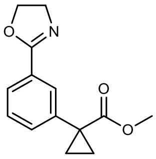

Prepared according to **General Procedure A**, using 2-phenyl-2-oxazoline (52.7 µL, 0.40 mmol) as substrate, methyl 1-bromocyclopropane-1-carboxylate (124 µL, 1.20 mmol) as alkyl bromide and *tert*-amyl alcohol (*t*-AmOH, 4.0 mL, 0.1 M) as solvent. The crude reaction mixture was analysed by LC-MS using an acidic mobile phase at pH 3 and the UV chromatograms are shown below:

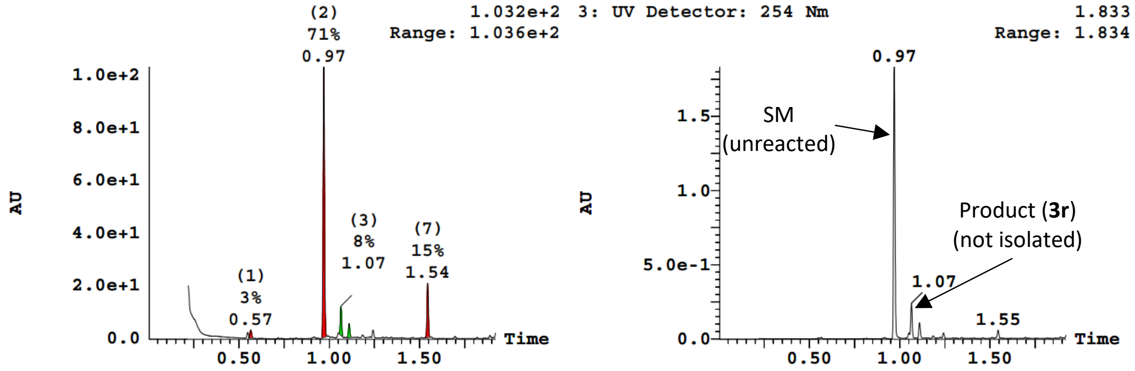

Supplementary Fig. 26. UV chromatograms (TIC & 254 nm) of the crude reaction mixture forming product **3r**.

LC-MS analysis of the crude reaction mixture showed around 7% conversion into the desired alkylated product, **3r** was not isolated.

Ethyl 1-(3-(4,5-dihydrooxazol-2-yl)phenyl)cyclobutane-1-carboxylate (3s)

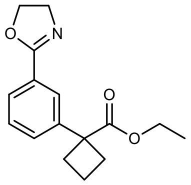

Prepared according to **General Procedure A**, using 2-phenyl-2-oxazoline (52.7 µL, 0.40 mmol) as substrate and ethyl 1-bromocyclobutane-1-carboxylate (194 µL, 1.20 mmol) as alkyl bromide. The crude reaction mixture was analysed by LC-MS using an acidic mobile phase at pH 3 and the UV chromatograms are shown below:

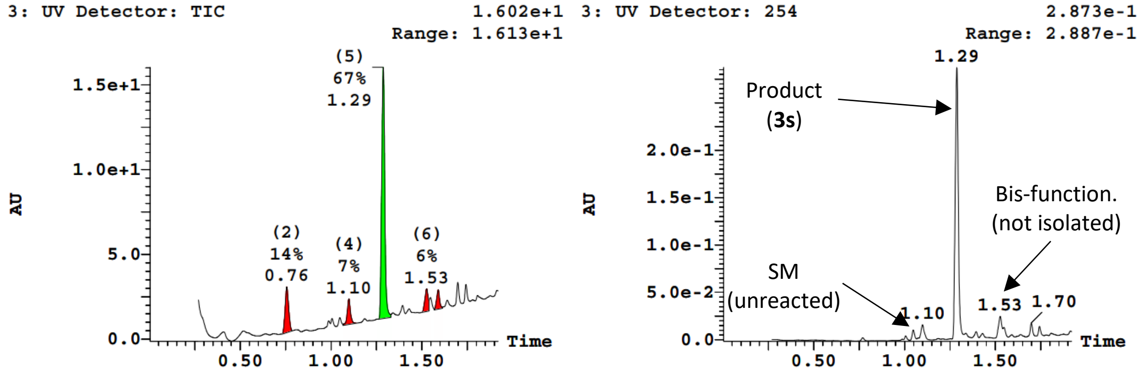

Supplementary Fig. 27. UV chromatograms (TIC & 254 nm) of the crude reaction mixture forming product **3s**.

Purification by automated flash column chromatography (0-40% EtOAc in *n*-heptane, 25 g SiO_2). **3s** was isolated as a colourless oil (86.0 mg, 79%). **^{1}H NMR** (500 MHz, $CDCl_3$) δ (ppm): 7.89 (t, *J* = 1.8 Hz, 1H), 7.80 (dt, *J* = 7.5, 1.5 Hz, 1H), 7.40 (dt, *J* = 7.8, 1.6 Hz, 1H), 7.36 (t, *J* = 7.7 Hz, 1H), 4.43 (t, *J* = 9.6 Hz, 2H), 4.08 (q, *J* = 7.1 Hz, 2H), 4.06 (t, *J* = 9.4 Hz, 2H), 2.87 – 2.80 (m, 2H), 2.55 – 2.47 (m, 2H), 2.10 – 2.00 (m, 1H), 1.91 – 1.82 (m, 1H), 1.15 (t, *J* = 7.1 Hz, 3H). **^{13}C NMR** (126 MHz, $CDCl_3$) δ (ppm): 175.6, 164.7, 144.2, 129.5, 128.4, 127.8, 126.5, 126.1, 67.7, 61.0, 55.0, 52.4, 32.4 (2C), 16.8, 14.1. **HRMS** (ESI): *m/z* calcd. for $C_{16}H_{19}NO_3$ $[M+H]^+$: 274.1443, found: 274.1439.

Ethyl 2-(3-(4,5-dihydrooxazol-2-yl)phenyl)-2,2-difluoroacetate (3t)

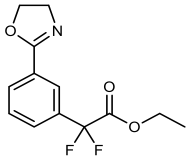

Prepared according to **General Procedure A**, using 2-phenyl-2-oxazoline (52.7 µL, 0.40 mmol) as substrate and ethyl 2-bromo-2,2-difluoroacetate (154 µL, 1.20 mmol) as alkyl bromide. The crude reaction mixture was analysed by LC-MS using an acidic mobile phase at pH 3 and the UV chromatograms are shown below:

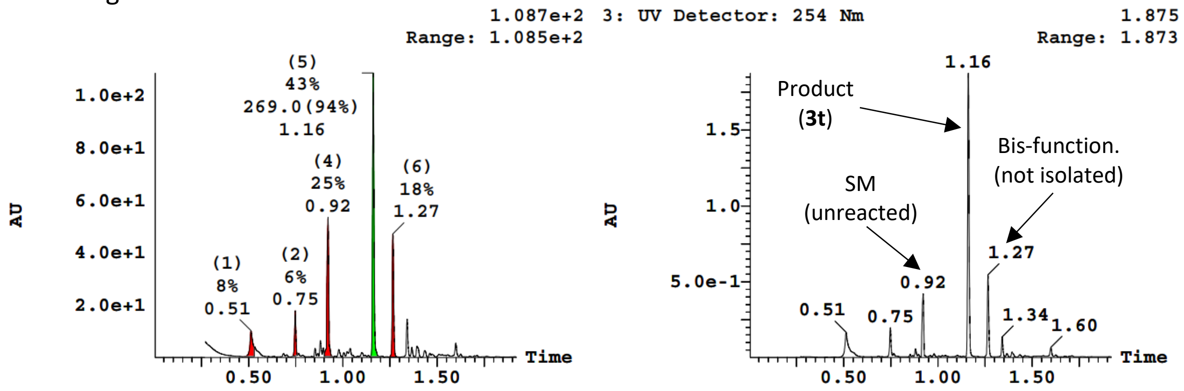

Supplementary Fig. 28. UV chromatograms (TIC & 254 nm) of the crude reaction mixture forming product **3t**.

Purification by automated flash column chromatography (0-40% EtOAc in *n*-heptane, 25 g SiO_2). **3t** was isolated as a colourless oil (51.1 mg, 47%). **^{1}H NMR** (500 MHz, $CDCl_3$) δ (ppm): 8.19 (t, *J* = 1.9 Hz, 1H), 8.08 (dt, *J* = 7.8, 1.4 Hz, 1H), 7.72 (dt, *J* = 7.8, 1.5 Hz, 1H), 7.51 (t, *J* = 7.8 Hz, 1H), 4.46 (t, *J* = 9.7 Hz, 2H), 4.30 (q, *J* = 7.1 Hz, 2H), 4.08 (t, *J* = 9.6 Hz, 2H), 1.30 (t, *J* = 7.1 Hz, 3H). **^{13}C NMR** (126 MHz, $CDCl_3$) δ (ppm): 164.0 (t, $^{2}J_{C-F}$ = 35.0 Hz), 163.8, 133.3 (t, $^{2}J_{C-F}$ = 25.9 Hz), 130.8 (t, $^{4}J_{C-F}$ = 1.8 Hz), 128.9, 128.6, 128.3 (t, $^{3}J_{C-F}$ = 6.0 Hz), 125.5 (t, $^{3}J_{C-F}$ = 6.3 Hz), 113.1 (t, $^{1}J_{C-F}$ = 252.6 Hz), 68.0, 63.4, 55.1, 14.0. **^{19}F NMR** (471 MHz, $CDCl_3$) δ (ppm): -103.9 (s, 2F). **HRMS** (ESI): *m/z* calcd. for $C_{13}H_{13}F_2NO_3$ $[M+H]^+$: 270.0942, found: 270.0945.

Ethyl 1-(benzo[*h*]quinolin-7-yl)cyclobutane-1-carboxylate (3u)

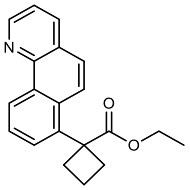

Prepared according to **General Procedure A**, using benzo[*h*]quinoline (71.7 mg, 0.40 mmol) as substrate and ethyl 1-bromocyclobutane-1-carboxylate (194 µL, 1.20 mmol) as alkyl bromide. The crude reaction mixture was analysed by LC-MS using an acidic mobile phase at pH 3 and the UV chromatograms are shown below:

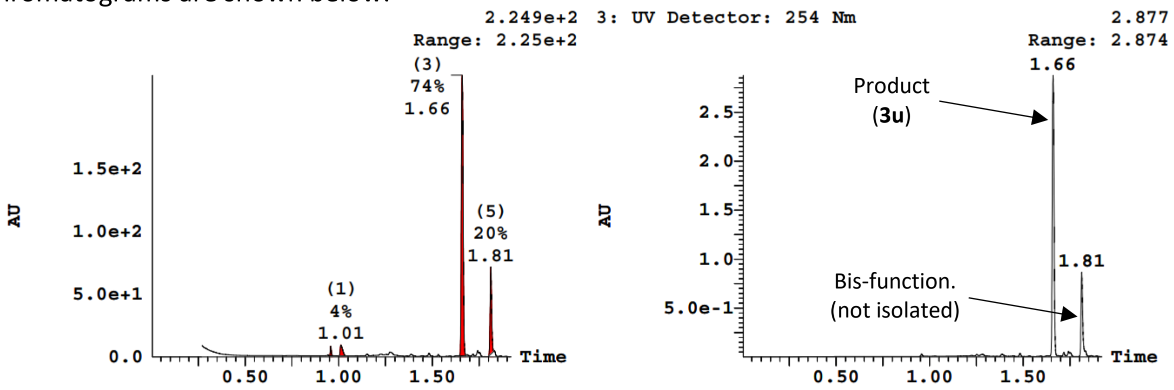

Supplementary Fig. 29. UV chromatograms (TIC & 254 nm) of the crude reaction mixture forming product **3u**.

Purification by automated flash column chromatography (0-20% EtOAc in *n*-heptane, 25 g SiO_2). **3u** was isolated as a white solid (95.1 mg, 78%). **^{1}H NMR** (500 MHz, $CDCl_3$) δ (ppm): 9.30 (ddd, *J* = 8.0, 1.5, 0.8 Hz, 1H), 8.99 (dd, *J* = 4.4, 1.8 Hz, 1H), 8.16 (dd, *J* = 8.0, 1.8 Hz, 1H), 7.85 (dd, *J* = 9.2, 0.8 Hz, 1H), 7.73 (dd, *J* = 8.1, 7.3 Hz, 1H), 7.69 (dd, *J* = 7.4, 1.5 Hz, 1H), 7.66 (d, *J* = 9.2 Hz, 1H), 7.51 (dd, *J* = 8.0, 4.3 Hz, 1H), 4.08 (q, *J* = 7.1 Hz, 2H), 3.14 – 3.04 (m, 2H), 2.82 – 2.70 (m, 2H), 2.28 (dp, *J* = 11.1, 8.9 Hz, 1H), 2.00 – 1.90 (m, 1H), 1.06 (t, *J* = 7.1 Hz, 3H). **^{13}C NMR** (126 MHz, $CDCl_3$) δ (ppm): 176.2, 148.8, 146.9, 140.2, 135.7, 132.3, 131.3, 127.1, 126.5, 125.8, 125.2, 124.6, 123.7, 121.9, 61.2, 53.1, 33.4 (2C), 17.2, 14.1. **HRMS** (ESI): *m/z* calcd. for $C_{20}H_{19}NO_2$ $[M+H]^+$: 306.1494, found: 306.1501.

Ethyl 1-(3-(pyridazin-3-yl)phenyl)cyclobutane-1-carboxylate (3v)

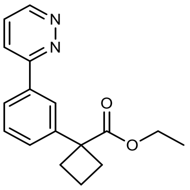

Prepared according to **General Procedure A**, using 3-phenylpyrazine (62.5 mg, 0.40 mmol) as substrate and ethyl 1-bromocyclobutane-1-carboxylate (194 µL, 1.20 mmol) as alkyl bromide. The crude reaction mixture was analysed by LC-MS using an acidic mobile phase at pH 3 and the UV chromatograms are shown below:

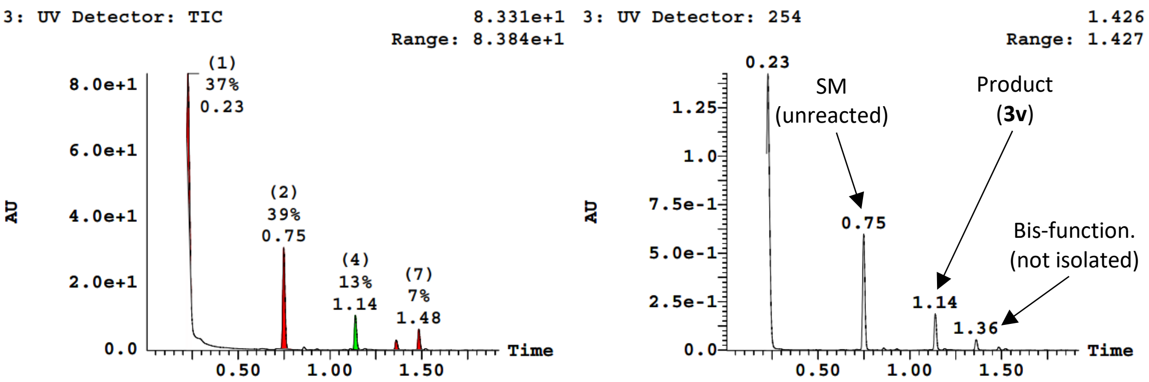

Supplementary Fig. 30. UV chromatograms (TIC & 254 nm) of the crude reaction mixture forming product **3v**.

Purification by automated flash column chromatography (0-50% EtOAc in *n*-heptane, 25 g SiO_2). **3v** was isolated as a pale yellow oil (22.7 mg, 20%). ^{1}**H NMR** (500 MHz, $CDCl_3$) δ (ppm): 9.16 (dd, *J* = 4.9, 1.6 Hz, 1H), 8.04 (t, *J* = 1.8 Hz, 1H), 7.93 (dt, *J* = 7.6, 1.5 Hz, 1H), 7.86 (dd, *J* = 8.6, 1.6 Hz, 1H), 7.54 (dd, *J* = 8.6, 4.9 Hz, 1H), 7.49 (t, *J* = 7.6 Hz, 1H), 7.45 (dt, *J* = 7.8, 1.5 Hz, 1H), 4.11 (q, *J* = 7.1 Hz, 2H), 2.93 – 2.86 (m, 2H), 2.63 – 2.55 (m, 2H), 2.14 – 2.03 (m, 1H), 1.96 – 1.86 (m, 1H), 1.18 (t, *J* = 7.1 Hz, 3H). ^{13}**C NMR** (126 MHz, $CDCl_3$) δ (ppm): 175.8, 159.6, 150.2, 145.0, 136.5, 129.1, 128.3, 126.9, 125.5, 125.3, 124.2, 61.2, 52.7, 32.6 (2C), 16.9, 14.2. **HRMS** (ESI): *m/z* calcd. for $C_{17}H_{18}N_2O_2$ $[M+H]^+$: 283.1447, found: 283.1447.

Ethyl 1-(3-(pyrazin-2-yl)phenyl)cyclobutane-1-carboxylate (3w)

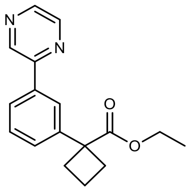

Prepared according to **General Procedure A**, using 2-phenylpyrazine (62.5 mg, 0.40 mmol) as substrate and ethyl 1-bromocyclobutane-1-carboxylate (194 µL, 1.20 mmol) as alkyl bromide. The crude reaction mixture was analysed by LC-MS using an acidic mobile phase at pH 3 and the UV chromatograms are shown below:

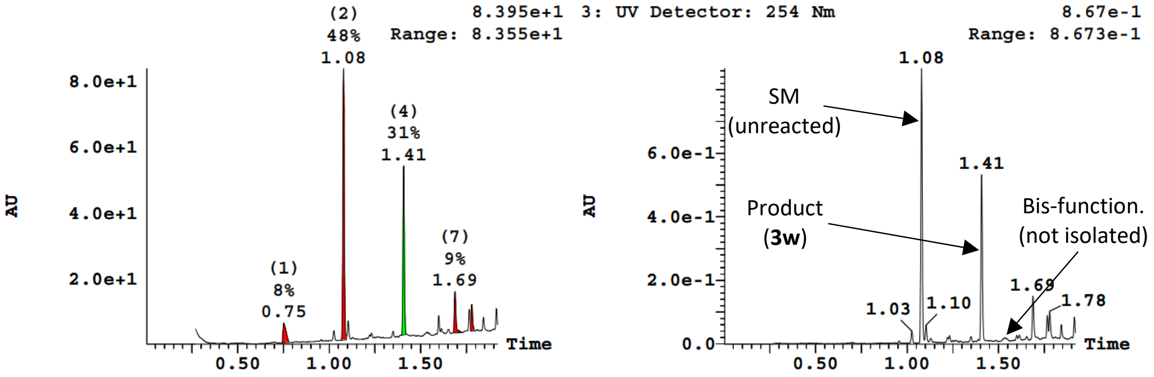

Supplementary Fig. 31. UV chromatograms (TIC & 254 nm) of the crude reaction mixture forming product **3w**.

Purification by automated flash column chromatography (0-20% EtOAc in *n*-heptane, 25 g SiO_2). **3w** was isolated as a colourless oil (52.5 mg, 46%). ^{1}**H NMR** (500 MHz, $CDCl_3$) δ (ppm): 9.02 (d, *J* = 1.6 Hz, 1H), 8.64 (dd, *J* = 2.5, 1.6 Hz, 1H), 8.51 (d, *J* = 2.5 Hz, 1H), 7.96 (t, *J* = 1.8 Hz, 1H), 7.87 (dt, *J* = 7.6, 1.5 Hz, 1H), 7.48 (t, *J* = 7.7 Hz, 1H), 7.42 (ddd, *J* = 7.7, 1.9, 1.2 Hz, 1H), 4.12 (q, *J* = 7.1 Hz, 2H), 2.95 – 2.85 (m, 2H), 2.63 – 2.53 (m, 2H), 2.15 – 2.04 (m, 1H), 1.96 – 1.86 (m, 1H), 1.18 (t, *J* = 7.1 Hz, 3H). ^{13}**C NMR** (126 MHz, $CDCl_3$) δ (ppm): 175.8, 153.0, 145.0, 144.3, 143.1, 142.5, 136.5, 129.1, 128.1, 125.3, 125.0, 61.2, 52.6, 32.5 (2C), 16.8, 14.2. **HRMS** (ESI): *m/z* calcd. for $C_{17}H_{18}N_2O_2$ $[M+H]^+$: 283.1447, found: 283.1440.

Ethyl 1-(3-(pyrimidin-4-yl)phenyl)cyclobutane-1-carboxylate (3x)

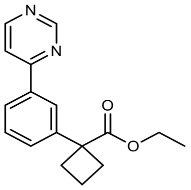

Prepared according to **General Procedure A**, using 4-phenylpyrimidine (62.5 mg, 0.40 mmol) as substrate and ethyl 1-bromocyclobutane-1-carboxylate (194 µL, 1.20 mmol) as alkyl bromide. The crude reaction mixture was analysed by LC-MS using an acidic mobile phase at pH 3 and the UV chromatograms are shown below:

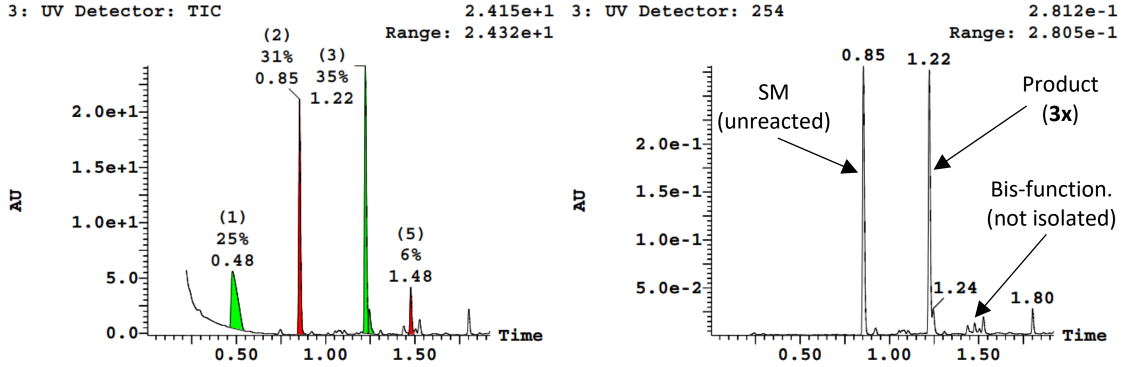

Supplementary Fig. 32. UV chromatograms (TIC & 254 nm) of the crude reaction mixture forming product **3x**.

Purification by automated flash column chromatography (0-25% EtOAc in *n*-heptane, 25 g SiO_2). **3x** was isolated as a colourless oil (59.0 mg, 52%). **^{1}H NMR** (500 MHz, $CDCl_3$) δ (ppm): 9.27 (d, *J* = 1.5 Hz, 1H), 8.76 (d, *J* = 5.4 Hz, 1H), 8.03 (dt, *J* = 2.4, 1.0 Hz, 1H), 7.95 (dt, *J* = 6.5, 2.3 Hz, 1H), 7.72 (dd, *J* = 5.3, 1.5 Hz, 1H), 7.50 – 7.44 (m, 2H), 4.11 (q, *J* = 7.1 Hz, 2H), 2.94 – 2.86 (m, 2H), 2.62 – 2.53 (m, 2H), 2.14 – 2.04 (m, 1H), 1.96 – 1.86 (m, 1H), 1.18 (t, *J* = 7.1 Hz, 3H). **^{13}C NMR** (126 MHz, $CDCl_3$) δ (ppm): 175.7, 164.0, 159.2, 157.6, 145.0, 136.6, 129.3, 129.1, 125.5, 125.1, 117.3, 61.2, 52.6, 32.5 (2C), 16.8, 14.2. **HRMS** (ESI): *m/z* calcd. for $C_{17}H_{18}N_2O_2$ $[M+H]^+$: 283.1447, found: 283.1451.

Ethyl 1-(3-(1,3,5-triazin-2-yl)phenyl)cyclobutane-1-carboxylate (3y)

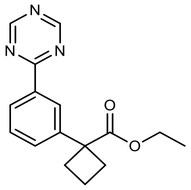

Prepared according to **General Procedure A**, using 2-phenyl-1,3,5-triazine (62.9 mg, 0.40 mmol) as substrate and ethyl 1-bromocyclobutane-1-carboxylate (194 µL, 1.20 mmol) as alkyl bromide. The crude reaction mixture was analysed by LC-MS using an acidic mobile phase at pH 3 and the UV chromatograms are shown below:

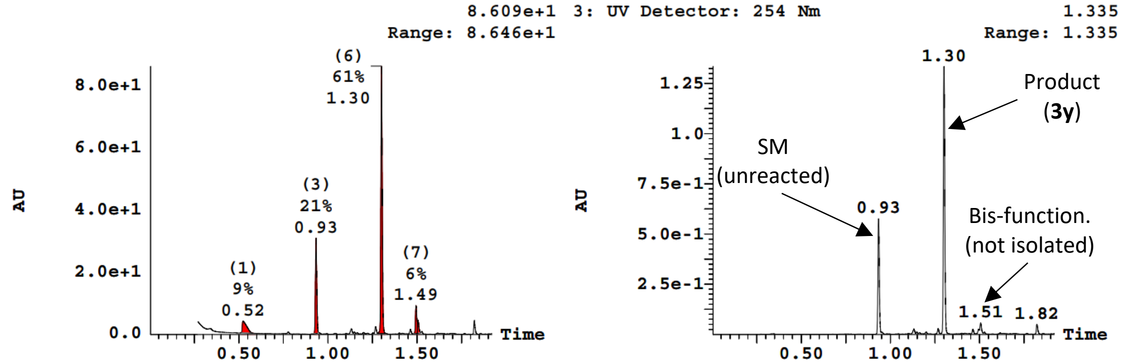

Supplementary Fig. 33. UV chromatograms (TIC & 254 nm) of the crude reaction mixture forming product **3y**.

Purification by automated flash column chromatography (0-20% EtOAc in *n*-heptane, 25 g SiO_2). **3y** was isolated as a colourless oil (81.8 mg, 72%). **^{1}H NMR** (500 MHz, $CDCl_3$) δ (ppm): 9.21 (s, 2H), 8.48 (t, *J* = 1.6 Hz, 1H), 8.40 (dt, *J* = 7.7, 1.5 Hz, 1H), 7.54 (ddd, *J* = 7.8, 2.0, 1.3 Hz, 1H), 7.49 (td, *J* = 7.7, 0.6 Hz, 1H), 4.11 (q, *J* = 7.1 Hz, 2H), 2.94 – 2.87 (m, 2H), 2.62 – 2.54 (m, 2H), 2.15 – 2.04 (m, 1H), 1.96 – 1.86 (m, 1H), 1.17 (t, *J* = 7.1 Hz, 3H). **^{13}C NMR** (126 MHz, $CDCl_3$) δ (ppm): 175.6, 171.2, 166.4 (2C), 144.8, 135.0, 131.3, 128.9, 127.3, 126.8, 61.1, 52.5, 32.5 (2C), 16.8, 14.1. **HRMS** (ESI): *m/z* calcd. for $C_{16}H_{17}N_3O_2$ $[M+H]^+$: 284.1399, found: 284.1393.

Ethyl 1-(3-(9-*iso*-propyl-9*H*-purin-6-yl)phenyl)cyclobutane-1-carboxylate (3z)

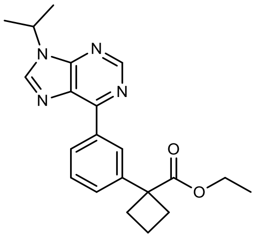

Prepared according to **General Procedure A**, using 9-*iso*-propyl-6-phenyl-9*H*-purine (95.3 mg, 0.40 mmol) as substrate and ethyl 1-bromocyclobutane-1-carboxylate (194 µL, 1.20 mmol) as alkyl bromide. The crude reaction mixture was analysed by LC-MS using an acidic mobile phase at pH 3 and the UV chromatograms are shown below:

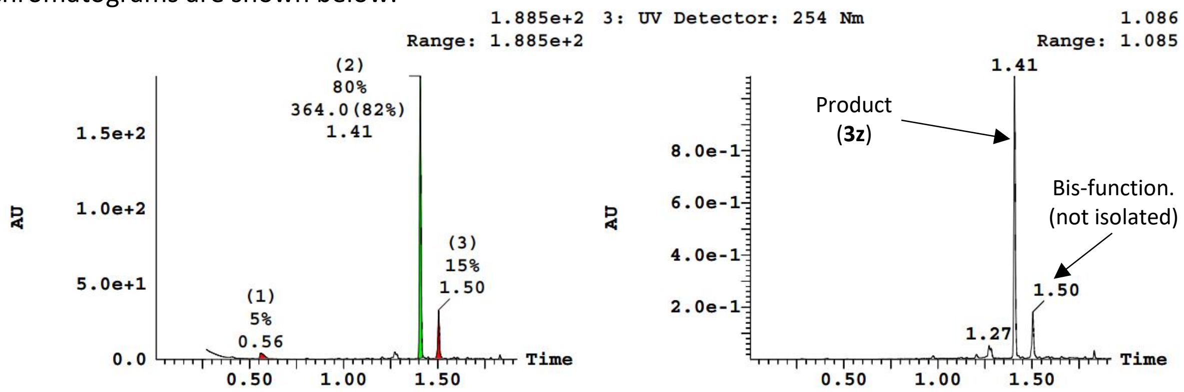

Supplementary Fig. 34. UV chromatograms (TIC & 254 nm) of the crude reaction mixture forming product **3z**.

Purification by automated flash column chromatography (0-30% EtOAc in *n*-heptane, 25 g SiO_2). **3z** was isolated as a white solid (118.3 mg, 81%). **^{1}H NMR** (500 MHz, $CDCl_3$) δ (ppm): 9.01 (s, 1H), 8.74 – 8.67 (m, 2H), 8.18 (s, 1H), 7.51 (t, *J* = 7.9 Hz, 1H), 7.46 (dt, *J* = 7.7, 1.7 Hz, 1H), 4.98 (h, *J* = 6.8 Hz, 1H), 4.10 (q, *J* = 7.1 Hz, 2H), 2.95 – 2.87 (m, 2H), 2.68 – 2.59 (m, 2H), 2.14 – 2.03 (m, 1H), 1.97 – 1.88 (m, 1H), 1.67 (d, *J* = 6.9 Hz, 6H), 1.16 (t, *J* = 7.1 Hz, 3H). **^{13}C NMR** (126 MHz, $CDCl_3$) δ (ppm): 175.8, 154.7, 152.2, 152.0, 144.3, 142.0, 135.8, 131.6, 129.0, 1y28.5, 128.4, 127.4, 60.9, 52.5, 47.2, 32.4 (2C), 22.6 (2C), 16.7, 14.1. **HRMS** (ESI): *m/z* calcd. for $C_{21}H_{24}N_4O_2$ $[M+H]^+$: 365.1977, found: 365.1995.

Diethyl 1,1'-(1-(pyridin-2-yl)-1*H*-indole-4,6-diyl)bis(cyclobutane-1-carboxylate) (3za)

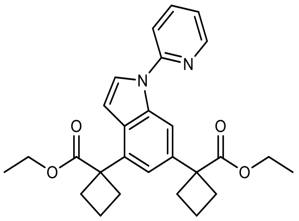

Prepared according to **General Procedure A**, using 1-(pyridin-2-yl)-1*H*-indole (77.7 mg, 0.40 mmol) as substrate and ethyl 1-bromocyclobutane-1-carboxylate (194 µL, 1.20 mmol) as alkyl bromide. The crude reaction mixture was analysed by LC-MS using an acidic mobile phase at pH 3 and the UV chromatograms are shown below:

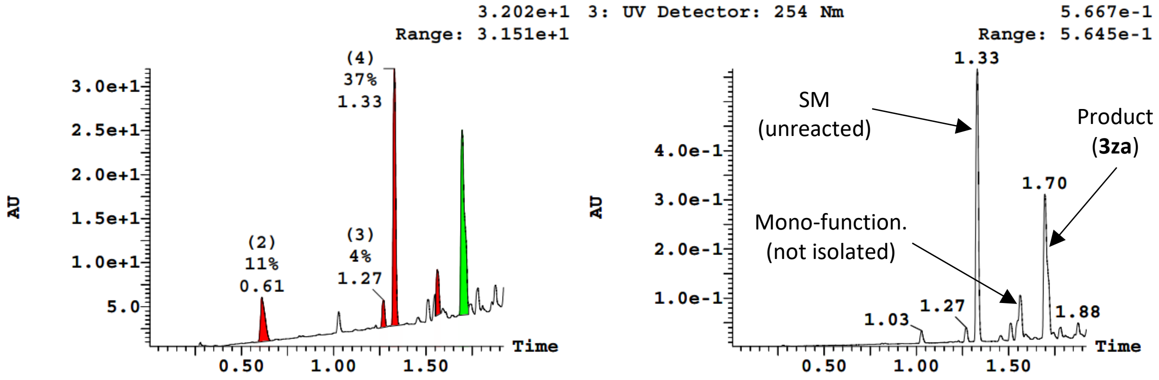

Supplementary Fig. 35. UV chromatograms (TIC & 254 nm) of the crude reaction mixture forming product **3za**.

Purification by automated flash column chromatography (0-15% EtOAc in *n*-heptane, 25 g SiO_2). **3za** was isolated as a colourless oil (69.4 mg, 39%). ^{1}**H NMR** (500 MHz, $CDCl_3$) δ (ppm): 8.57 (ddd, *J* = 4.9, 1.9, 0.9 Hz, 1H), 7.99 (dd, *J* = 1.5, 0.9 Hz, 1H), 7.82 (ddd, *J* = 8.2, 7.4, 2.0 Hz, 1H), 7.64 (d, *J* = 3.5 Hz, 1H), 7.46 (dt, *J* = 8.2, 0.9 Hz, 1H), 7.17 (ddd, *J* = 7.4, 4.9, 1.0 Hz, 1H), 7.09 (d, *J* = 1.5 Hz, 1H), 6.66 (dd, *J* = 3.5, 0.9 Hz, 1H), 4.10 (q, *J* = 7.1 Hz, 2H), 4.07 (q, *J* = 7.1 Hz, 2H), 3.02 – 2.94 (m, 2H), 2.93 – 2.85 (m, 2H), 2.74 – 2.66 (m, 2H), 2.64 – 2.56 (m, 2H), 2.20 – 2.10 (m, 1H), 2.09 – 1.99 (m, 1H), 1.95 – 1.86 (m, 2H), 1.18 (t, *J* = 7.1 Hz, 3H), 1.13 (t, *J* = 7.1 Hz, 3H). ^{13}**C NMR** (126 MHz, $CDCl_3$) δ (ppm): 176.5, 176.0, 152.6, 149.2, 138.8, 138.5, 135.8, 135.4, 126.9, 126.0, 120.3, 118.1, 115.1, 109.3, 105.1, 61.0, 60.9, 53.0, 52.9, 32.8 (2C), 32.5 (2C), 17.3, 16.7, 14.2, 14.2. **HRMS** (ESI): *m/z* calcd. for $C_{27}H_{30}N_2O_4$ $[M+H]^+$: 447.2284, found: 447.2292.

Ethyl 1-(3-(pyrimidin-2-ylamino)phenyl)cyclobutane-1-carboxylate (3zb)

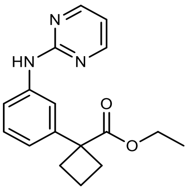

Prepared according to **General Procedure A**, using *N*-phenylpyrimidin-2-amine (68.5 mg, 0.40 mmol) as substrate and ethyl 1-bromocyclobutane-1-carboxylate (194 µL, 1.20 mmol) as alkyl bromide. The crude reaction mixture was analysed by LC-MS using an acidic mobile phase at pH 3 and the UV chromatograms are shown below:

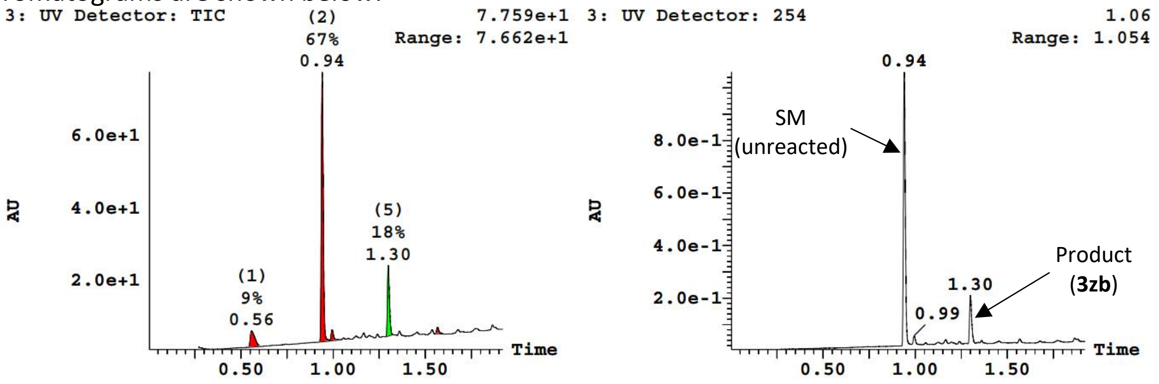

Supplementary Fig. 36. UV chromatograms (TIC & 254 nm) of the crude reaction mixture forming product **3zb**.

Purification by automated flash column chromatography (0-20% EtOAc in *n*-heptane, 25 g SiO_2). **3zb** was isolated as a colourless oil (20.6 mg, 17%). ^{1}**H NMR** (500 MHz, $CDCl_3$) δ (ppm): 8.41 (d, *J* = 4.8 Hz, 2H), 7.62 (ddd, *J* = 8.1, 2.3, 1.0 Hz, 1H), 7.43 (t, *J* = 2.0 Hz, 1H), 7.31 (br s, 1H), 7.30 (t, *J* = 7.9 Hz, 1H), 7.00 (ddd, *J* = 7.7, 1.8, 1.0 Hz, 1H), 6.71 (t, *J* = 4.8 Hz, 1H), 4.11 (q, *J* = 7.1 Hz, 2H), 2.87 – 2.79 (m, 2H), 2.57 – 2.48 (m, 2H), 2.08 – 1.97 (m, 1H), 1.93 – 1.83 (m, 1H), 1.19 (t, *J* = 7.1 Hz, 3H). ^{13}**C NMR** (126 MHz, $CDCl_3$) δ (ppm): 176.0, 160.3, 158.1 (2C), 144.8, 139.4, 128.9, 120.9, 118.0, 117.8, 112.7, 61.1, 52.6, 32.5 (2C), 16.7, 14.2. **HRMS** (ESI): *m/z* calcd. for $C_{17}H_{19}N_3O_2$ $[M+H]^+$: 298.1555, found: 298.1563.

Ethyl 1-(3-(1-methyl-1*H*-1,2,3-triazol-4-yl)phenyl)cyclobutane-1-carboxylate (3zc)

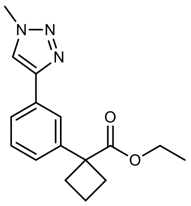

Prepared according to **General Procedure A**, using 1-methyl-4-phenyl-1*H*-1,2,3-triazole (63.7 mg, 0.40 mmol) as substrate and ethyl 1-bromocyclobutane-1-carboxylate (194 µL, 1.20 mmol) as alkyl bromide. The crude reaction mixture was analysed by LC-MS using an acidic mobile phase at pH 3 and the UV chromatograms are shown below:

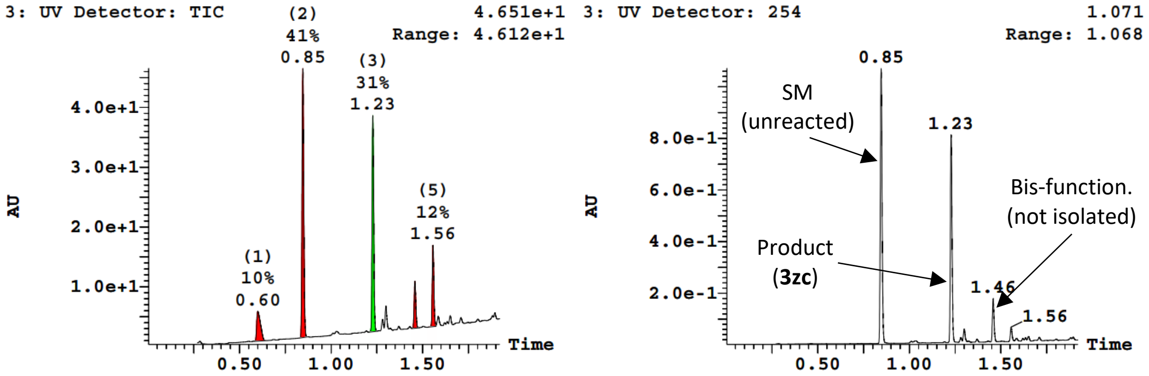

Supplementary Fig. 37. UV chromatograms (TIC & 254 nm) of the crude reaction mixture forming product **3zc**.

Purification by automated flash column chromatography (0-50% EtOAc in *n*-heptane, 25 g SiO_2). **3zc** was isolated as a colourless oil (48.4 mg, 42%). **^{1}H NMR** (500 MHz, $CDCl_3$) δ (ppm): 7.78 – 7.73 (m, 2H), 7.68 (dt, *J* = 7.7, 1.4 Hz, 1H), 7.37 (t, *J* = 7.7 Hz, 1H), 7.29 – 7.26 (m, 1H), 4.14 (s, 3H), 4.10 (q, *J* = 7.1 Hz, 2H), 2.89 – 2.82 (m, 2H), 2.59 – 2.51 (m, 2H), 2.10 – 2.00 (m, 1H), 1.93 – 1.83 (m, 1H), 1.17 (t, *J* = 7.1 Hz, 3H). **^{13}C NMR** (126 MHz, $CDCl_3$) δ (ppm): 175.8, 148.0, 144.6, 130.6, 128.8, 126.2, 124.0, 123.7, 120.8, 61.0, 52.5, 36.8, 32.4 (2C), 16.7, 14.1. **HRMS** (ESI): *m/z* calcd. for $C_{16}H_{19}N_3O_2$ $[M+H]^+$: 286.1555, found: 286.1553.

Ethyl 1-(3-(1*H*-benzo[*d*]imidazol-2-yl)phenyl)cyclobutane-1-carboxylate (3zd)

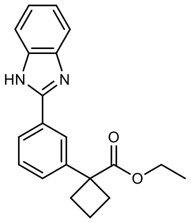

Prepared according to **General Procedure A**, using 2-phenyl-1*H*-benzo[*d*]imidazole (77.7 mg, 0.40 mmol) as substrate and ethyl 1-bromocyclobutane-1-carboxylate (194 µL, 1.20 mmol) as alkyl bromide. The crude reaction mixture was analysed by LC-MS using an acidic mobile phase at pH 3 and the UV chromatograms are shown below:

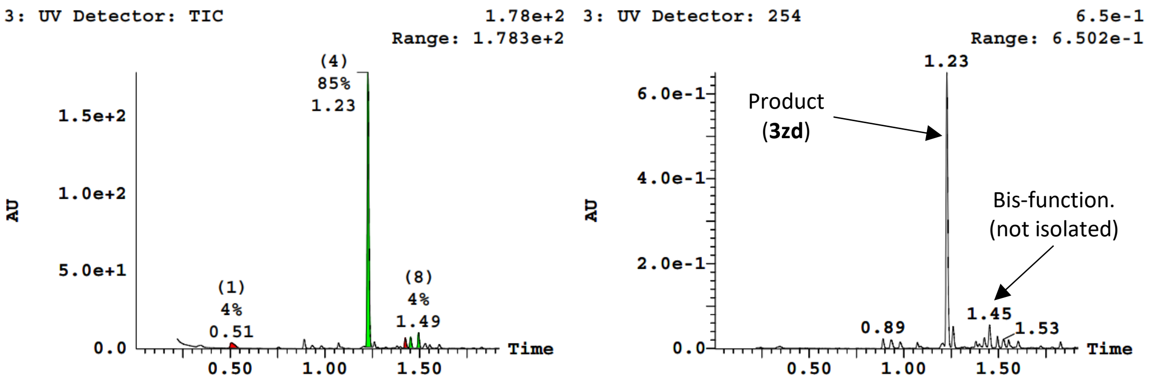

Supplementary Fig. 38. UV chromatograms (TIC & 254 nm) of the crude reaction mixture forming product **3zd**.

Purification by automated flash column chromatography (0-30% EtOAc in *n*-heptane, 25 g SiO_2). **3zd** was isolated as a white solid (107.9 mg, 84%). **^{1}H NMR** (500 MHz, $CDCl_3$) δ (ppm): 8.13 (dt, *J* = 2.4, 1.0 Hz, 1H), 8.04 (ddd, *J* = 5.5, 3.3, 1.8 Hz, 1H), 7.60 (dd, *J* = 6.0, 3.1 Hz, 2H), 7.33 – 7.28 (m, 2H), 7.21 (dd, *J* = 6.1, 3.1 Hz, 2H), 3.98 (q, *J* = 7.1 Hz, 2H), 2.70 – 2.62 (m, 2H), 2.38 – 2.29 (m, 2H), 1.99 – 1.88 (m, 1H), 1.75 – 1.65 (m, 1H), 1.05 (t, *J* = 7.1 Hz, 3H). **^{13}C NMR** (126 MHz, $CDCl_3$) δ (ppm): 175.7, 152.5, 145.1, 139.3 (2C), 130.0, 129.1, 128.3, 125.3, 125.0, 123.0 (2C), 115.2 (2C), 61.1, 52.4, 32.1 (2C), 16.7, 14.0. **HRMS** (ESI): *m/z* calcd. for $C_{20}H_{20}N_2O_2$ $[M+H]^+$: 321.1603, found: 321.1596.

Ethyl 1-(3-(thiazol-2-yl)phenyl)cyclobutane-1-carboxylate (3ze)

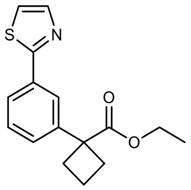

Prepared according to **General Procedure A**, using 2-phenylthiazole (55.1 µL, 0.40 mmol) as substrate and ethyl 1-bromocyclobutane-1-carboxylate (194 µL, 1.20 mmol) as alkyl bromide. The crude reaction mixture was analysed by LC-MS using an acidic mobile phase at pH 3 and the UV chromatograms are shown below:

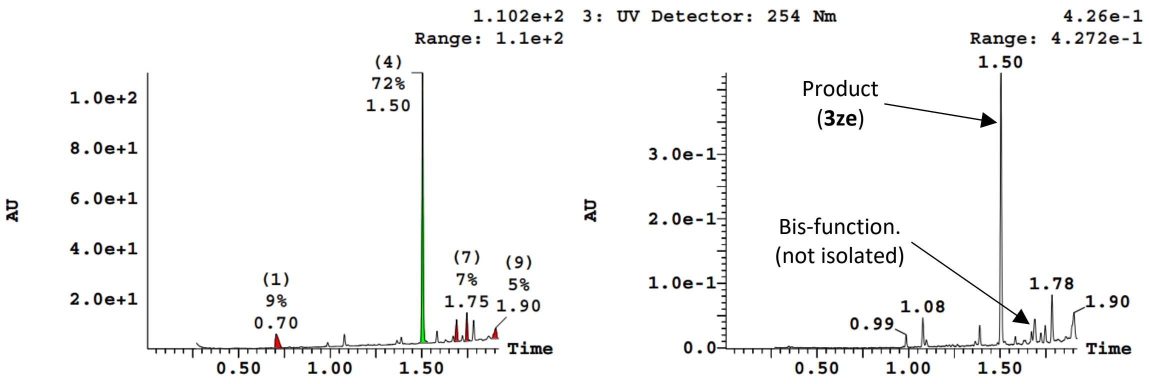

Supplementary Fig. 39. UV chromatograms (TIC & 254 nm) of the crude reaction mixture forming product **3ze**.

Purification by automated flash column chromatography (0-15% EtOAc in *n*-heptane, 25 g SiO_2). **3ze** was isolated as a colourless oil (102.2 mg, 89%). **^{1}H NMR** (500 MHz, $CDCl_3$) δ (ppm): 7.92 (t, *J* = 1.8 Hz, 1H), 7.87 (d, *J* = 3.3 Hz, 1H), 7.81 (dt, *J* = 7.5, 1.6 Hz, 1H), 7.40 (t, *J* = 7.5 Hz, 1H), 7.36 (dt, *J* = 7.8, 1.6 Hz, 1H), 7.33 (d, *J* = 3.3 Hz, 1H), 4.11 (q, *J* = 7.1 Hz, 2H), 2.92 – 2.83 (m, 2H), 2.61 – 2.52 (m, 2H), 2.13 – 2.02 (m, 1H), 1.95 – 1.85 (m, 1H), 1.18 (t, *J* = 7.1 Hz, 3H). **^{13}C NMR** (126 MHz, $CDCl_3$) δ (ppm): 175.6, 168.5, 144.9, 143.8, 133.6, 129.0, 128.2, 125.0, 124.5, 118.9, 61.1, 52.4, 32.4 (2C), 16.8, 14.1. **HRMS** (ESI): *m/z* calcd. for $C_{16}H_{17}NO_2S$ $[M+H]^+$: 288.1058, found: 288.1054.

4.2. Late-stage functionalisation (5a-5l)

Ethyl 1-(2-(2-(methyl(4-methyl-5-sulfamoylthiazol-2-yl)amino)-2-oxoethyl)-5-(pyridin-2-yl)phenyl)cyclobutane-1-carboxylate (5a)

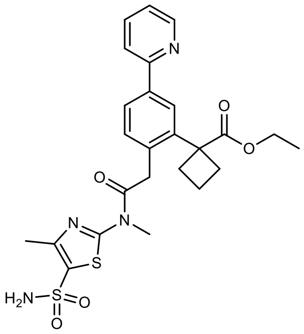

Prepared according to **General Procedure B**, using *N*-methyl-*N*-(4-methyl-5-sulfamoylthiazol-2-yl)-2-(4-(pyridin-2-yl)phenyl)acetamide (*Pritelivir*, 80.5 mg, 0.20 mmol) as LSF substrate and ethyl 1-bromocyclobutane-1-carboxylate (97.1 µL, 0.60 mmol) as alkyl bromide. The crude reaction mixture was analysed by LC-MS using an acidic mobile phase at pH 3 and the UV chromatograms are shown below:

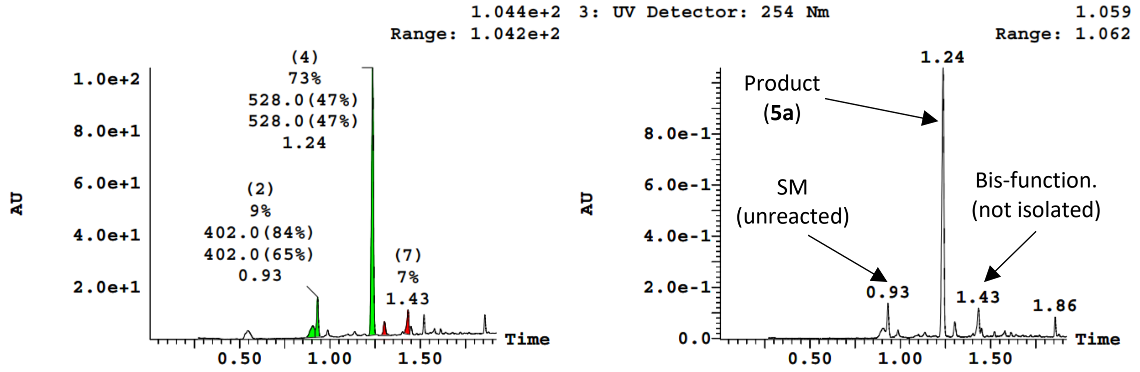

Supplementary Fig. 40. UV chromatograms (TIC & 254 nm) of the crude reaction mixture forming product **5a**.

Purification by preparative reverse phase HPLC (20-80% MeCN in acidic HCO_2H buffer, 254 nm). **5a** was isolated as a white solid (68.3 mg, 64%). ^{1}**H NMR** (500 MHz, $CDCl_3$) δ (ppm): 8.71 (ddd, *J* = 4.9, 1.8, 0.9 Hz, 1H), 7.93 (d, *J* = 1.9 Hz, 1H), 7.81 – 7.75 (m, 2H), 7.73 (dt, *J* = 8.1, 1.1 Hz, 1H), 7.29 – 7.23 (m, 1H), 7.15 (d, *J* = 8.0 Hz, 1H), 5.39 (s, 2H), 4.07 (q, *J* = 7.1 Hz, 2H), 4.03 (s, 2H), 3.68 (s, 3H), 2.87 – 2.79 (m, 2H), 2.68 – 2.59 (m, 2H), 2.54 (s, 3H), 2.18 – 2.07 (m, 1H), 1.89 – 1.80 (m, 1H), 1.15 (t, *J* = 7.1 Hz, 3H). ^{13}**C NMR** (126 MHz, $CDCl_3$) δ (ppm): 175.8, 171.6, 159.8, 156.9, 150.7, 149.6, 142.5, 138.7, 137.2, 132.0, 131.6, 127.1, 126.7, 126.0, 122.5, 121.0, 61.7, 53.2, 38.9, 34.4, 33.0 (2C), 17.4, 16.6, 14.2. **HRMS** (ESI): *m/z* calcd. for $C_{25}H_{28}N_4O_5S_2$ $[M+H]^+$: 529.1579, found: 529.1556.

Ethyl 1-(2-((5*S*,8*S*,9*S*)-8-benzyl-5-(*tert*-butyl)-9-hydroxy-11-((*S*)-2-((methoxycarbonyl)amino)-3,3-dimethylbutanamido)-3,6-dioxo-2-oxa-4,7,11-triazadodecan-12-yl)-5-(pyridin-2-yl)phenyl)cyclobutane-1-carboxylate (5b)

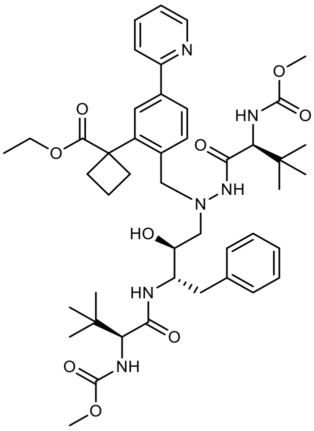

Prepared according to **General Procedure B**, using methyl ((5*S*,10*S*,11*S*,14*S*)-11-benzyl-5-(*tert*-butyl)-10-hydroxy-15,15-dimethyl-3,6,13-trioxo-8-(4-(pyridin-2-yl)benzyl)-2-oxa-4,7,8,12-tetraazahexadecan-14-yl)carbamate (*Atazanavir*, 141.0 mg, 0.20 mmol) as LSF substrate and ethyl 1-bromocyclobutane-1-carboxylate (97.1 µL, 0.60 mmol) as alkyl bromide. The crude reaction mixture was analysed by LC-MS using an acidic mobile phase at pH 3 and the UV chromatograms are shown below:

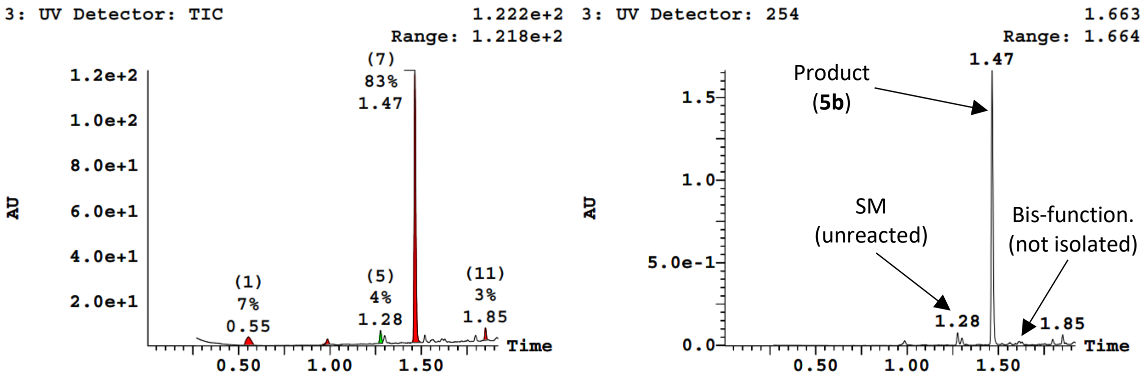

Supplementary Fig. 41. UV chromatograms (TIC & 254 nm) of the crude reaction mixture forming product **5b**.

Purification by preparative reverse phase HPLC (35-95% MeCN in acidic HCO_2H buffer, 254 nm). **5b** was isolated as a white solid (148.2 mg, 89%). **^{1}H NMR** (500 MHz, $CDCl_3$) δ (ppm): 8.67 (dd, *J* = 4.9, 1.8 Hz, 1H), 7.81 (s, 1H), 7.78 – 7.70 (m, 3H), 7.65 (d, *J* = 7.9 Hz, 1H), 7.24 – 7.18 (m, 5H), 7.18 – 7.10 (m, 2H), 6.42 (br d, *J* = 9.2 Hz, 1H), 5.29 (br d, *J* = 9.4 Hz, 1H), 5.24 (br d, *J* = 8.8 Hz, 1H), 5.11 (br s, 1H), 4.21 (dq, *J* = 10.8, 7.1 Hz, 1H), 4.12 (dq, *J* = 10.9, 7.1 Hz, 1H), 4.08 – 3.98 (m, 2H), 3.92 (d, *J* = 13.8 Hz, 1H), 3.80 (d, *J* = 8.9 Hz, 1H), 3.66 (s, 3H), 3.64 – 3.47 (m, 5H), 3.01 – 2.89 (m, 3H), 2.87 – 2.74 (m, 2H), 2.70 – 2.52 (m, 3H), 2.22 (h, *J* = 10.2 Hz, 1H), 1.90 – 1.82 (m, 1H), 1.27 (t, *J* = 7.1 Hz, 3H), 0.87 (s, 9H), 0.56 (s, 9H). **^{13}C NMR** (126 MHz, $CDCl_3$) δ (ppm): 176.6, 171.1, 170.6, 157.1, 156.9, 156.8, 149.7, 142.6, 138.6, 138.2, 136.8, 135.9, 130.4, 129.5 (2C), 128.3 (2C), 126.3, 126.2, 125.8, 122.2, 120.6, 67.2, 63.5, 62.3, 61.6, 61.4, 58.6, 52.8, 52.4, 52.1, 38.9, 34.8, 34.3 (2C), 34.0, 33.0, 26.6 (3C), 25.9 (3C), 17.5, 14.2. **HRMS** (ESI): *m/z* calcd. for $C_{45}H_{62}N_6O_9$ $[M+H]^+$: 831.4656, found: 831.4676.

Ethyl 1-(3-(5-(*iso*-propyl(4-(2-(methylsulfonamido)-2-oxoethoxy)butyl)amino)-3-phenylpyrazin-2-yl)phenyl)cyclobutane-1-carboxylate (5c) and **ethyl 1-(3-(6-(1-(ethoxycarbonyl)cyclobutyl)-5-(*iso*-propyl(4-(2-(methylsulfonamido)-2-oxoethoxy)butyl)amino)-3-phenylpyrazin-2-yl)phenyl)cyclobutane-1-carboxylate (5c2)**

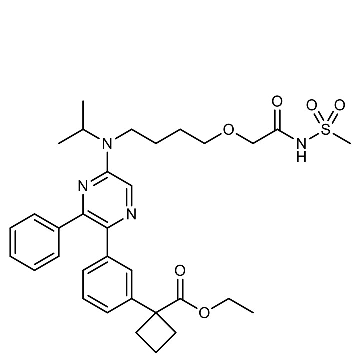

and

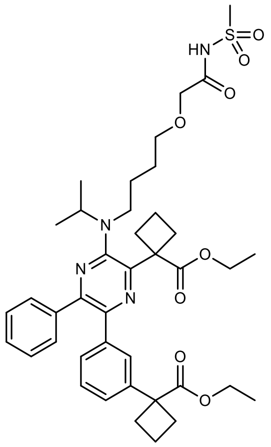

Prepared according to **General Procedure B**, using 2-(4-((5,6-diphenylpyrazin-2-yl)(*iso*-propyl)amino)butoxy)-*N*-(methylsulfonyl)acetamide (*Selexipag*, 99.3 mg, 0.20 mmol) as LSF substrate and ethyl 1-bromocyclobutane-1-carboxylate (97.1 µL, 0.60 mmol) as alkyl bromide. The crude reaction mixture was analysed by LC-MS using an acidic mobile phase at pH 3 and the UV chromatograms are shown below:

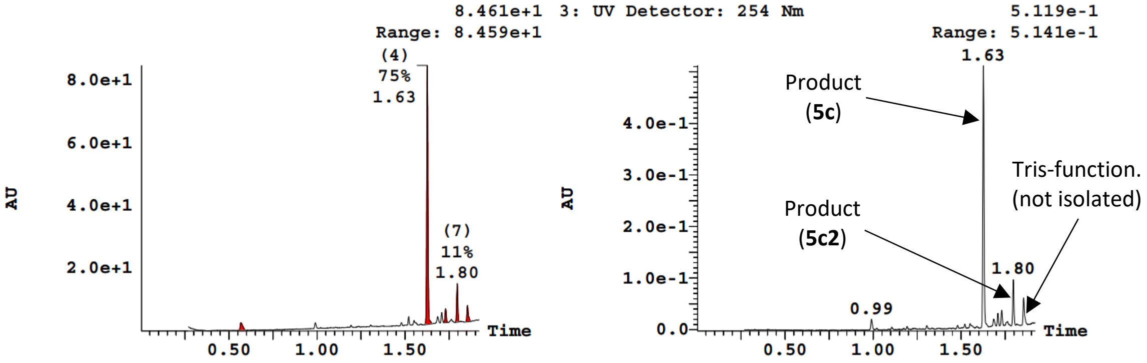

Supplementary Fig. 42. UV chromatograms (TIC & 254 nm): crude reaction mixture forming products **5c** & **5c2**.

Purification by preparative reverse phase HPLC (40-100% MeCN in acidic HCO_2H buffer, 254 nm) afforded **5c** (90.3 mg, 72%) and bis-functionalised product **5c2** (15.5 mg, 10%) as white solids.

5c: ^{1}**H NMR** (500 MHz, $CDCl_3$) δ (ppm): 8.01 (s, 1H), 7.44 – 7.39 (m, 2H), 7.36 (dt, *J* = 7.8, 1.4 Hz, 1H), 7.30 – 7.26 (m, 3H), 7.26 – 7.22 (m, 1H), 7.19 – 7.12 (m, 2H), 4.72 (h, *J* = 6.7 Hz, 1H), 4.01 (q, *J* = 7.1 Hz, 2H), 3.97 (s, 2H), 3.59 (t, *J* = 6.2 Hz, 2H), 3.46 (t, *J* = 7.4 Hz, 2H), 3.29 (s, 3H), 2.68 – 2.59 (m, 2H), 2.26 – 2.17 (m, 2H), 1.95 – 1.84 (m, 1H), 1.79 – 1.65 (m, 5H), 1.29 (d, *J* = 6.7 Hz, 6H), 1.11 (t, *J* = 7.1 Hz, 3H). ^{13}**C NMR** (126 MHz, $CDCl_3$) δ (ppm): 175.9, 169.0, 151.7, 149.3, 143.1, 139.7, 139.3, 139.2, 129.9 (2C), 128.2, 128.1, 127.9 (2C), 127.8, 127.4, 127.1, 125.0, 71.8, 69.8, 60.8, 52.1, 46.5, 42.0, 41.5, 32.1 (2C), 26.9, 25.9, 20.4 (2C), 16.5, 14.1. **HRMS** (ESI): *m/z* calcd. for $C_{33}H_{42}N_4O_6S$ $[M+H]^+$: 623.2903, found: 623.2927.

5c2: ^{1}**H NMR** (500 MHz, $CDCl_3$) δ (ppm): 8.75 (br s, 1H), 7.63 (dt, *J* = 7.7, 1.4 Hz, 1H), 7.51 (dd, *J* = 6.6, 3.0 Hz, 2H), 7.35 – 7.27 (m, 5H), 7.22 (dt, *J* = 7.8, 1.5 Hz, 1H), 4.17 (q, *J* = 7.1 Hz, 2H), 4.03 (q, *J* = 7.1 Hz, 2H), 3.79 (s, 2H), 3.46 – 3.36 (m, 3H), 3.25 (s, 3H), 3.25 – 3.18 (m, 2H), 3.06 – 2.92 (m, 2H), 2.74 – 2.61 (m, 4H), 2.27 – 2.15 (m, 3H), 2.03 – 1.94 (m, 1H), 1.93 – 1.84 (m, 1H), 1.76 – 1.67 (m, 1H), 1.54 (p, *J* = 7.0 Hz, 2H), 1.38 (p, *J* = 7.7 Hz, 2H), 1.23 (t, *J* = 7.1 Hz, 3H), 1.15 (t, *J* = 7.1 Hz, 3H), 1.10 (d, *J* = 6.5 Hz, 6H). ^{13}**C NMR** (126 MHz, $CDCl_3$) δ (ppm): 176.0, 175.3, 169.0, 152.6, 145.8, 145.7, 143.3, 143.2, 139.2, 138.8, 130.0 (2C), 128.4, 128.2 (2C), 128.1 (2C), 127.8, 125.9, 72.0, 69.7, 61.1, 60.9, 52.8, 52.4, 52.2, 42.0, 41.6, 32.2 (2C), 31.1 (2C), 27.4, 24.8, 19.8 (2C), 16.6, 16.1, 14.2, 14.1. **HRMS** (ESI): *m/z* calcd. for $C_{40}H_{52}N_4O_8S$ $[M+H]^+$: 749.3584, found: 749.3588.

Ethyl 1-(3-(7-chloro-1-methyl-2-oxo-2,3-dihydro-1*H*-benzo[*e*][1,4]diazepin-5-yl)phenyl)cyclobutane-1-carboxylate (5d)

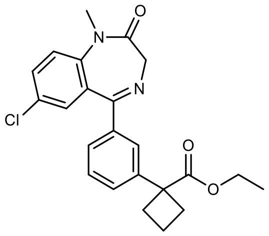

Prepared according to **General Procedure B**, using 7-chloro-1-methyl-5-phenyl-1,3-dihydro-2*H*-benzo[*e*][1,4]diazepin-2-one (*Diazepam*, 56.9 mg, 0.20 mmol) as LSF substrate and ethyl 1-bromocyclobutane-1-carboxylate (97.1 µL, 0.60 mmol) as alkyl bromide. The crude reaction mixture was analysed by LC-MS using an acidic mobile phase at pH 3 and the UV chromatograms are shown below:

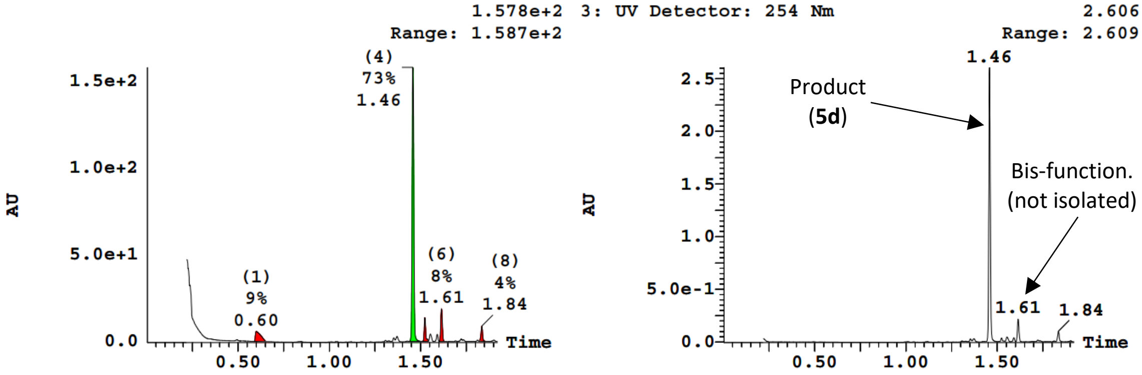

Supplementary Fig. 43. UV chromatograms (TIC & 254 nm) of the crude reaction mixture forming product **5d**.

Purification by preparative reverse phase HPLC (30-100% MeCN in acidic HCO_2H buffer, 254 nm). **5d** was isolated as a white solid (77.5 mg, 94%). **^{1}H NMR** (500 MHz, $CDCl_3$) δ (ppm): 7.55 (td, *J* = 1.9, 0.5 Hz, 1H), 7.51 (dd, *J* = 8.8, 2.5 Hz, 1H), 7.46 – 7.40 (m, 2H), 7.37 (t, *J* = 7.7 Hz, 1H), 7.30 (d, *J* = 8.9 Hz, 1H), 7.27 (d, *J* = 2.5 Hz, 1H), 4.84 (d, *J* = 10.8 Hz, 1H), 4.11 (qd, *J* = 7.1, 5.2 Hz, 2H), 3.76 (d, *J* = 10.8 Hz, 1H), 3.40 (s, 3H), 2.87 – 2.78 (m, 2H), 2.57 – 2.44 (m, 2H), 2.10 – 1.99 (m, 1H), 1.93 – 1.83 (m, 1H), 1.18 (t, *J* = 7.1 Hz, 3H). **^{13}C NMR** (126 MHz, $CDCl_3$) δ (ppm): 175.7, 170.0, 168.9, 144.2, 142.7, 138.1, 131.6, 130.1, 130.0, 129.3, 128.9, 128.4, 128.1, 127.4, 122.7, 61.1, 57.0, 52.4, 35.0, 32.6, 32.3, 16.7, 14.2. **HRMS** (ESI): *m/z* calcd. for $C_{23}H_{23}ClN_2O_3$ $[M+H]^+$: 411.1475, found: 411.1455.

Ethyl 1-(3-(9-(2-(benzyl(ethyl)amino)-2-oxoethyl)-7-methyl-8-oxo-8,9-dihydro-7*H*-purin-2-yl)phenyl)cyclobutane-1-carboxylate (5e)

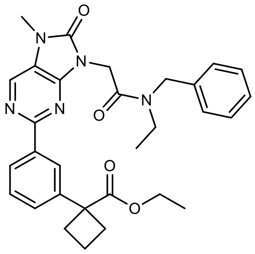

Prepared according to **General Procedure B**, using *N*-benzyl-*N*-ethyl-2-(7-methyl-8-oxo-2-phenyl-7,8-dihydro-9*H*-purin-9-yl)acetamide (*Emapunil*, 80.3 mg, 0.20 mmol) as LSF substrate and ethyl 1-bromocyclobutane-1-carboxylate (97.1 µL, 0.60 mmol) as alkyl bromide. The crude reaction mixture was analysed by LC-MS using an acidic mobile phase at pH 3 and the UV chromatograms are shown below:

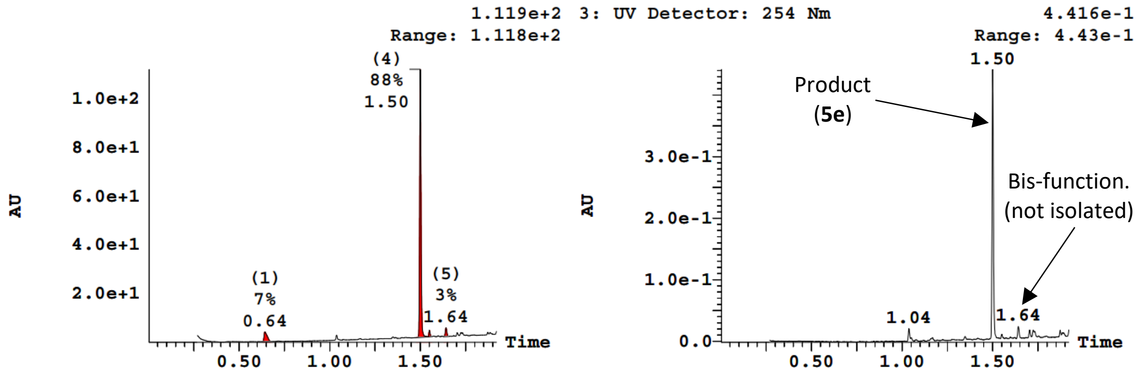

Supplementary Fig. 44. UV chromatograms (TIC & 254 nm) of the crude reaction mixture forming product **5e**.

Purification by preparative reverse phase HPLC (35-90% MeCN in acidic HCO_2H buffer, 254 nm). **5e** was isolated as a white solid (97.4 mg, 92%). **^{1}H NMR** (500 MHz, $CDCl_3$, observed as a 1:1 mixture of *E:Z* amide isomers) δ (ppm): 8.32 (t, *J* = 1.8 Hz, 0.5H), 8.32 (t, *J* = 1.8 Hz, 0.5H), 8.27 (s, 0.5H), 8.26 – 8.21 (m, 1.5H), 7.49 – 7.35 (m, 4H), 7.29 – 7.22 (m, 3H), 4.93 (s, 1H), 4.85 (s, 1H), 4.70 (s, 1H), 4.63 (s, 1H), 4.16 – 4.06 (m, 2H), 3.53 (s, 1.5H), 3.49 (s, 1.5H), 3.46 (q, *J* = 7.3 Hz, 2H), 2.95 – 2.82 (m, 2H), 2.66 – 2.54 (m, 2H), 2.15 – 2.01 (m, 1H), 1.99 – 1.84 (m, 1H), 1.37 (t, *J* = 7.1 Hz, 1.5H), 1.22 – 1.12 (m, 4.5H). **^{13}C NMR** (126 MHz, $CDCl_3$, observed as a 1:1 mixture of *E:Z* amide isomers) δ (ppm): 175.93 and 175.91 (1C), 165.51 and 165.46 (1C), 157.98 (1C), 153.64 and 153.57 (1C), 150.00 and 149.98 (1C), 144.02 (1C), 137.90 and 137.84 (1C), 137.04 and 136.03 (1C), 131.92 and 131.86 (1C), 129.20 and 128.69 (2C), 128.38 and 128.29 (1C), 128.10 and 126.34 (2C), 128.05 and 127.50 (1C), 128.01 and 127.93 (1C), 126.15 and 126.08 (1C), 125.57 (1C), 122.17 and 122.12 (1C), 60.95 and 60.92 (1C), 52.51 (1C), 50.04 and 48.77 (1C), 41.90 and 41.25 (1C), 41.42 and 40.84 (1C), 32.44 and 32.38 (2C), 27.55 and 27.49 (1C), 16.73 (1C), 14.13 (1C), 13.90 and 12.63 (1C). **HRMS** (ESI): *m/z* calcd. for $C_{30}H_{33}N_5O_4$ $[M+H]^+$: 528.2610, found: 528.2609.

Ethyl 1-(3-(5-methyl-6-((2-morpholinoethyl)amino)pyridazin-3-yl)phenyl)cyclobutane-1-carboxylate (5f) and **ethyl 1-(3-(6-((2-(3-(1-(ethoxycarbonyl)cyclobutyl)morpholino)ethyl)amino)-5-methylpyridazin-3-yl)phenyl)cyclobutane-1-carboxylate (5f2)**

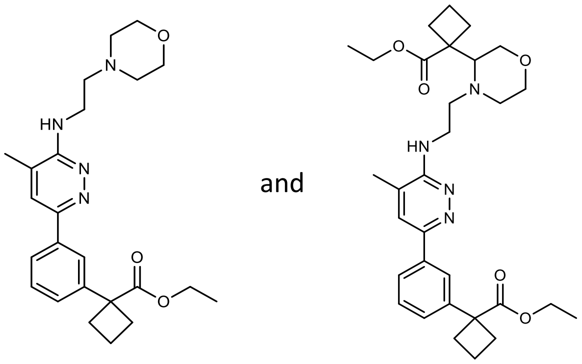

Prepared according to **General Procedure B**, using 4-methyl-*N*-(2-morpholinoethyl)-6-phenylpyridazin-3-amine (*Minaprine*, 59.7 mg, 0.20 mmol) as LSF substrate, ethyl 1-bromocyclobutane-1-carboxylate (97.1 µL, 0.60 mmol) as alkyl bromide. The crude reaction mixture was analysed by LC-MS using a basic mobile phase at pH 10 and the UV chromatograms are shown below:

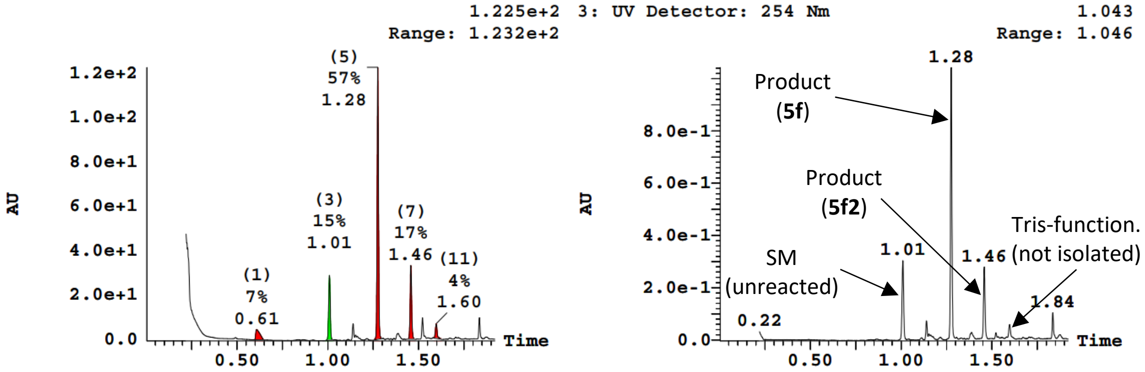

Supplementary Fig. 45. UV chromatograms (TIC & 254 nm): crude reaction mixture forming products **5f** & **5f2**.

Purification by preparative reverse phase HPLC (20-80% MeCN in basic NH_4HCO_3 buffer, 254 nm) afforded **5f** (46.9 mg, 55%) and bis-functionalised product **5f2** (13.7 mg, 12%) as colourless oils.

5f: ^{1}H NMR (500 MHz, $CDCl_3$) δ (ppm): 7.96 (t, *J* = 1.8 Hz, 1H), 7.81 (dt, *J* = 7.8, 1.3 Hz, 1H), 7.43 (d, *J* = 1.0 Hz, 1H), 7.40 (t, *J* = 7.7 Hz, 1H), 7.32 (ddd, *J* = 7.7, 1.9, 1.1 Hz, 1H), 5.19 (br s, 1H), 4.09 (q, *J* = 7.1 Hz, 2H), 3.79 – 3.70 (m, 6H), 2.90 – 2.82 (m, 2H), 2.75 (t, *J* = 5.9 Hz, 2H), 2.62 – 2.50 (m, 6H), 2.21 (d, *J* = 1.0 Hz, 3H), 2.09 – 2.01 (m, 1H), 1.93 – 1.83 (m, 1H), 1.16 (t, *J* = 7.1 Hz, 3H). **^{13}C NMR** (126 MHz, $CDCl_3$) δ (ppm): 176.0, 157.3, 151.4, 144.4, 137.2, 128.7, 126.6, 125.0, 124.3, 124.2, 124.0, 67.1 (2C), 61.0, 56.9, 53.3 (2C), 52.6, 37.8, 32.5 (2C), 16.8, 16.7, 14.2. **HRMS** (ESI): *m/z* calcd. for $C_{24}H_{32}N_4O_3$ $[M+H]^+$: 425.2552, found: 425.2555.

5f2: ^{1}H NMR (500 MHz, $CDCl_3$) δ (ppm): 8.01 (t, *J* = 2.0 Hz, 1H), 7.84 (dt, *J* = 6.5, 2.0 Hz, 1H), 7.47 (d, *J* = 1.2 Hz, 1H), 7.41 – 7.34 (m, 2H), 5.25 (br s, 1H), 4.13 (dq, *J* = 10.8, 7.2 Hz, 1H), 4.11 (q, *J* = 7.2 Hz, 2H), 3.89 (dq, *J* = 10.8, 7.2 Hz, 1H), 3.81 – 3.67 (m, 7H), 3.07 – 2.98 (m, 1H), 2.77 (t, *J* = 6.4 Hz, 2H), 2.63 – 2.50 (m, 5H), 2.29 – 2.24 (m, 1H), 2.22 (d, *J* = 1.0 Hz, 3H), 2.20 – 2.01 (m, 5H), 1.87 – 1.80 (m, 2H), 1.14 (t, *J* = 7.2 Hz, 3H), 1.10 (t, *J* = 7.1 Hz, 3H). **^{13}C NMR** (126 MHz, $CDCl_3$) δ (ppm): 176.6, 174.2, 157.3, 151.6, 145.0, 137.1, 128.6, 126.8, 125.1, 124.5, 124.2, 124.1, 67.0, 61.2, 60.6, 57.1, 55.0, 53.4, 49.2, 47.6, 37.8, 30.5 (2C), 29.9 (2C), 24.1, 18.7, 16.8, 16.0, 14.1, 13.8. **HRMS** (ESI): *m/z* calcd. for $C_{31}H_{42}N_4O_5$ $[M+H]^+$: 551.3233, found: 551.3251.

Ethyl 1-(5-(3-(2-(dimethylamino)-2-oxoethyl)-6-methylimidazo[1,2-*a*]pyridin-2-yl)-2-methylphenyl)cyclobutane-1-carboxylate (5g)

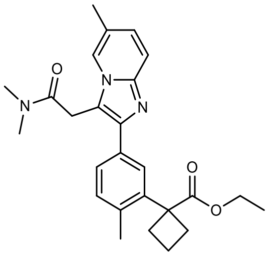

Prepared according to **General Procedure B**, using *N*,*N*-dimethyl-2-(6-methyl-2-(*p*-tolyl)imidazo[1,2-*a*]pyridin-3-yl)acetamide (*Zolpidem*, 61.5 mg, 0.20 mmol) as LSF substrate and ethyl 1-bromocyclobutane-1-carboxylate (97.1 µL, 0.60 mmol) as alkyl bromide. The crude reaction mixture was analysed by LC-MS using a basic mobile phase at pH 10 and the UV chromatograms are shown below:

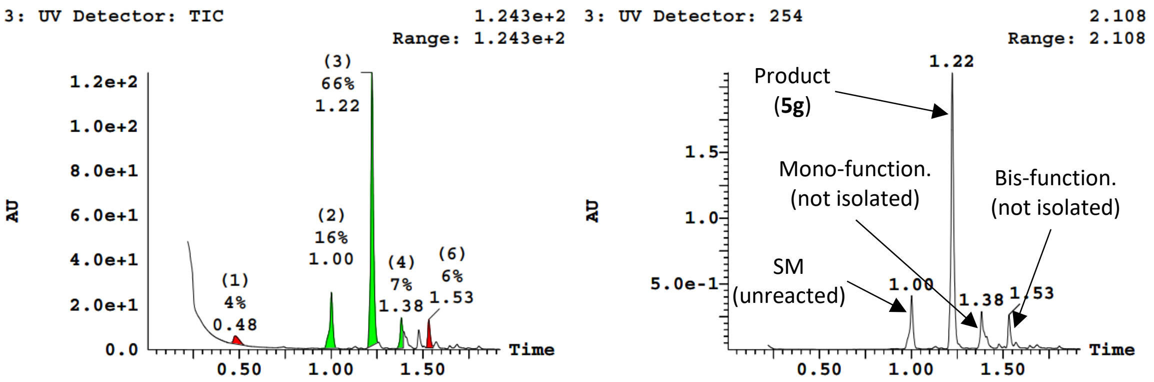

Supplementary Fig. 46. UV chromatograms (TIC & 254 nm) of the crude reaction mixture forming product **5g**.

Purification by preparative reverse phase HPLC (20-80% MeCN in basic NH_4HCO_3 buffer, 254 nm). **5g** was isolated as a pale yellow solid (53.1 mg, 61%). **^{1}H NMR** (500 MHz, $CDCl_3$) δ (ppm): 8.02 (s, 1H), 7.58 (d, *J* = 9.1 Hz, 1H), 7.48 (d, *J* = 1.8 Hz, 1H), 7.44 (dd, *J* = 7.7, 1.8 Hz, 1H), 7.18 (d, *J* = 7.7 Hz, 1H), 7.07 (dd, *J* = 9.2, 1.6 Hz, 1H), 4.12 (q, *J* = 7.1 Hz, 2H), 4.09 (s, 2H), 2.95 (s, 3H), 2.89 (s, 3H), 2.86 – 2.78 (m, 2H), 2.64 – 2.55 (m, 2H), 2.35 (s, 3H), 2.22 (s, 3H), 2.21 – 2.14 (m, 1H), 1.89 – 1.81 (m, 1H), 1.16 (t, *J* = 7.1 Hz, 3H). **^{13}C NMR** (126 MHz, $CDCl_3$) δ (ppm): 175.9, 168.4, 144.0, 143.5, 142.2, 135.9, 131.7, 131.4, 127.9, 127.3, 126.9, 122.2, 122.1, 116.4, 114.0, 61.0, 53.1, 37.6, 35.9, 32.5 (2C), 30.3, 19.7, 18.5, 17.1, 14.2. **HRMS** (ESI): *m/z* calcd. for $C_{26}H_{31}N_3O_3$ $[M+H]^+$: 434.2444, found: 434.2429.

Ethyl 1-(2-chloro-5-(3-((2-((methoxycarbonyl)amino)benzyl)oxy)-1*H*-pyrazol-1-yl)phenyl)cyclobutane-1-carboxylate (5h)

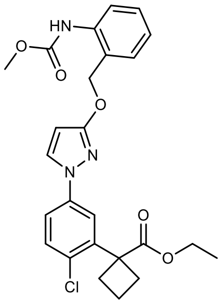

Prepared according to **General Procedure B**, using methyl (2-(((1-(4-chlorophenyl)-1*H*-pyrazol-3-yl)oxy)methyl)phenyl)(methoxy)carbamate (*Pyraclostrobin*, 77.6 mg, 0.20 mmol) as LSF substrate and ethyl 1-bromocyclobutane-1-carboxylate (97.1 µL, 0.60 mmol) as alkyl bromide. The crude reaction mixture was analysed by LC-MS using an acidic mobile phase at pH 3 and the UV chromatograms are shown below:

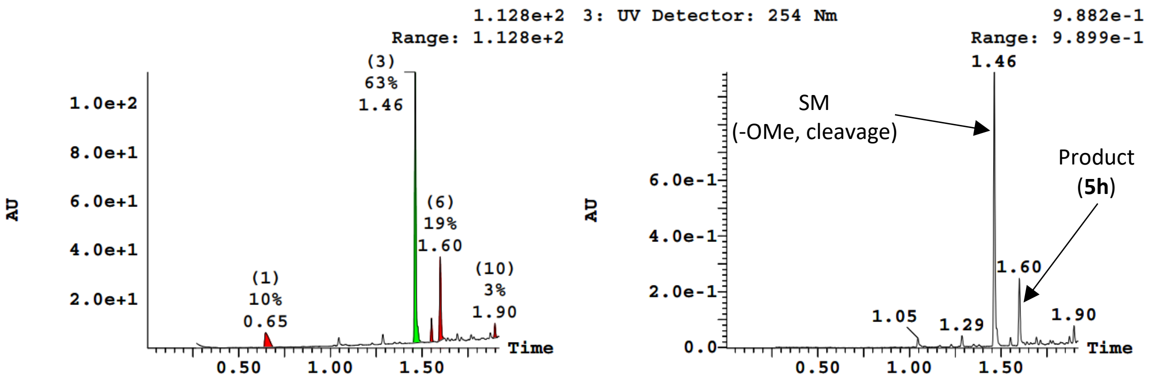

Supplementary Fig. 47. UV chromatograms (TIC & 254 nm) of the crude reaction mixture forming product **5h**.

Purification by preparative reverse phase HPLC (40-100% MeCN in acidic HCO_2H buffer, 254 nm). **5h** was isolated as a white solid (20.8 mg, 21%). **^{1}H NMR** (500 MHz, $CDCl_3$) δ (ppm): 8.16 (br s, 1H), 7.88 (br s, 1H), 7.71 (d, *J* = 2.6 Hz, 1H), 7.53 (d, *J* = 2.6 Hz, 1H), 7.46 (dd, *J* = 8.6, 2.6 Hz, 1H), 7.43 (dd, *J* = 7.6,

1.6 Hz, 1H), 7.39 – 7.34 (m, 2H), 7.11 (td, *J* = 7.5, 1.2 Hz, 1H), 5.92 (d, *J* = 2.6 Hz, 1H), 5.32 (s, 2H), 4.16 (q, *J* = 7.1 Hz, 2H), 3.74 (s, 3H), 2.93 – 2.82 (m, 2H), 2.62 – 2.52 (m, 2H), 2.31 (dp, *J* = 11.1, 8.8 Hz, 1H), 1.96 – 1.85 (m, 1H), 1.19 (t, *J* = 7.1 Hz, 3H). **^{13}C NMR** (126 MHz, $CDCl_3$) δ (ppm): 174.8, 163.8, 154.7, 142.8, 138.6, 137.5, 131.1, 130.8, 130.5, 129.9, 128.7, 126.4 (br), 124.0, 122.2 (br), 118.2, 117.9, 94.8, 68.8, 61.3, 52.6, 52.4, 31.8 (2C), 16.7, 14.2. **HRMS** (ESI): *m/z* calcd. for $C_{25}H_{26}ClN_3O_5$ $[M+H]^+$: 484.1639, found: 484.1653.

Ethyl 1-(3-(2-(3-methoxy-3-oxopropyl)-5-phenyloxazol-4-yl)phenyl)cyclobutane-1-carboxylate (5i)

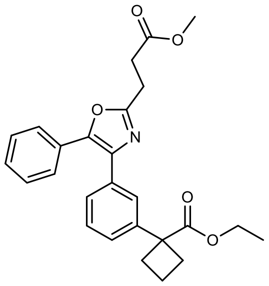

Prepared according to **General Procedure B**, using methyl 3-(4,5-diphenyloxazol-2-yl)propanoate (*Oxaprozin methyl ester*, 61.5 mg, 0.20 mmol) as LSF substrate and ethyl 1-bromocyclobutane-1-carboxylate (97.1 µL, 0.60 mmol) as alkyl bromide. The crude reaction mixture was analysed by LC-MS using an acidic mobile phase at pH 3 and the UV chromatograms are shown below:

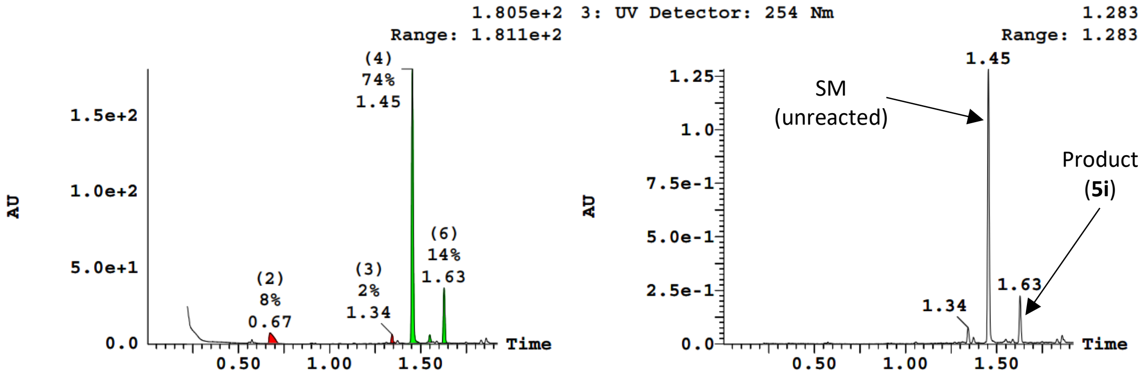

Supplementary Fig. 48. UV chromatograms (TIC & 254 nm) of the crude reaction mixture forming product **5i**.

Purification by preparative reverse phase HPLC (40-100% MeCN in acidic HCO_2H buffer, 254 nm). **5i** was isolated as a white solid (19.8 mg, 23%). **^{1}H NMR** (500 MHz, $CDCl_3$) δ (ppm): 7.60 – 7.54 (m, 3H), 7.51 (dt, *J* = 7.7, 1.5 Hz, 1H), 7.38 – 7.28 (m, 4H), 7.26 (ddd, *J* = 7.8, 1.9, 1.2 Hz, 1H), 4.08 (q, *J* = 7.1 Hz, 2H), 3.74 (s, 3H), 3.19 (dd, *J* = 8.2, 6.9 Hz, 2H), 2.92 (dd, *J* = 8.3, 6.8 Hz, 2H), 2.80 – 2.73 (m, 2H), 2.49 – 2.41 (m, 2H), 2.06 – 1.95 (m, 1H), 1.89 – 1.80 (m, 1H), 1.15 (t, *J* = 7.1 Hz, 3H). **^{13}C NMR** (126 MHz, $CDCl_3$) δ (ppm): 175.9, 172.6, 161.8, 145.7, 144.2, 135.3, 132.5, 129.1, 128.7 (2C), 128.7, 128.6, 126.6 (2C), 126.3, 126.2, 126.0, 61.0, 52.5, 52.1, 32.4 (2C), 31.1, 23.7, 16.7, 14.2. **HRMS** (ESI): *m/z* calcd. for $C_{26}H_{27}NO_5$ $[M+H]^+$: 434.1967, found: 434.1974.

Ethyl 1-(6-(4-([1,1'-biphenyl]-2-carboxamido)benzoyl)-2-methyl-1,4,5,6-tetrahydrobenzo[*b*]imidazo[4,5-*d*]azepin-7-yl)cyclobutane-1-carboxylate (5j)

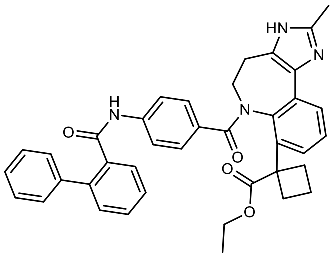

Prepared according to **General Procedure B**, using *N*-(4-(2,3-dimethyl-3,4,5,6-tetrahydrobenzo[*b*]imidazo[4,5-*d*]azepine-6-carbonyl)phenyl)-[1,1'-biphenyl]-2-carboxamide (*Conivaptan*, 99.7 mg, 0.20 mmol) as LSF substrate and ethyl 1-bromocyclobutane-1-carboxylate (97.1 µL, 0.60 mmol) as alkyl bromide. The crude reaction mixture was analysed by LC-MS using a basic mobile phase at pH 10 and the UV chromatograms are shown below:

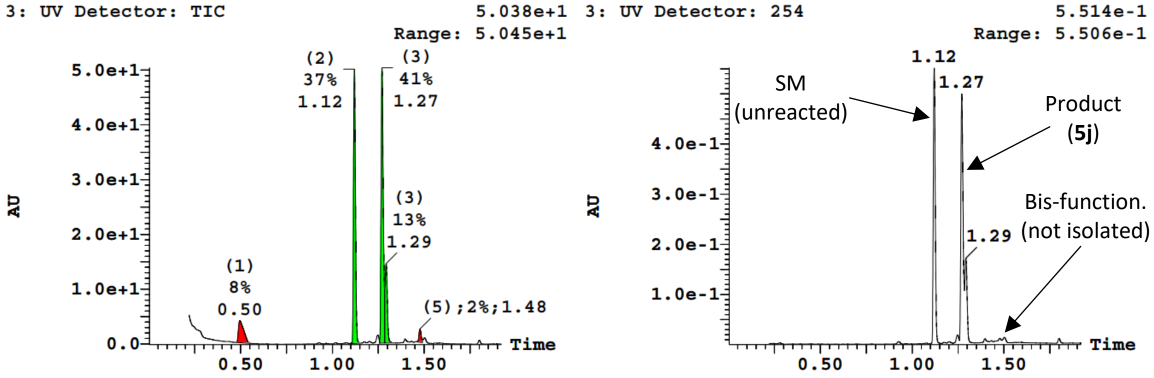

Supplementary Fig. 49. UV chromatograms (TIC & 254 nm) of the crude reaction mixture forming product **5j**.

Purification by preparative reverse phase HPLC (20-70% MeCN in basic NH_4HCO_3 buffer, 254 nm). **5j** was isolated as a white solid (49.0 mg, 39%). **^{1}H NMR** (500 MHz, DMSO-*d₆*, observed as a 4:1 mixture of *E:Z* amide isomers) δ (ppm): 11.86 (br s, 1H), 10.49 (s, 0.2H), 10.24 (s, 0.8H), 8.02 (br s, 0.8H), 7.65 (d, *J* = 8.3 Hz, 0.4H), 7.63 –7.58 (m, 0.4H), 7.55 (td, *J* = 7.5, 1.7 Hz, 0.8H), 7.52 – 7.42 (m, 3.6H), 7.41 – 7.33 (m, 3H), 7.32–7.21 (m, 3H), 7.17 (d, *J* = 8.5 Hz, 1.6H), 7.05 (dd, *J* = 7.8, 1.5 Hz, 0.8H), 6.79 (d, *J* = 8.6 Hz, 1.6H), 4.64 (dd, *J* = 13.2, 6.0 Hz, 0.8H), 4.17 (dd, *J* = 14.9, 6.2 Hz, 0.2H), 4.10 (dq, *J* = 11.0, 7.0 Hz, 0.2H), 3.98 (dq, *J* = 11.0, 7.1 Hz, 0.2H), 3.66 (dq, *J* = 10.9, 7.1 Hz, 0.8H), 3.36 – 3.27 (m, 1.8H), 3.09 – 3.00 (m, 0.2H), 2.88 (td, *J* = 13.2, 3.9 Hz, 0.8H), 2.83 – 2.71 (m, 1H), 2.65 – 2.54 (m, 1.2H), 2.43 – 2.31 (m, 2H), 2.29 (s, 2.4H), 2.26 (s, 0.6H), 2.07 – 2.00 (m, 0.2H), 1.98 – 1.86 (m, 1.6H), 1.80 – 1.74 (m, 0.2H), 1.73 – 1.64 (m, 0.8H), 1.07 (t, *J* = 7.1 Hz, 0.6H), 0.89 (t, *J* = 7.1 Hz, 2.4H). **^{13}C NMR** (126 MHz, DMSO-*d₆*, observed as a 4:1 mixture of *E:Z* amide isomers, only signals from the major isomer are reported) δ (ppm): 173.0, 168.0, 165.5, 143.6, 140.7, 139.8, 139.2, 137.3, 136.7, 133.5, 131.6, 130.1 (2C), 130.0, 129.2, 128.3, 128.22 (2C), 128.20, 127.9, 127.7, 127.4, 127.3 (2C), 127.2, 126.6, 125.3, 119.0, 117.3 (2C), 60.1, 50.8, 46.4, 35.5, 31.0, 24.0, 16.6, 13.8, 13.6. **HRMS** (ESI): *m/z* calcd. for $C_{39}H_{36}N_4O_4$ $[M+H]^+$: 625.2815, found: 625.2830.

Ethyl 1-(6-phenyl-2,3-dihydroimidazo[2,1-*b*]thiazol-5-yl)cyclobutane-1-carboxylate (5k) and **ethyl 1-(3-(5-(1-(ethoxycarbonyl)cyclobutyl)-2,3-dihydroimidazo[2,1-*b*]thiazol-6-yl)phenyl)cyclobutane-1-carboxylate (5k2)**

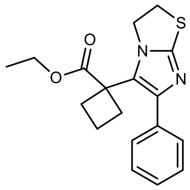

and

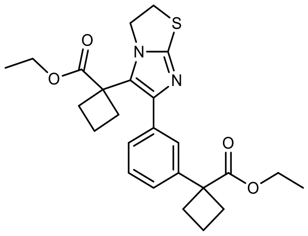

Prepared according to **General Procedure B**, using (*S*)-6-phenyl-2,3,5,6-tetrahydroimidazo[2,1-*b*]thiazole (*Levamisole*, 40.9 mg, 0.20 mmol) as LSF substrate and ethyl 1-bromocyclobutane-1-carboxylate (97.1 µL, 0.60 mmol) as alkyl bromide. The crude reaction mixture was analysed by LC-MS using an acidic mobile phase at pH 3 and the UV chromatograms are shown below:

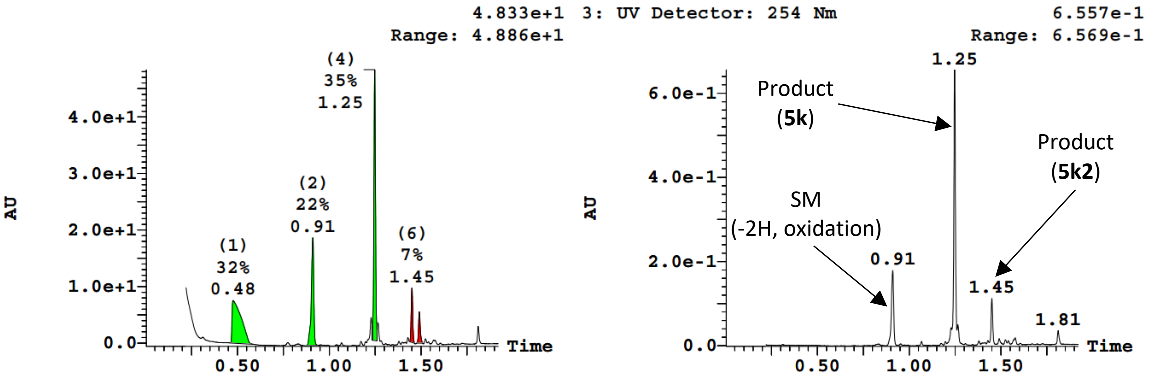

Supplementary Fig. 50. UV chromatograms (TIC & 254 nm): crude reaction mixture forming products **5k** & **5k2**.

Purification by preparative reverse phase HPLC (20-80% MeCN in acidic HCO_2H buffer, 254 nm) afforded **5k** (31.0 mg, 47%) and bis-functionalised product **5k2** (8.8 mg, 10%) as colourless oils.

5k: ^{1}**H NMR** (500 MHz, $CDCl_3$) δ (ppm): 7.52 – 7.45 (m, 2H), 7.36 – 7.30 (m, 2H), 7.30 – 7.26 (m, 1H), 4.28 (q, *J* = 7.1 Hz, 2H), 4.23 (t, *J* = 7.3 Hz, 2H), 3.83 (t, *J* = 7.3 Hz, 2H), 2.65 (tt, *J* = 8.9, 2.8 Hz, 2H), 2.28 – 2.20 (m, 2H), 2.14 – 2.04 (m, 1H), 1.76 – 1.68 (m, 1H), 1.33 (t, *J* = 7.1 Hz, 3H). ^{13}**C NMR** (126 MHz, $CDCl_3$) δ (ppm): 174.1, 149.1, 141.2 (br), 133.0 (br), 128.8 (2C), 128.1 (2C), 128.0, 127.8, 61.9, 47.3, 46.4, 35.5, 31.5 (2C), 17.2, 14.3. **HRMS** (ESI): *m/z* calcd. for $C_{18}H_{20}N_2O_2S$ $[M+H]^+$: 329.1324, found: 329.1308.

5k2: ^{1}**H NMR** (500 MHz, $CDCl_3$) δ (ppm): 7.44 (dt, *J* = 7.7, 1.4 Hz, 1H), 7.40 (t, *J* = 1.8 Hz, 1H), 7.30 (t, *J* = 7.7 Hz, 1H), 7.19 (ddd, *J* = 7.7, 1.9, 1.2 Hz, 1H), 4.29 (q, *J* = 7.1 Hz, 2H), 4.18 (t, *J* = 7.1 Hz, 2H), 4.06 (q, *J* = 7.1 Hz, 2H), 3.77 (t, *J* = 7.1 Hz, 2H), 2.85 – 2.79 (m, 2H), 2.68 (tt, *J* = 9.0, 2.9 Hz, 2H), 2.58 – 2.51 (m, 2H), 2.27 – 2.20 (m, 2H), 2.13 – 1.97 (m, 2H), 1.90 – 1.81 (m, 1H), 1.75 – 1.67 (m, 1H), 1.35 (t, *J* = 7.1 Hz, 3H), 1.13 (t, *J* = 7.1 Hz, 3H). ^{13}**C NMR** (126 MHz, $CDCl_3$) δ (ppm): 176.1, 174.5, 148.9, 143.3, 143.1, 134.8, 128.0, 127.9, 126.9, 126.8, 125.3, 61.8, 60.9, 52.6, 46.8, 46.7, 35.2, 32.4 (2C), 31.5 (2C), 17.2, 16.8, 14.4, 14.2. **HRMS** (ESI): *m/z* calcd. for $C_{25}H_{30}N_2O_4S$ $[M+H]^+$: 455.2004, found: 455.1996.

Ethyl 1-(4-(3-(cyanomethyl)phenyl)-2-((3-fluoro-4-(4-methylpiperazin-1-yl)phenyl)amino)-5-methyl-7*H*-pyrrolo[2,3-*d*]pyrimidin-6-yl)cyclobutane-1-carboxylate (5l)

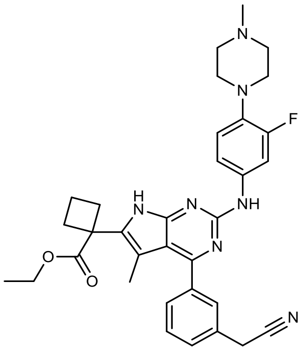

Prepared according to **General Procedure B**, using 2-(3-(2-((3-fluoro-4-(4-methylpiperazin-1-yl)phenyl)amino)-5-methyl-7*H*-pyrrolo[2,3-*d*]pyrimidin-4-yl)phenyl)acetonitrile (*SGI-7079*, 91.1 mg, 0.20 mmol) as LSF substrate and ethyl 1-bromocyclobutane-1-carboxylate (97.1 µL, 0.60 mmol) as alkyl bromide. The crude reaction mixture was analysed by LC-MS using an acidic mobile phase at pH 3 and the UV chromatograms are shown below:

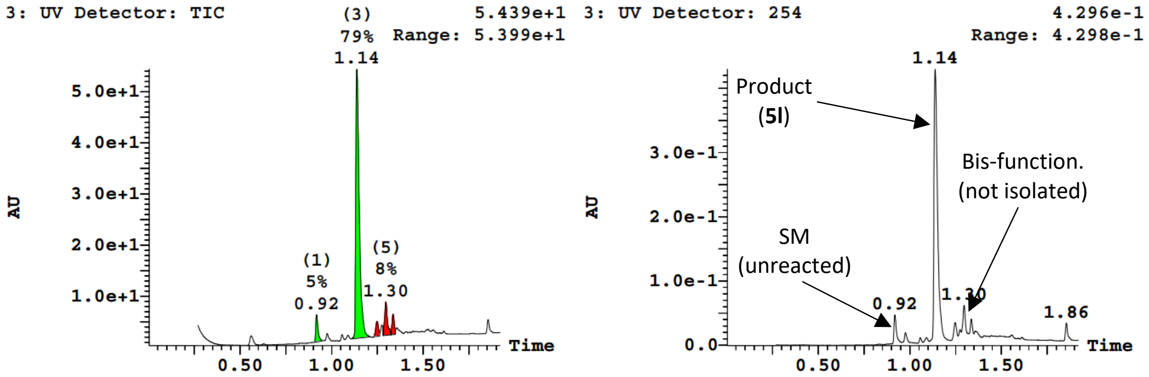

Supplementary Fig. 51. UV chromatograms (TIC & 254 nm) of the crude reaction mixture forming product **5l**.

Purification by preparative reverse phase HPLC (20-70% MeCN in acidic HCO_2H buffer, 254 nm). **5l** was isolated as a yellow solid (73.6 mg, 63%). **^{1}H NMR** (500 MHz, $CDCl_3$) δ (ppm): 9.36 (br s, 1H), 7.73 (dd, *J* = 14.8, 2.5 Hz, 1H), 7.68 – 7.59 (m, 2H), 7.51 (t, *J* = 7.7 Hz, 1H), 7.49 (br s, 1H), 7.44 (d, *J* = 7.7 Hz, 1H), 7.19 (dd, *J* = 8.7, 2.5 Hz, 1H), 6.89 (t, *J* = 9.2 Hz, 1H), 4.17 (q, *J* = 7.1 Hz, 2H), 3.85 (s, 2H), 3.11 (br s, 4H), 2.90 – 2.81 (m, 2H), 2.73 (br s, 4H), 2.69 – 2.58 (m, 2H), 2.43 (s, 3H), 2.12 (dp, *J* = 11.1, 8.9 Hz, 1H), 2.02 – 1.93 (m, 1H), 1.87 (s, 3H), 1.22 (t, *J* = 7.1 Hz, 3H). **^{13}C NMR** (126 MHz, $CDCl_3$) δ (ppm): 174.7, 159.3, 155.8 (d, $^1J_{C\text{-}F}$ = 243.9 Hz), 155.2, 153.8, 139.8, 136.5 (d, $^3J_{C\text{-}F}$ = 10.9 Hz), 134.2, 133.7 (d, $^2J_{C\text{-}F}$ = 9.6 Hz), 129.9, 129.2, 129.0, 129.0, 128.7, 119.4 (d, $^3J_{C\text{-}F}$ = 4.1 Hz), 117.8, 114.4 (d, $^4J_{C\text{-}F}$ = 3.1 Hz), 111.9, 107.5 (d, $^2J_{C\text{-}F}$ = 25.8 Hz), 107.0, 61.7, 54.9 (2C), 50.4 (2C), 48.1, 45.5, 31.6 (2C), 23.8, 17.7, 14.2, 11.8. **^{19}F NMR** (471 MHz, $CDCl_3$) δ (ppm): -122.1 (s, 1F). **HRMS** (ESI): *m/z* calcd. for $C_{33}H_{36}FN_7O_2$ [M+H]$^+$: 582.2993, found: 582.3018.

4.3. Emapunil analogues preparation (6a-6k)

Ethyl 2-(3-(9-(2-(benzyl(ethyl)amino)-2-oxoethyl)-7-methyl-8-oxo-8,9-dihydro-7*H*-purin-2-yl)phenyl)acetate (6a)

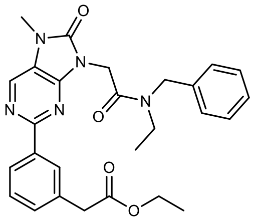

Prepared according to **General Procedure B**, using *N*-benzyl-*N*-ethyl-2-(7-methyl-8-oxo-2-phenyl-7,8-dihydro-9*H*-purin-9-yl)acetamide (*Emapunil*, 80.3 mg, 0.20 mmol) as LSF substrate and ethyl 2-bromoacetate (66.4 µL, 0.60 mmol) as alkyl bromide. The crude reaction mixture was analysed by LC-MS using an acidic mobile phase at pH 3 and the UV chromatograms are shown below:

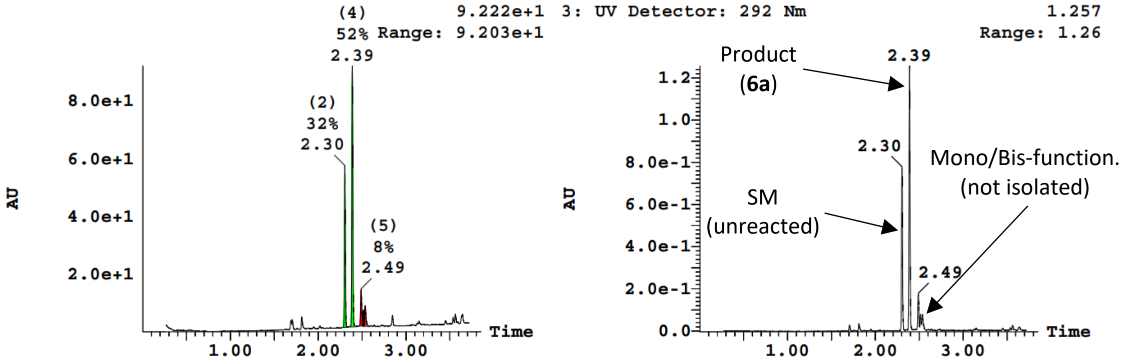

Supplementary Fig. 52. UV chromatograms (TIC & 254 nm) of the crude reaction mixture forming product **6a**.

Purification by preparative reverse phase HPLC (25-90% MeCN in acidic HCO_2H buffer, 254 nm). **6a** was isolated as a white solid (50.0 mg, 51%). **^{1}H NMR** (500 MHz, $CDCl_3$, observed as a 1:1 mixture of *E:Z* amide isomers) δ (ppm): 8.30 – 8.24 (m, 2.5H), 8.22 (s, 0.5H), 7.47 – 7.34 (m, 4H), 7.30 – 7.23 (m, 3H), 4.91 (s, 1H), 4.84 (s, 1H), 4.68 (s, 1H), 4.63 (s, 1H), 4.20 – 4.11 (m, 2H), 3.71 (s, 1H), 3.69 (s, 1H), 3.52 (s, 1.5H), 3.48 (s, 1.5H), 3.45 (q, *J* = 7.0 Hz, 2H), 1.35 (t, *J* = 7.1 Hz, 1.5H), 1.29 – 1.21 (m, 3H), 1.14 (t, *J* = 7.2 Hz, 1.5H). **^{13}C NMR** (126 MHz, $CDCl_3$, observed as a 1:1 mixture of *E:Z* amide isomers) δ (ppm): 171.65 and 171.64 (1C), 165.53 and 165.47 (1C), 157.89 and 157.83 (1C), 153.68 and 153.65 (1C), 150.12 and 150.06 (1C), 138.24 and 138.21 (1C), 137.12 and 136.06 (1C), 134.41 (1C), 131.96 and 131.89 (1C), 130.86 (1C), 129.26 and 128.77 (2C), 128.75 (1C), 128.68 (1C), 128.20 and 126.38 (2C), 128.01 and 127.59 (1C), 126.66 and 126.59 (1C), 122.28 and 122.24 (1C), 60.99 and 60.96 (1C), 50.03 and 48.82 (1C), 41.97 and 41.31 (1C), 41.57 (1C), 41.51 and 40.91 (1C), 27.62 and 27.56 (1C), 14.30 (1C), 13.93 and 12.70 (1C). **HRMS** (ESI): *m/z* calcd. for $C_{27}H_{29}N_5O_4$ $[M+H]^+$: 488.2298, found: 488.2278.

Ethyl 2-(3-(9-(2-(benzyl(ethyl)amino)-2-oxoethyl)-7-methyl-8-oxo-8,9-dihydro-7*H*-purin-2-yl)phenyl)-2-methylpropanoate (6b)

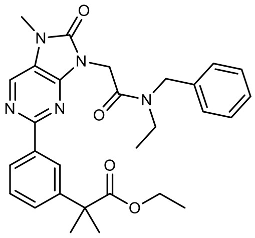

Prepared according to **General Procedure B**, using *N*-benzyl-*N*-ethyl-2-(7-methyl-8-oxo-2-phenyl-7,8-dihydro-9*H*-purin-9-yl)acetamide (*Emapunil*, 80.3 mg, 0.20 mmol) as LSF substrate and ethyl 2-bromo-2-methylpropanoate (88.1 µL, 0.60 mmol) as alkyl bromide. The crude reaction mixture was analysed by LC-MS using an acidic mobile phase at pH 3 and the UV chromatograms are shown below:

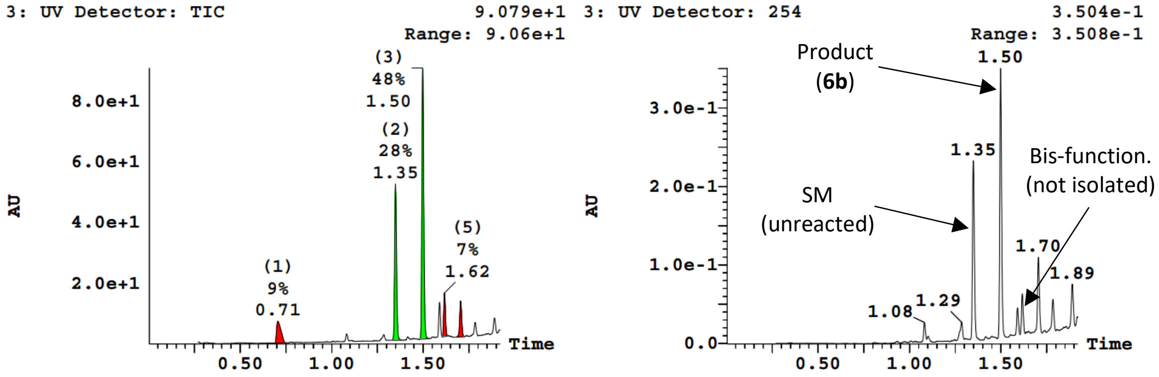

Supplementary Fig. 53. UV chromatograms (TIC & 254 nm) of the crude reaction mixture forming product **6b**.

Purification by preparative reverse phase HPLC (35-95% MeCN in acidic HCO_2H buffer, 254 nm). **6b** was isolated as a white solid (65.4 mg, 63%). ^{1}**H NMR** (500 MHz, $CDCl_3$, observed as a 1:1 mixture of *E:Z* amide isomers) δ (ppm): 8.40 – 8.35 (m, 1H), 8.26 (s, 0.5H), 8.25 – 8.20 (m, 1.5H), 7.48 – 7.39 (m, 3H), 7.38 – 7.34 (m, 1H), 7.28 – 7.21 (m, 3H), 4.91 (s, 1H), 4.84 (s, 1H), 4.68 (s, 1H), 4.62 (s, 1H), 4.18 – 4.08 (m, 2H), 3.52 (s, 1.5H), 3.49 (s, 1.5H), 3.49 – 3.41 (m, 2H), 1.65 (s, 3H), 1.63 (s, 3H), 1.36 (t, *J* = 7.2 Hz, 1.5H), 1.21 – 1.15 (m, 3H), 1.14 (t, *J* = 7.1 Hz, 1.5H). ^{13}**C NMR** (126 MHz, $CDCl_3$, observed as a 1:1 mixture of *E:Z* amide isomers) δ (ppm): 176.89 and 176.85 (1C), 165.55 and 165.48 (1C), 158.14 (1C), 153.72 and 153.66 (1C), 150.12 and 150.08 (1C), 145.15 and 145.11 (1C), 138.03 and 137.97 (1C), 137.08 and 136.06 (1C), 131.97 and 131.90 (1C), 129.28 and 128.77 (2C), 128.53 and 128.43 (1C), 128.18 and 126.40 (2C), 128.02 and 127.57 (1C), 127.59 (1C), 126.29 and 126.24 (1C), 125.06 and 125.05 (1C), 122.25 and 122.18 (1C), 60.92 and 60.90 (1C), 50.11 and 48.83 (1C), 46.70 and 46.67 (1C), 41.97 and 41.28 (1C), 41.50 and 40.93 (1C), 27.64 and 27.58 (1C), 26.71 and 26.65 (2C), 14.18 (1C), 13.95 and 12.69 (1C). **HRMS** (ESI): *m/z* calcd. for $C_{29}H_{33}N_5O_4$ $[M+H]^+$: 516.2610, found: 516.2634.

Methyl 1-(3-(9-(2-(benzyl(ethyl)amino)-2-oxoethyl)-7-methyl-8-oxo-8,9-dihydro-7*H*-purin-2-yl)phenyl)cyclopropane-1-carboxylate (6c)

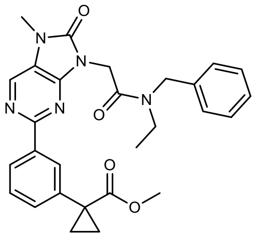

Prepared according to **General Procedure B**, using *N*-benzyl-*N*-ethyl-2-(7-methyl-8-oxo-2-phenyl-7,8-dihydro-9*H*-purin-9-yl)acetamide (*Emapunil*, 80.3 mg, 0.20 mmol) as LSF substrate, methyl 1-bromocyclopropane-1-carboxylate (62.2 µL, 0.60 mmol) as alkyl bromide and *tert*-amyl alcohol (*t*-AmOH, 2.0 mL, 0.1 M) as solvent. The crude reaction mixture was analysed by LC-MS using an acidic mobile phase at pH 3 and the UV chromatograms are shown below:

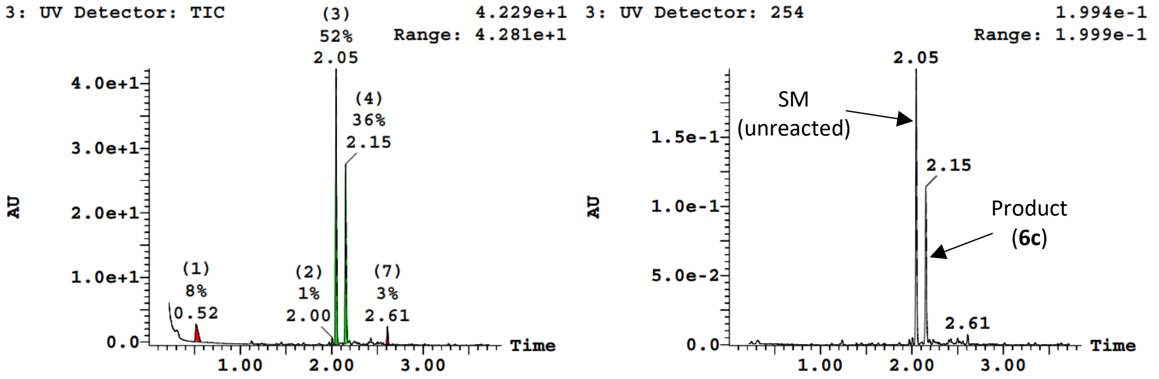

Supplementary Fig. 54. UV chromatograms (TIC & 254 nm) of the crude reaction mixture forming product **6c**.

Purification by preparative reverse phase HPLC (20-90% MeCN in acidic HCO_2H buffer, 254 nm). **6c** was isolated as a white solid (40.8 mg, 41%). **^{1}H NMR** (500 MHz, $CDCl_3$, observed as a 1:1 mixture of *E:Z* amide isomers) δ (ppm): 8.33 (t, *J* = 1.8 Hz, 0.5H), 8.32 (t, *J* = 1.8 Hz, 0.5H), 8.29 – 8.26 (m, 1H), 8.26 (s, 0.5H), 8.22 (s, 0.5H), 7.47 – 7.33 (m, 4H), 7.28 – 7.21 (m, 3H), 4.91 (s, 1H), 4.85 (s, 1H), 4.68 (s, 1H), 4.62 (s, 1H), 3.63 (s, 1.5H), 3.62 (s, 1.5H), 3.52 (s, 1.5H), 3.49 (s, 1.5H), 3.45 (q, *J* = 6.9 Hz, 2H), 1.65 (q, *J* = 3.9 Hz, 1H), 1.63 (q, *J* = 3.9 Hz, 1H), 1.36 (t, *J* = 7.1 Hz, 1.5H), 1.28 (q, *J* = 4.0 Hz, 1H), 1.25 (q, *J* = 4.0 Hz, 1H), 1.14 (t, *J* = 7.1 Hz, 1.5H). **^{13}C NMR** (126 MHz, $CDCl_3$, observed as a 1:1 mixture of *E:Z* amide isomers) δ (ppm): 175.34 and 175.31 (1C), 165.57 and 165.53 (1C), 158.01 and 157.96 (1C), 153.75 and 153.70 (1C), 150.17 and 150.14 (1C), 139.86 and 139.84 (1C), 137.96 and 137.93 (1C), 137.12 and 136.06 (1C), 132.48 and 132.43 (1C), 131.99 and 131.91 (1C), 129.90 and 129.84 (1C), 129.30 and 128.80 (2C), 128.40 and 128.33 (1C), 128.22 and 126.38 (2C), 128.06 and 127.63 (1C), 126.91 and 126.82 (1C), 122.30 and 122.26 (1C), 52.58 and 52.56 (1C), 50.10 and 48.89 (1C), 42.04 and 41.36 (1C), 41.57 and 40.96 (1C), 29.28 and 29.24 (1C), 27.68 and 27.62 (1C), 17.06 and 16.96 (2C), 13.97 and 12.75 (1C). **HRMS** (ESI): *m/z* calcd. for $C_{28}H_{29}N_5O_4$ $[M+H]^+$: 500.2298, found: 500.2303.

N-Benzyl-2-(2-(3-(1-cyanocyclobutyl)phenyl)-7-methyl-8-oxo-7,8-dihydro-9*H*-purin-9-yl)-*N*-ethylacetamide (6d)

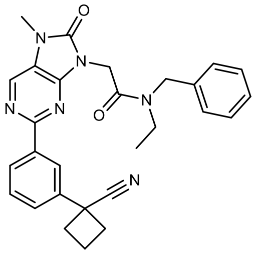

Prepared according to **General Procedure B**, using *N*-benzyl-*N*-ethyl-2-(7-methyl-8-oxo-2-phenyl-7,8-dihydro-9*H*-purin-9-yl)acetamide (*Emapunil*, 80.3 mg, 0.20 mmol) as LSF substrate and 1-bromocyclobutane-1-carbonitrile (96.0 mg, 0.60 mmol) as alkyl bromide. The crude reaction mixture was analysed by LC-MS using an acidic mobile phase at pH 3 and the UV chromatograms are shown below:

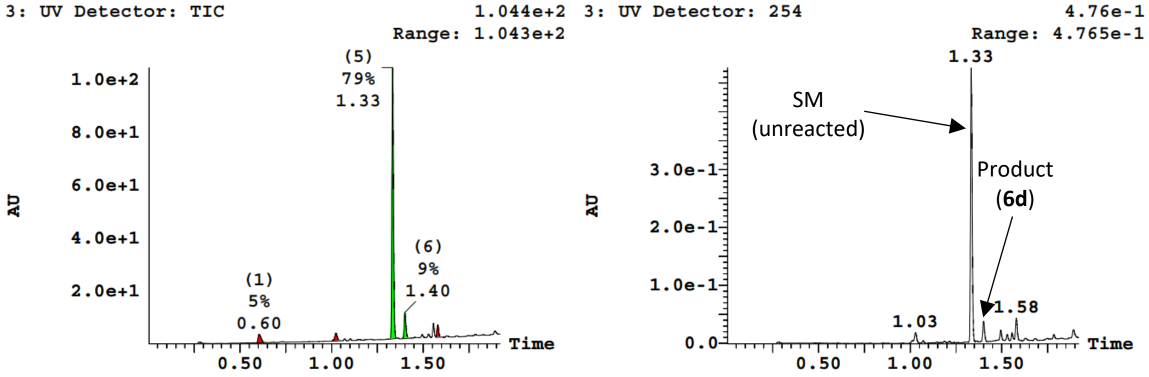

Supplementary Fig. 55. UV chromatograms (TIC & 254 nm) of the crude reaction mixture forming product **6d**.

Purification by preparative reverse phase HPLC (25-85% MeCN in acidic HCO_2H buffer, 254 nm). **6d** was isolated as a white solid (8.9 mg, 9%). **^{1}H NMR** (500 MHz, $CDCl_3$, observed as a 1:1 mixture of *E:Z* amide isomers) δ (ppm): 8.49 – 8.39 (m, 1H), 8.35 – 8.29 (m, 1H), 8.28 (s, 0.5H), 8.24 (s, 0.5H), 7.52 – 7.47 (m, 2H), 7.46 – 7.35 (m, 2H), 7.28 – 7.23 (m, 3H), 4.93 (s, 1H), 4.85 (s, 1H), 4.69 (s, 1H), 4.63 (s, 1H), 3.53 (s, 1.5H), 3.50 (s, 1.5H), 3.46 (q, *J* = 7.0 Hz, 2H), 2.93 – 2.80 (m, 2H), 2.78 – 2.66 (m, 2H), 2.52 – 2.41 (m, 1H), 2.17 – 2.06 (m, 1H), 1.36 (t, *J* = 7.1 Hz, 1.5H), 1.15 (t, *J* = 7.1 Hz, 1.5H). **^{13}C NMR** (126 MHz, $CDCl_3$, observed as a 1:1 mixture of *E:Z* amide isomers) δ (ppm): 165.51 and 165.45 (1C), 157.40 (1C), 153.71 and 153.64 (1C), 150.30 and 150.28 (1C), 140.21 and 140.15 (1C), 138.54 and 138.46 (1C), 137.10 and 136.05 (1C), 131.70 and 131.61 (1C), 129.32 and 128.81 (2C), 129.23 and 129.15 (1C), 128.23 and 126.42 (2C), 128.11 and 127.67 (1C), 127.50 and 127.43 (1C), 127.39 and 127.31 (1C), 125.09 (1C), 124.62 and 124.59 (1C), 122.54 and 122.48 (1C), 50.17 and 48.92 (1C), 42.08 and 41.38 (1C), 41.63 and

41.04 (1C), 40.43 and 40.39 (1C), 34.82 (2C), 27.75 and 27.69 (1C), 17.31 and 17.28 (1C), 14.02 and 12.74 (1C). **HRMS** (ESI): *m/z* calcd. for $C_{28}H_{28}N_6O_2$ $[M+H]^+$: 481.2352, found: 481.2338.

Methyl 1-(3-(9-(2-(benzyl(ethyl)amino)-2-oxoethyl)-7-methyl-8-oxo-8,9-dihydro-7*H*-purin-2-yl)phenyl)cyclopentane-1-carboxylate (6e)

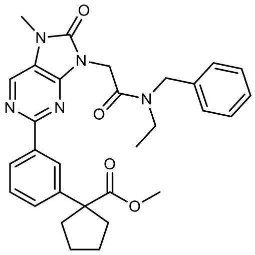

Prepared according to **General Procedure B**, using *N*-benzyl-*N*-ethyl-2-(7-methyl-8-oxo-2-phenyl-7,8-dihydro-9*H*-purin-9-yl)acetamide (*Emapunil*, 80.3 mg, 0.20 mmol) as LSF substrate and methyl 1-bromocyclopentane-1-carboxylate (88.4 µL, 0.60 mmol) as alkyl bromide. The crude reaction mixture was analysed by LC-MS using an acidic mobile phase at pH 3 and the UV chromatograms are shown below:

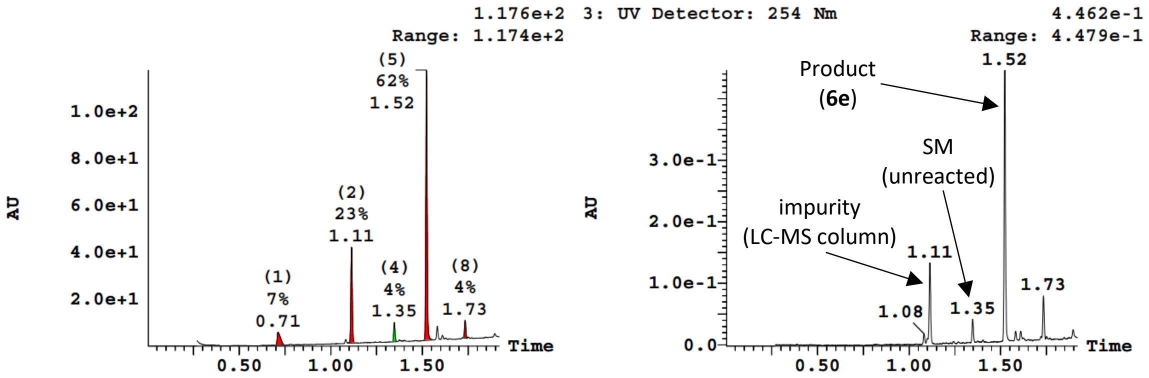

Supplementary Fig. 56. UV chromatograms (TIC & 254 nm) of the crude reaction mixture forming product **6e**.

Purification by preparative reverse phase HPLC (30-100% MeCN in acidic HCO_2H buffer, 254 nm). **6e** was isolated as a white solid (89.1 mg, 84%). **^{1}H NMR** (500 MHz, $CDCl_3$, observed as a 1:1 mixture of *E:Z* amide isomers) δ (ppm): 8.38 (s, 0.5H), 8.37 (s, 0.5H), 8.26 (s, 0.5H), 8.25 – 8.18 (m, 1.5H), 7.48 – 7.33 (m, 4H), 7.31 – 7.19 (m, 3H), 4.92 (s, 1H), 4.84 (s, 1H), 4.69 (s, 1H), 4.62 (s, 1H), 3.62 (s, 1.5H), 3.60 (s, 1.5H), 3.52 (s, 1.5H), 3.49 (s, 1.5H), 3.45 (q, *J* = 7.1 Hz, 2H), 2.77 – 2.65 (m, 2H), 2.07 – 1.94 (m, 2H), 1.81 – 1.70 (m, 4H), 1.37 (t, *J* = 7.1 Hz, 1.5H), 1.14 (t, *J* = 7.1 Hz, 1.5H). **^{13}C NMR** (126 MHz, $CDCl_3$, observed as a 1:1 mixture of *E:Z* amide isomers) δ (ppm): 176.54 and 176.52 (1C), 165.51 and 165.46 (1C), 158.03 and 158.02 (1C), 153.68 and 153.61 (1C), 150.08 and 150.06 (1C), 143.59 and 143.58 (1C), 137.86 and 137.81 (1C), 137.06 and 136.05 (1C), 131.85 and 131.78 (1C), 129.25 and 128.73 (2C), 128.71 and 128.67 (1C), 128.46 and 128.37 (1C), 128.14 and 126.38 (2C), 127.97 and 127.55 (1C), 126.32 (1C), 126.20 (1C), 122.21 and 122.15 (1C), 59.19 and 59.18 (1C), 52.45 and 52.43 (1C), 50.09 and 48.83 (1C), 41.94 and 41.29 (1C), 41.47 and 40.91 (1C), 36.36 and 36.31 (2C), 27.62 and 27.55 (1C), 23.69 (2C), 13.95 and 12.66 (1C). **HRMS** (ESI): *m/z* calcd. for $C_{30}H_{33}N_5O_4$ $[M+H]^+$: 528.2610, found: 528.2588.

Ethyl 2-(3-(9-(2-(benzyl(ethyl)amino)-2-oxoethyl)-7-methyl-8-oxo-8,9-dihydro-7*H*-purin-2-yl)phenyl)-2,2-difluoroacetate (6f)

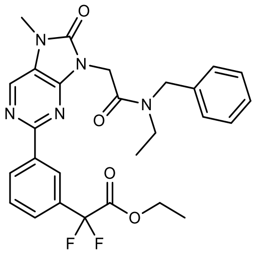

Prepared according to **General Procedure B**, using *N*-benzyl-*N*-ethyl-2-(7-methyl-8-oxo-2-phenyl-7,8-dihydro-9*H*-purin-9-yl)acetamide (*Emapunil*, 80.3 mg, 0.20 mmol) as LSF substrate and ethyl 2-bromo-2,2-difluoroacetate (76.9 µL, 0.60 mmol) as alkyl bromide. The crude reaction mixture was analysed by LC-MS using an acidic mobile phase at pH 3 and the UV chromatograms are shown below:

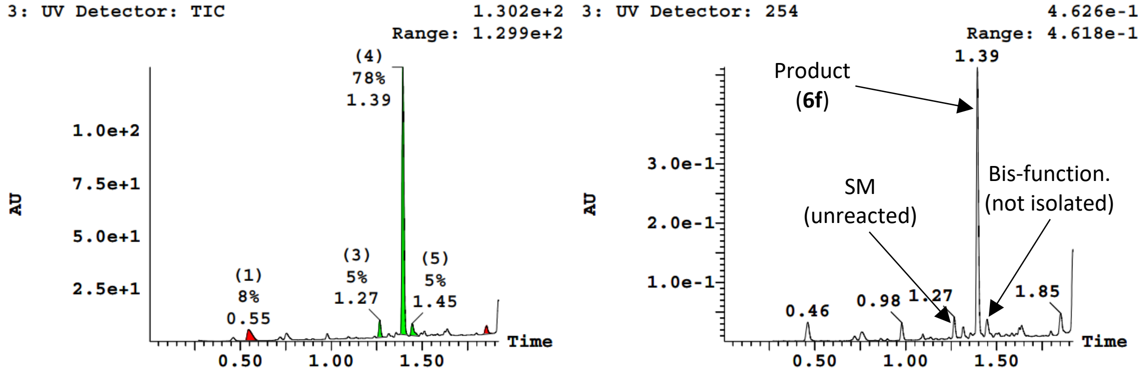

Supplementary Fig. 57. UV chromatograms (TIC & 254 nm) of the crude reaction mixture forming product **6f**.

Purification by preparative reverse phase HPLC (30-90% MeCN in acidic HCO_2H buffer, 254 nm). **6f** was isolated as a white solid (92.0 mg, 88%). **^{1}H NMR** (500 MHz, $CDCl_3$, observed as a 1:1 mixture of *E:Z* amide isomers) δ (ppm): 8.65 (t, *J* = 1.8 Hz, 0.5H), 8.63 (t, *J* = 1.8 Hz, 0.5H), 8.53 – 8.47 (m, 1H), 8.27 (s, 0.5H), 8.24 (s, 0.5H), 7.71 – 7.64 (m, 1H), 7.59 – 7.51 (m, 1H), 7.50 – 7.43 (m, 1H), 7.41 – 7.33 (m, 1.5H), 7.30 – 7.22 (m, 2.5H), 4.92 (s, 1H), 4.84 (s, 1H), 4.70 (s, 1H), 4.63 (s, 1H), 4.35 – 4.25 (m, 2H), 3.52 (s, 1.5H), 3.50 (s, 1.5H), 3.50 – 3.41 (m, 2H), 1.38 (t, *J* = 7.2 Hz, 1.5H), 1.33 – 1.26 (m, 3H), 1.15 (t, *J* = 7.2 Hz, 1.5H). **^{13}C NMR** (126 MHz, $CDCl_3$, observed as a 1:1 mixture of *E:Z* amide isomers) δ (ppm): 165.46 and 165.40 (1C), 164.23 and 164.21 (t, $^2J_{C\text{-}F}$ = 35.4 Hz, 1C), 156.73 and 156.71 (1C), 153.59 and 153.54 (1C), 150.16 and 150.14 (1C), 138.51 and 138.46 (1C), 137.01 and 135.99 (1C), 133.15 (t, $^2J_{C\text{-}F}$ = 25.5 Hz, 1C), 131.82 and 131.76 (1C), 130.31 and 130.23 (1C), 129.28 and 128.73 (2C), 128.89 and 128.82 (1C), 128.11 and 126.32 (2C), 128.03 and 127.56 (1C), 126.85 and 126.81 (t, $^3J_{C\text{-}F}$ = 5.8 Hz, 1C), 124.86 and 124.80 (t, $^3J_{C\text{-}F}$ = 6.3 Hz, 1C), 122.57 and 122.52 (1C), 113.56 and 113.52 (t, $^1J_{C\text{-}F}$ = 252.1 Hz, 1C), 63.25 and 63.22 (1C), 50.11 and 48.78 (1C), 41.99 and 41.30 (1C), 41.50 and 40.94 (1C), 27.62 and 27.56 (1C), 13.96 (1C), 13.82 and 12.64 (1C). **^{19}F NMR** (471 MHz, $CDCl_3$, observed as a 1:1 mixture of *E:Z* amide isomers) δ (ppm): -103.65 (s, 1F), -103.77 (s, 1F). **HRMS** (ESI): *m/z* calcd. for $C_{27}H_{27}F_2N_5O_4$ $[M+H]^+$: 524.2109, found: 524.2097.

Diethyl ((3-(9-(2-(benzyl(ethyl)amino)-2-oxoethyl)-7-methyl-8-oxo-8,9-dihydro-7*H*-purin-2-yl)phenyl)difluoromethyl)phosphonate (6g)

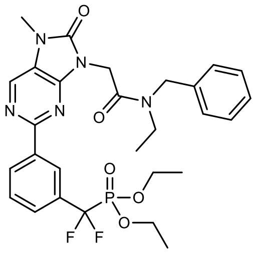

Prepared according to **General Procedure B**, using *N*-benzyl-*N*-ethyl-2-(7-methyl-8-oxo-2-phenyl-7,8-dihydro-9*H*-purin-9-yl)acetamide (*Emapunil*, 80.3 mg, 0.20 mmol) as LSF substrate and diethyl (bromodifluoromethyl)phosphonate (106.6 µL, 0.60 mmol) as alkyl bromide. The crude reaction mixture was analysed by LC-MS using an acidic mobile phase at pH 3 and the UV chromatograms are shown below:

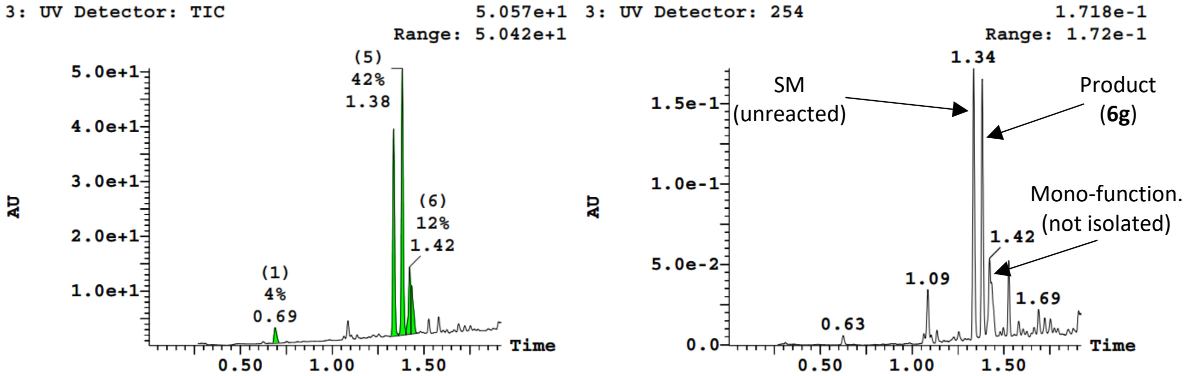

Supplementary Fig. 58. UV chromatograms (TIC & 254 nm) of the crude reaction mixture forming product **6g**.

Purification by preparative reverse phase HPLC (30-90% MeCN in acidic HCO_2H buffer, 254 nm). **6g** was isolated as a white solid (56.7 mg, 48%). **^{1}H NMR** (500 MHz, $CDCl_3$, observed as a 1:1 mixture of *E:Z* amide isomers) δ (ppm): 8.65 (d, *J* = 1.8 Hz, 0.5H), 8.64 (d, *J* = 1.8 Hz, 0.5H), 8.50 (dd, *J* = 2.7, 1.4 Hz, 0.5H), 8.49 (dd, *J* = 2.7, 1.4 Hz, 0.5H), 8.27 (s, 0.5H), 8.24 (s, 0.5H), 7.72 (dd, *J* = 5.9, 1.7 Hz, 0.5H), 7.70 (dd, *J* = 5.9, 1.7 Hz, 0.5H), 7.59 – 7.52 (m, 1H), 7.50 – 7.43 (m, 1H), 7.41 – 7.34 (m, 1.5H), 7.30 – 7.23 (m, 2.5H), 4.92 (s, 1H), 4.84 (s, 1H), 4.69 (s, 1H), 4.62 (s, 1H), 4.27 – 4.10 (m, 4H), 3.53 (s, 1.5H), 3.50 (s, 1.5H), 3.50 – 3.41 (m, 2H), 1.37 (t, *J* = 7.2 Hz, 1.5H), 1.34 – 1.27 (m, 6H), 1.14 (t, *J* = 7.2 Hz, 1.5H). **^{13}C NMR** (126 MHz, $CDCl_3$, observed as a 1:1 mixture of *E:Z* amide isomers) δ (ppm): 165.52 and 165.43 (1C), 157.01 and 156.97 (1C), 153.68 and 153.64 (1C), 150.18 and 150.15 (1C), 138.36 and 138.33 (1C), 137.04 and 136.01 (1C), 132.96 (td, $J_{C\text{-}F,C\text{-}P}$ = 21.9, 13.7 Hz, 1C), 132.00 and 131.95 (1C), 130.15 and 130.07 (1C), 129.33 and 128.80 (2C), 128.75 and 128.69 (1C), 128.20 and 126.36 (2C), 128.07 and 127.64 (1C), 127.83 and 127.80 (td, $J_{C\text{-}F,C\text{-}P}$ = 5.9, 2.1 Hz, 1C), 125.60 and 125.58 (td, $J_{C\text{-}F,C\text{-}P}$ = 7.2, 2.5 Hz, 1C), 122.58 and 122.54 (1C), 118.24 and 118.22 (td, $J_{C\text{-}F,C\text{-}P}$ = 263.0, 218.0 Hz, 1C), 64.99 and 64.93 (2C), 50.13 and 48.78 (1C), 42.02 and 41.28 (1C) 41.54 and 40.99 (1C), 27.68 and 27.62 (1C), 16.49 and 16.45 (2C), 13.87 and 12.69 (1C). **^{19}F NMR** (471 MHz, $CDCl_3$, observed as a 1:1 mixture of *E:Z* amide isomers) δ (ppm): -108.32 (d, $J_{F\text{-}P}$ = 115.5 Hz, 1F), -108.43 (d, $J_{F\text{-}P}$ = 115.5 Hz, 1F). **^{31}P NMR** (203 MHz, $CDCl_3$) δ (ppm): 6.40 (t, $J_{P\text{-}F}$ = 116.4 Hz, 1P). **HRMS** (ESI): *m/z* calcd. for $C_{28}H_{32}F_2N_5O_5P$ $[M+H]^+$: 588.2188, found: 588.2170.

Ethyl 3-(3-(9-(2-(benzyl(ethyl)amino)-2-oxoethyl)-7-methyl-8-oxo-8,9-dihydro-7*H*-purin-2-yl)phenyl)oxetane-3-carboxylate (6h)

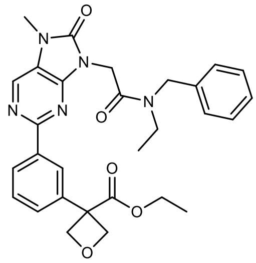

Prepared according to **General Procedure B**, using *N*-benzyl-*N*-ethyl-2-(7-methyl-8-oxo-2-phenyl-7,8-dihydro-9*H*-purin-9-yl)acetamide (*Emapunil*, 80.3 mg, 0.20 mmol) as LSF substrate and ethyl 3-bromooxetane-3-carboxylate (125.4 mg, 0.60 mmol) as alkyl bromide. The crude reaction mixture was analysed by LC-MS using an acidic mobile phase at pH 3 and the UV chromatograms are shown below:

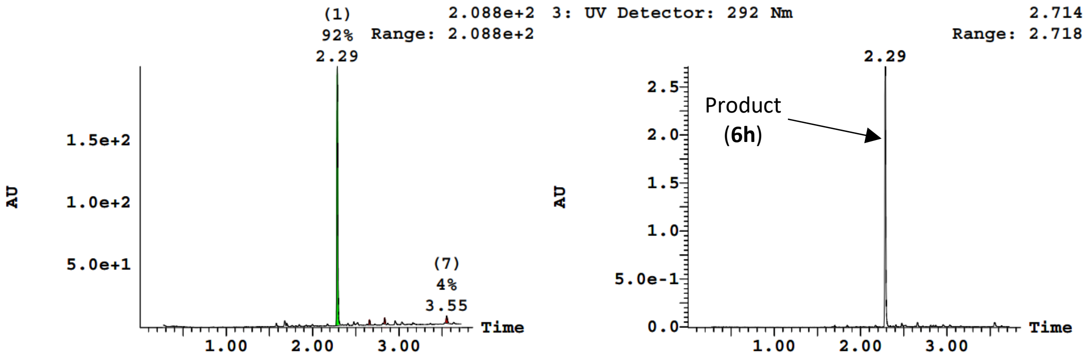

Supplementary Fig. 59. UV chromatograms (TIC & 254 nm) of the crude reaction mixture forming product **6h**.

Purification by preparative reverse phase HPLC (25-85% MeCN in acidic HCO_2H buffer, 254 nm). **6h** was isolated as a white solid (96.2 mg, 91%). **^{1}H NMR** (500 MHz, $CDCl_3$, observed as a 1:1 mixture of *E:Z* amide isomers) δ (ppm): 8.34 – 8.28 (m, 2H), 8.26 (s, 0.5H), 8.23 (s, 0.5H), 7.52 – 7.42 (m, 2H), 7.40 – 7.30 (m, 2H), 7.29 – 7.22 (m, 3H), 5.31 (d, *J* = 6.2 Hz, 1H), 5.28 (d, *J* = 6.2 Hz, 1H), 5.12 (d, *J* = 6.2 Hz, 1H), 5.09 (d, *J* = 6.2 Hz, 1H), 4.92 (s, 1H), 4.85 (s, 1H), 4.69 (s, 1H), 4.63 (s, 1H), 4.26 – 4.17 (m, 2H), 3.53 (s, 1.5H), 3.50 (s, 1.5H), 3.46 (q, *J* = 7.3 Hz, 2H), 1.37 (t, *J* = 7.2 Hz, 1.5H), 1.28 – 1.20 (m, 3H), 1.15 (t, *J* = 7.1 Hz, 1.5H). **^{13}C NMR** (126 MHz, $CDCl_3$, observed as a 1:1 mixture of *E:Z* amide isomers) δ (ppm): 173.36 and 173.35 (1C), 165.46 and 165.40 (1C), 157.40 (1C), 153.59 and 153.54 (1C), 150.05 and 150.03 (1C), 139.80 and 139.78 (1C), 138.48 and 138.43 (1C), 137.00 and 135.99 (1C), 131.90 and 131.83 (1C), 129.20 (1C), 128.87 and 128.79 (1C), 128.70 and 128.12 (2C), 127.97 and 127.58 (1C), 127.55 and 126.32 (2C), 127.09 and 127.03 (1C), 125.39 (1C), 122.36 and 122.31 (1C), 79.67 and 79.64 (2C), 61.72 and 61.69 (1C), 53.31 (1C), 50.02 and 48.77 (1C), 41.91 and 41.23 (1C), 41.45 and 40.87 (1C), 27.57 and 27.51 (1C), 14.10 (1C), 13.87 and 12.63 (1C). **HRMS** (ESI): *m/z* calcd. for $C_{29}H_{31}N_5O_5$ $[M+H]^+$: 530.2404, found: 530.2430.

Methyl 3-(3-(9-(2-(benzyl(ethyl)amino)-2-oxoethyl)-7-methyl-8-oxo-8,9-dihydro-7*H*-purin-2-yl)phenyl)tetrahydrofuran-3-carboxylate (6i)

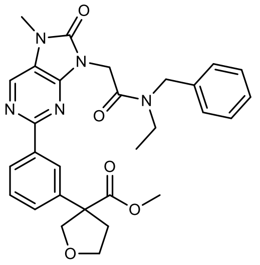

Prepared according to **General Procedure B**, using *N*-benzyl-*N*-ethyl-2-(7-methyl-8-oxo-2-phenyl-7,8-dihydro-9*H*-purin-9-yl)acetamide (*Emapunil*, 80.3 mg, 0.20 mmol) as LSF substrate and methyl 3-bromotetrahydrofuran-3-carboxylate (125.4 mg, 0.60 mmol) as alkyl bromide. The crude reaction mixture was analysed by LC-MS using an acidic mobile phase at pH 3 and the UV chromatograms are shown below:

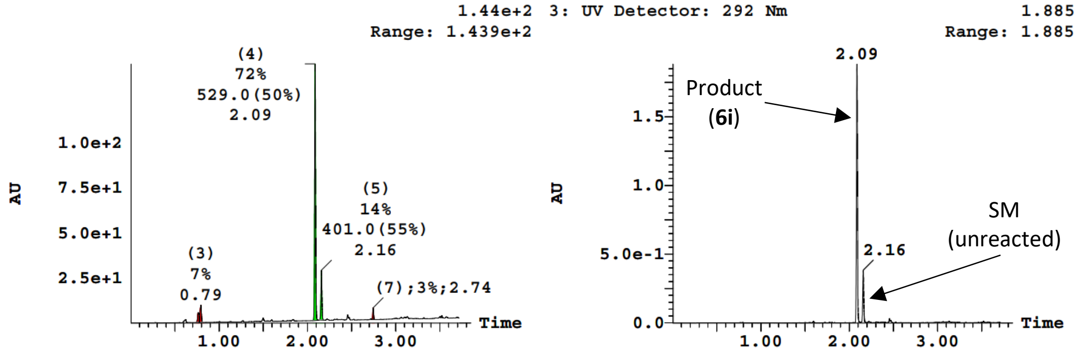

Supplementary Fig. 60. UV chromatograms (TIC & 254 nm) of the crude reaction mixture forming product **6i**.

Purification by preparative reverse phase HPLC (25-85% MeCN in acidic HCO_2H buffer, 254 nm). **6i** was isolated as a white solid (84.2 mg, 79%). **^{1}H NMR** (500 MHz, $CDCl_3$, observed as a 1:1 mixture of *E:Z* amide isomers) δ (ppm): 8.33 – 8.26 (m, 2H), 8.26 (s, 0.5H), 8.23 (s, 0.5H), 7.48 – 7.40 (m, 2H), 7.40 – 7.33 (m, 2H), 7.27 – 7.22 (m, 3H), 4.92 (s, 1H), 4.84 (s, 1H), 4.78 (dd, *J* = 8.5, 0.9 Hz, 0.5H), 4.75 (dd, *J* = 8.5, 0.9 Hz, 0.5H), 4.68 (s, 1H), 4.66 – 4.58 (m, 1H), 4.06 – 3.95 (m, 3H), 3.69 (s, 1.5H), 3.67 (s, 1.5H), 3.52 (s, 1.5H), 3.49 (s, 1.5H), 3.48 – 3.42 (m, 2H), 3.14 – 3.02 (m, 1H), 2.39 – 2.27 (m, 1H), 1.37 (t, *J* = 7.2 Hz, 1.5H), 1.14 (t, *J* = 7.1 Hz, 1.5H). **^{13}C NMR** (126 MHz, $CDCl_3$, observed as a 1:1 mixture of *E:Z* amide isomers) δ (ppm): 174.33 and 174.30 (1C), 165.49 and 165.43 (1C), 157.59 and 157.57 (1C), 153.62 and 153.56 (1C), 150.05 and 150.03 (1C), 139.94 and 139.93 (1C), 138.35 and 138.30 (1C), 137.03 and 136.02 (1C), 131.93 and 131.86 (1C), 129.22 (1C), 128.78 and 128.70 (1C), 128.68 and 128.12 (2C), 128.46 and 127.98 (1C), 127.55 and 126.35 (2C), 127.02 and 126.98 (1C), 126.16 (1C), 122.32 and 122.26 (1C), 75.14 and 75.09 (1C), 67.73 and 67.71 (1C), 59.10 (1C), 52.87 and 52.85 (1C), 50.05 and 48.81 (1C), 41.93 and 41.28 (1C), 41.46 and 40.88 (1C), 35.71 and 35.66 (1C), 27.59 and 27.52 (1C), 13.94 and 12.64 (1C). **HRMS** (ESI): *m/z* calcd. for $C_{29}H_{31}N_5O_5$ $[M+H]^+$: 530.2404, found: 530.2397.

3-Benzyl 1-(*tert*-butyl) 3-(3-(9-(2-(benzyl(ethyl)amino)-2-oxoethyl)-7-methyl-8-oxo-8,9-dihydro-7*H*-purin-2-yl)phenyl)azetidine-1,3-dicarboxylate (6j)

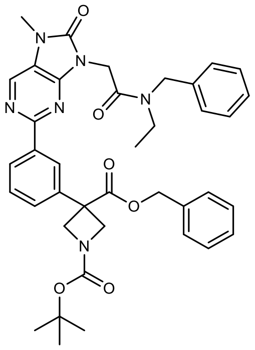

Prepared according to **General Procedure B**, using *N*-benzyl-*N*-ethyl-2-(7-methyl-8-oxo-2-phenyl-7,8-dihydro-9*H*-purin-9-yl)acetamide (*Emapunil*, 80.3 mg, 0.20 mmol) as LSF substrate and 3-benzyl 1-(*tert*-butyl) 3-bromoazetidine-1,3-dicarboxylate (222.1 mg, 0.60 mmol) as alkyl bromide. The crude reaction mixture was analysed by LC-MS using an acidic mobile phase at pH 3 and the UV chromatograms are shown below:

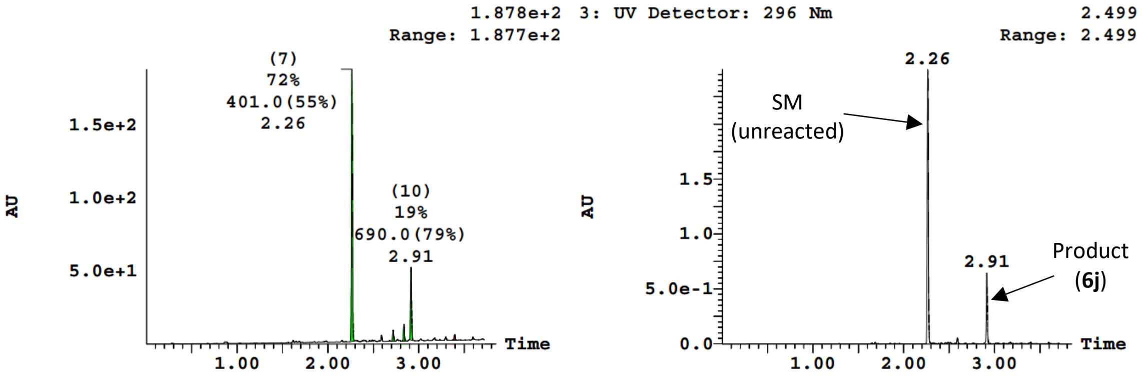

Supplementary Fig. 61. UV chromatograms (TIC & 254 nm) of the crude reaction mixture forming product **6j**.

Purification by preparative reverse phase HPLC (35-100% MeCN in acidic HCO_2H buffer, 254 nm). **6j** was isolated as a white solid (33.3 mg, 24%). **^{1}H NMR** (500 MHz, $CDCl_3$, observed as a 1:1 mixture of *E:Z* amide isomers) δ (ppm): 8.36 – 8.29 (m, 2H), 8.27 (s, 0.5H), 8.23 (s, 0.5H), 7.50 – 7.41 (m, 2H), 7.39 – 7.32 (m, 2H), 7.30 – 7.23 (m, 6H), 7.22 – 7.18 (m, 2H), 5.15 (s, 1H), 5.13 (s, 1H), 4.90 (s, 1H), 4.83 (s, 1H), 4.68 (s, 1H), 4.64 (br d, *J* = 8.7 Hz, 2H), 4.61 (s, 1H), 4.40 (br s, 2H), 3.54 (s, 1.5H), 3.50 (s, 1.5H), 3.50 – 3.39 (m, 2H), 1.43 (s, 4.5H), 1.43 (s, 4.5H), 1.35 (t, *J* = 7.1 Hz, 1.5H), 1.14 (t, *J* = 7.1 Hz, 1.5H). **^{13}C NMR** (126 MHz, $CDCl_3$, observed as a 1:1 mixture of *E:Z* amide isomers) δ (ppm): 173.06 and 173.04

(1C), 165.54 and 165.44 (1C), 157.41 (1C), 156.19 and 156.16 (1C), 153.72 and 153.65 (1C), 150.22 (1C), 139.88 and 139.83 (1C), 138.40 and 138.34 (1C), 137.05 and 136.04 (1C), 135.48 (1C), 131.82 and 131.74 (1C), 129.32 (1C), 129.00 and 128.91 (1C), 128.80 and 128.38 (2C), 128.66 (2C), 128.22 (1C), 128.13 and 126.39 (2C), 128.09 and 127.65 (1C), 127.95 (2C), 127.34 and 127.27 (1C), 125.89 (1C), 122.50 and 122.44 (1C), 80.08 (1C), 67.46 (1C), 59.10 and 57.64 (br, 2C), 50.17 and 48.86 (1C), 47.52 (1C), 42.08 and 41.32 (1C), 41.59 and 41.00 (1C), 28.46 (3C), 27.73 and 27.66 (1C), 13.97 and 12.75 (1C). **HRMS** (ESI): *m/z* calcd. for $C_{39}H_{42}N_6O_6$ $[M+H]^+$: 691.3244, found: 691.3215.

4-Benzyl 1-(*tert*-butyl) 4-(3-(9-(2-(benzyl(ethyl)amino)-2-oxoethyl)-7-methyl-8-oxo-8,9-dihydro-7*H*-purin-2-yl)phenyl)piperidine-1,4-dicarboxylate (6k)

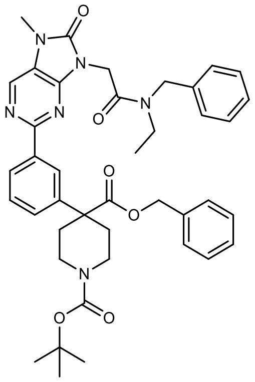

Prepared according to **General Procedure B**, using *N*-benzyl-*N*-ethyl-2-(7-methyl-8-oxo-2-phenyl-7,8-dihydro-9*H*-purin-9-yl)acetamide (*Emapunil*, 80.3 mg, 0.20 mmol) as LSF substrate and 4-benzyl 1-(*tert*-butyl) 4-bromopiperidine-1,4-dicarboxylate (239.0 mg, 0.60 mmol) as alkyl bromide. The crude reaction mixture was analysed by LC-MS using an acidic mobile phase at pH 3 and the UV chromatograms are shown below:

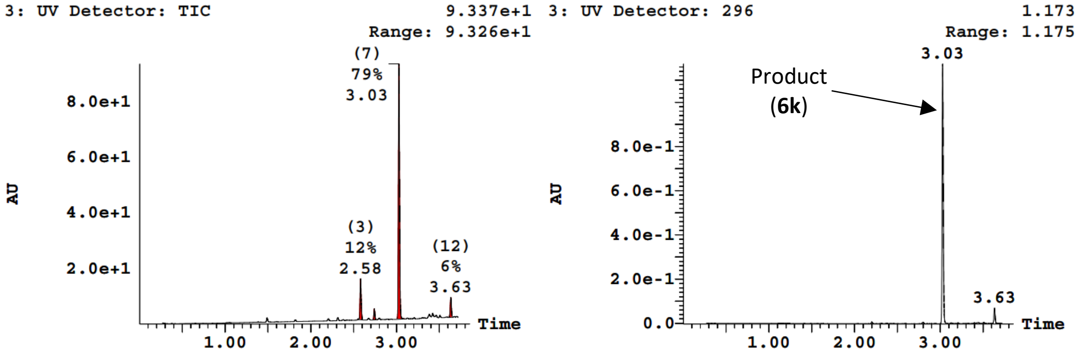

Supplementary Fig. 62. UV chromatograms (TIC & 254 nm) of the crude reaction mixture forming product **6k**.

Purification by preparative reverse phase HPLC (40-100% MeCN in acidic HCO_2H buffer, 254 nm). **6k** was isolated as a white solid (129.6 mg, 90%). **^{1}H NMR** (500 MHz, $CDCl_3$, observed as a 1:1 mixture of *E:Z* amide isomers) δ (ppm): 8.43 (t, *J* = 1.7 Hz, 0.5H), 8.41 (t, *J* = 1.7 Hz, 0.5H), 8.29 – 8.24 (m, 1.5H), 8.23 (s, 0.5H), 7.46 – 7.34 (m, 4H), 7.29 – 7.21 (m, 6H), 7.21 – 7.15 (m, 2H), 5.12 (s, 1H), 5.10 (s, 1H), 4.90 (s, 1H), 4.83 (s, 1H), 4.68 (s, 1H), 4.61 (s, 1H), 3.98 (br s, 2H), 3.53 (s, 1.5H), 3.49 (s, 1.5H), 3.51 – 3.39 (m, 2H), 3.03 (br s, 2H), 2.62 (t, *J* = 13.8 Hz, 2H), 1.95 (br s, 2H), 1.45 (s, 9H), 1.35 (t, *J* = 7.1 Hz, 1.5H), 1.14 (t, *J* = 7.1 Hz, 1.5H). **^{13}C NMR** (126 MHz, $CDCl_3$, observed as a 1:1 mixture of *E:Z* amide isomers) δ (ppm): 173.81 and 173.77 (1C), 165.43 and 165.36 (1C), 157.63 (1C), 154.77 and 154.74 (1C), 153.58 and 153.51 (1C), 150.03 and 150.02 (1C), 142.28 and 142.25 (1C), 138.19 and 138.11 (1C), 136.99 and 135.97 (1C), 135.68 (1C), 131.75 and 131.67 (1C), 129.17 (1C), 128.79 and 128.69 (1C), 128.66 and 128.15 (2C), 128.48 (2C), 128.07 (1C), 127.94 (2C), 127.92 and 127.54 (1C), 127.51 and 126.30 (2C), 126.83 and 126.76 (1C), 125.23 (1C), 122.25 and 122.19 (1C), 79.54 (1C), 66.82 and 66.81 (1C), 50.02 and 48.77 (1C), 49.58 and 49.57 (1C), 41.89 and 41.24 (1C), 41.68 and 41.00 (br, 2C), 41.42 and 40.86 (1C), 33.89 and 33.46 (br, 2C), 28.46 (3C), 27.56 and 27.49 (1C), 13.87 and 12.62 (1C). **HRMS** (ESI): *m/z* calcd. for $C_{41}H_{46}N_6O_6$ $[M+H]^+$: 719.3557, found: 719.3540.

General procedure for the saponification of carboxylate esters (5e', 6a'-6f', 6h'-6k')

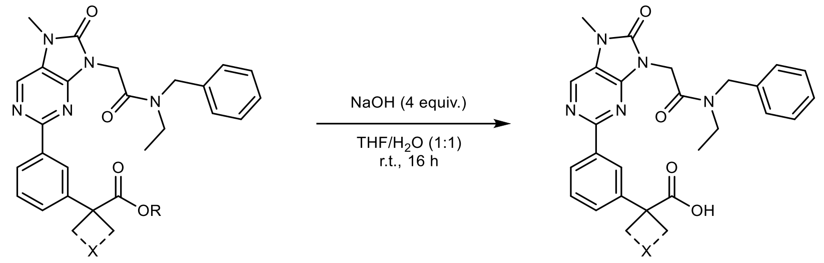

5e, **6a-6f**, **6h-6k** → **5e'**, **6a'-6f'**, **6h'-6k'**

In a screw-cap vial, a 0.1 N solution of sodium hydroxide (NaOH, 0.12-0.52 mmol, 4 equiv.) was added to a solution of the appropriate carboxylate **5e**, **6a-6f**, **6h-6k** (0.03-0.13 mmol, 1.0 equiv.) in tetrahydrofuran (THF, total concentration of 0.0125 M). The mixture was vigorously stirred at room temperature for 16 hours (progress of the reaction was monitored by LC-MS) and THF was removed under reduced pressure. The residue was diluted with water (5 mL) and the aqueous layer was washed with CH_2Cl_2 (2 x 5 mL), then acidified with 0.5 N HCl (pH = 1-2). The reaction mixture was extracted with CH_2Cl_2 (4 x 5 mL) and the combined organic layer was dried over Isolute® phase separator and concentrated under reduced pressure, affording the desired carboxylic acids **5e'**, **6a'-6f'**, **6h'-6k'** (almost quantitative yields) as white solids. These were pure enough (96-100% LC-MS purity) to be used for subsequent drug discovery assays.

Procedure for the dealkylation of phosphonate ester (6g')

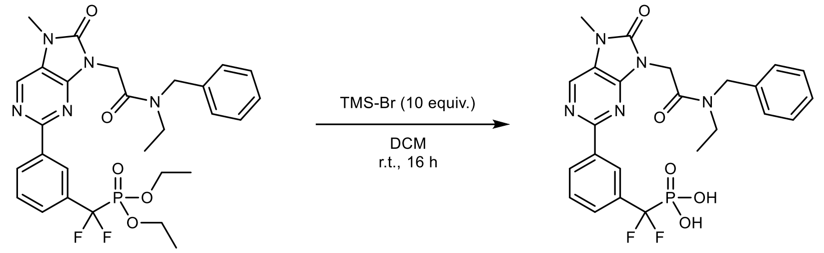

6g → **6g'**

In a screw-cap vial, bromotrimethylsilane (TMS–Br, 52.8 µL, 0.40 mmol, 10 equiv.) was added to a solution of diethyl phosphonate **6g** (23.5 mg, 0.04 mmol, 1.0 equiv.) in dry CH_2Cl_2 (DCM, 2 mL, 0.02 M). The reaction mixture was stirred at room temperature for 16 hours (progress of the reaction was monitored by LC-MS) and the volatiles were removed under reduced pressure. The residue was dissolved in a 1:1 mixture of H_2O and MeCN (5 mL) and the solution was freeze dried, affording the desired phosphonic acid **6g'** (20.9 mg, 99%) as a white solid. This was pure enough (99% LC-MS purity) to be used for subsequent drug discovery assays.

4.4. Synthetic handle diversification (7, 8a-8d)

Ethyl 3-(3-(7-chloro-1-methyl-2-oxo-2,3-dihydro-1*H*-benzo[*e*][1,4]diazepin-5-yl)phenyl)oxetane-3-carboxylate (7)

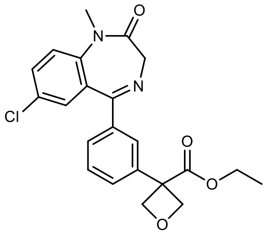

Prepared according to **General Procedure B** on a 12.5 times larger scale with reduced stoichiometry of the coupling partner, using 7-chloro-1-methyl-5-phenyl-1,3-dihydro-2*H*-benzo[*e*][1,4]diazepin-2-one (*Diazepam*, 711.9 mg, 2.50 mmol) as LSF substrate and ethyl 3-bromooxetane-3-carboxylate (1045.2 mg, 5.00 mmol, 2.0 equiv.) as alkyl bromide. The crude reaction mixture was analysed by LC-MS using an acidic mobile phase at pH 3 and the UV chromatograms are shown below:

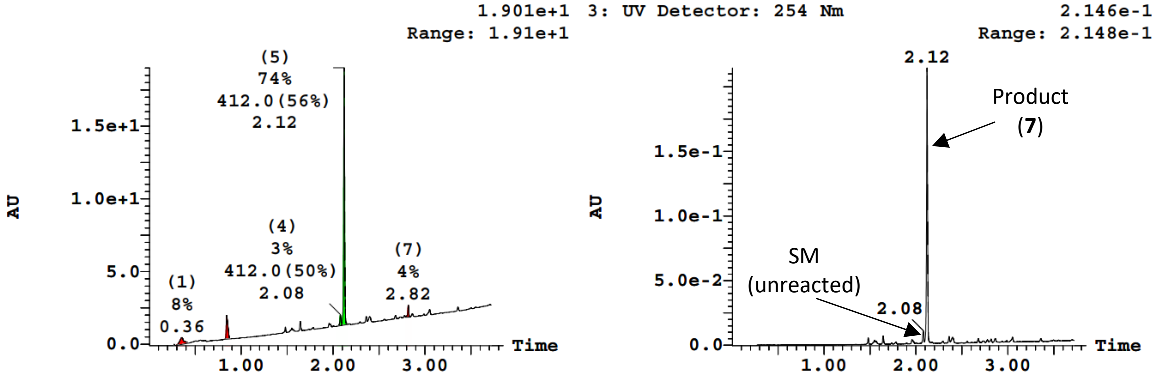

Supplementary Fig. 63. UV chromatograms (TIC & 254 nm) of the crude reaction mixture forming product **7**.

Purification by preparative reverse phase HPLC (30-90% MeCN in acidic HCO_2H buffer, 254 nm). **7** was isolated as a white solid (807.2 mg, 78%). **^{1}H NMR** (500 MHz, $CDCl_3$) δ (ppm): 7.54 (t, *J* = 1.8 Hz, 1H), 7.53 – 7.48 (m, 2H), 7.43 (t, *J* = 7.7 Hz, 1H), 7.38 (dt, *J* = 7.8, 1.5 Hz, 1H), 7.31 (d, *J* = 8.8 Hz, 1H), 7.26 (d, J = 2.4 Hz, 1H), 5.26 (d, *J* = 6.2 Hz, 1H), 5.24 (d, *J* = 6.2 Hz, 1H), 5.01 (d, *J* = 6.2 Hz, 2H), 4.84 (d, *J* = 10.8 Hz, 1H), 4.21 (qd, *J* = 7.1, 3.1 Hz, 2H), 3.76 (d, *J* = 10.8 Hz, 1H), 3.40 (s, 3H), 1.24 (t, *J* = 7.1 Hz, 3H). **^{13}C NMR** (126 MHz, $CDCl_3$) δ (ppm): 172.9, 169.7, 168.3, 142.6, 140.0, 138.6, 131.7, 129.7 (2C), 129.2, 129.0, 128.8, 128.4, 127.0, 122.8, 79.52, 79.49, 61.8, 56.9, 53.1, 34.9, 14.0. **HRMS** (ESI): *m/z* calcd. for $C_{22}H_{21}ClN_2O_4$ $[M+H]^+$: 413.1268, found: 413.1280.

3-(3-(7-Chloro-1-methyl-2-oxo-2,3-dihydro-1*H*-benzo[*e*][1,4]diazepin-5-yl)phenyl)oxetane-3-carboxylic acid (8a)

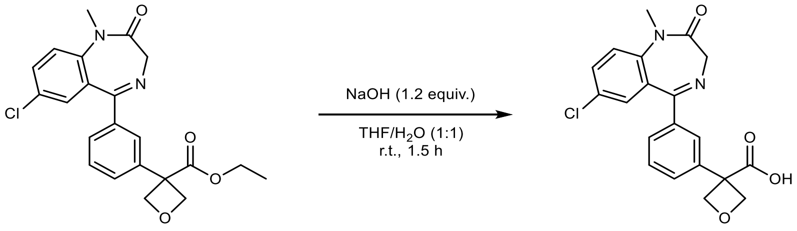

7 8a

In a round bottom flask, a solution of sodium hydroxide (NaOH, 72.0 mg, 1.80 mmol, 1.2 equiv.) in water (H_2O, 15 mL, 0.12 N) was added to a solution of ethyl ester **7** (619.3 mg, 1.50 mmol, 1.0 equiv.) in tetrahydrofuran (THF, 15 mL, total concentration of 0.05 M). The mixture was vigorously stirred at room temperature for 1.5 hour. The crude reaction mixture was analysed by LC-MS using an acidic mobile phase at pH 3 and the UV chromatograms are shown below:

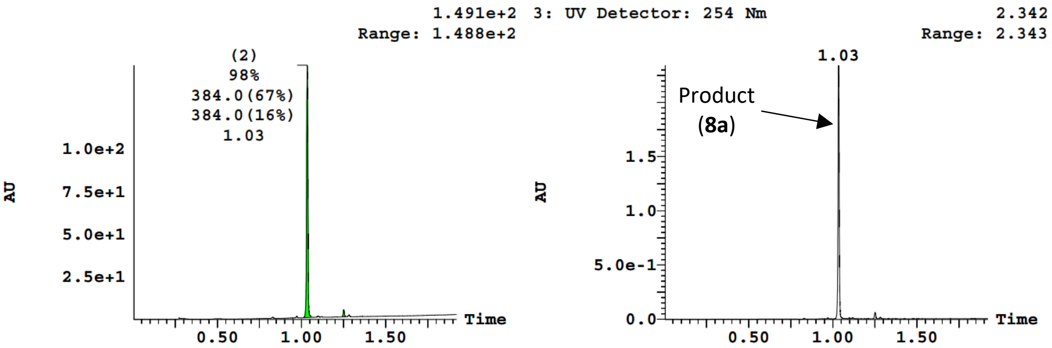

Supplementary Fig. 64. UV chromatograms (TIC & 254 nm) of the crude reaction mixture forming product **8a**.

THF was removed under reduced pressure and the residue was diluted with water (10 mL). The aqueous layer was washed with EtOAc (2 x 10 mL), then acidified with 0.5 N HCl (pH = 1-2). The reaction mixture was extracted with EtOAc (4 x 50 mL) and the combined organic layer was washed with brine (20 mL), dried over anhydrous $MgSO_4$ and concentrated under reduced pressure, yielding the desired acid **8a** (567.4 mg, 98 %) as a white solid. This was pure enough to be used for subsequent transformations without further purification.

^{1}H NMR (500 MHz, $CDCl_3$) δ (ppm): 7.73 (t, *J* = 1.9 Hz, 1H), 7.55 (dd, *J* = 8.8, 2.5 Hz, 1H), 7.41 (dt, *J* = 7.8, 1.7 Hz, 1H), 7.37 (t, *J* = 7.6 Hz, 1H), 7.31 (d, *J* = 8.8 Hz, 1H), 7.27 (d, *J* = 2.5 Hz, 1H), 7.23 (dt, *J* = 7.4, 1.6 Hz, 1H), 5.31 (d, *J* = 6.3 Hz, 2H), 5.08 (d, *J* = 6.3 Hz, 1H), 5.02 (d, *J* = 6.2 Hz, 1H), 4.90 (d, *J* = 11.1 Hz, 1H), 3.75 (d, *J* = 11.2 Hz, 1H), 3.38 (s, 3H). **^{13}C NMR** (126 MHz, $CDCl_3$) δ (ppm): 175.4, 170.2, 169.7, 142.7, 140.8, 137.7, 132.3, 130.4, 129.7, 129.6 (2C), 129.4, 128.8, 127.3, 122.8, 79.8, 79.6, 55.9, 53.0, 35.2. **HRMS** (ESI): *m/z* calcd. for $C_{20}H_{17}ClN_2O_4$ $[M+H]^+$: 385.0955, found: 385.0957.

N-(Bicyclo[1.1.1]pentan-1-yl)-3-(3-(7-chloro-1-methyl-2-oxo-2,3-dihydro-1*H*-benzo[*e*][1,4]diazepin-5-yl)phenyl)oxetane-3-carboxamide (8b)

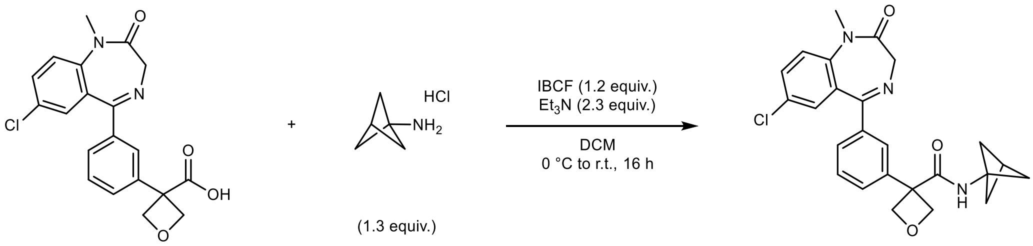

8a **8b**

In a microwave vial, carboxylic acid **8a** (38.5 mg, 0.10 mmol, 1.0 equiv.) and triethylamine (Et_3N, 32.1 µL, 0.23 mmol, 2.3 equiv.) were dissolved in dry CH_2Cl_2 (DCM, 2 mL, 0.05 M) under a N_2 atmosphere. The solution was cooled down to 0 °C and isobutyl chloroformate (IBCF, 15.6 µL, 0.12 mmol, 1.2 equiv.) was added dropwise to the mixture. After stirring for 1 hour, bicyclo[1.1.1]pentan-1-amine hydrochloride (15.6 mg, 0.13 mmol, 1.3 equiv.) was added and the reaction mixture was allowed to stir at room temperature for 16 hours. The crude reaction mixture was analysed by LC-MS using an acidic mobile phase at pH 3 and the UV chromatograms are shown below:

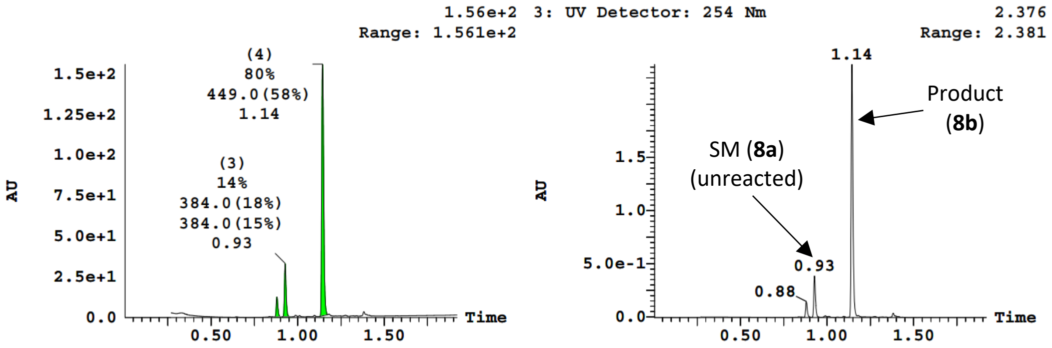

Supplementary Fig. 65. UV chromatograms (TIC & 254 nm) of the crude reaction mixture forming product **8b**.

The reaction mixture was diluted with CH_2Cl_2 (10 mL), washed with H_2O (2 x 5 mL) and saturated aqueous $NaHCO_3$ (2 x 5 mL). The organic layer was dried over $MgSO_4$ and the volatiles were removed under reduced pressure. The residue was purified by automated flash column chromatography (0-80% EtOAc in *n*-heptane, 10 g SiO_2), yielding the desired amide **8b** (36.7 mg, 82 %) as a white solid.
^{1}H NMR (500 MHz, $CDCl_3$) δ (ppm): 7.55 (t, *J* = 1.9 Hz, 1H), 7.54 – 7.49 (m, 2H), 7.46 (t, *J* = 7.7 Hz, 1H), 7.34 (ddd, *J* = 7.6, 2.0, 1.3 Hz, 1H), 7.32 (d, *J* = 8.8 Hz, 1H), 7.27 (d, *J* = 2.5 Hz, 1H), 5.76 (br s, 1H), 5.28 (d, *J* = 5.8 Hz, 1H), 5.19 (d, *J* = 6.0 Hz, 1H), 4.99 (d, *J* = 6.0 Hz, 1H), 4.87 (d, *J* = 5.8 Hz, 1H), 4.83 (d, *J* = 10.7 Hz, 1H), 3.77 (d, *J* = 10.8 Hz, 1H), 3.39 (s, 3H), 2.41 (s, 1H), 2.03 (s, 6H). **^{13}C NMR** (126 MHz, $CDCl_3$) δ (ppm): 172.6, 169.8, 168.5, 142.8, 140.9, 139.1, 131.9, 129.8, 129.7, 129.6, 129.5, 129.3, 128.9, 127.2, 122.9, 79.8, 79.4, 57.1, 53.7, 52.8 (3C), 49.0, 35.1, 24.8. **HRMS** (ESI): *m/z* calcd. for $C_{25}H_{24}ClN_3O_3$ $[M+H]^+$: 450.1584, found: 450.1572.

tert-Butyl (3-(3-(7-chloro-1-methyl-2-oxo-2,3-dihydro-1*H*-benzo[*e*][1,4]diazepin-5-yl)phenyl)oxetan-3-yl)carbamate (8c)

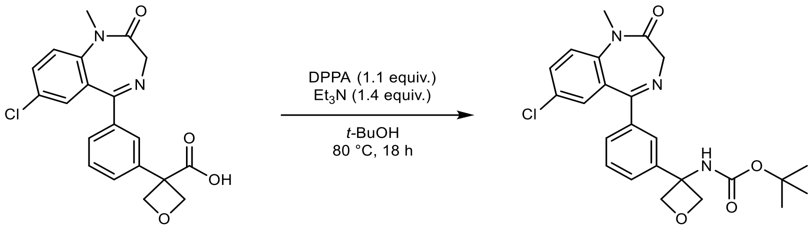

In a microwave vial, triethylamine (Et_3N, 19.5 µL, 0.14 mmol, 1.4 equiv.) and diphenylphosphoryl azide (DPPA, 23.7 µL, 0.11 mmol, 1.1 equiv.) were added to a solution of carboxylic acid **8a** (38.5 mg, 0.10 mmol, 1.0 equiv.) in dry *tert*-butyl alcohol (*t*-BuOH, 0.25 mL, 0.4 M) at room temperature under a N_2 atmosphere. The reaction mixture was stirred at 80 °C for 18 hours. The crude reaction mixture was analysed by LC-MS using an acidic mobile phase at pH 3 and the UV chromatograms are shown below:

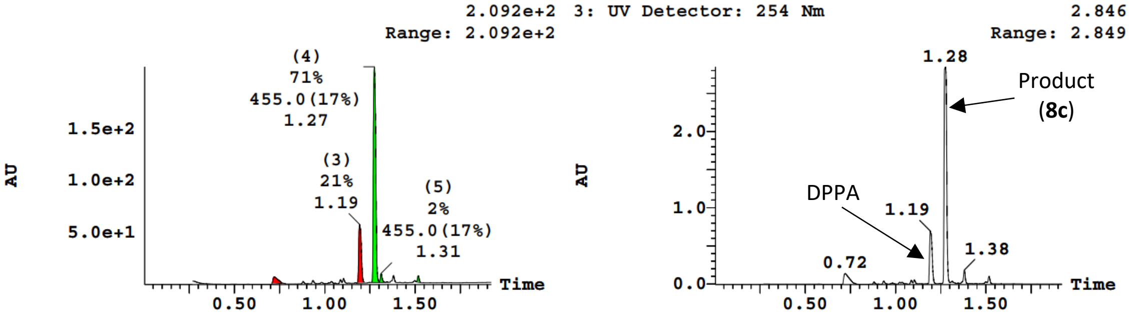

Supplementary Fig. 66. UV chromatograms (TIC & 254 nm) of the crude reaction mixture forming product **8c**.

The volatiles were removed under reduced pressure and the residue was dissolved in CH_2Cl_2 (10 mL). The reaction mixture was washed with saturated aqueous $NaHCO_3$ (2 x 5 mL) and brine (2 x 5 mL). The organic layer was dried over MgSO4 and concentrated under reduced pressure. The residue was purified by automated flash column chromatography (0-70% EtOAc in *n*-heptane, 10g SiO_2), yielding the desired carbamate **8c** (32.0 mg, 70 %) as a white solid.
^{1}H NMR (500 MHz, $CDCl_3$) δ (ppm): 7.85 (dt, *J* = 2.1, 1.1 Hz, 1H), 7.73 – 7.66 (m, 1H), 7.52 (dd, *J* = 8.8, 2.5 Hz, 1H), 7.49 – 7.40 (m, 2H), 7.34 – 7.26 (m, 2H), 5.38 (br s, 1H), 5.01 (br s, 1H), 4.97 (br s, 1H), 4.89 (d, *J* = 6.7 Hz, 1H), 4.87 – 4.73 (m, 2H), 3.77 (d, *J* = 10.8 Hz, 1H), 3.39 (s, 3H), 1.41 (br s, 9H). **^{13}C NMR** (126 MHz, $CDCl_3$) δ (ppm): 170.0, 168.8, 154.5, 142.9 (br), 142.7, 138.6, 131.7, 130.01, 129.96, 129.5, 129.1, 128.8, 127.3 (br), 125.8, 122.7, 83.2 (br), 83.0, 80.4 (br), 58.4, 57.0, 35.0, 28.4 (br, 3C). **HRMS** (ESI): *m/z* calcd. for $C_{24}H_{26}ClN_3O_4$ $[M+H]^+$: 456.1690, found: 456.1669.

1,3-Dioxoisoindolin-2-yl 3-(3-(7-chloro-1-methyl-2-oxo-2,3-dihydro-1*H*-benzo[*e*][1,4]diazepin-5-yl)phenyl)oxetane-3-carboxylate (8d)

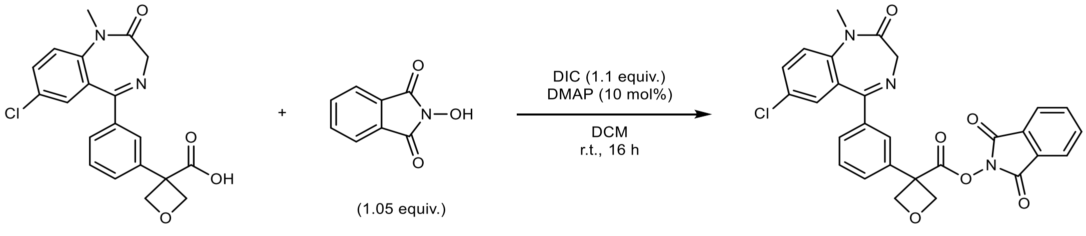

In a round bottom flask, carboxylic acid **8a** (288.6 mg, 0.75 mmol, 1.0 equiv.), *N*-hydroxyphthalimide (128.5 mg, 0.79 mmol, 1.05 equiv.) and 4-dimethylaminopyridine (DMAP, 9.2 mg, 0.08 mmol, 10 mol%) were dissolved in dry CH_2Cl_2 (DCM, 15 mL, 0.05 M) under a N_2 atmosphere. Then, *N,N'*-diisopropylcarbodiimide (DIC, 807.1 µL, 0.83 mmol, 1.1 equiv.) was added dropwise to the solution at room temperature and the reaction mixture was vigorously stirred for 16 hours. The crude reaction mixture was analysed by LC-MS using an acidic mobile phase at pH 3 and the UV chromatograms are shown below:

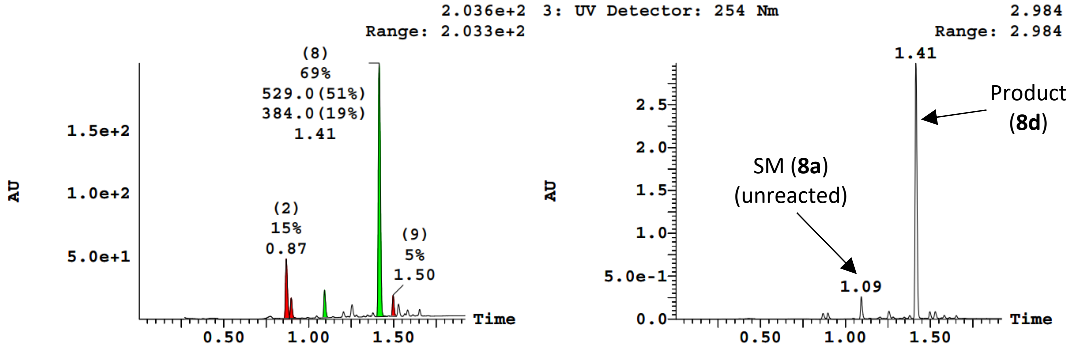

Supplementary Fig. 67. UV chromatograms (TIC & 254 nm) of the crude reaction mixture forming product **8d**.

The precipitate formed was removed by filtration and the volatiles were removed under reduced pressure. The residue was purified by automated flash column chromatography (0-70% EtOAc in *n*-heptane, 25 g SiO_2), yielding the desired redox active ester **8d** (257.1 mg, 65 %) as a white solid.
^{1}H NMR (500 MHz, $CDCl_3$) δ (ppm): 7.87 (dd, *J* = 5.5, 3.1 Hz, 2H), 7.80 (dd, *J* = 5.5, 3.1 Hz, 2H), 7.77 (t, *J* = 1.8 Hz, 1H), 7.64 (dt, *J* = 7.6, 1.5 Hz, 1H), 7.59 (ddd, *J* = 7.8, 2.0, 1.3 Hz, 1H), 7.53 (t, *J* = 7.7 Hz, 1H), 7.50 (dd, *J* = 8.8, 2.5 Hz, 1H), 7.34 – 7.30 (m, 2H), 5.49 – 5.44 (m, 2H), 5.12 (d, *J* = 3.8 Hz, 1H), 5.11 (d, *J* = 3.8 Hz, 1H), 4.87 (d, *J* = 10.8 Hz, 1H), 3.79 (d, *J* = 10.8 Hz, 1H), 3.42 (s, 3H). **^{13}C NMR** (126 MHz, $CDCl_3$) δ (ppm): 169.9, 169.5, 168.4, 161.6 (2C), 142.8, 139.1, 137.8, 135.0 (2C), 131.8, 129.9, 129.78, 129.76, 129.5, 129.3, 128.9 (2C), 128.6, 127.5, 124.1 (2C), 122.8, 79.32, 79.26, 57.1, 51.8, 35.1. **HRMS** (ESI): *m/z* calcd. for $C_{28}H_{20}ClN_3O_6$ $[M+H]^+$: 530.1119, found: 530.1125.

5. Pharmaceutically relevant properties modulation

Product analogues **5e**, **6a-6k** obtained after late-stage *meta*-C–H alkylation of the pharmaceutically active compound *Emapunil* (**4e**) were subjected to a range of fundamental *in vitro* drug discovery assays. To prevent hydrolysis of ester groups present on these analogues during biological testing (in order to compare the clearance and metabolic stability between these drug derivatives), their corresponding free acids **5e'**, **6a'-6k'** were synthesised (see **page S54**) and subjected to similar assays.

The experimental methods used for these physicochemical & drug metabolism and pharmacokinetics (DMPK) assays are described below[11].

Solubility
This method measures the thermodynamic solubility of compounds based on a shake-flask approach[12]. The nature of the solid state was unknown in these experiments and may be subject to variability. The compounds were dissolved in DMSO (30 µL, 10 mM) in glass vials, and were dried using a Genevac vacuum evaporator (Genevac Ltd, Ipswich, UK). When the samples were dry, 300 µL of phosphate buffer (0.1 M, pH 7.4) was added to the glass vials. The vials were put on an Edmund Bühler shaker (Edmund Bühler GmbH, Hechingen, Germany) at 20 °C for 18 hours. Samples were filtered through a Whatman GF/B 96-well filter and 20 µL of filtrated samples were transferred to separate wells in a plate containing 180 µL MeCN/H_2O (1:1). Standards were prepared by diluting the 10 mM compound solutions with MeCN/H_2O (1:1) to 200 µM. Three further dilution steps of 10 times were applied to both the samples and standards and they were all analysed by LC-MS/MS (Waters Xevo-TQS). Solubility was determined using the integrated peak areas of the samples in the linear MS response range.

Octanol/water partitioning *(LogD)*
Partitioning of compounds between 1-octanol and phosphate buffer (0.1 M, pH 7.4) at 20 °C were determined using a modified version of the shake-flask method described by Leo *et al*[13]. Compounds were dissolved in a 96-well plate, in 400 µL octanol, and 400 µL of buffer was added to each well. The plate was vigorously stirred for 5 min and then put on an Edmund Bühler shaker (Edmund Bühler GmbH, Hechingen, Germany) at 20 °C for 18 hours. Aliquots of 5 µL octanol were transferred and diluted with 495 µL MeCN/H_2O (1:1) and, to avoid contamination of the buffer, the rest of the octanol was removed before 150 µL of buffer samples were transferred. Octanol and buffer samples were diluted with MeCN/H_2O (1:1) in four steps of 10 times to yield octanol samples diluted 10^2 to 10^6 times and buffer samples diluted 1 to 10^4 times. LC-MS/MS (Waters Xevo-TQS) was used for analysis, and distribution coefficient at pH 7.4 (Log$D_{7.4}$) was calculated from the integrated peak areas of the samples in the linear MS response range. Log$D_{7.4}$ results were reported as median values.

Fraction unbound in human plasma *(Prot. Bind.)*
Equilibrium dialysis of compounds was used to assess the fraction unbound in human plasma. Stock solutions of compounds dissolved in DMSO were pooled into cassettes with up to 10 compounds in each cassette with a concentration of 1 mM. The pooled stock solutions were diluted in plasma to a compound concentration of 5 µM. Warfarin, propranolol and metoprolol were used as reference compounds in each run. The equilibrium dialysis device (RED, Thermo Fischer Scientific Inc., Rockford, USA) was used for dialysis against phosphate buffer (pH 7.4) at 37 °C for 18 hours. Calibration curves ranging from 0.001 to 7 µM were made using aliquots of the 7 µM spiked plasma pools that were transferred to a new plate followed by serial dilutions with blank plasma. Calibration curve samples and dialysis samples were precipitated with MeCN and after centrifugation, supernatants were removed and analysed using LC-MS/MS (Waters Xevo-TQS). Fraction unbound in the incubation was calculated as the concentration in the media buffer sample divided by the concentration in the plasma sample. The fraction unbound was reported as the median value.

Metabolic stability in human liver microsomes *(CL_{int} HLM)*

Human liver microsomes were defrosted on ice and diluted to 1 mg/mL of microsomal protein in phosphate buffer (0.1 M, pH 7.4). Compounds at 1 µM were incubated with the liver microsome suspensions and 1 mM NADPH at 37 °C in a 96-well plate. At 0.5, 5, 10, 15, 20 and 30 min, aliquots of 30 µL were transferred to a 96-well plate containing 120 µL MeCN. This plate was centrifuged for 20 min and supernatant was removed and diluted with H_2O (1:1) before analysis by LC-MS/MS (Waters Xevo-TQS). Peak areas were determined from extracted ion chromatograms, and the *in vitro* intrinsic clearance (*in vitro* CL_{int}, in µL/min/mg microsomal protein) of parent compound was calculated from the slope in the regression analysis of the natural logarithm of parent concentration *vs.* time curve. Human Liver Microsomes (InVitroCYP™ 150-Donor, Mixed Gender) were harvested and supplied by BioIVT (Product No. X008070, Lot No. QQY).

Metabolic stability in male Han Wistar rat hepatocytes *(CL_{int} Rat Hep.)*

Hepatocyte metabolic stability was determined in accordance with the method described by Jacobson *et al*[14]. Cryopreserved hepatocytes at a concentration of 10^6 viable cells/mL were used. After thawing, hepatocytes were incubated for 10 min to warm to 37 °C, and compounds dissolved in MeCN were added to give a final concentration of 1 µM. At 0.5, 5, 15, 30, 45, 60, 80, 100 and 120 min, the incubation system was mixed and 20 µL aliquots were transferred at each time point to wells in a separate plate filled with 80 µL MeCN to stop the reaction. The quenching plate was then vortexed followed by centrifugation, and supernatants were analysed by LC-MS/MS (Waters Xevo-TQS). Peak areas were determined from extracted ion chromatograms, and the *in vitro* intrinsic clearance (*in vitro* CL_{int}, in µL/min/10^6 cells) of parent compound was calculated from the slope in the regression analysis of the natural logarithm of parent concentration *vs.* time curve. Rat Hepatocytes (Cryosuspension, Male Wistar Hannover) were harvested and supplied by BioIVT (Product No. M00065, Lot No. DVO).

The modulation of pharmaceutically relevant properties on *meta*-C–H alkylation is shown below in **Supplementary Fig. 68**, with a selection of physicochemical and DMPK data including **solubility**, **Log*D***, **human plasma protein binding** (Prot. Bind.) and **intrinsic clearance** (CL_{int}) in **human liver microsomes** (HLM) or **rat hepatocytes** (Rat Hep.).

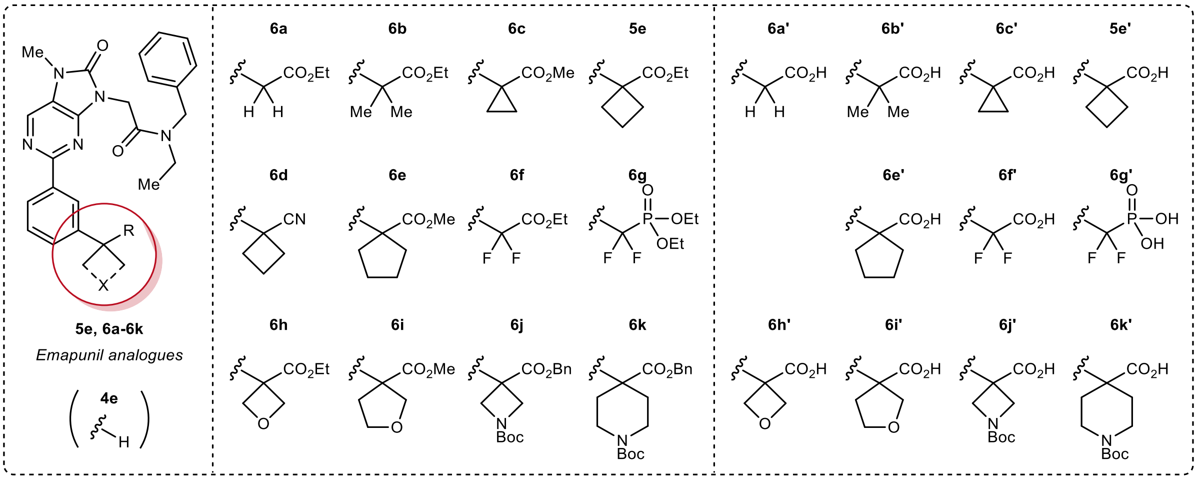

Compound	Solubility (µM)	Log*D* (octanol)	Prot. Bind. (% free)	CL_{int} HLM (µL/min/mg)	CL_{int} Rat Hep. (µL/min/10^6)
**4e**	6	3.2	0.8	218	164
**6a**	41	3.3	N/A	>300	>300
**6b**	8	4.2	0.8	>300	>300
**6c**	51	3.3	N/A	>300	>300
**5e**	17	4.4	0.4	>300	>300
**6d**	50	3.9	0.5	>300	>300
**6e**	36	4.3	0.4	>300	>300
**6f**	12	4.3	N/A	>300	>300
**6g**	46	4.4	2.0	>300	>300
**6h**	58	3.1	N/A	>300	>300
**6i**	104	2.9	7.8	>300	>300
**6j**	<2.4	>3.6	<0.1	>300	>300
**6k**	6	>3.2	<0.3	>300	157
**6a’**	924	-0.5	12.0	<3.0	10.5
**6b’**	>1000	0.5	3.0	11.1	10.3
**6c’**	965	0.3	6.6	<3.0	36.2
**5e’**	>1000	0.7	2.0	5.5	15.9
**6e’**	864	1.4	0.7	<3.0	51.0
**6f’**	852	-0.4	3.3	4.0	6.8
**6g’**	>1000	-0.8	3.7	<3.0	4.2
**6h’**	>1000	-1.0	14.0	4.6	19.3
**6i’**	>1000	-0.7	5.9	<3.0	12.9
**6j’**	910	0.4	0.8	7.6	17.2
**6k’**	806	1.4	<0.3	24.1	18.0

Supplementary Fig. 68. Selection of physicochemical & drug metabolism and pharmacokinetics (DMPK) data for the late-stage *meta*-C–H alkylated compounds **5e**, **6a-6k** and their corresponding free acids **5e’**, **6a’-6k’** including the parent compound *Emapunil* (**4e**) as reference. All values are measured (n = 1) and the assays were supplied by Discovery Sciences, AstraZeneca. **Solubility** and **Log*D*** at pH = 7.4. **Prot. Bind.** = human plasma protein binding, N/A = not available (low recovery, unstable in plasma). **CL_{int} HLM** = intrinsic clearance in human liver microsomes. **CL_{int} Rat Hep.** = intrinsic clearance in rat hepatocytes. Boc = *tert*-butyloxycarbonyl, Bn = benzyl.

Solubility

A general increase in solubility is observed in comparison with the parent molecule *Emapunil* (**4e**) due to the incorporation of ester groups. When comparing the reference compound bearing a methylene group (**6a**) with other ester analogues, a decrease in solubility is observed for compounds with higher number of carbons (dimethyl **6b**, cyclobutyl **5e** and cyclopentyl **6e**) or with the introduction of fluorine atoms (difluoro **6f**). An exception is noticed for cyclopropyl compound (**6c**) showing an increase in solubility. A higher solubility is observed by replacing the carboxylate group with cyano (**5e** *vs.* **6d**) and phosphonate (**6f** *vs.* **6g**) derivatives. A significant increase in solubility is observed with the installation of 4- and 5-membered heterocycles bearing an oxygen atom in comparison with their carbocyclic ring equivalents (oxetane **6h** *vs.* cyclobutyl **5e**, THF **6i** *vs.* cyclopentyl **6e**). However, a decrease in solubility is noticed for nitrogen-containing heterocycles (azetidine **6j** and piperidine **6k**), likely due to the low solubility of Boc-protecting groups and benzyl esters. Not surprisingly, the solubility increases drastically for all the free acids (**5e'**, **6a'-6k'**) in comparison with the corresponding ester derivatives. Thus, *meta*-C–H alkylation could be considered as a potential strategy for modulating solubility, especially by increasing the solubility of poorly soluble compounds.

Log*D*

High Log*D* values are observed for the parent molecule *Emapunil* (**4e**) and all compounds bearing an ester group (**5e**, **6a-6k**), reflecting the lipophilic nature of this chemical series. When comparing the reference compound bearing a methylene group (**6a**) with other ester analogues, a similar trend to that noticed for solubility is observed. Indeed, Log*D* values increase with the introduction of lipophilic substituents such as carbocyclic rings or fluorine atoms (dimethyl **6b**, cyclobutyl **5e**, **6d**, cyclopentyl **6e** and difluoro **6f**, **6g**). An exception is noticed for cyclopropyl compound (**6c**) showing a similar Log*D* value. A lower Log*D* is observed by replacing the carboxylate group with cyano (**5e** *vs.* **6d**), but no significant change is detected for the phosphonate derivative (**6f** *vs.* **6g**). A decrease in Log*D* is observed with the installation of more hydrophilic 4- and 5-membered heterocycles bearing an oxygen atom in comparison with their carbocyclic ring equivalents (oxetane **6h** *vs.* cyclobutyl **5e**, THF **6i** *vs.* cyclopentyl **6e**). However, an increase in Log*D* is noticed for nitrogen-containing heterocycles (azetidine **6j** and piperidine **6k**), likely due to the lipophilicity of Boc-protecting groups and benzyl esters. Not surprisingly, the Log*D* decreases drastically for all the free acids (**5e'**, **6a'-6k'**) in comparison with the corresponding ester derivatives. In particular, high hydrophilicity is observed for compounds with oxetane and THF rings (**6h'** and **6i'**), as well as for the phosphonic acid analogue (**6g'**). Thus, *meta*-C–H alkylation could be considered as a potential strategy for modulating Log*D*, especially by decreasing the lipophilicity of poorly hydrophilic compounds.

A graphical representation of the observed changes in both solubility and Log*D* for the *meta*-C–H alkylated compounds, in comparison with the parent molecule *Emapunil*, is shown below in **Supplementary Figs. 69-70**.

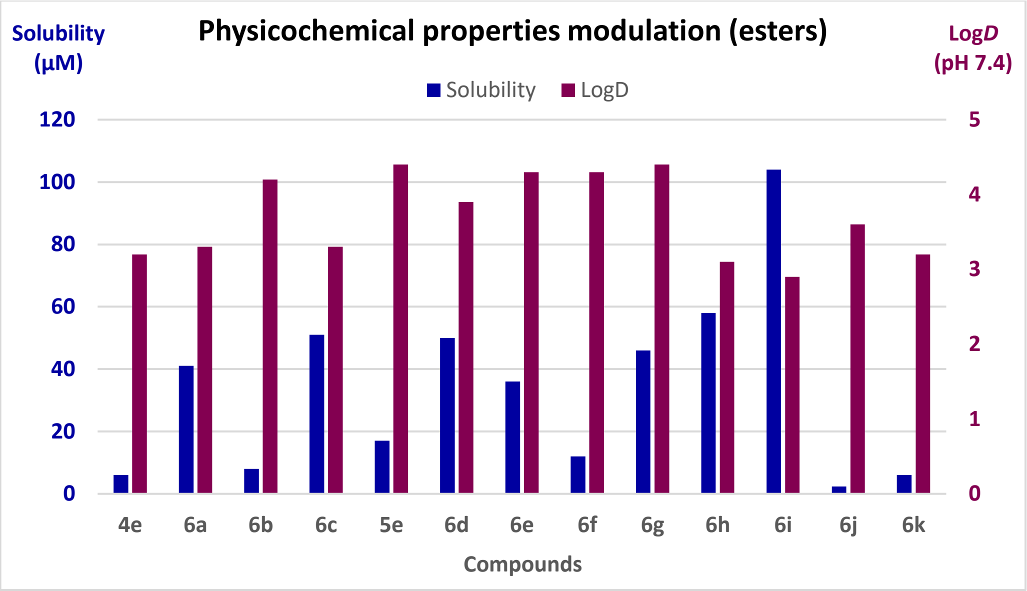

Supplementary Fig. 69. Graphical comparison of the solubility (µM) and Log*D* (pH 7.4) between the parent molecule *Emapunil* (**4e**) and the *meta*-C–H alkylated ester analogues (**5e**, **6a-6k**).

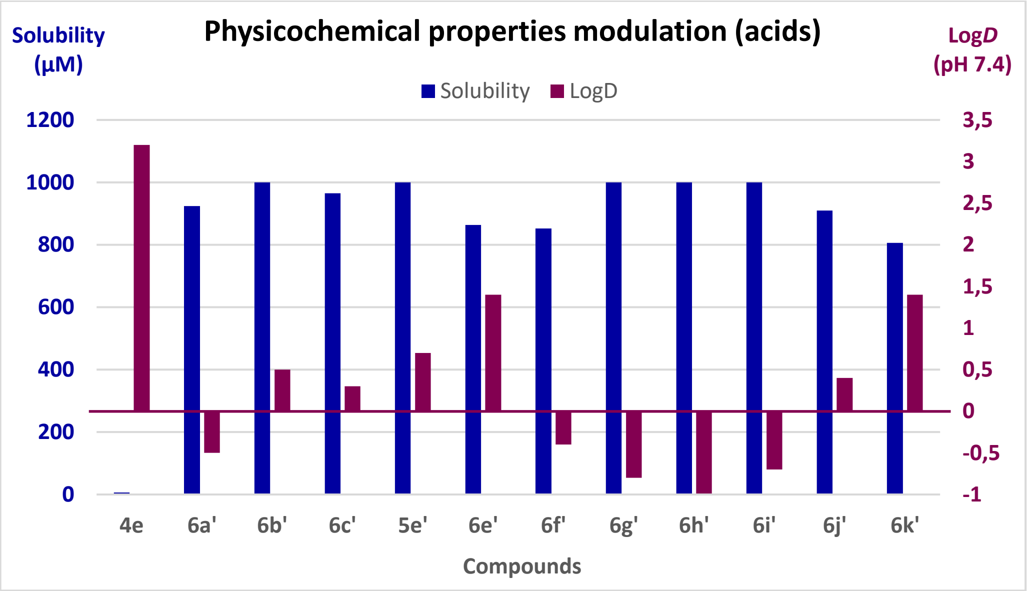

Supplementary Fig. 70. Graphical comparison of the solubility (µM) and Log*D* (pH 7.4) between the parent molecule *Emapunil* (**4e**) and the *meta*-C–H alkylated acid analogues (**5e'**, **6a'-6k'**).

Human plasma protein binding (Prot. Bind.)

Low unbound fractions are observed for the parent molecule *Emapunil* (**4e**) and most of the compounds bearing an ester group (**5e**, **6a-6k**), reflecting the high plasma protein binding of this chemical series. The values show no clear trend, but unbound fraction is generally higher for free acid derivatives (**5e'**, **6a'-6k'**). Significant impact is observed in some cases, notably for methylene (**6a'**), cyclopropyl (**6c'**), oxetane (**6h'**) and THF (**6i'**) analogues. Thus, *meta*-C–H alkylation could be envisaged if protein binding is considered as an optimisation parameter.

Intrinsic clearance in human liver microsomes (CL_{int} HLM)

High clearance values are observed for the parent molecule *Emapunil* (**4e**) and all compounds bearing an ester group (**5e**, **6a-6k**), reflecting the poor metabolic stability in human liver microsomes of this chemical series. Clearance values are above the detection limit (>300 µL/min/mg) for all of the latter analogues, likely due to hydrolysis of the ester groups during the assay. The corresponding free acid derivatives (**5e'**, **6a'-6k'**) were then prepared and used for comparison instead. These analogues are indeed much more stable, but the clearance values show no clear trend. Substantial changes are observed in some instances, notably with higher clearance for dimethyl (**6b'**), cyclobutyl (**5e'**), azetidine (**6j'**) and piperidine (**6k'**) compounds (likely due to the cleavage of Boc group in the last two cases). Thus, *meta*-C–H alkylation could be considered to improve the stability of metabolically unstable compounds.

Intrinsic clearance in rat hepatocytes (CL_{int} Rat Hep.)

High clearance values are observed for the parent molecule *Emapunil* (**4e**) and all compounds bearing an ester group (**5e**, **6a-6k**), reflecting the poor metabolic stability in rat hepatocytes of this chemical series. Clearance values are above the detection limit (>300 µL/min/10^6) for all of the latter analogues (except for piperidine compound **6k**), likely due to hydrolysis of the ester groups during the assay. The corresponding free acid derivatives (**5e'**, **6a'-6k'**) were then prepared and used for comparison instead. These analogues are indeed much more stable, but the clearance values show no clear trend. Substantial changes are observed in some instances, notably with higher clearance for cyclopropyl (**6c'**), cyclopentyl (**6e'**), oxetane (**6h'**), azetidine (**6j'**) and piperidine (**6k'**) compounds (likely due to the cleavage of Boc group in the last two cases) and lower clearance for difluoro derivatives (**6f'**, **6g'**). Thus, *meta*-C–H alkylation could be considered to improve the stability of metabolically unstable compounds.

A graphical representation of the observed changes in intrinsic clearance both in human liver microsomes and rat hepatocytes for the *meta*-C–H alkylated free acids, in comparison with the parent molecule *Emapunil*, is shown below in **Supplementary Fig. 71**.

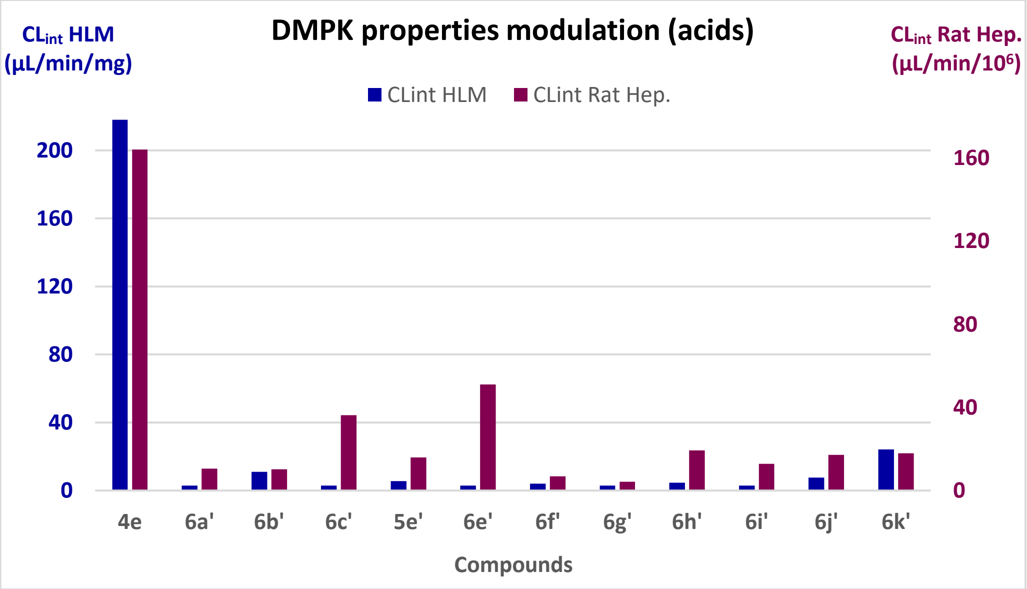

Supplementary Fig. 71. Graphical comparison of the intrinsic clearance (CL_{int}) in human liver microsomes (HLM, in µL/min/mg) and in rat hepatocytes (Rat Hep., in µL/min/10^6) between the parent molecule *Emapunil* (**4e**) and the *meta*-C–H alkylated acid analogues (**5e'**, **6a'-6k'**). DMPK = drug metabolism and pharmacokinetics.

6. NMR spectra (^{1}H, ^{13}C, ^{19}F and ^{31}P)

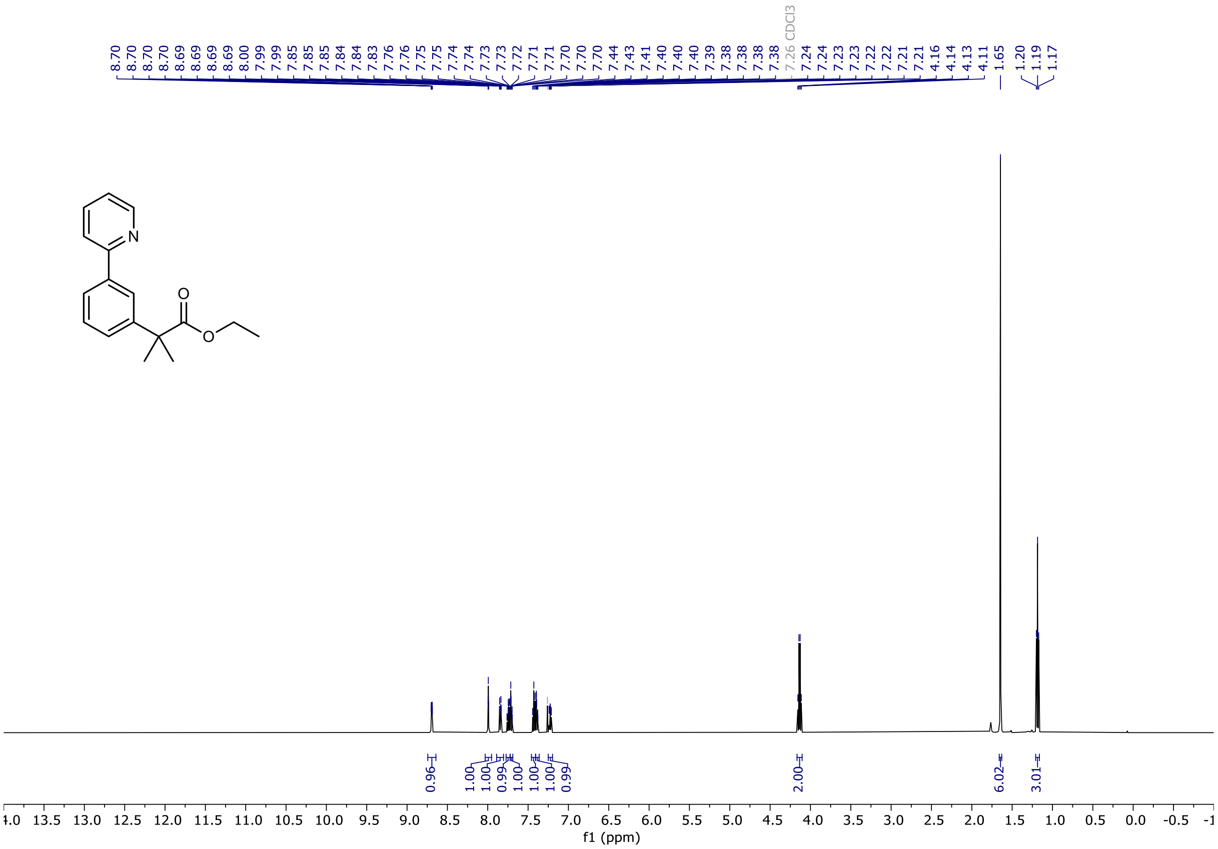

Supplementary Fig. 72. ^{1}H NMR spectra (500 MHz, $CDCl_3$, 298 K) of compound **3a**.

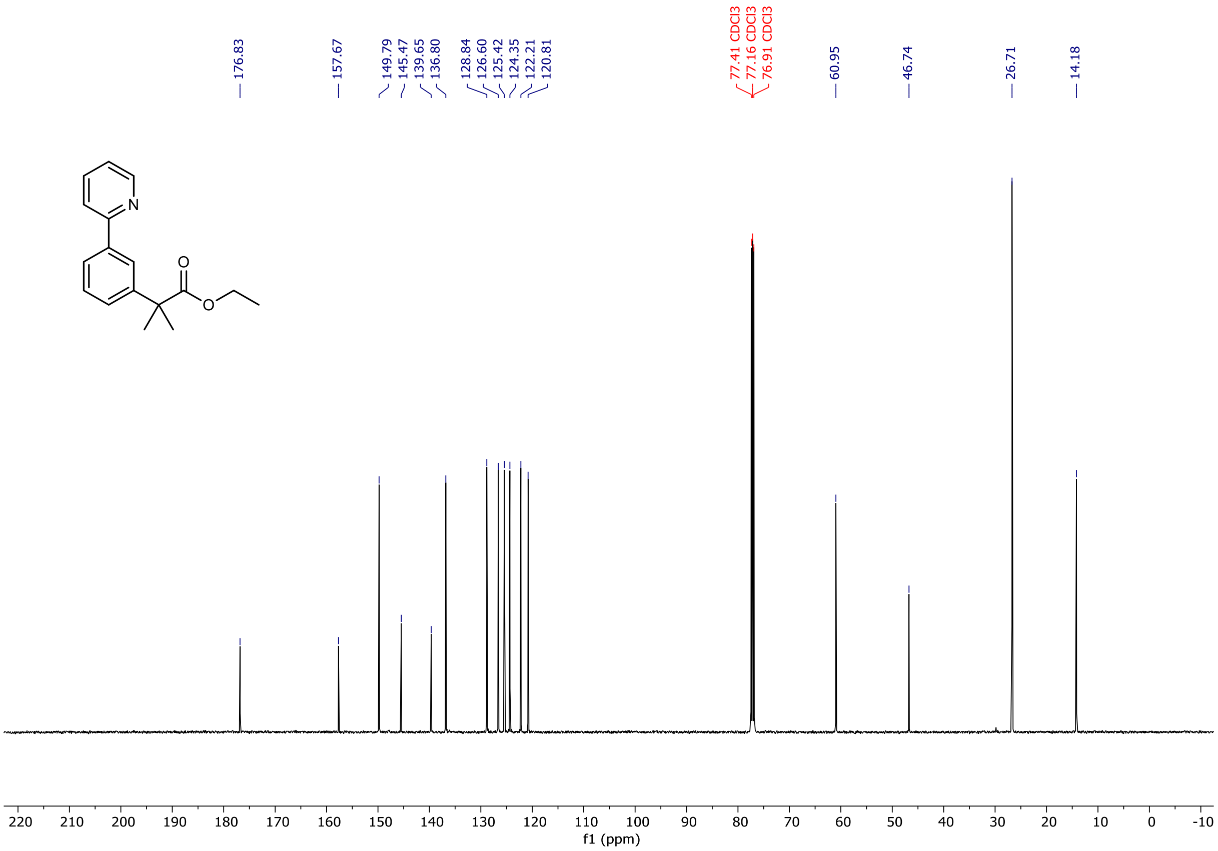

Supplementary Fig. 73. ^{13}C NMR spectra (126 MHz, $CDCl_3$, 298 K) of compound **3a**.

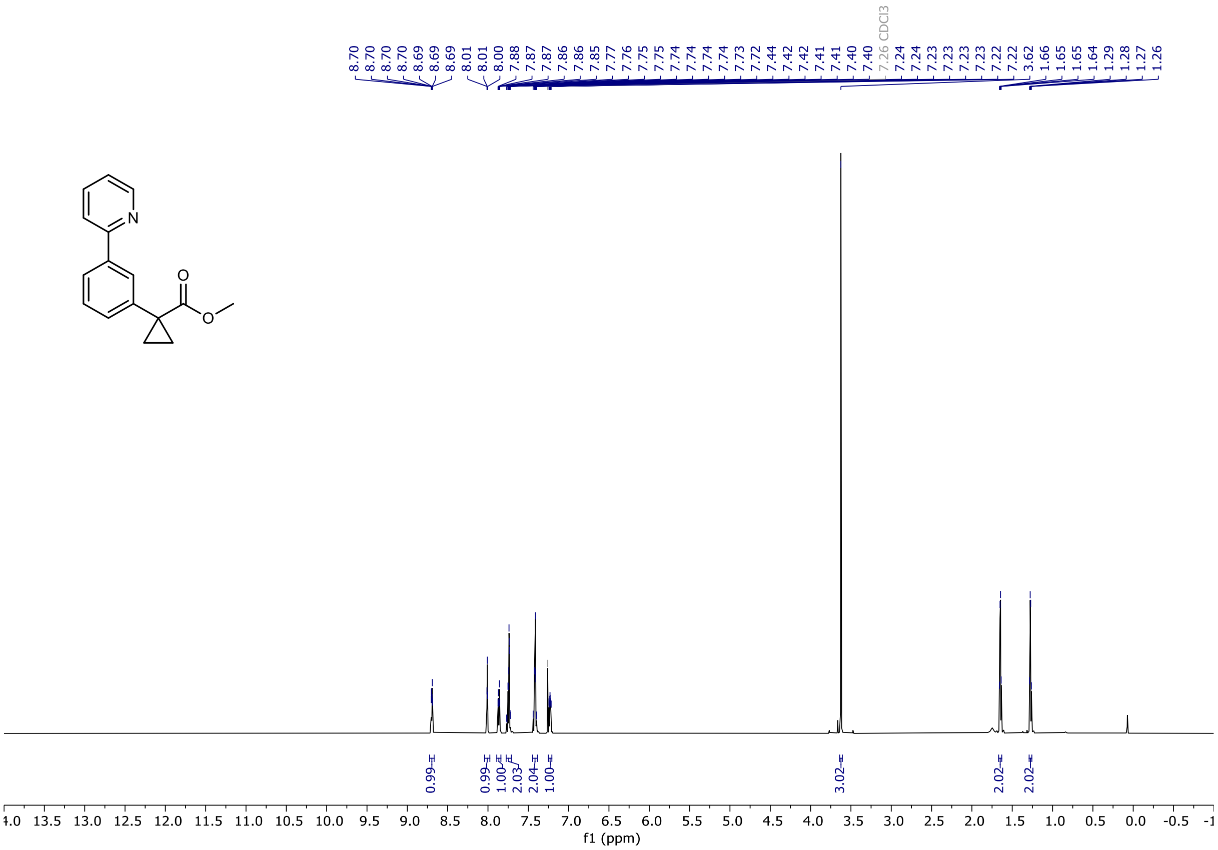

Supplementary Fig. 74. ^{1}H NMR spectra (500 MHz, $CDCl_3$, 298 K) of compound **3b**.

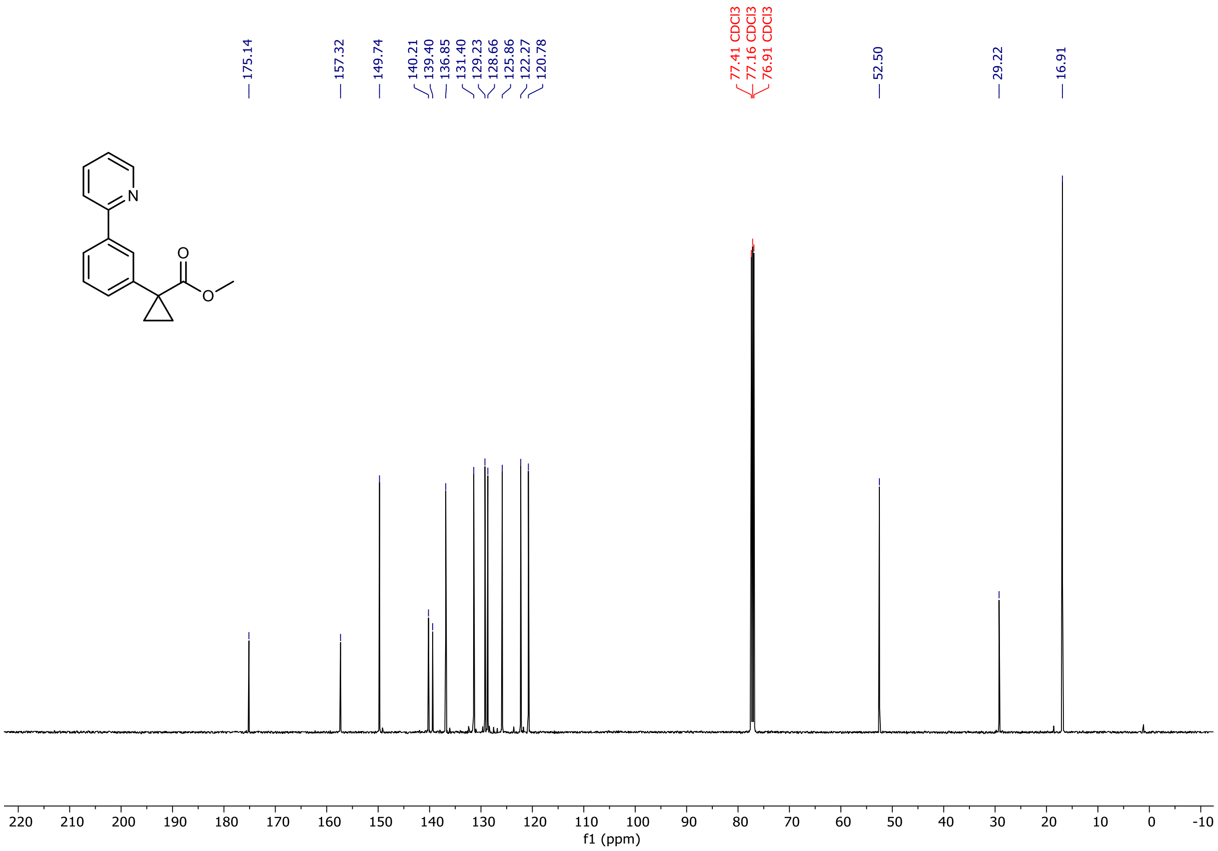

Supplementary Fig. 75. ^{13}C NMR spectra (126 MHz, $CDCl_3$, 298 K) of compound **3b**.

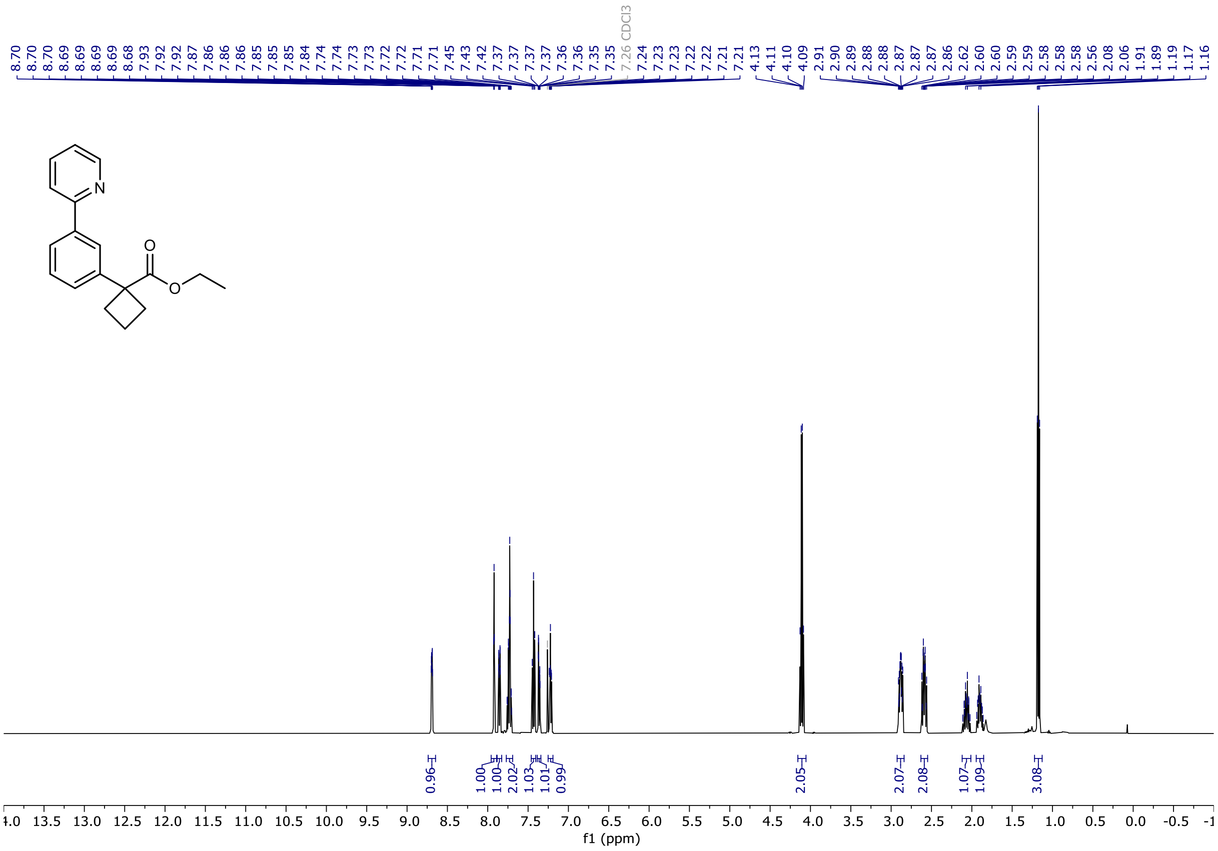

Supplementary Fig. 76. ^{1}H NMR spectra (500 MHz, $CDCl_3$, 298 K) of compound **3c**.

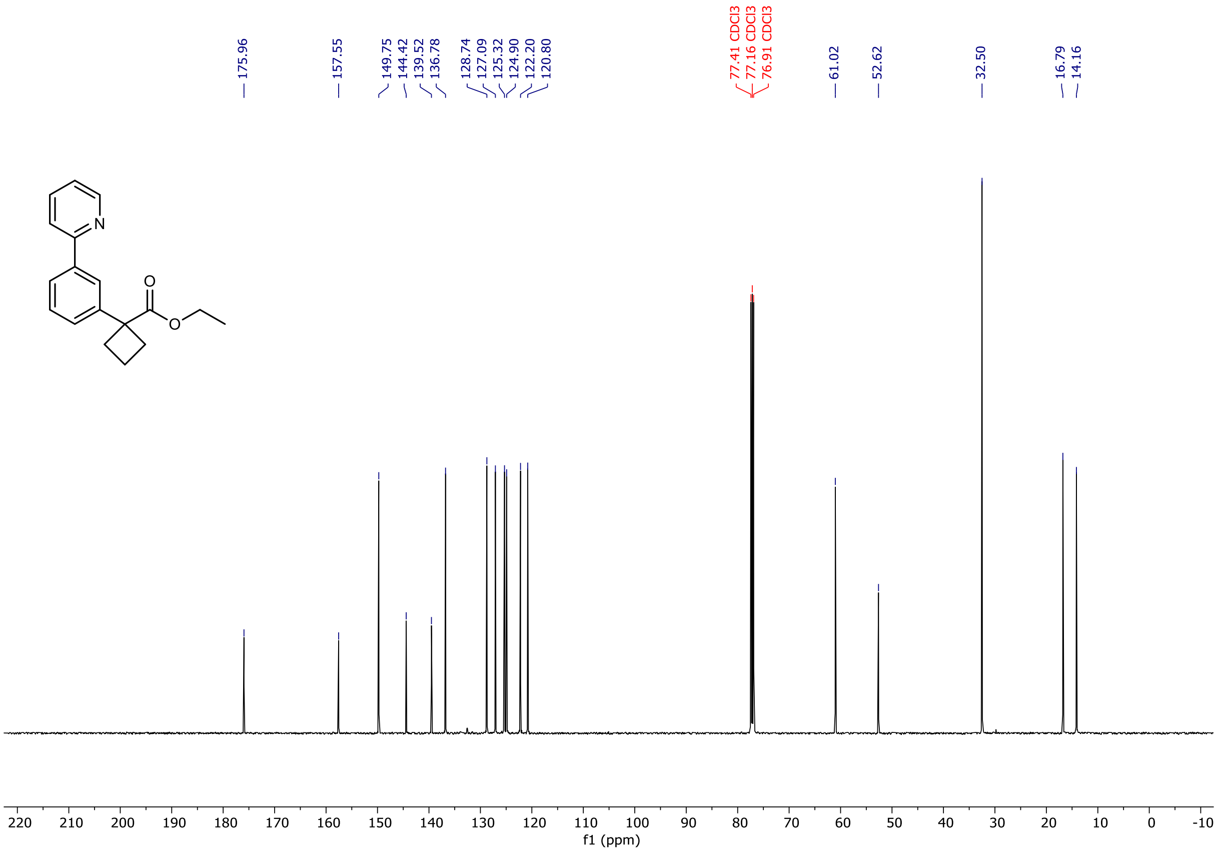

Supplementary Fig. 77. ^{13}C NMR spectra (126 MHz, $CDCl_3$, 298 K) of compound **3c**.

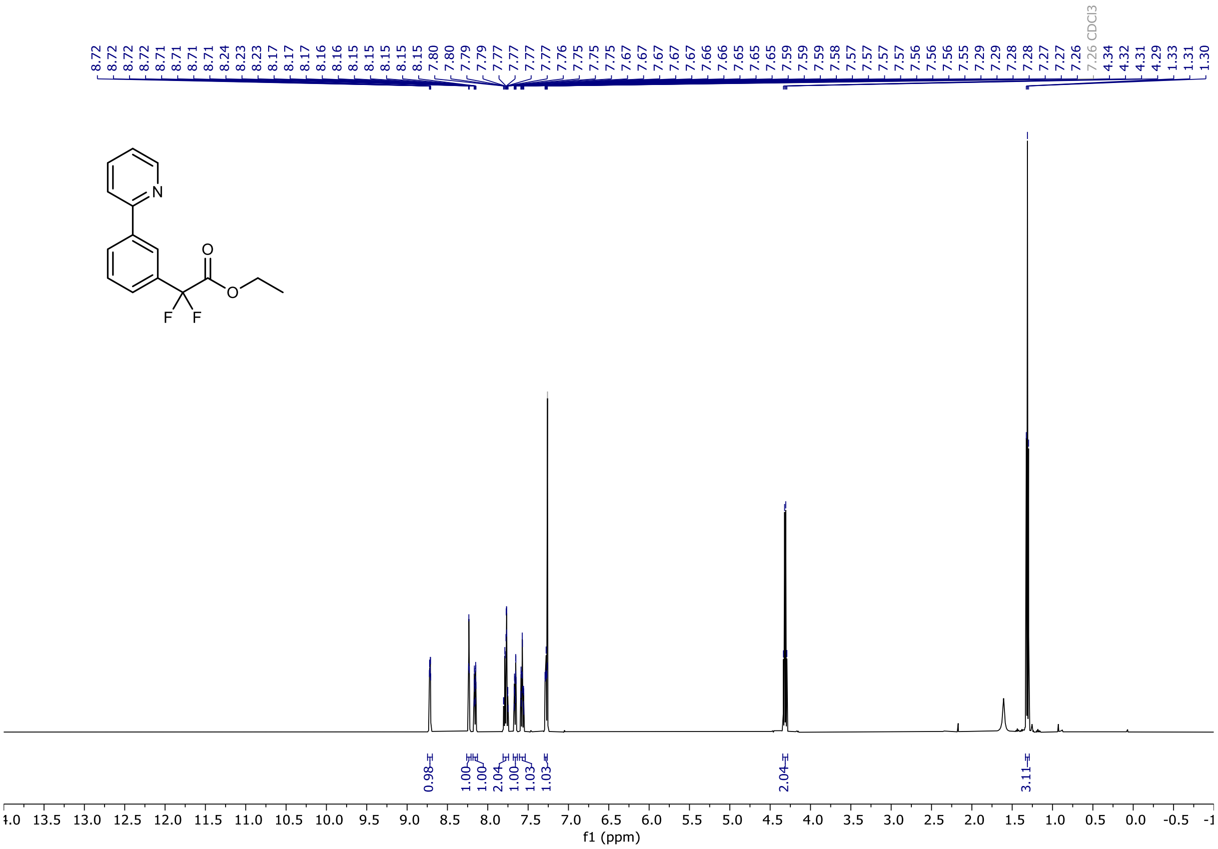

Supplementary Fig. 78. ^{1}H NMR spectra (500 MHz, $CDCl_3$, 298 K) of compound **3d**.

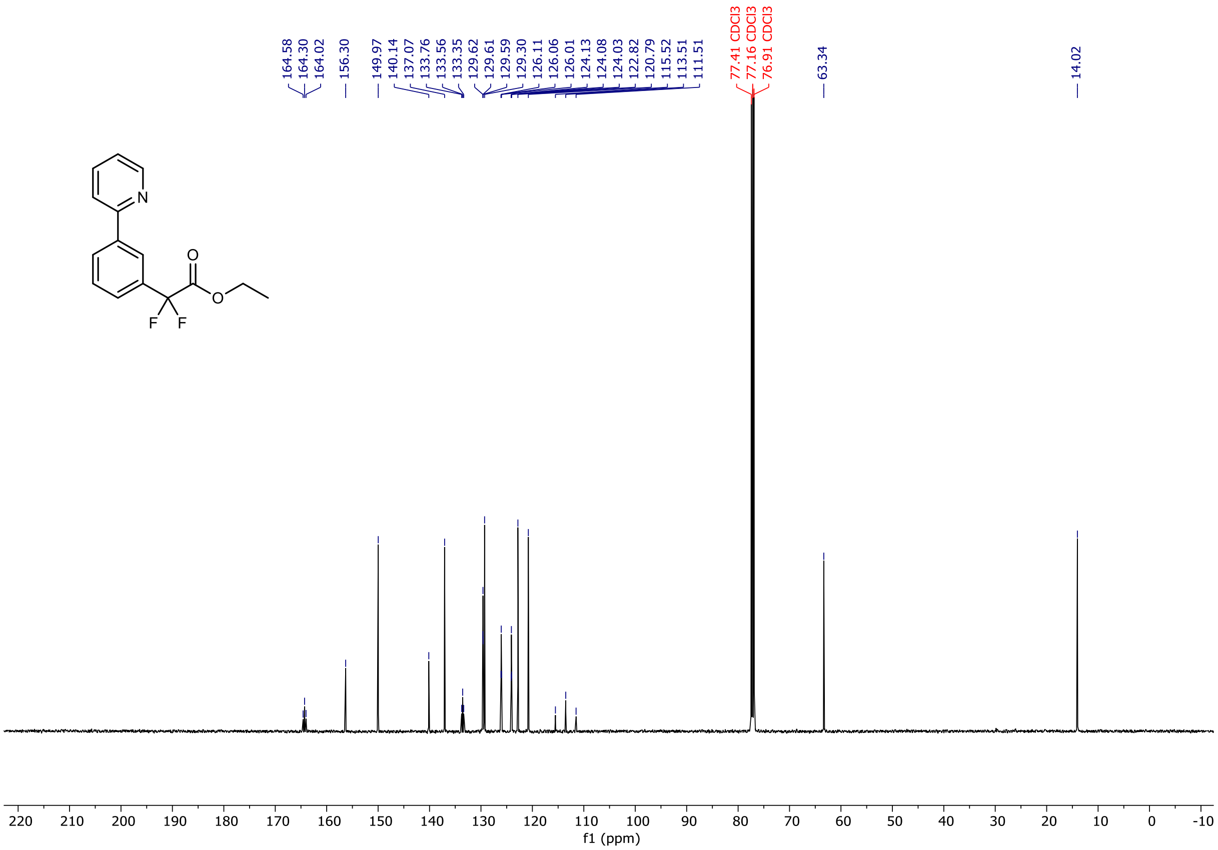

Supplementary Fig. 79. ^{13}C NMR spectra (126 MHz, $CDCl_3$, 298 K) of compound **3d**.

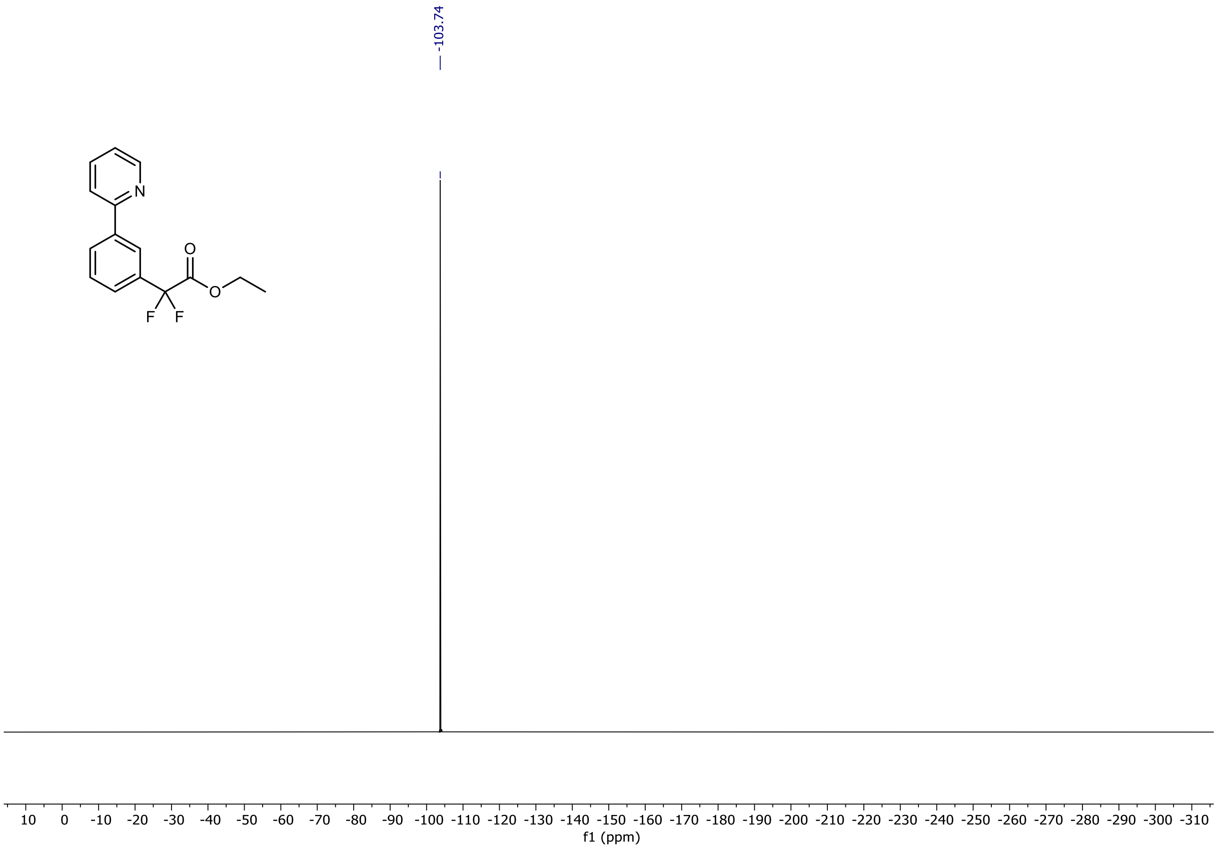

Supplementary Fig. 80. ^{19}F NMR spectra (471 MHz, $CDCl_3$, 298 K) of compound **3d**.

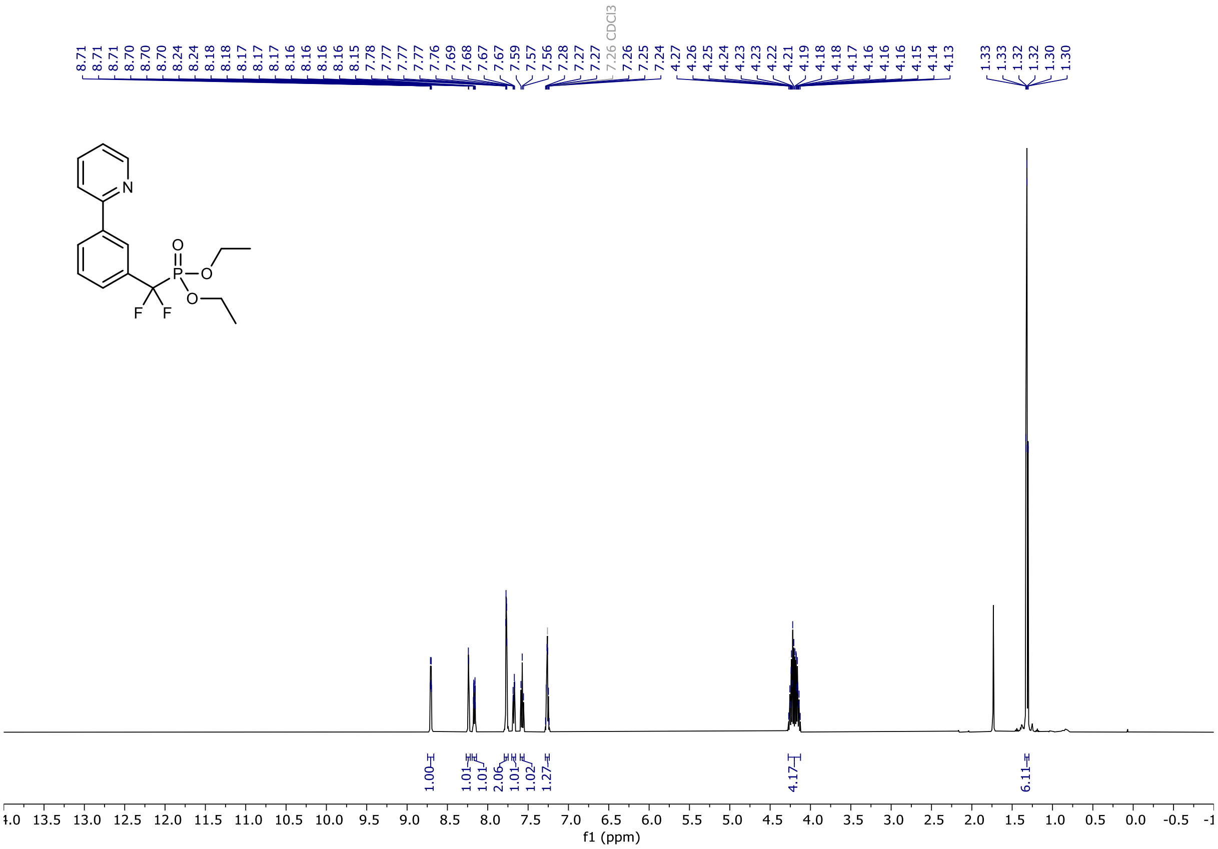

Supplementary Fig. 81. ^{1}H NMR spectra (500 MHz, $CDCl_3$, 298 K) of compound **3e**.

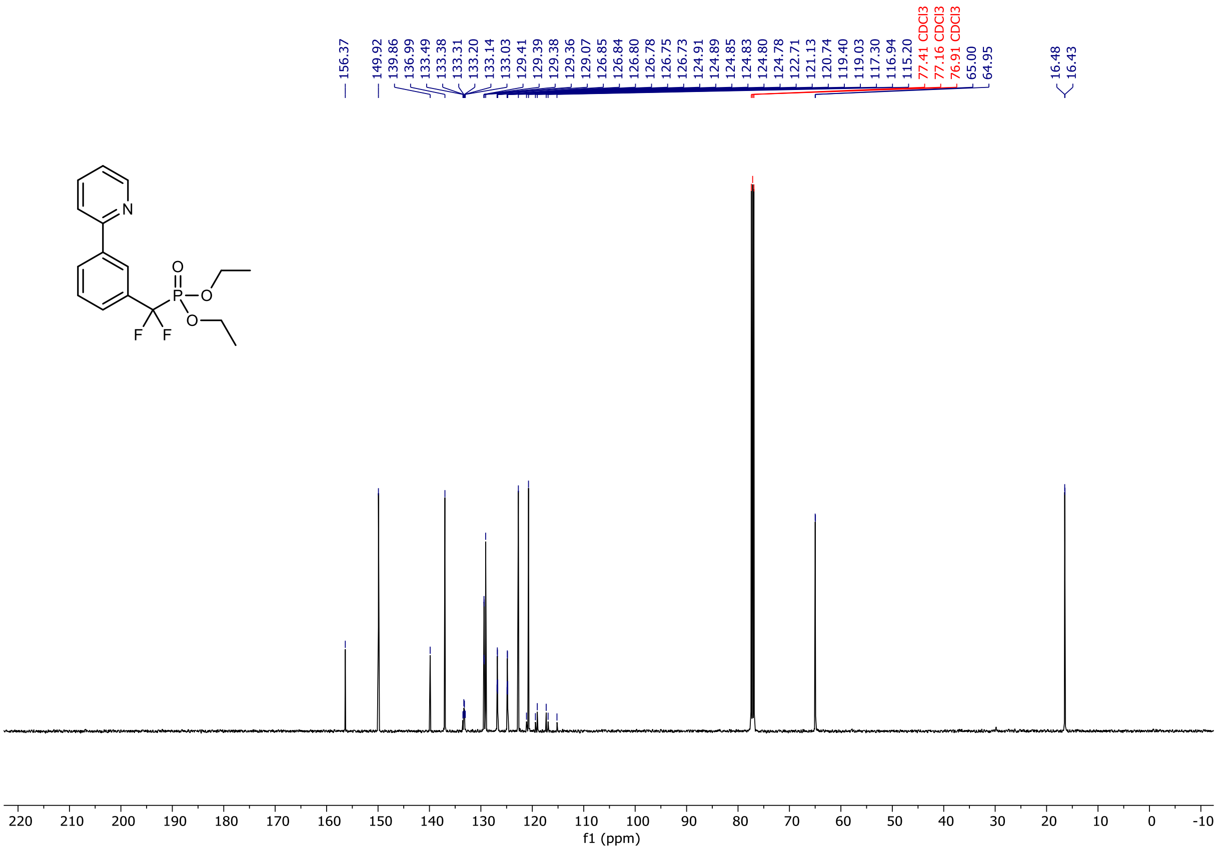

Supplementary Fig. 82. ^{13}C NMR spectra (126 MHz, $CDCl_3$, 298 K) of compound **3e**.

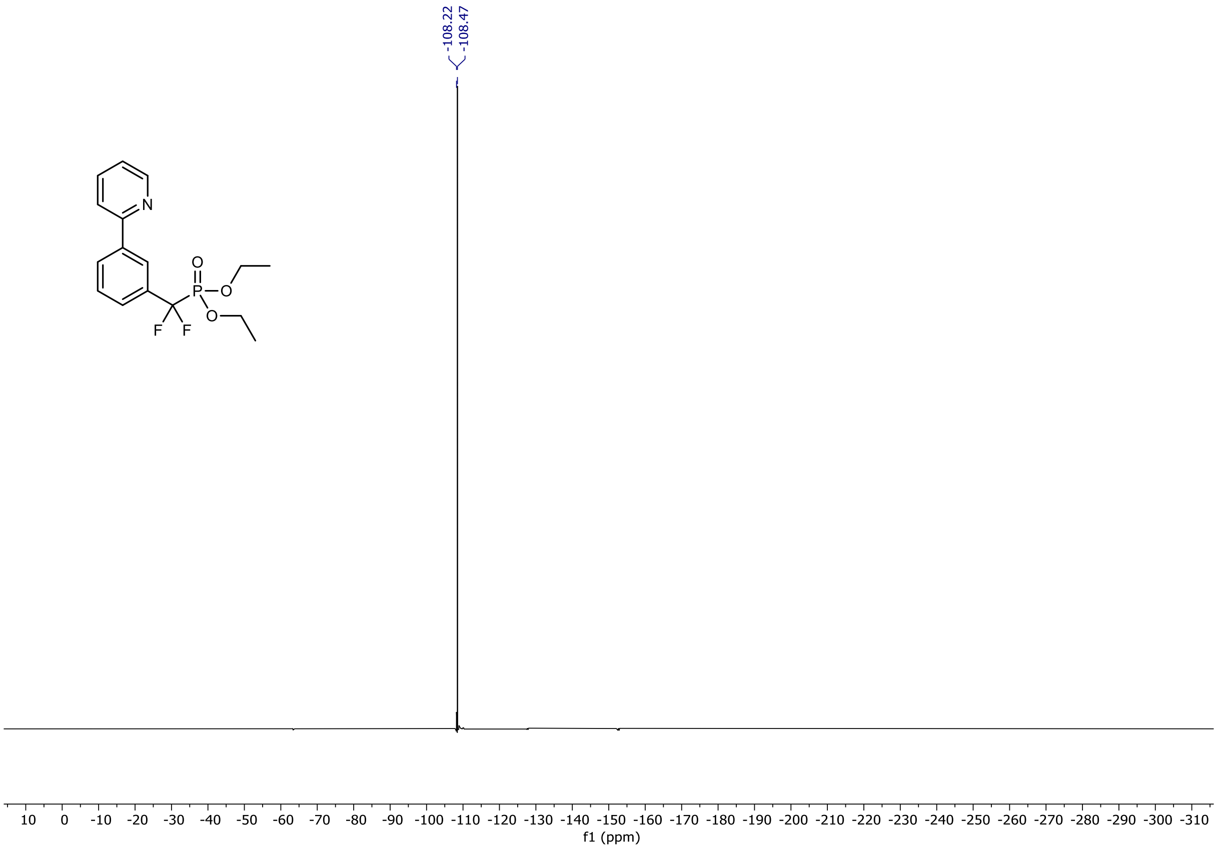

Supplementary Fig. 83. ^{19}F NMR spectra (471 MHz, $CDCl_3$, 298 K) of compound **3e**.

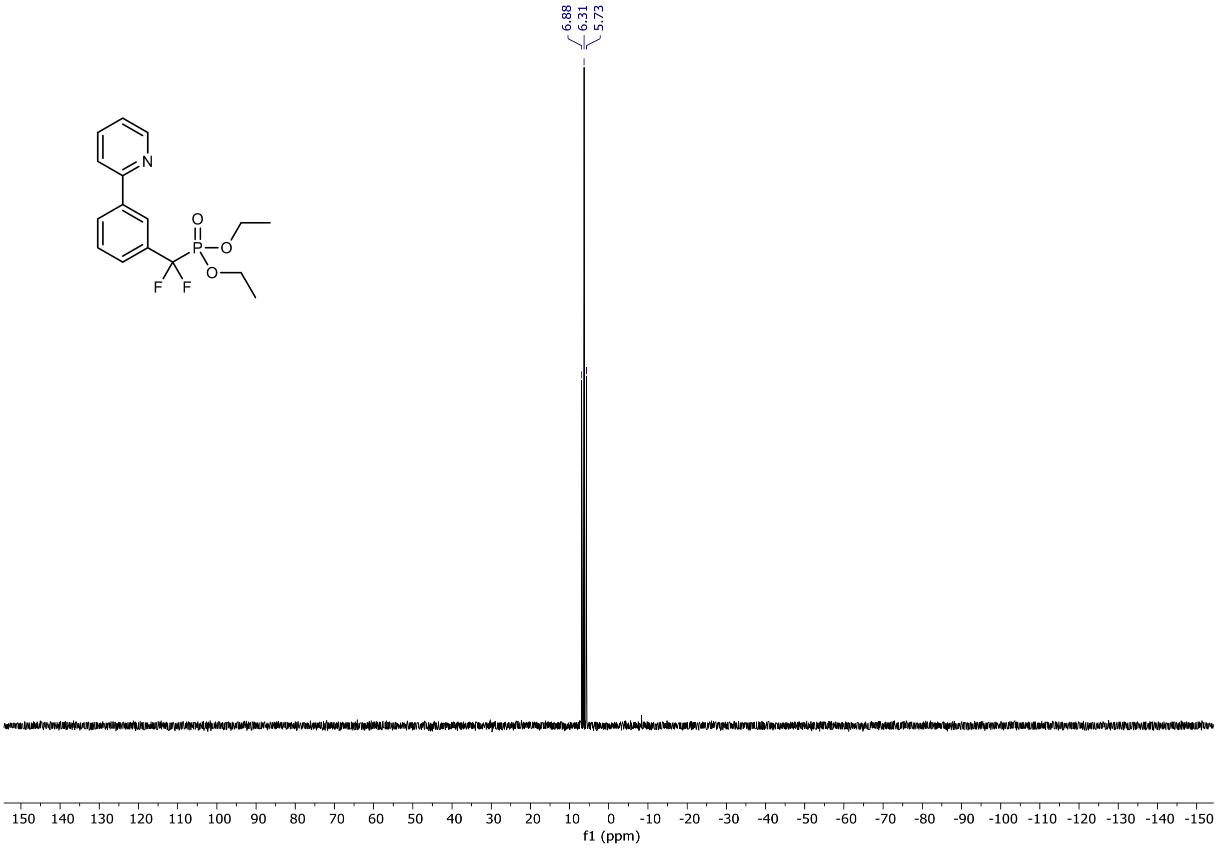

Supplementary Fig. 84. ^{31}P NMR spectra (203 MHz, $CDCl_3$, 298 K) of compound **3e**.

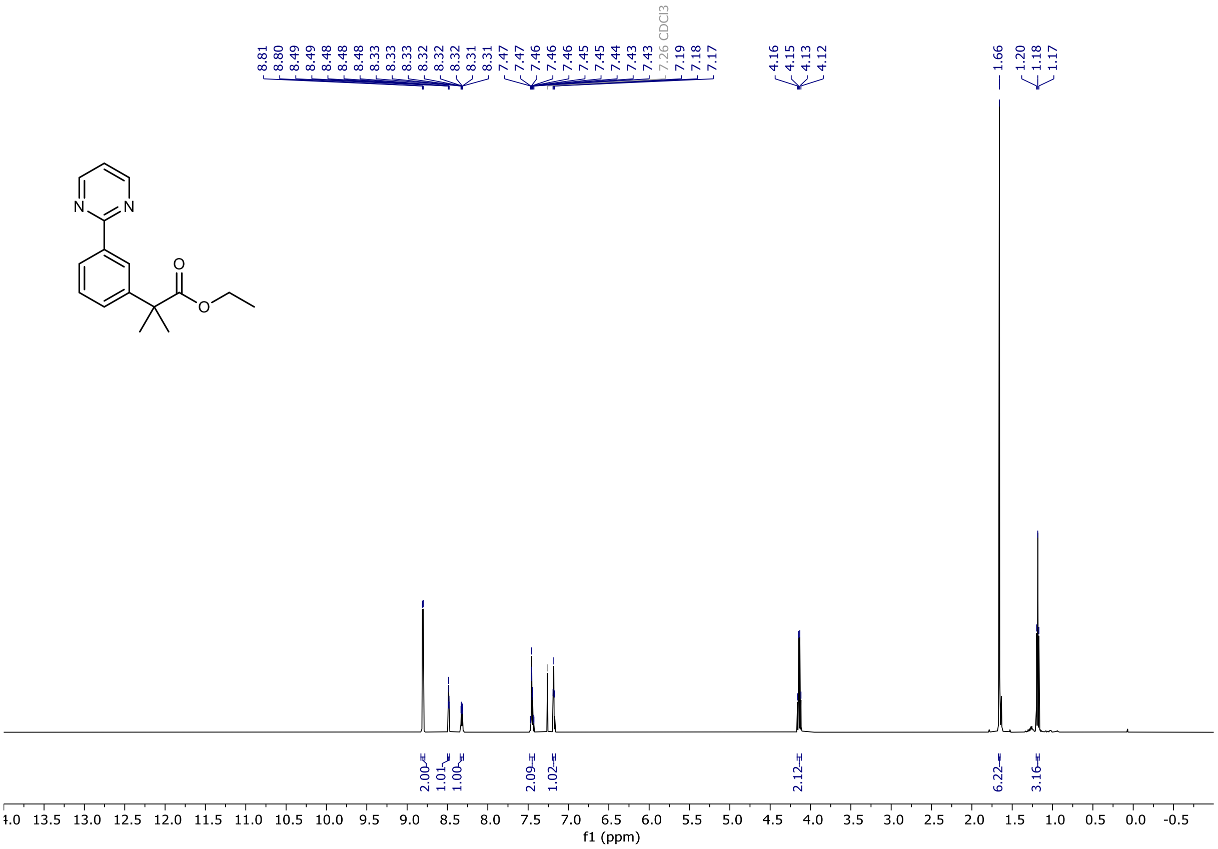

Supplementary Fig. 85. ^{1}H NMR spectra (500 MHz, $CDCl_3$, 298 K) of compound **3f**.

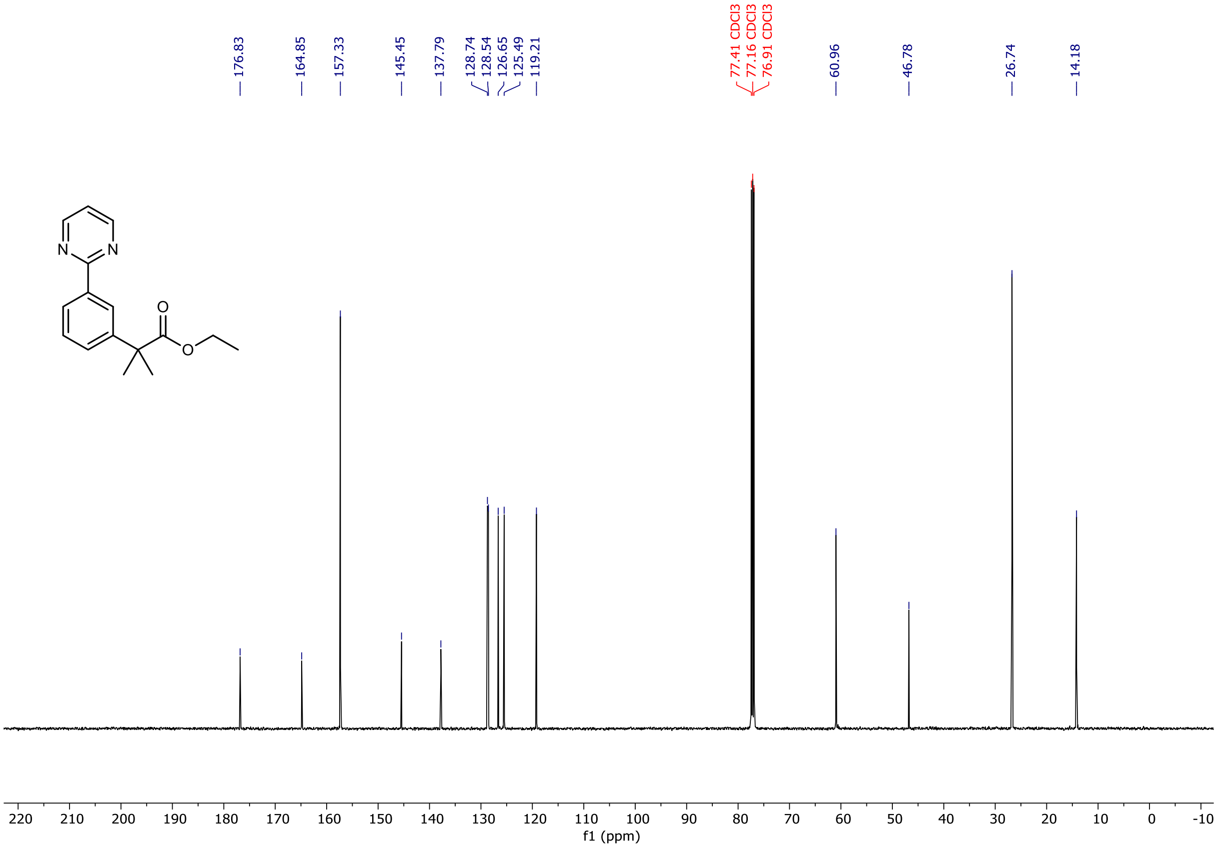

Supplementary Fig. 86. ^{13}C NMR spectra (126 MHz, $CDCl_3$, 298 K) of compound **3f**.

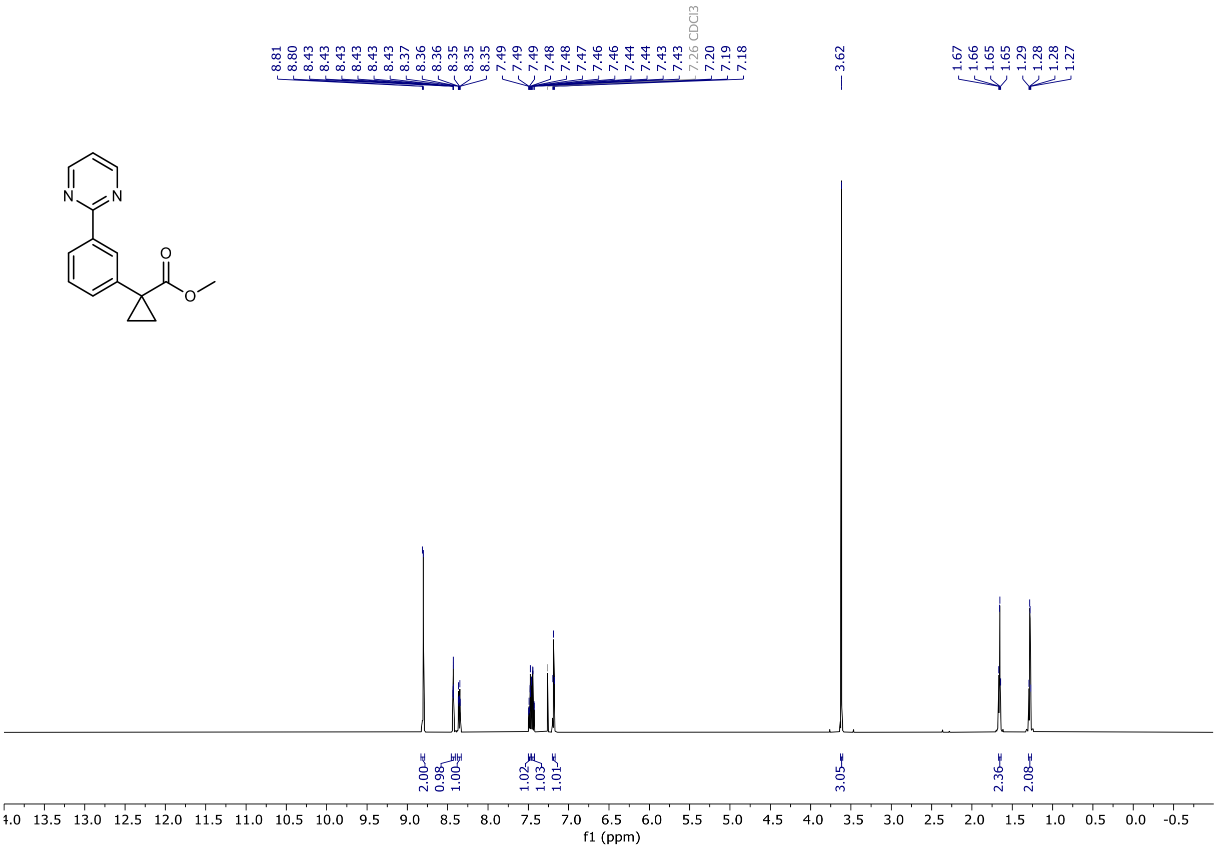

Supplementary Fig. 87. ^{1}H NMR spectra (500 MHz, $CDCl_3$, 298 K) of compound **3g**.

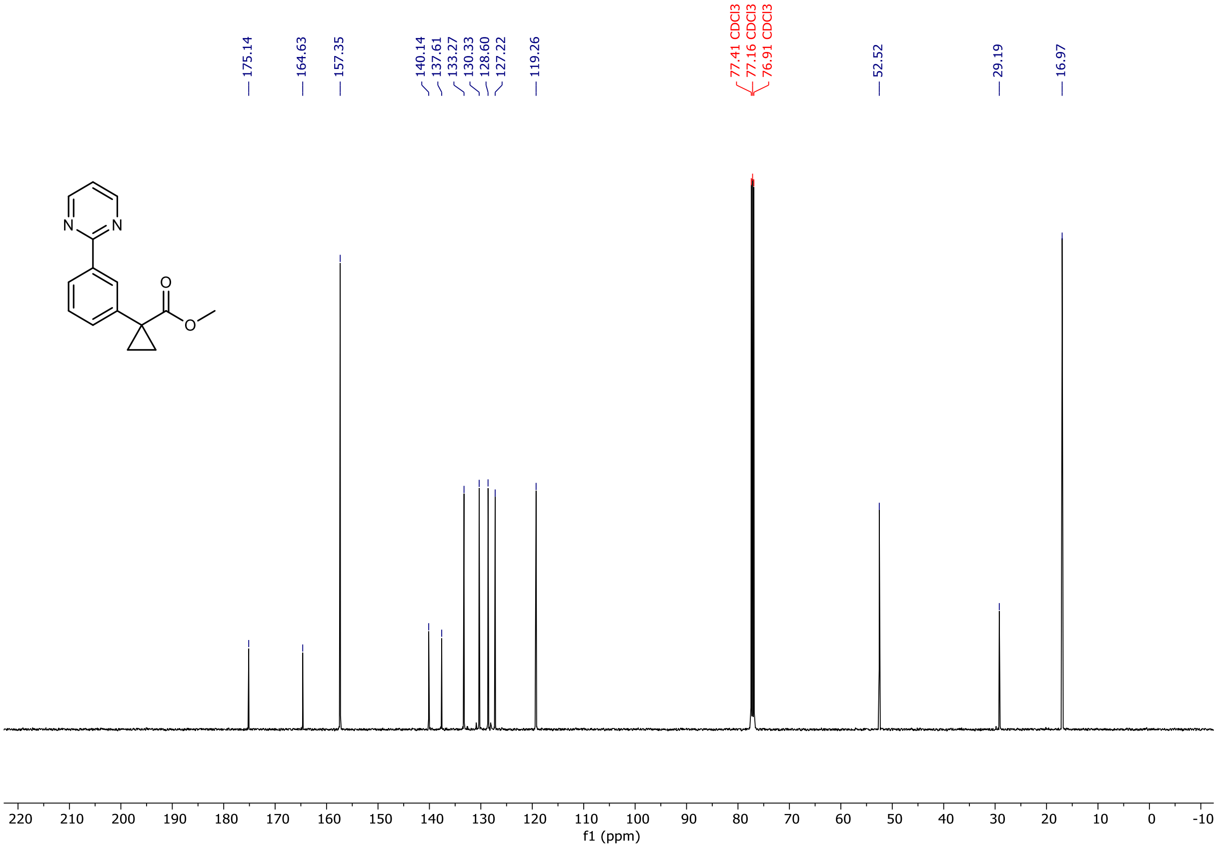

Supplementary Fig. 88. ^{13}C NMR spectra (126 MHz, $CDCl_3$, 298 K) of compound **3g**.

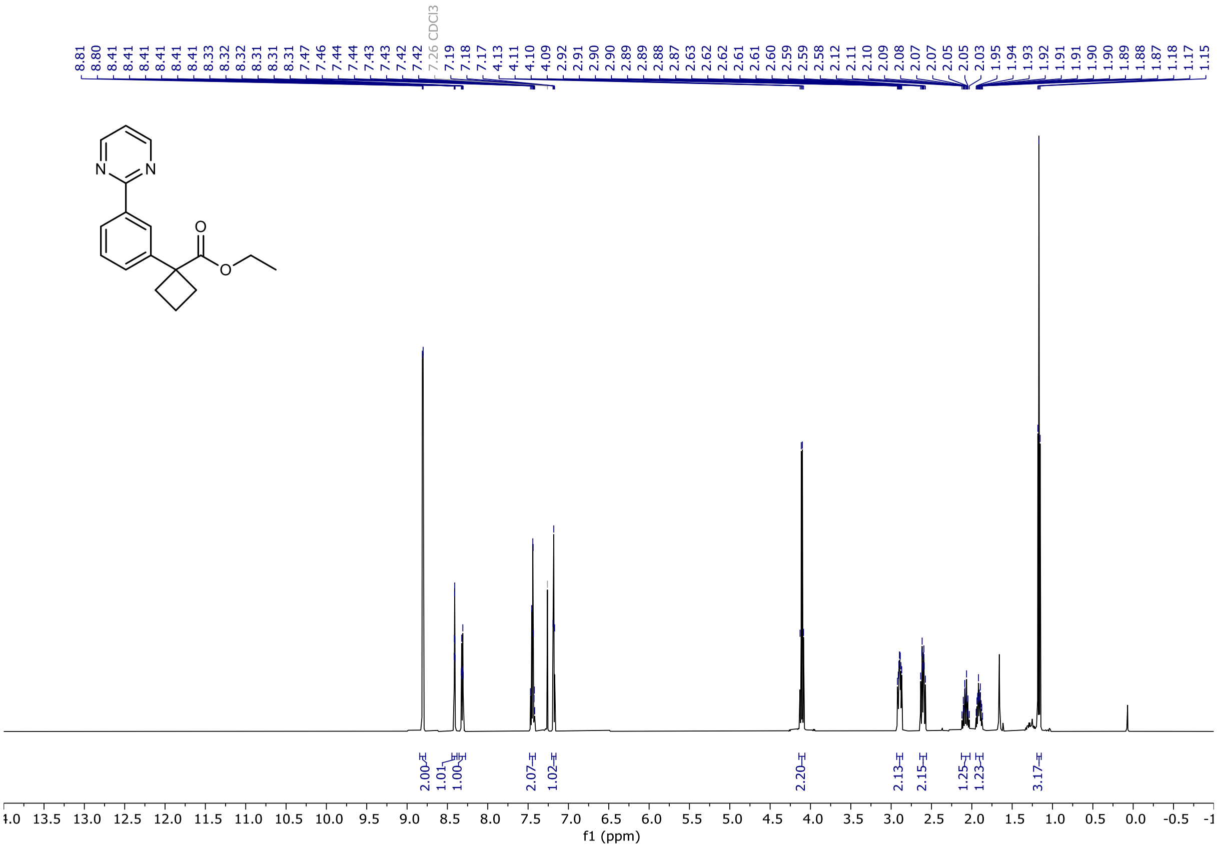

Supplementary Fig. 89. ^{1}H NMR spectra (500 MHz, $CDCl_3$, 298 K) of compound **3h**.

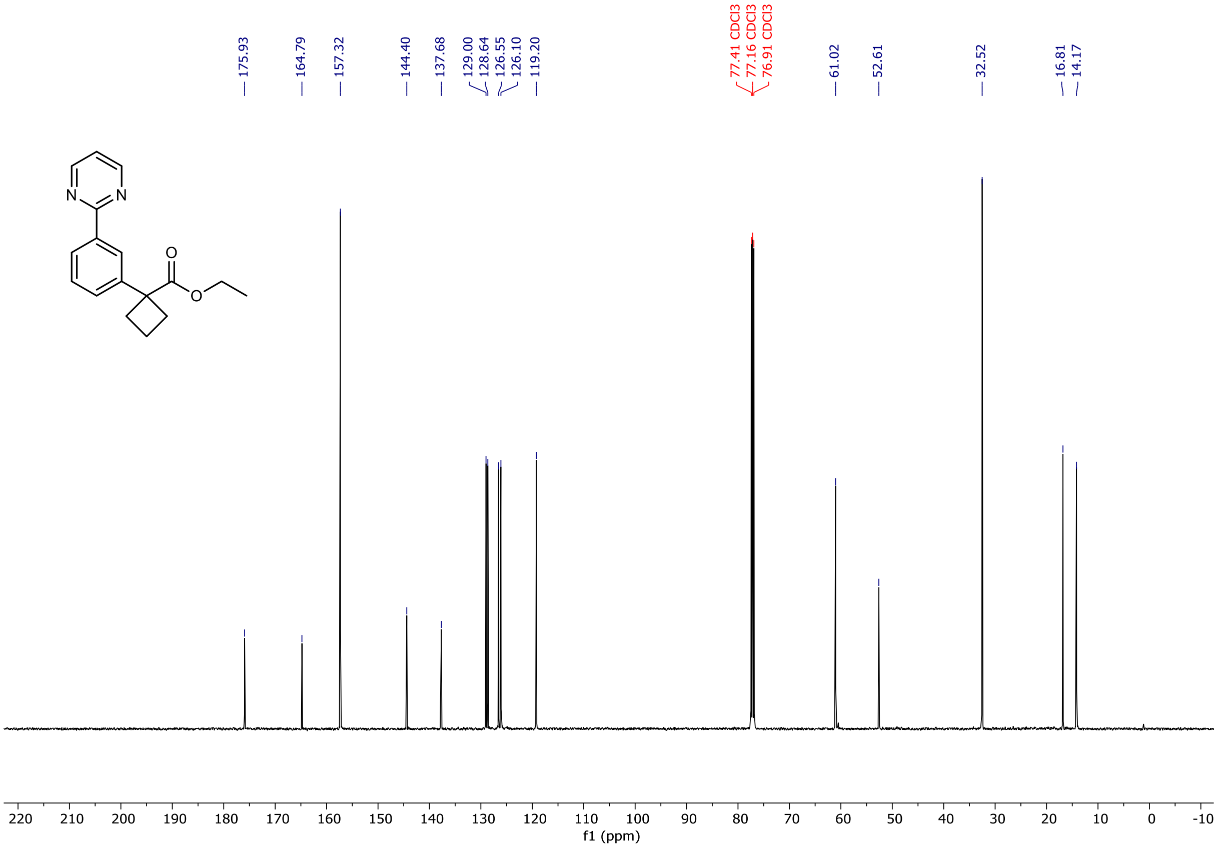

Supplementary Fig. 90. ^{13}C NMR spectra (126 MHz, $CDCl_3$, 298 K) of compound **3h**.

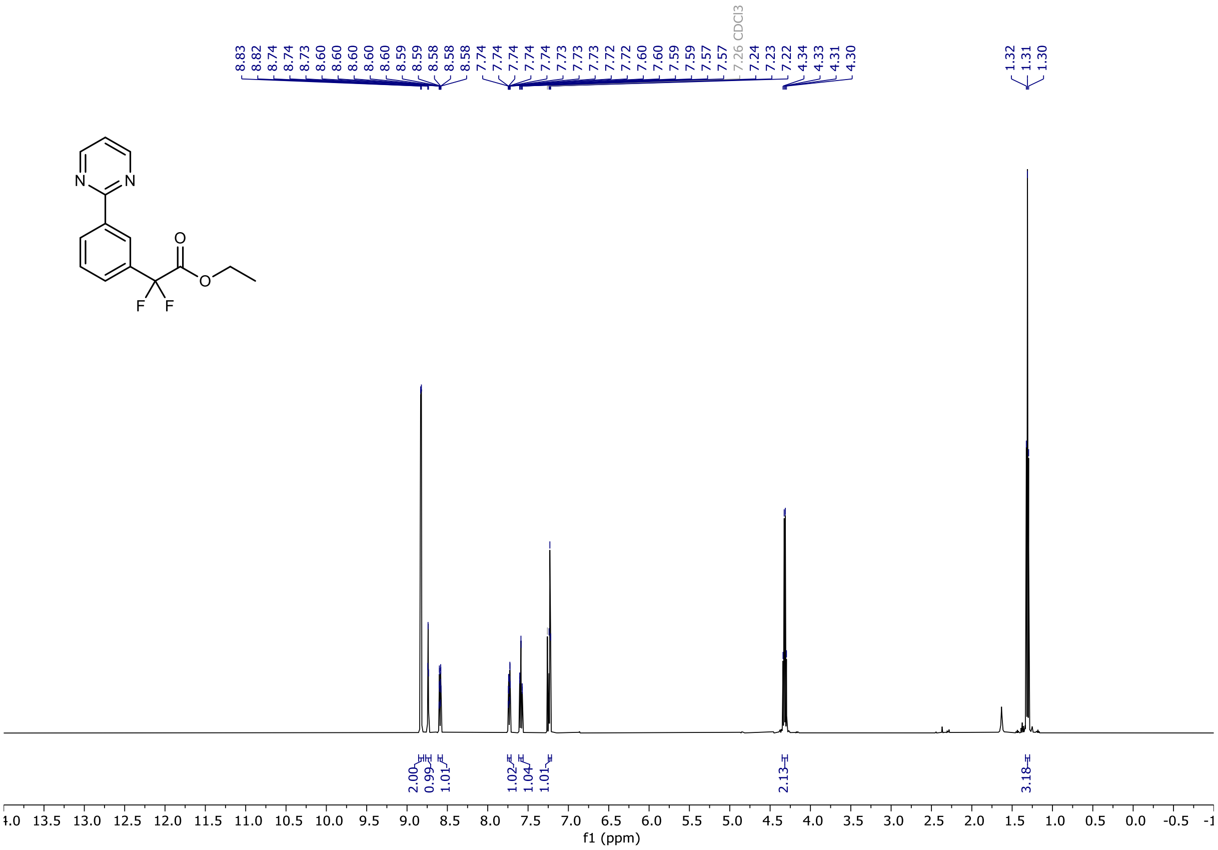

Supplementary Fig. 91. ^{1}H NMR spectra (500 MHz, $CDCl_3$, 298 K) of compound **3i**.

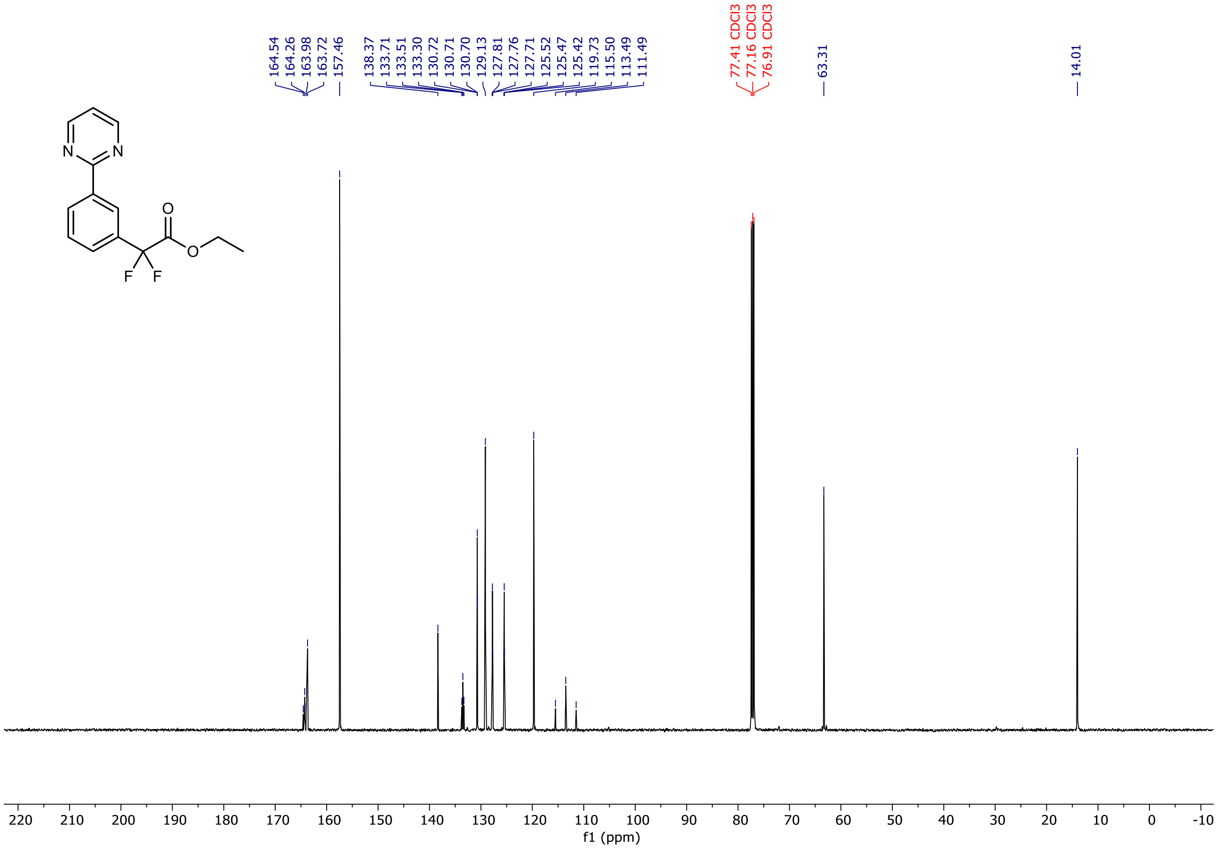

Supplementary Fig. 92. ^{13}C NMR spectra (126 MHz, $CDCl_3$, 298 K) of compound **3i**.

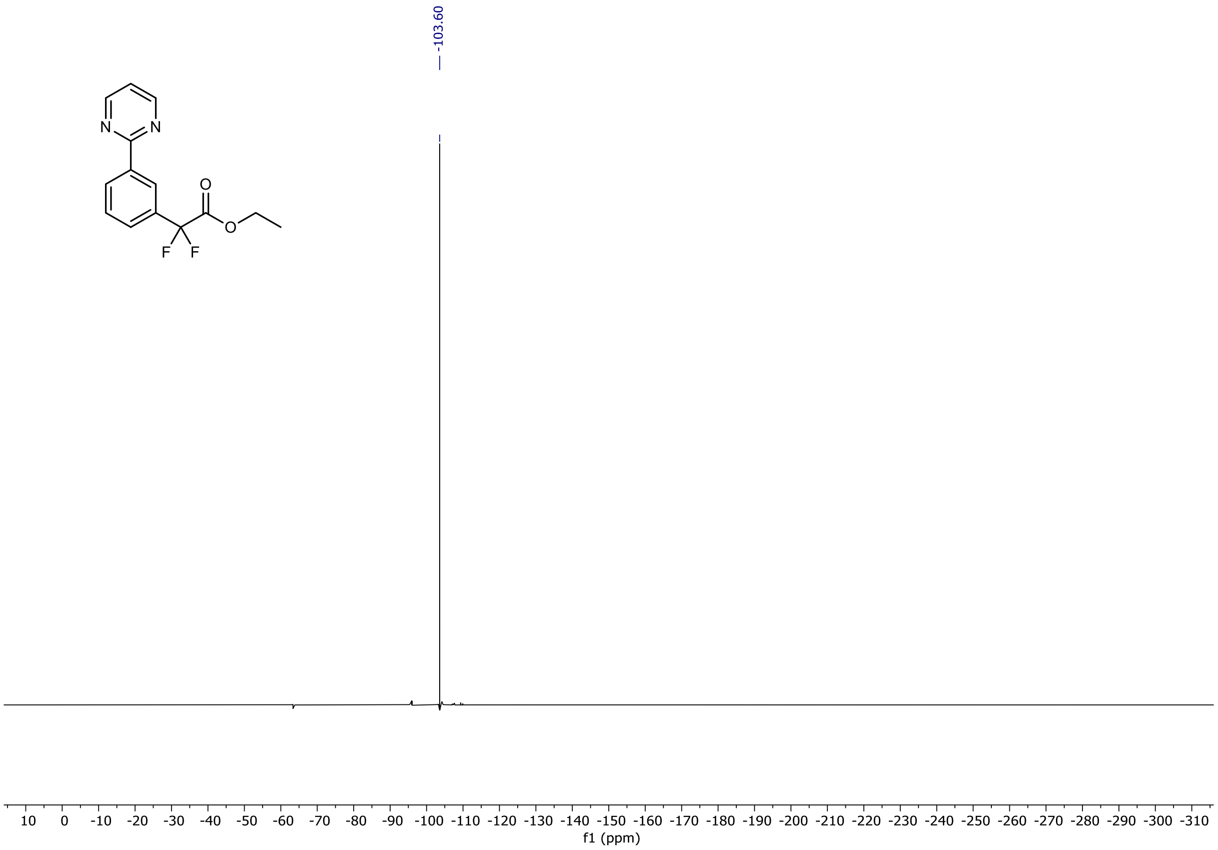

Supplementary Fig. 93. ^{19}F NMR spectra (471 MHz, $CDCl_3$, 298 K) of compound **3i**.

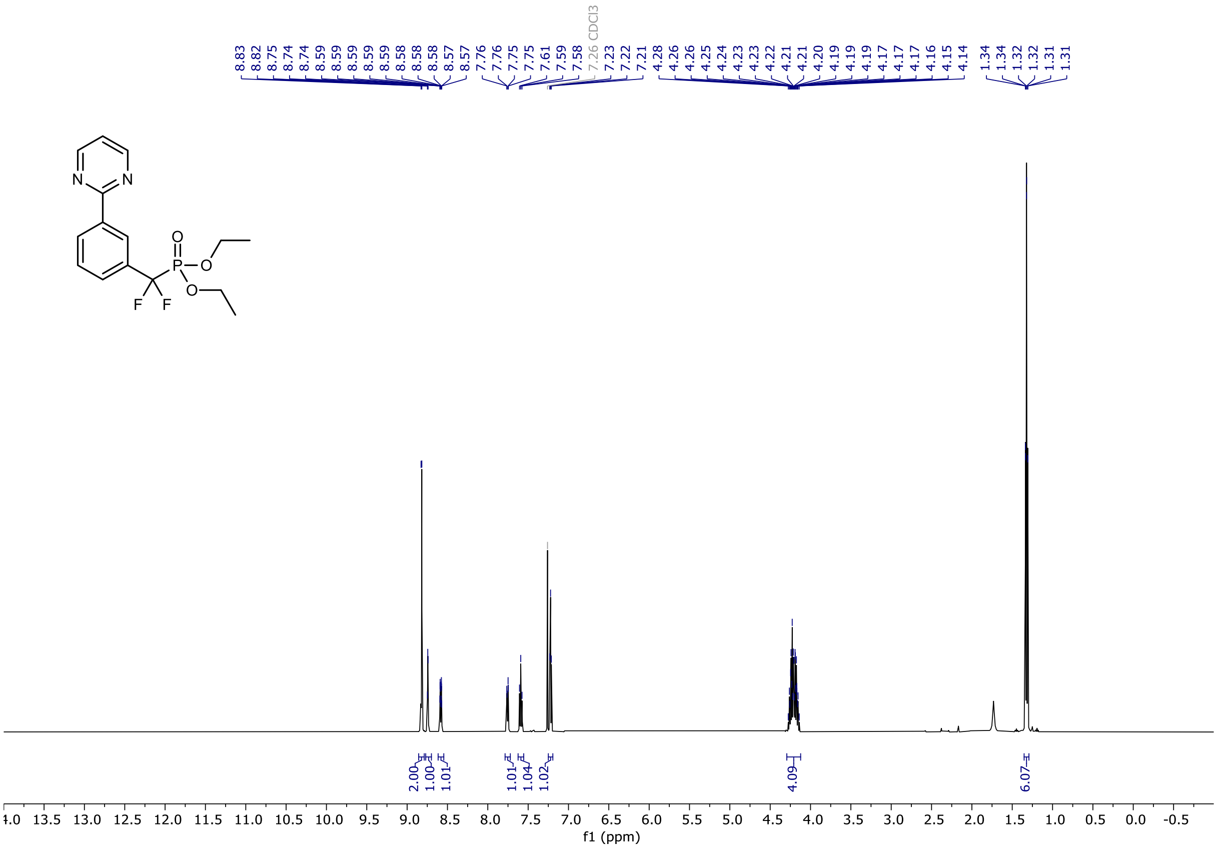

Supplementary Fig. 94. ^{1}H NMR spectra (500 MHz, $CDCl_3$, 298 K) of compound **3j**.

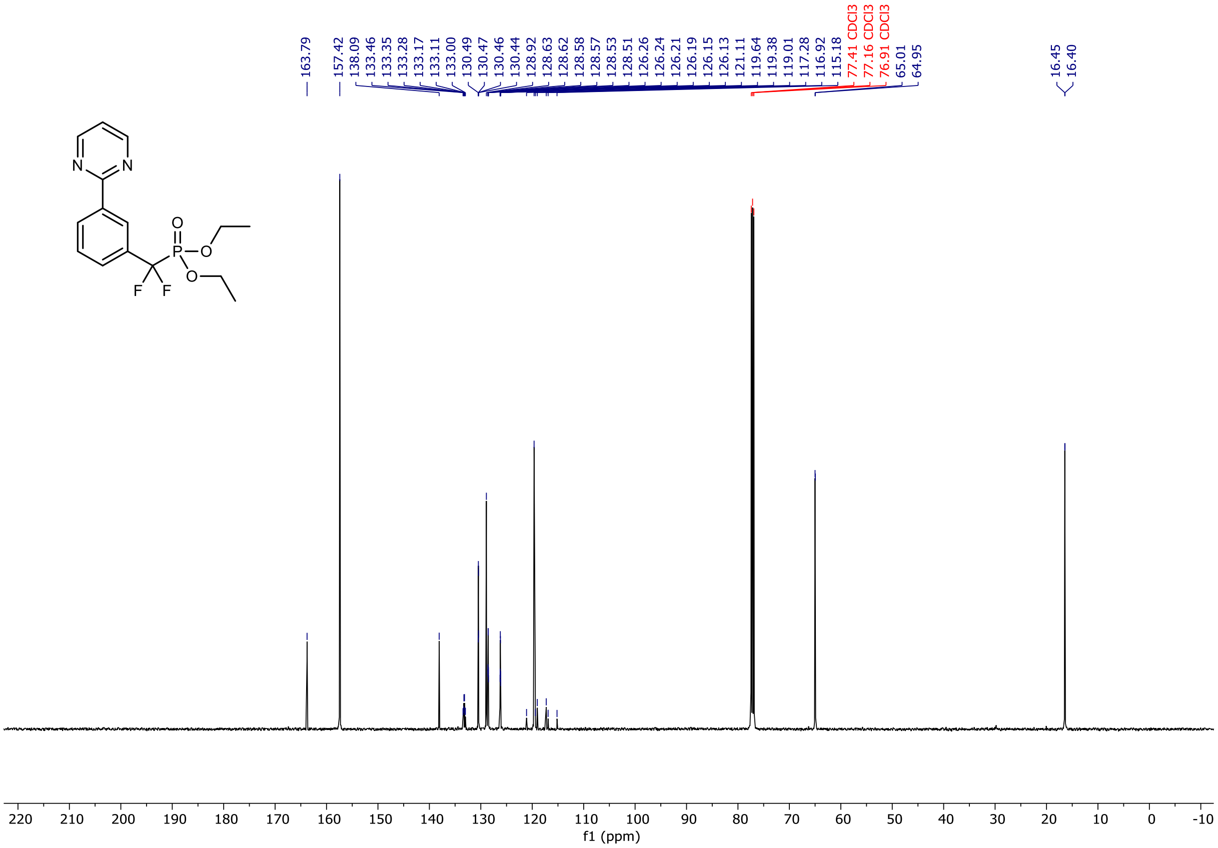

Supplementary Fig. 95. ^{13}C NMR spectra (126 MHz, $CDCl_3$, 298 K) of compound **3j**.

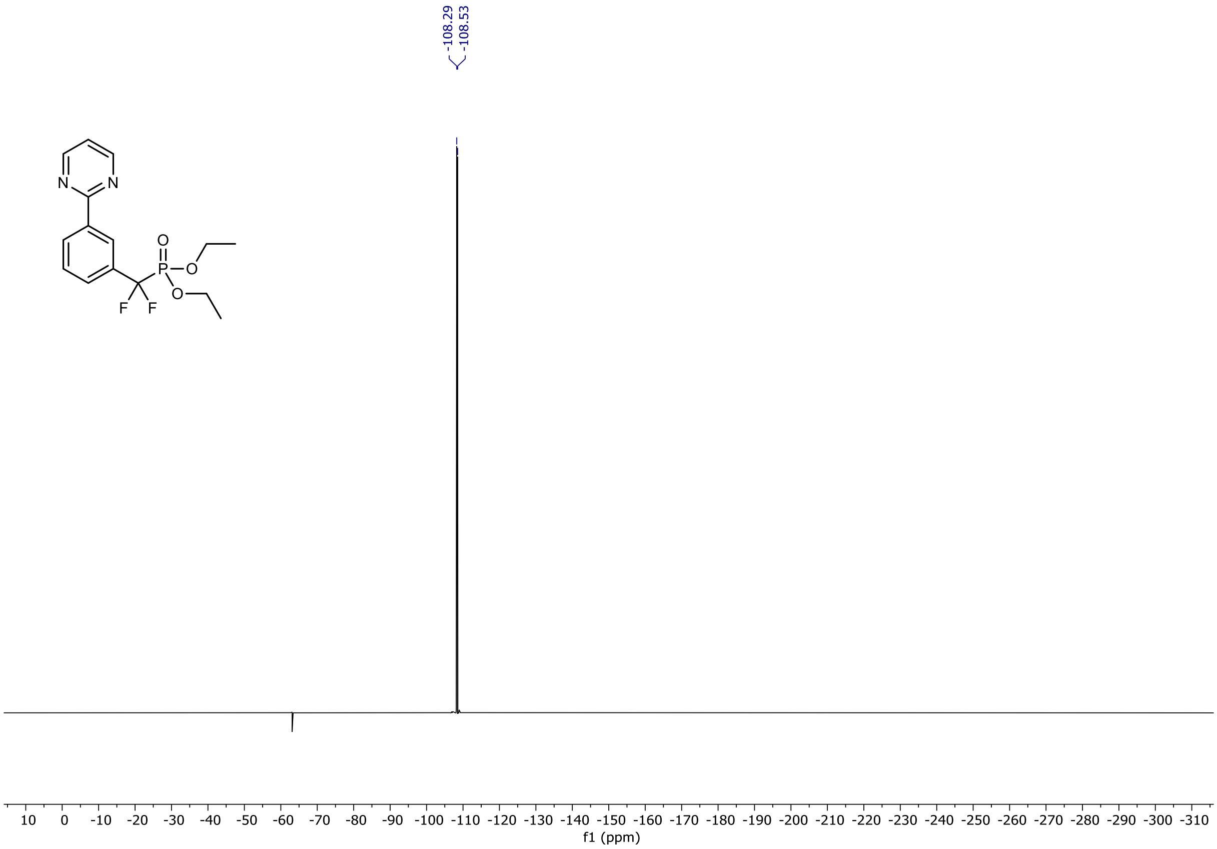

Supplementary Fig. 96. ^{19}F NMR spectra (471 MHz, $CDCl_3$, 298 K) of compound **3j**.

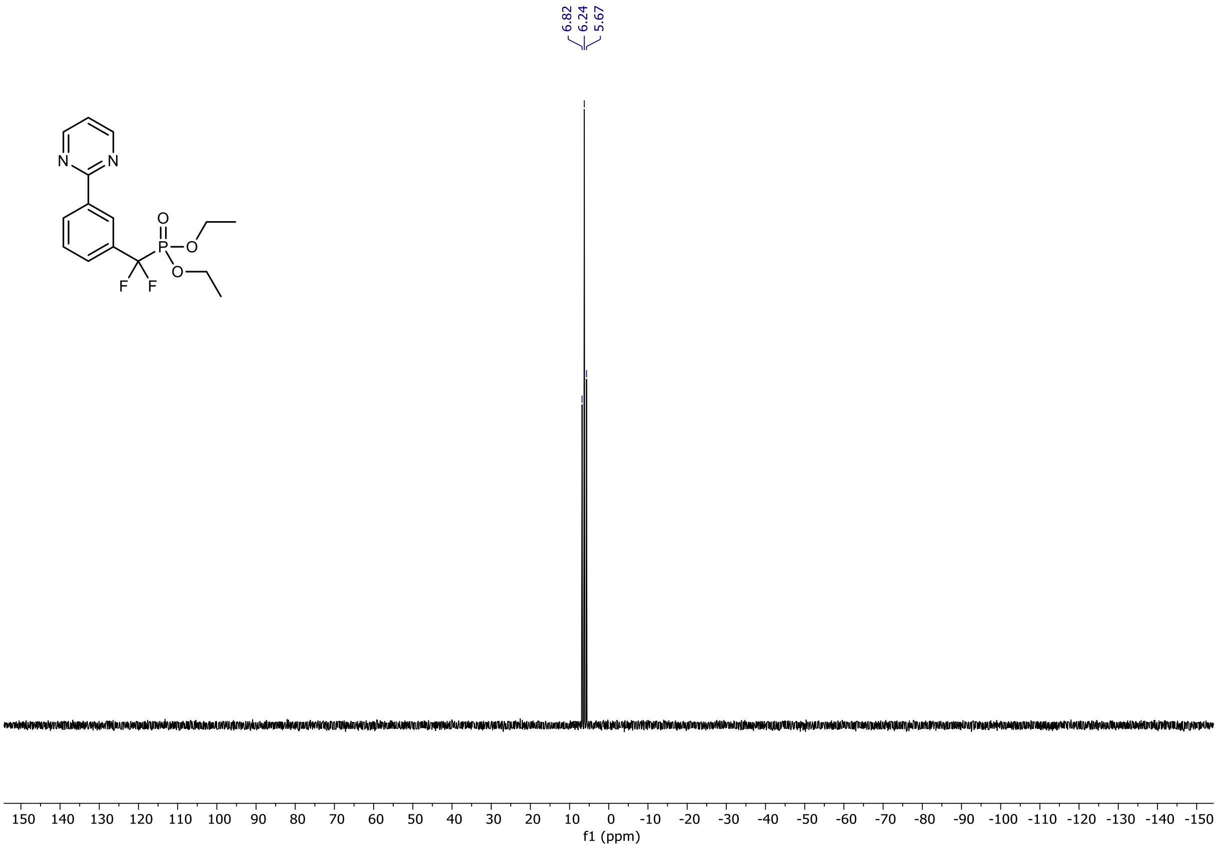

Supplementary Fig. 97. ^{31}P NMR spectra (203 MHz, $CDCl_3$, 298 K) of compound **3j**.

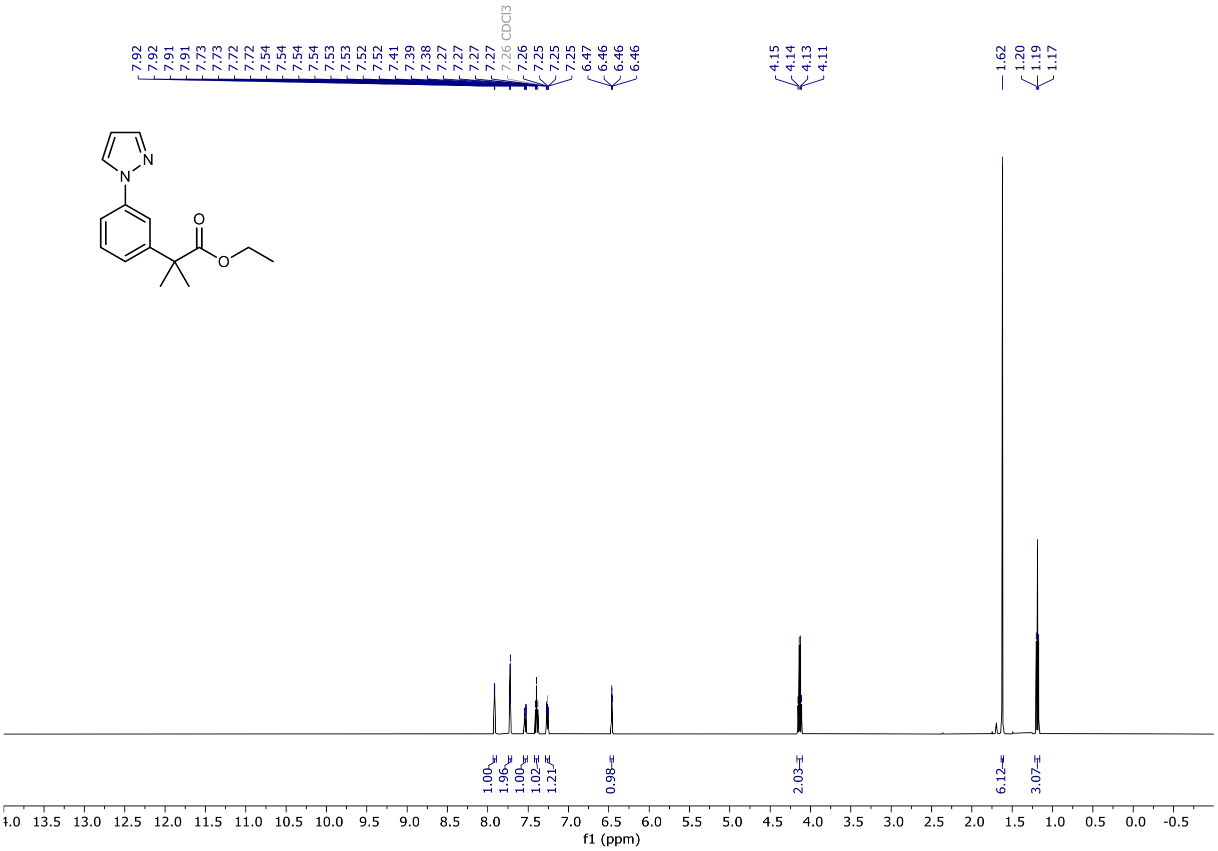

Supplementary Fig. 98. ^{1}H NMR spectra (500 MHz, $CDCl_3$, 298 K) of compound **3k**.

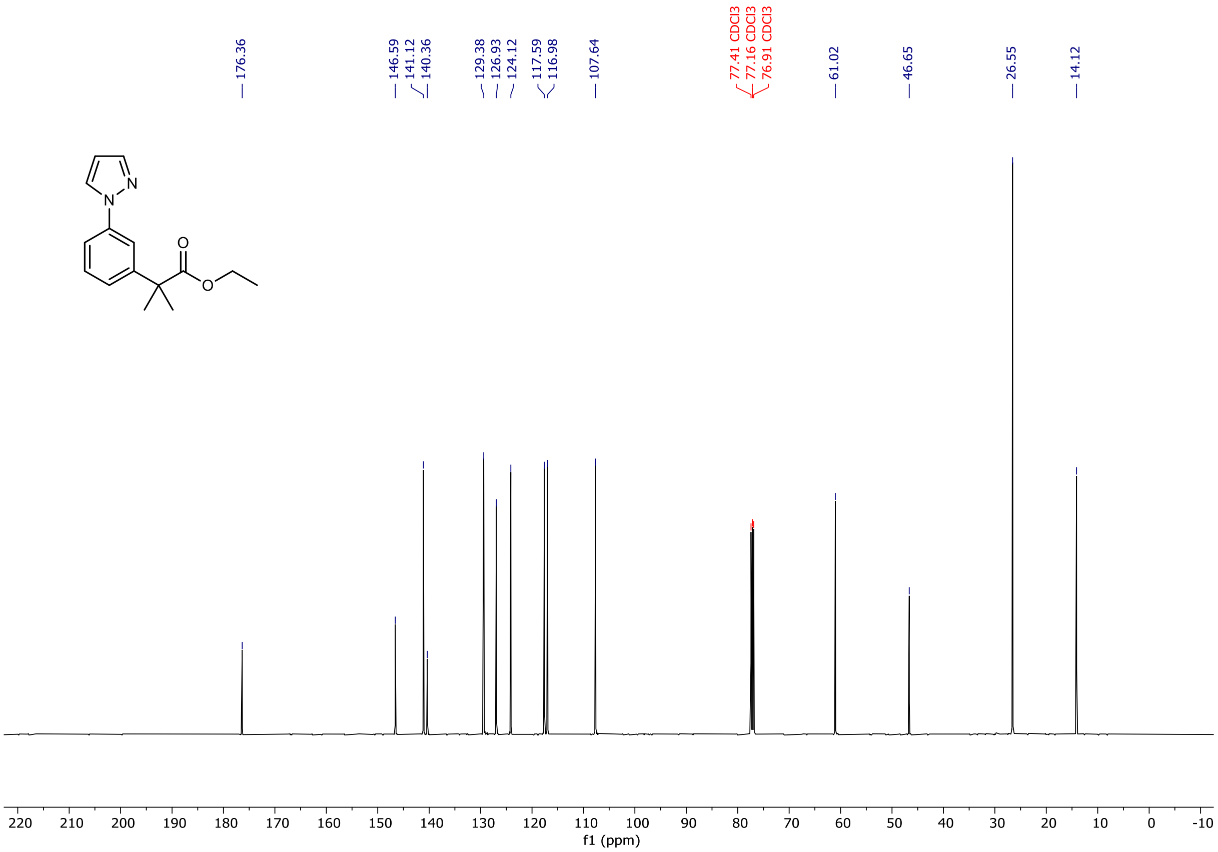

Supplementary Fig. 99. ^{13}C NMR spectra (126 MHz, $CDCl_3$, 298 K) of compound **3k**.

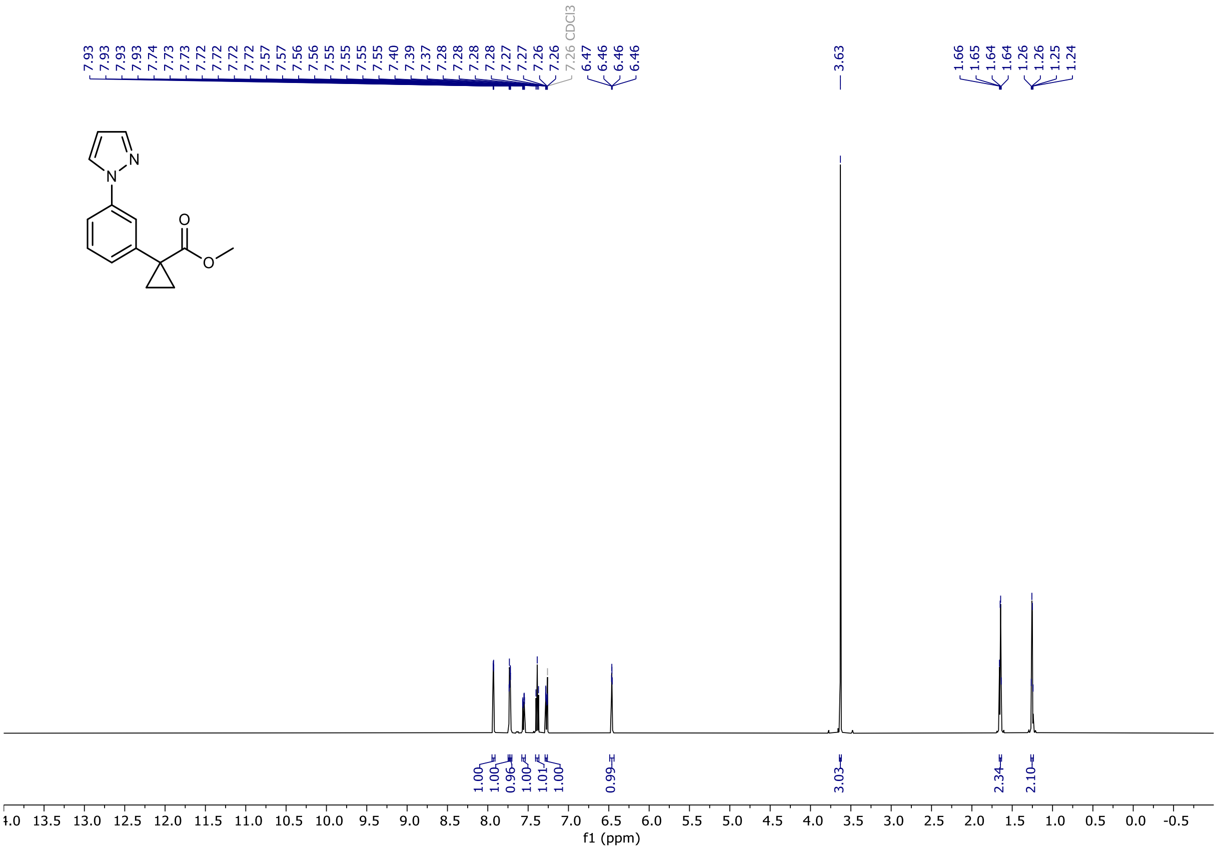

Supplementary Fig. 100. ^{1}H NMR spectra (500 MHz, $CDCl_3$, 298 K) of compound **3l**.

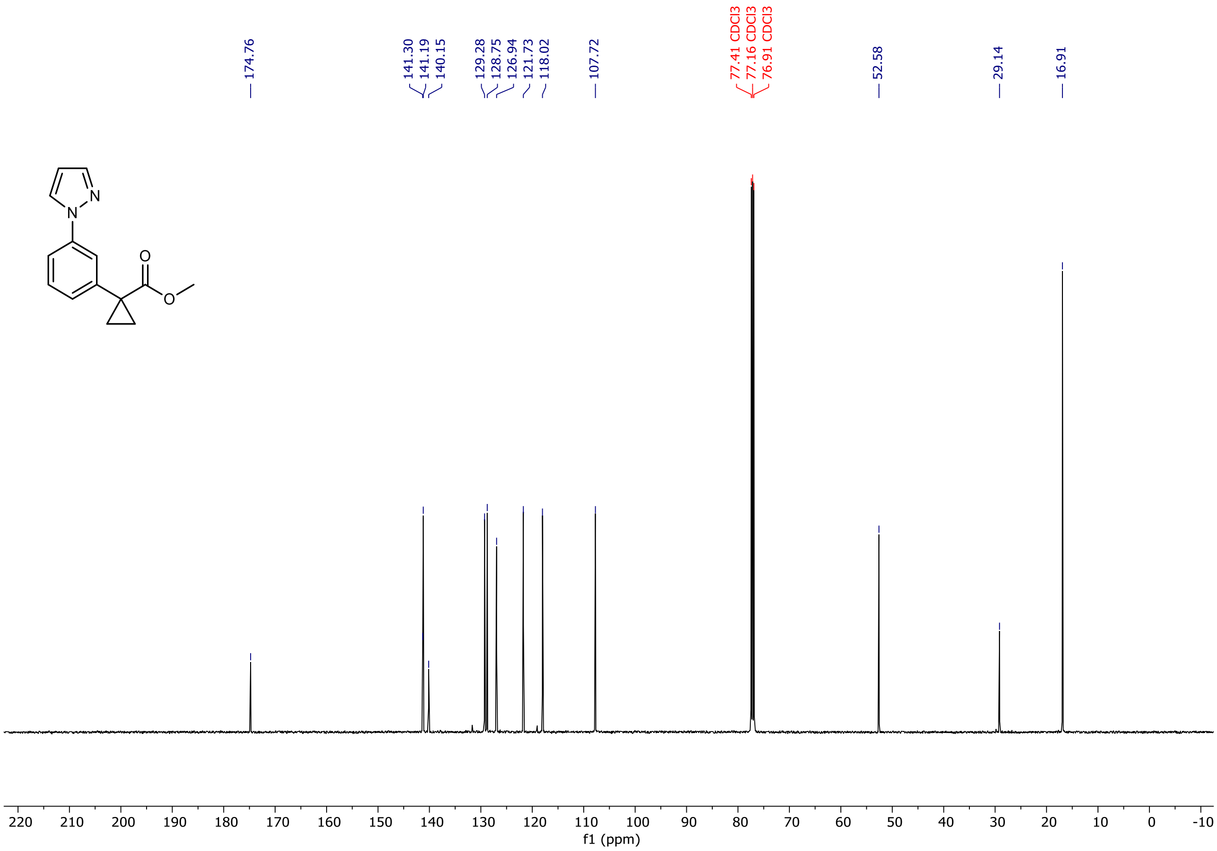

Supplementary Fig. 101. ^{13}C NMR spectra (126 MHz, $CDCl_3$, 298 K) of compound **3l**.

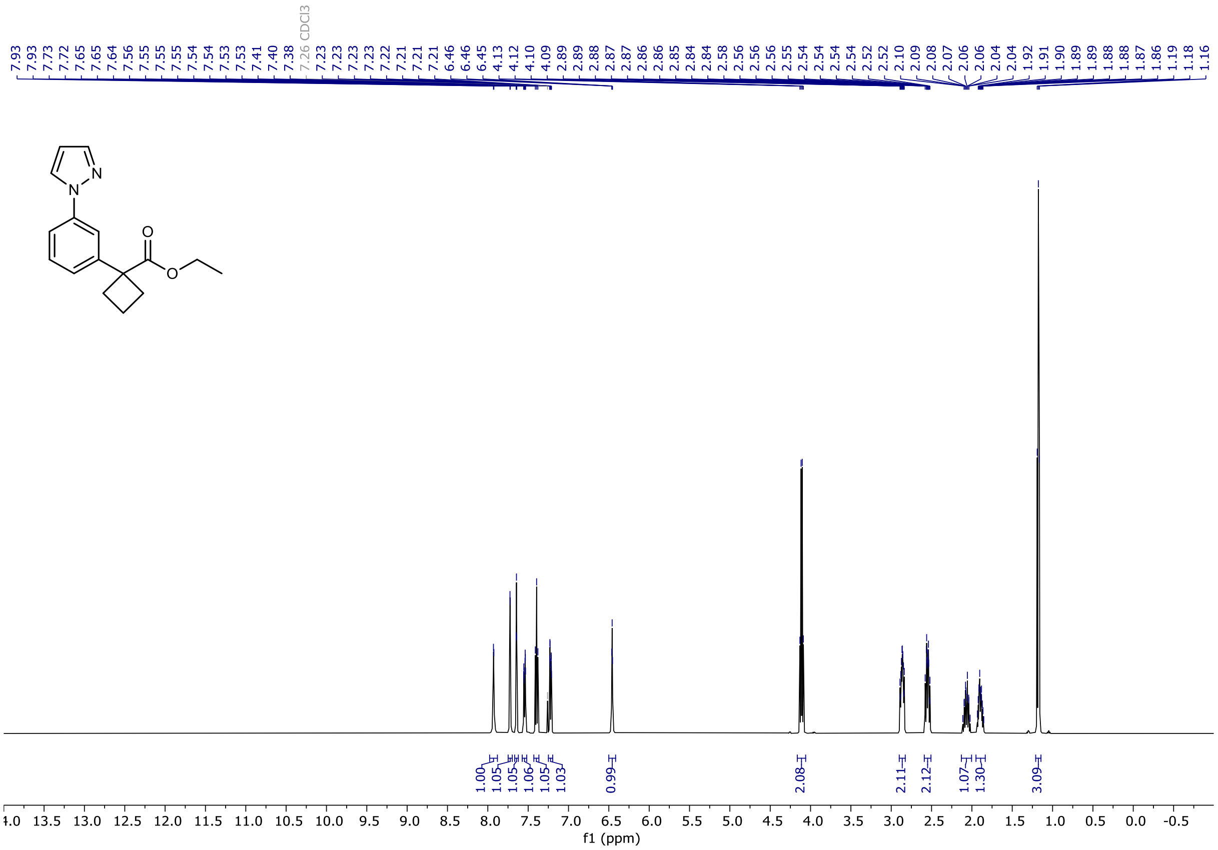

Supplementary Fig. 102. ^{1}H NMR spectra (500 MHz, $CDCl_3$, 298 K) of compound **3m**.

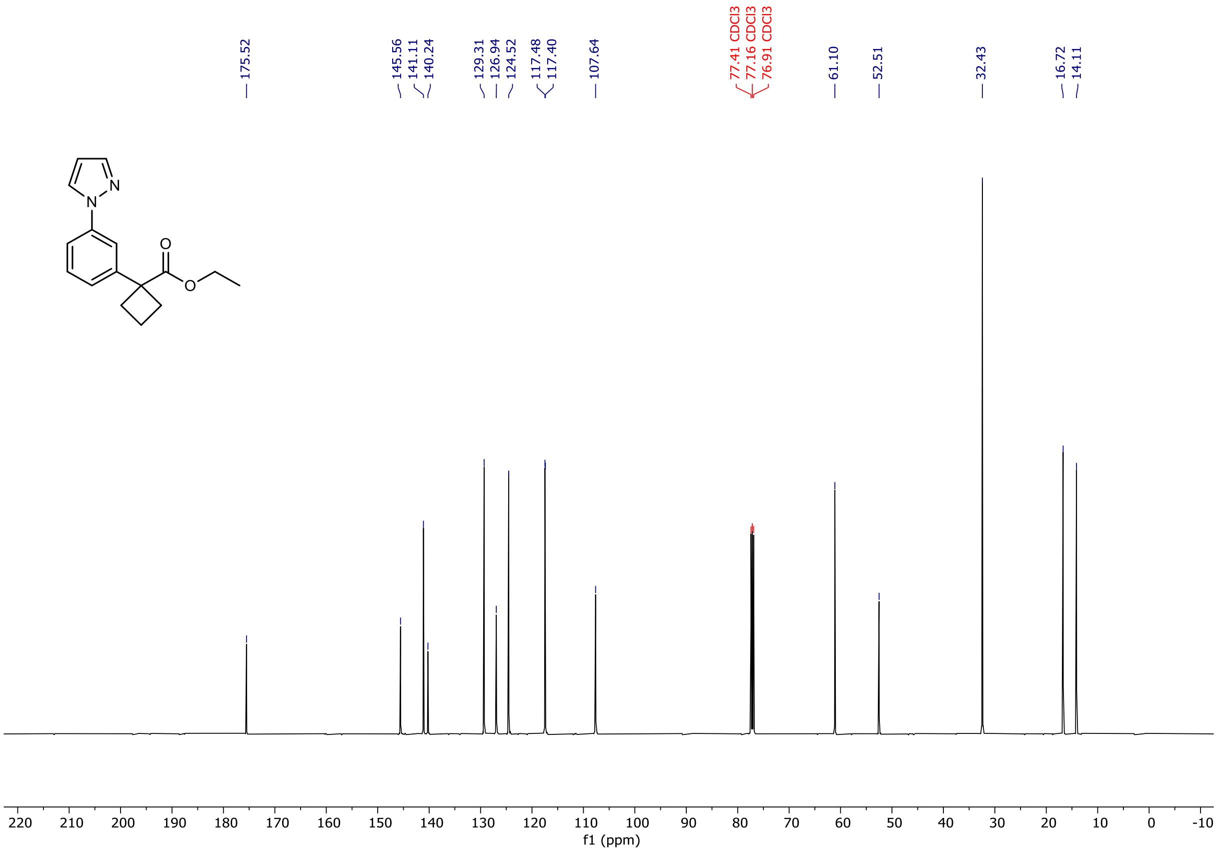

Supplementary Fig. 103. ^{13}C NMR spectra (126 MHz, $CDCl_3$, 298 K) of compound **3m**.

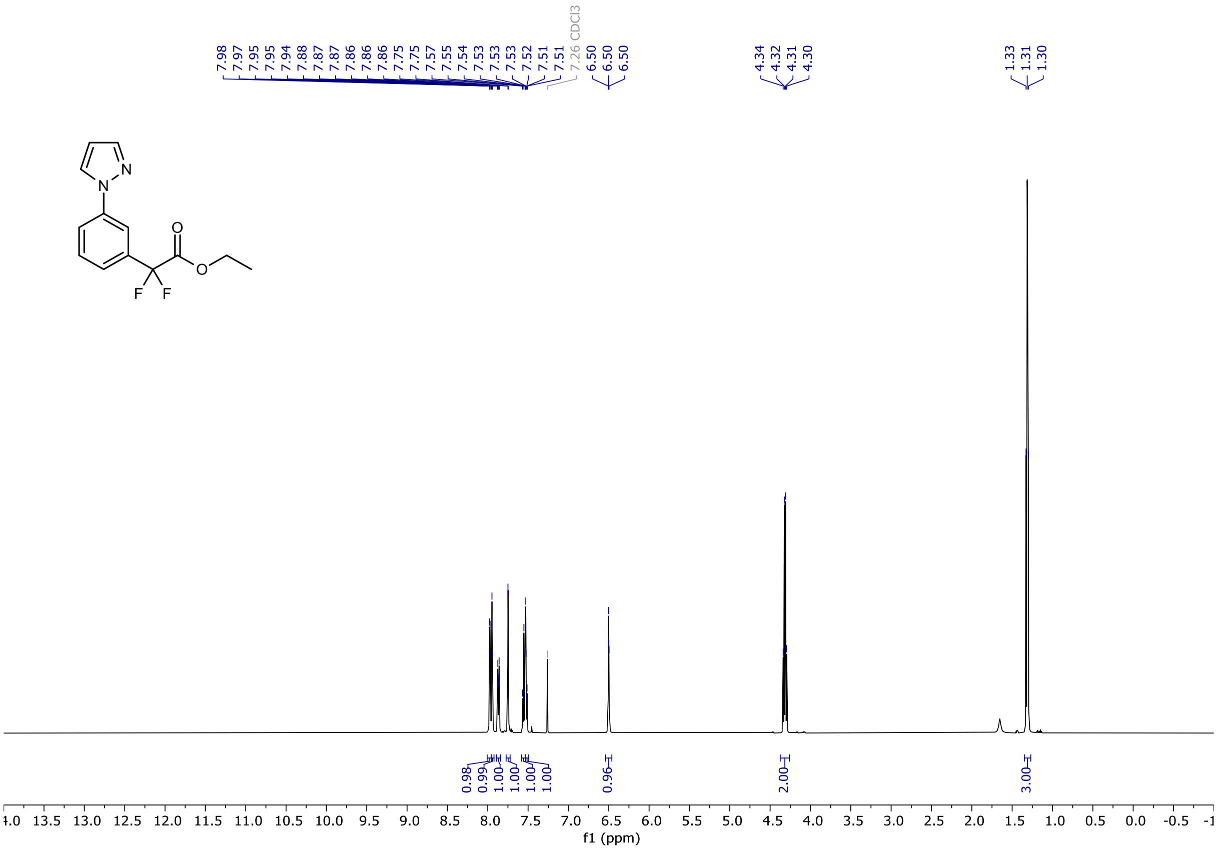

Supplementary Fig. 104. ^{1}H NMR spectra (500 MHz, $CDCl_3$, 298 K) of compound **3n**.

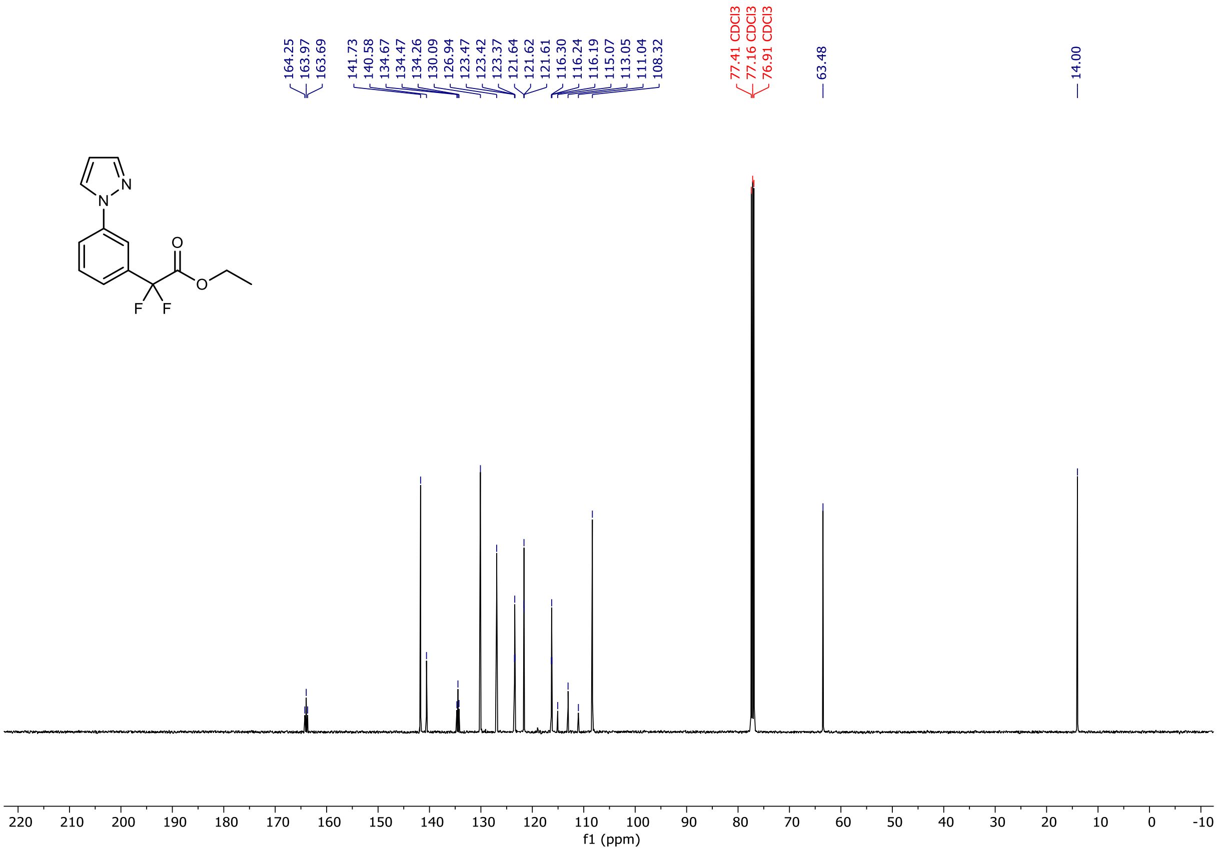

Supplementary Fig. 105. ^{13}C NMR spectra (126 MHz, $CDCl_3$, 298 K) of compound **3n**.

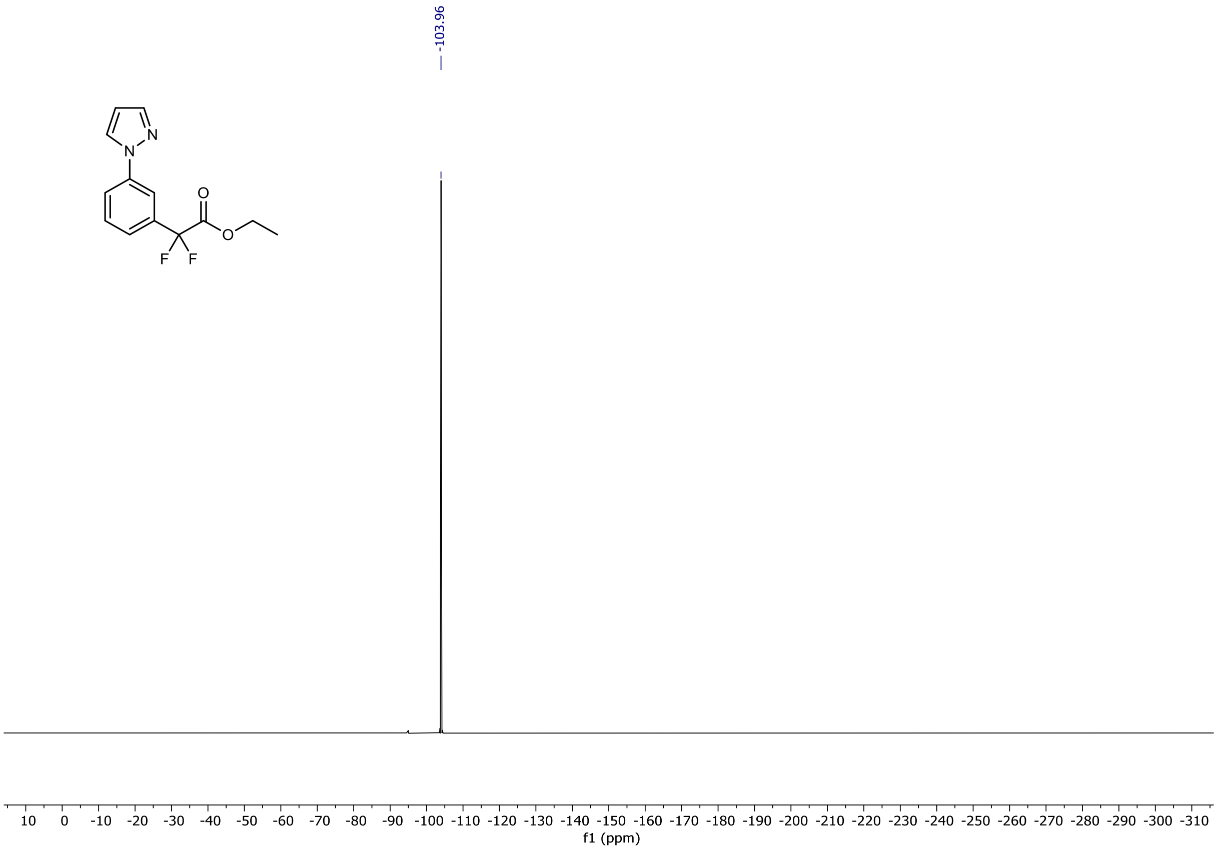

Supplementary Fig. 106. ^{19}F NMR spectra (471 MHz, $CDCl_3$, 298 K) of compound **3n**.

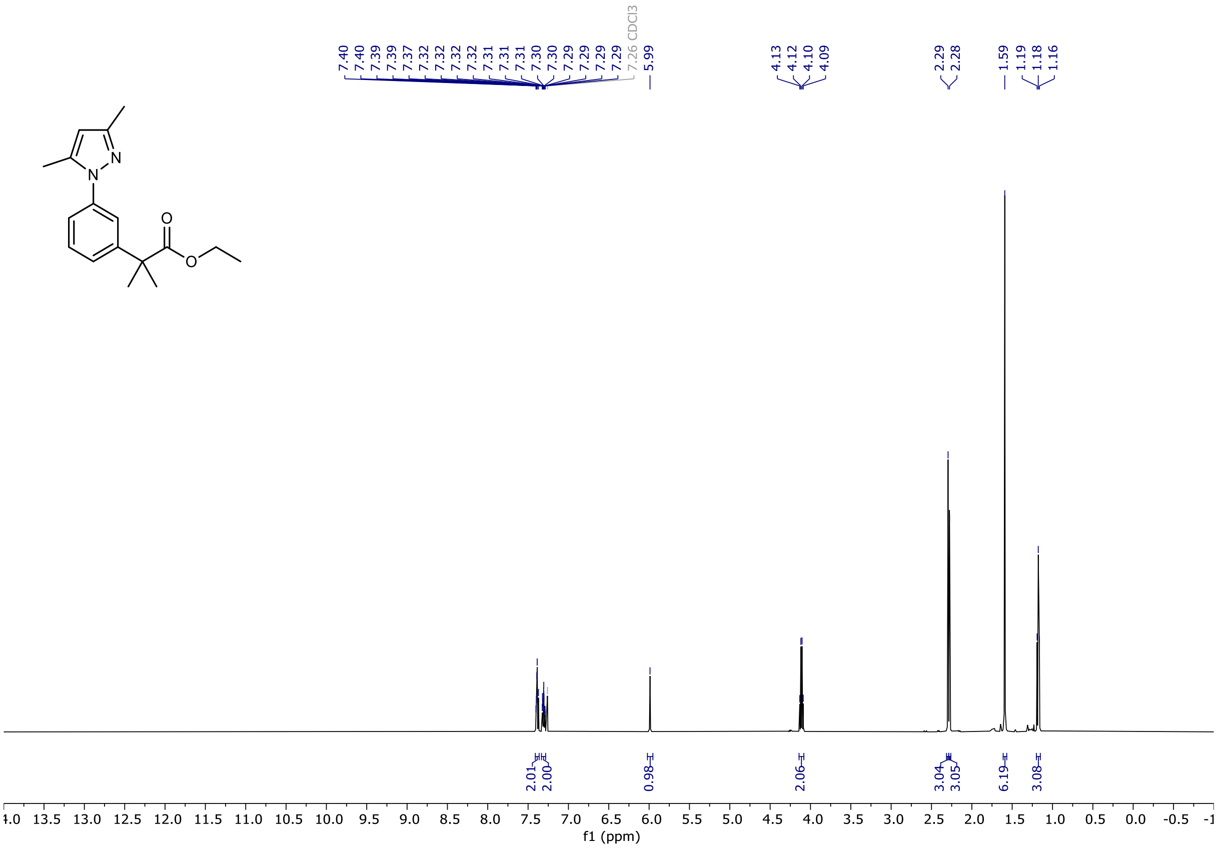

Supplementary Fig. 107. ^{1}H NMR spectra (500 MHz, $CDCl_3$, 298 K) of compound **3o**.

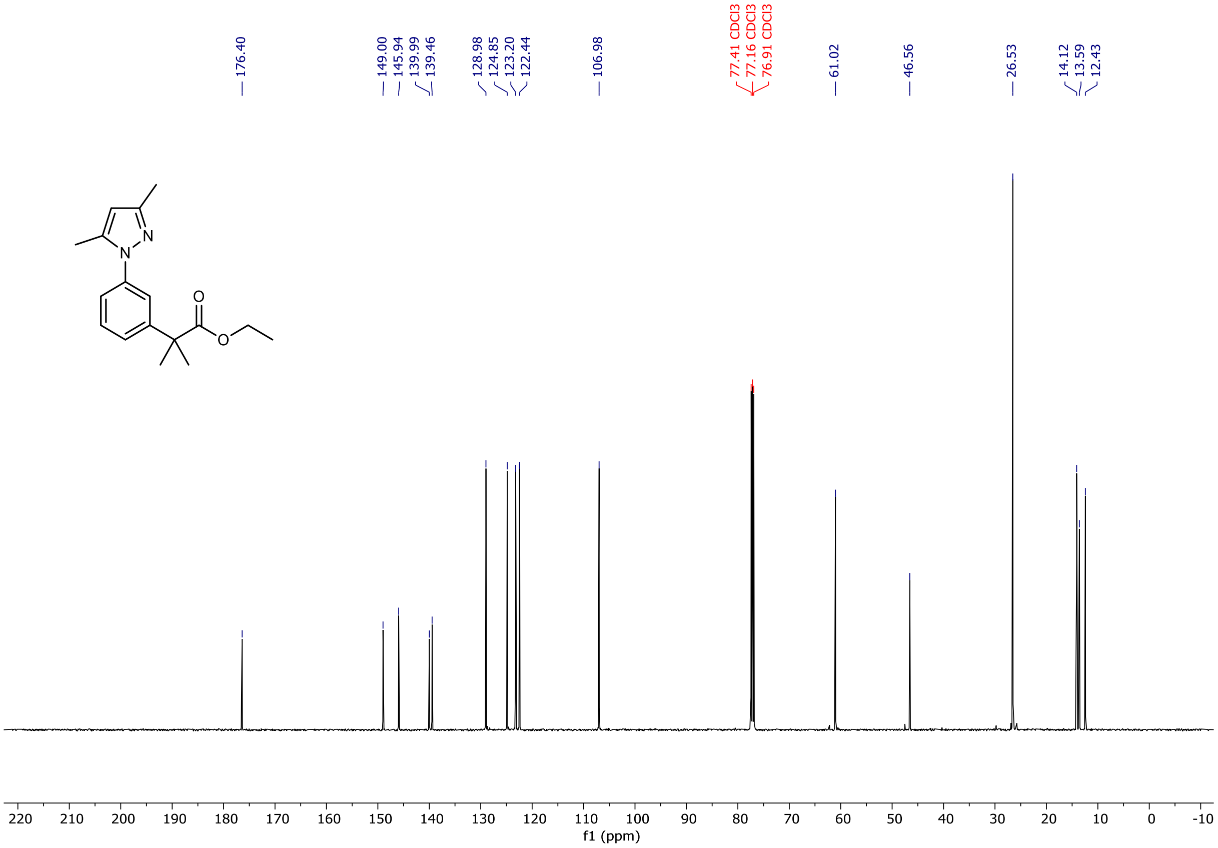

Supplementary Fig. 108. ^{13}C NMR spectra (126 MHz, $CDCl_3$, 298 K) of compound **3o**.

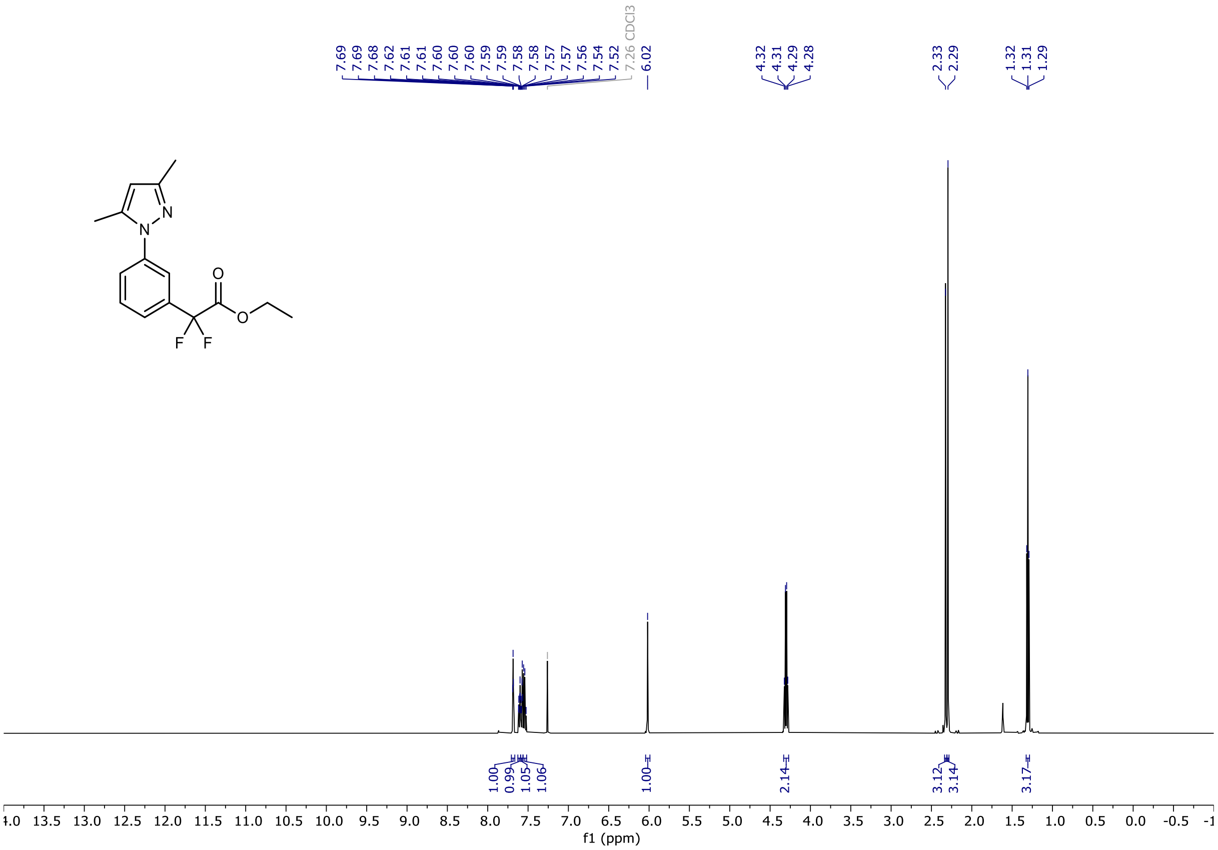

Supplementary Fig. 109. ^{1}H NMR spectra (500 MHz, $CDCl_3$, 298 K) of compound **3p**.

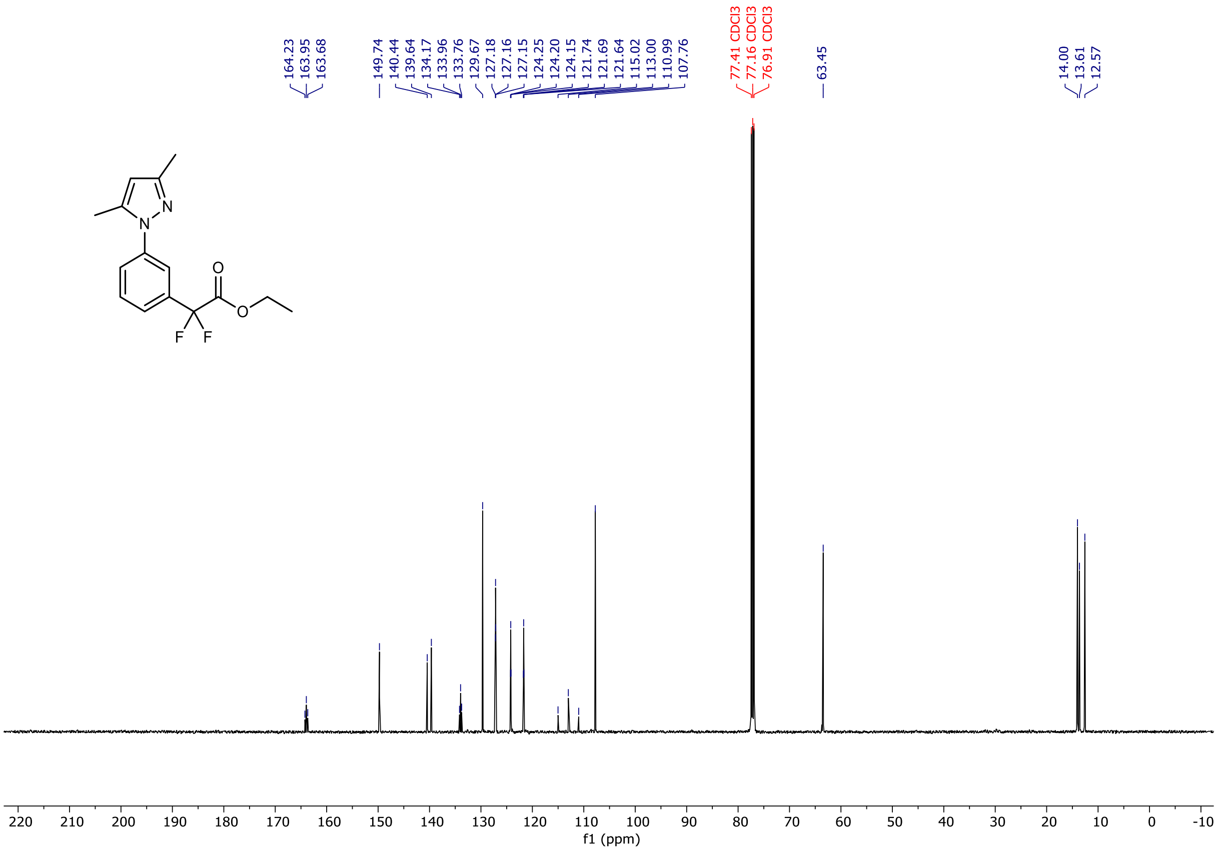

Supplementary Fig. 110. ^{13}C NMR spectra (126 MHz, $CDCl_3$, 298 K) of compound **3p**.

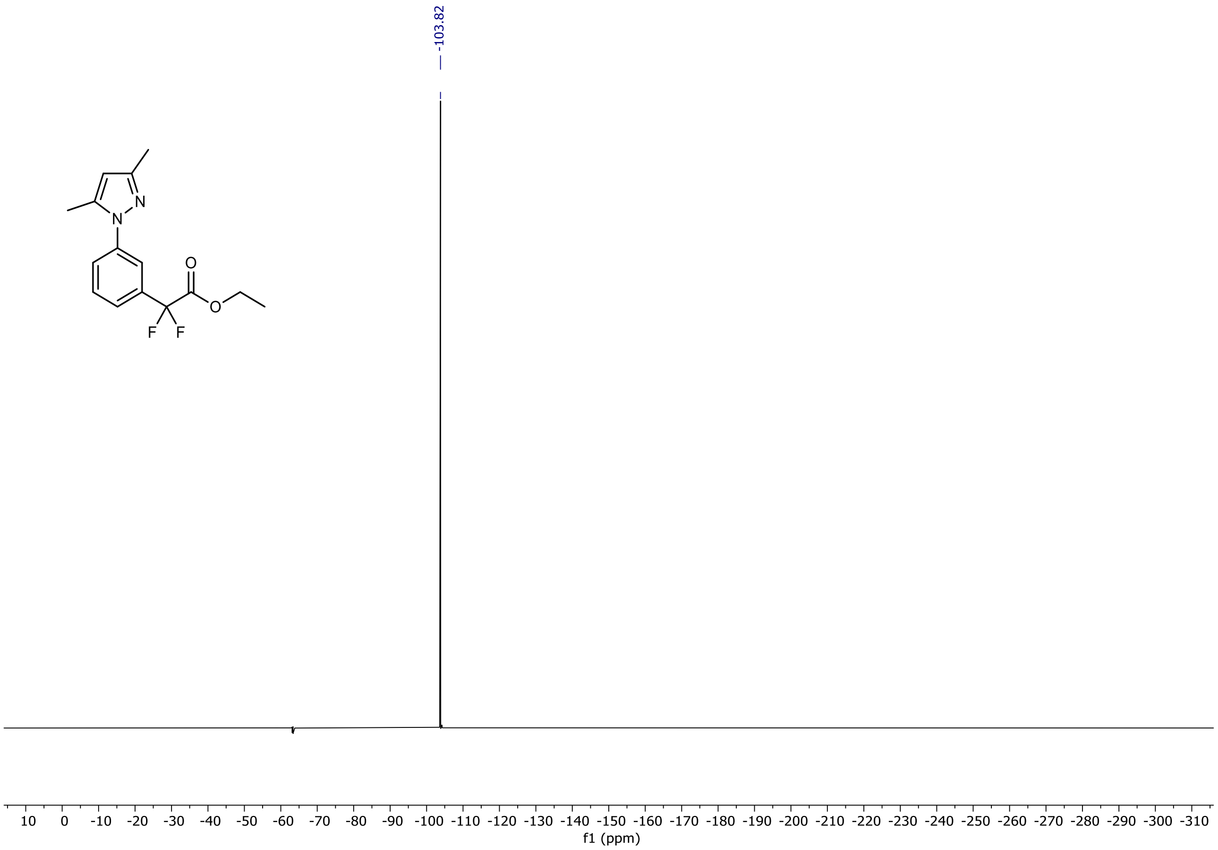

Supplementary Fig. 111. ^{19}F NMR spectra (471 MHz, $CDCl_3$, 298 K) of compound **3p**.

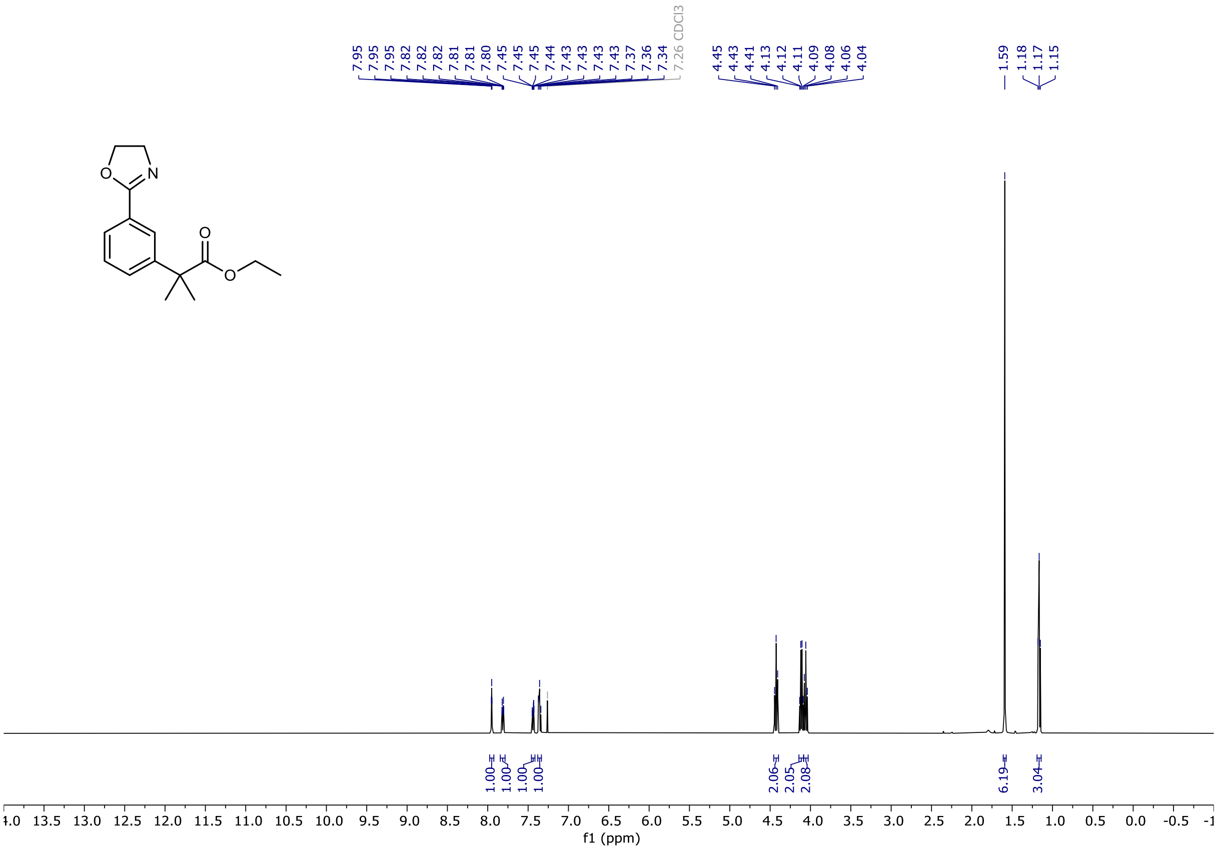

Supplementary Fig. 112. ^{1}H NMR spectra (500 MHz, $CDCl_3$, 298 K) of compound **3q**.

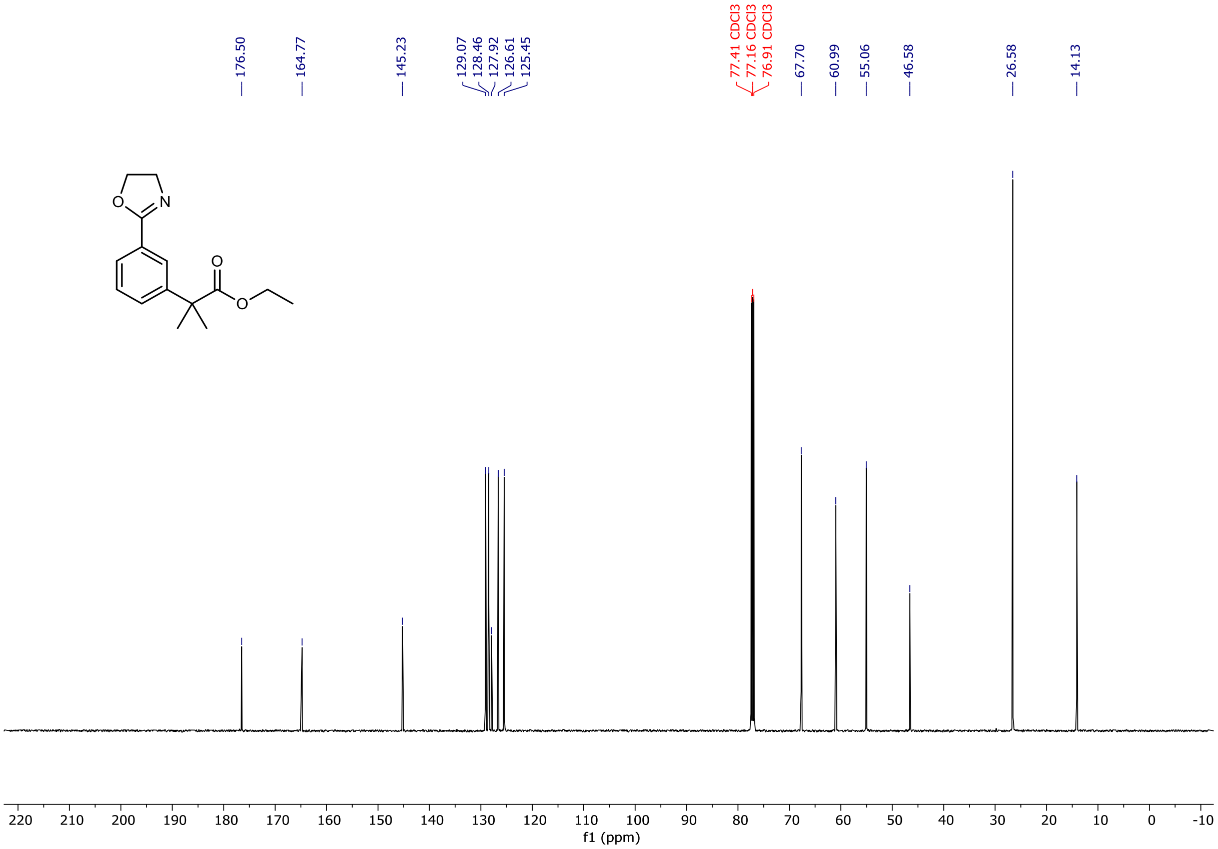

Supplementary Fig. 113. ^{13}C NMR spectra (126 MHz, $CDCl_3$, 298 K) of compound **3q**.

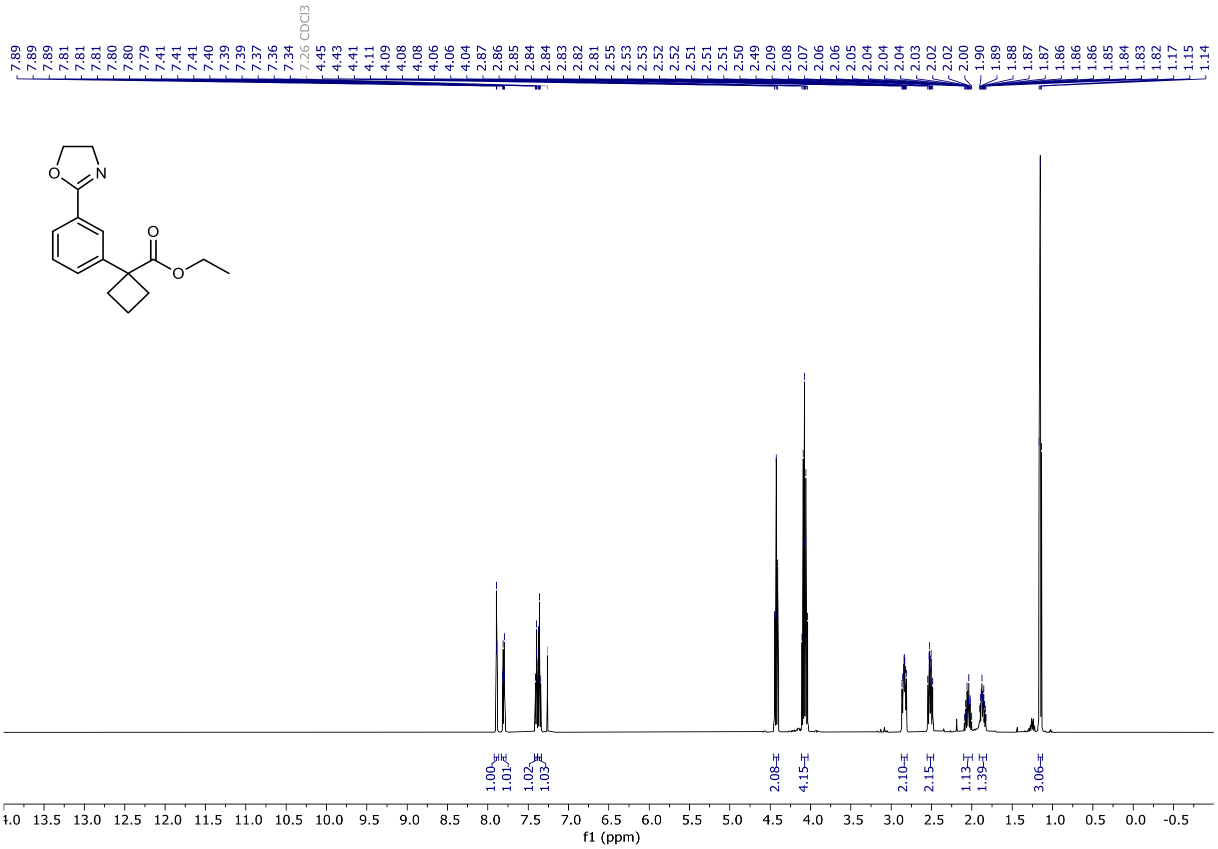

Supplementary Fig. 114. ^{1}H NMR spectra (500 MHz, $CDCl_3$, 298 K) of compound **3s**.

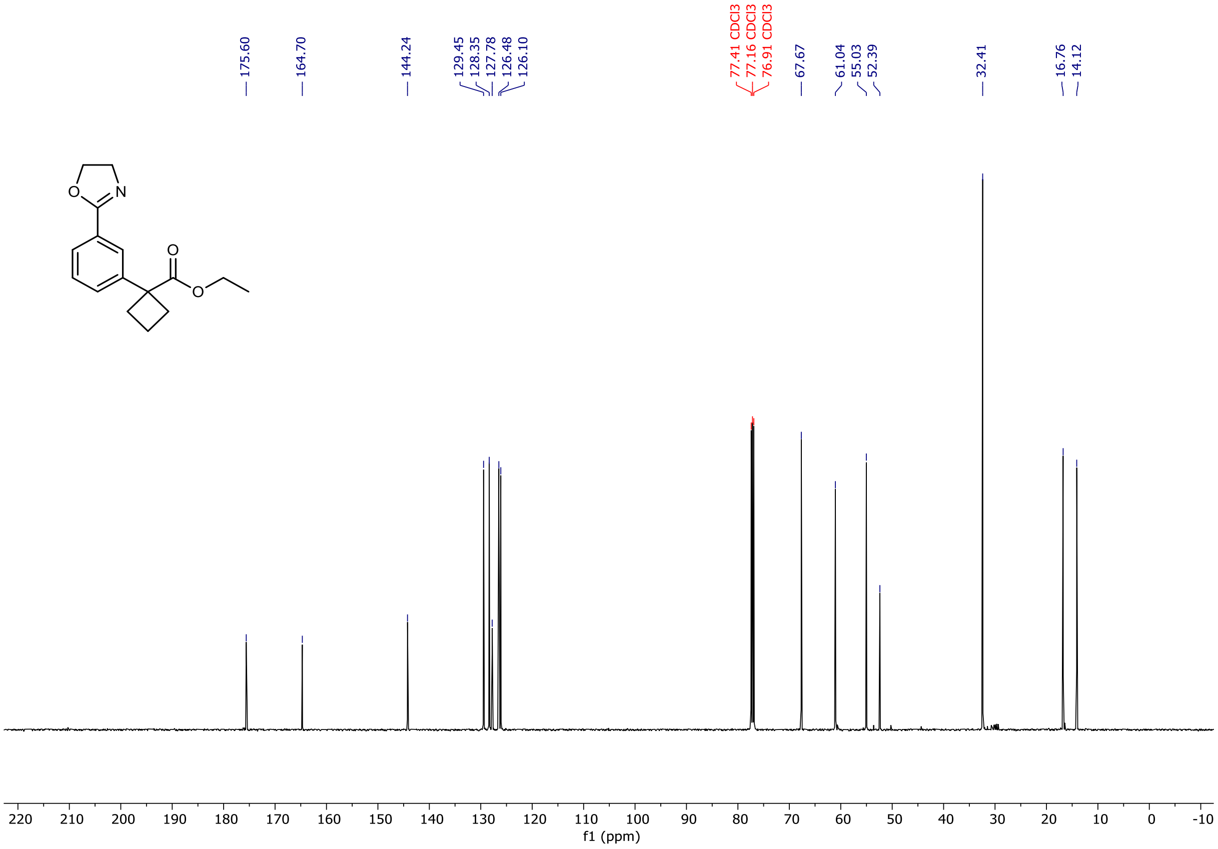

Supplementary Fig. 115. ^{13}C NMR spectra (126 MHz, $CDCl_3$, 298 K) of compound **3s**.

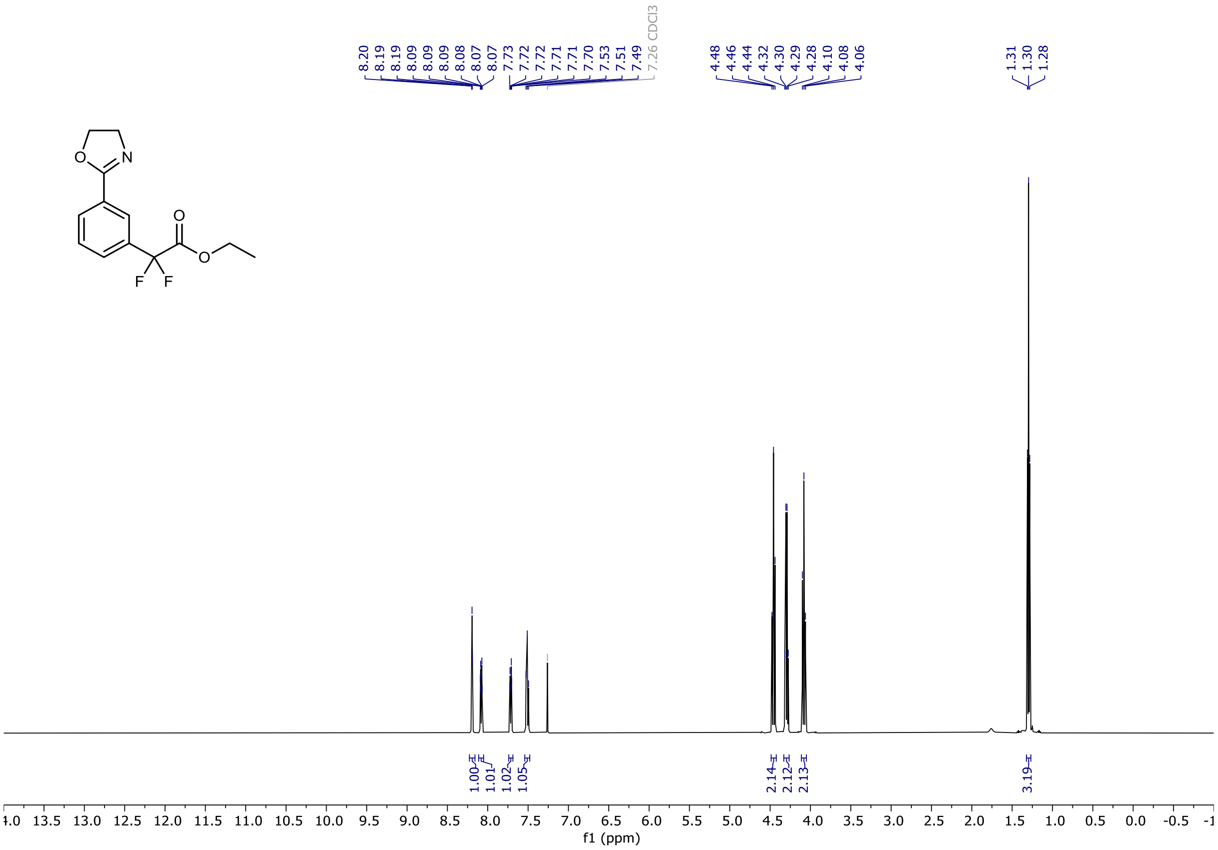

Supplementary Fig. 116. ^{1}H NMR spectra (500 MHz, $CDCl_3$, 298 K) of compound **3t**.

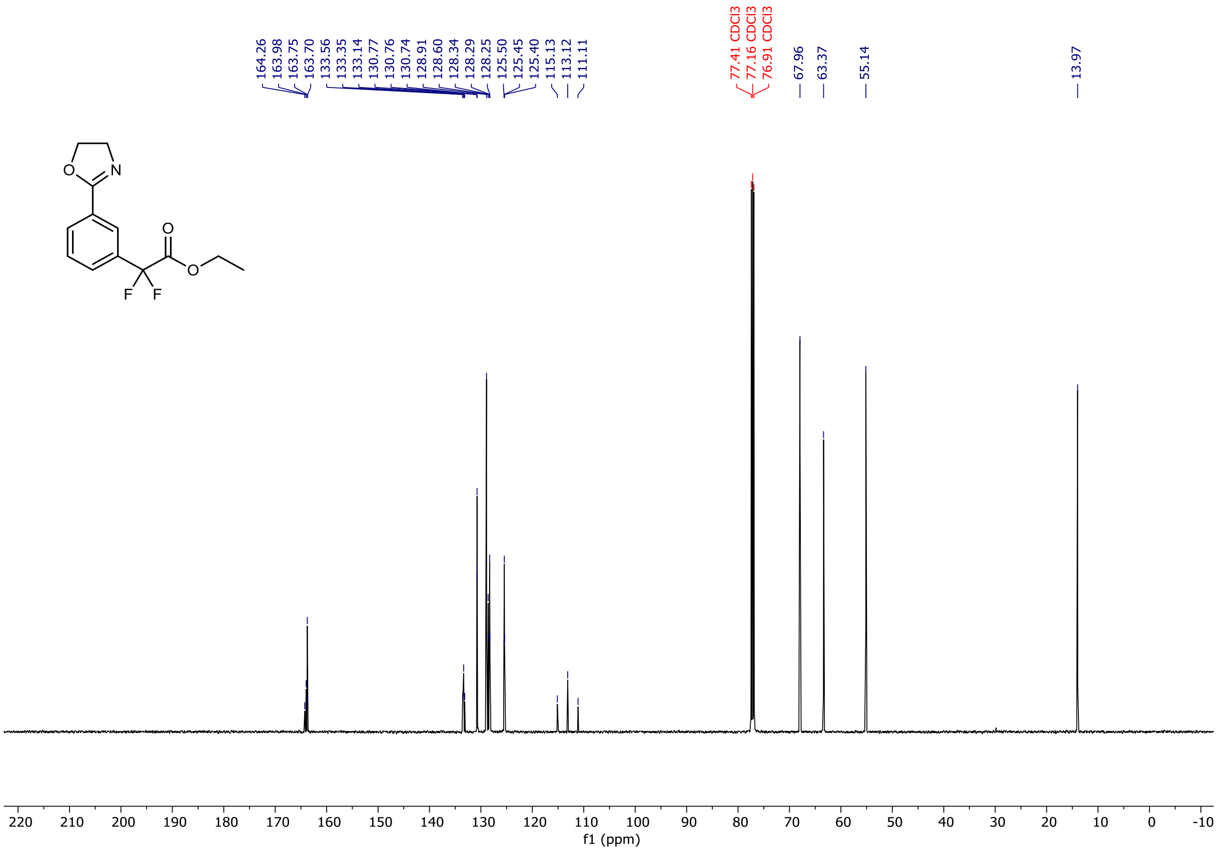

Supplementary Fig. 117. ^{13}C NMR spectra (126 MHz, $CDCl_3$, 298 K) of compound **3t**.

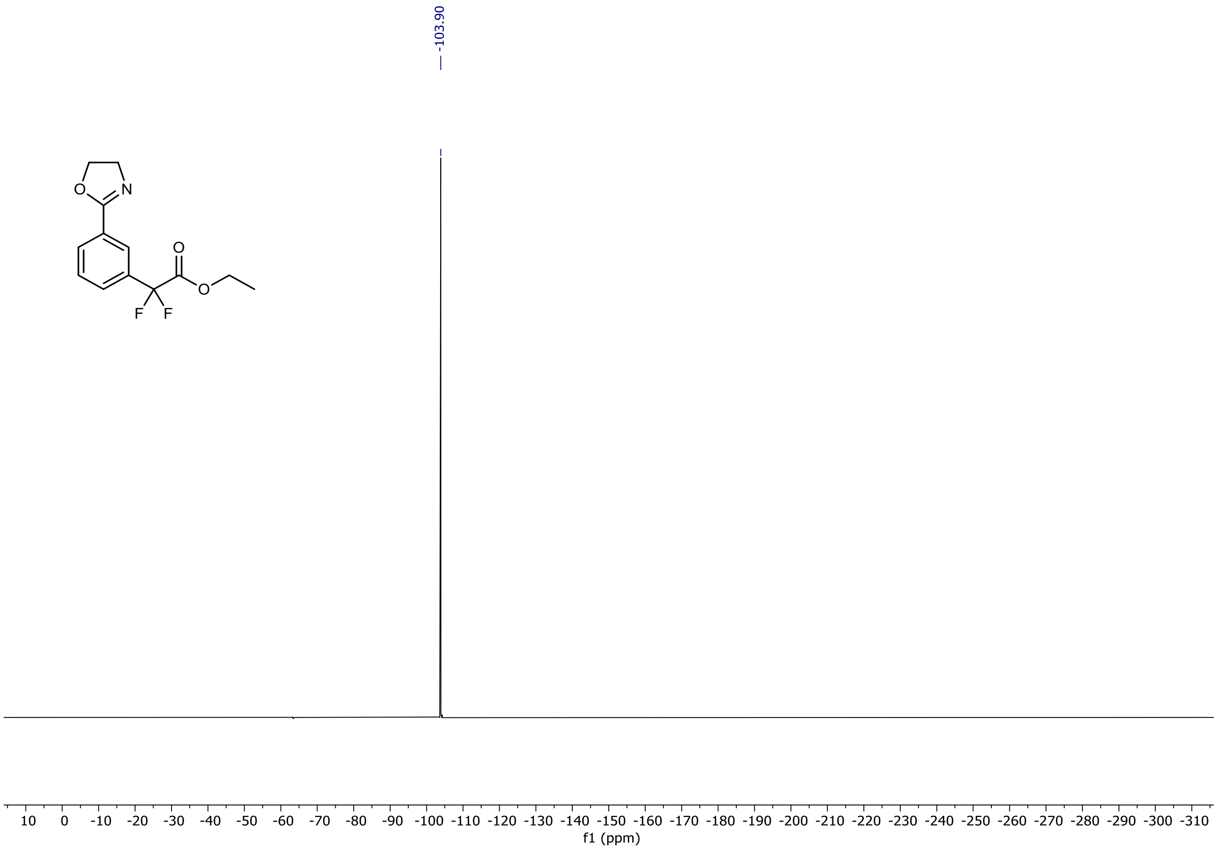

Supplementary Fig. 118. ^{19}F NMR spectra (471 MHz, $CDCl_3$, 298 K) of compound **3t**.

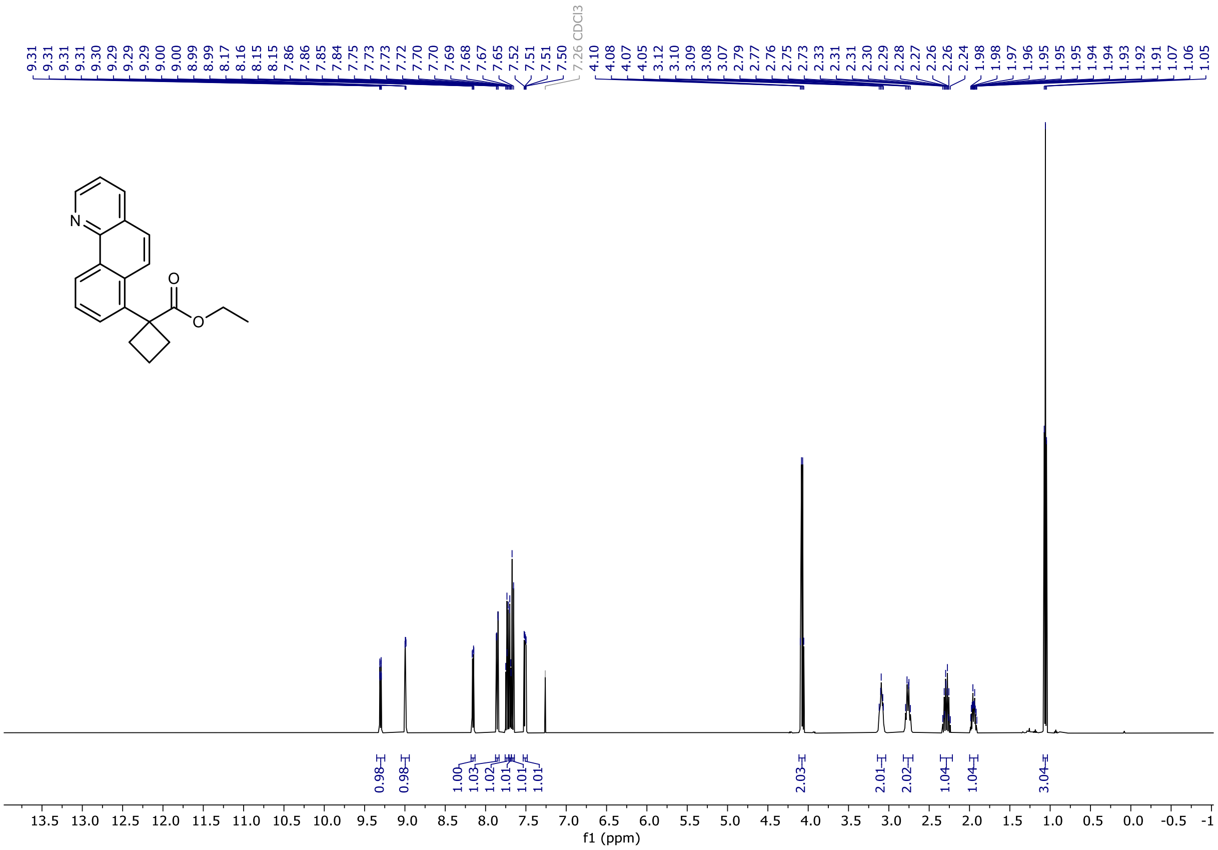

Supplementary Fig. 119. ^{1}H NMR spectra (500 MHz, $CDCl_3$, 298 K) of compound **3u**.

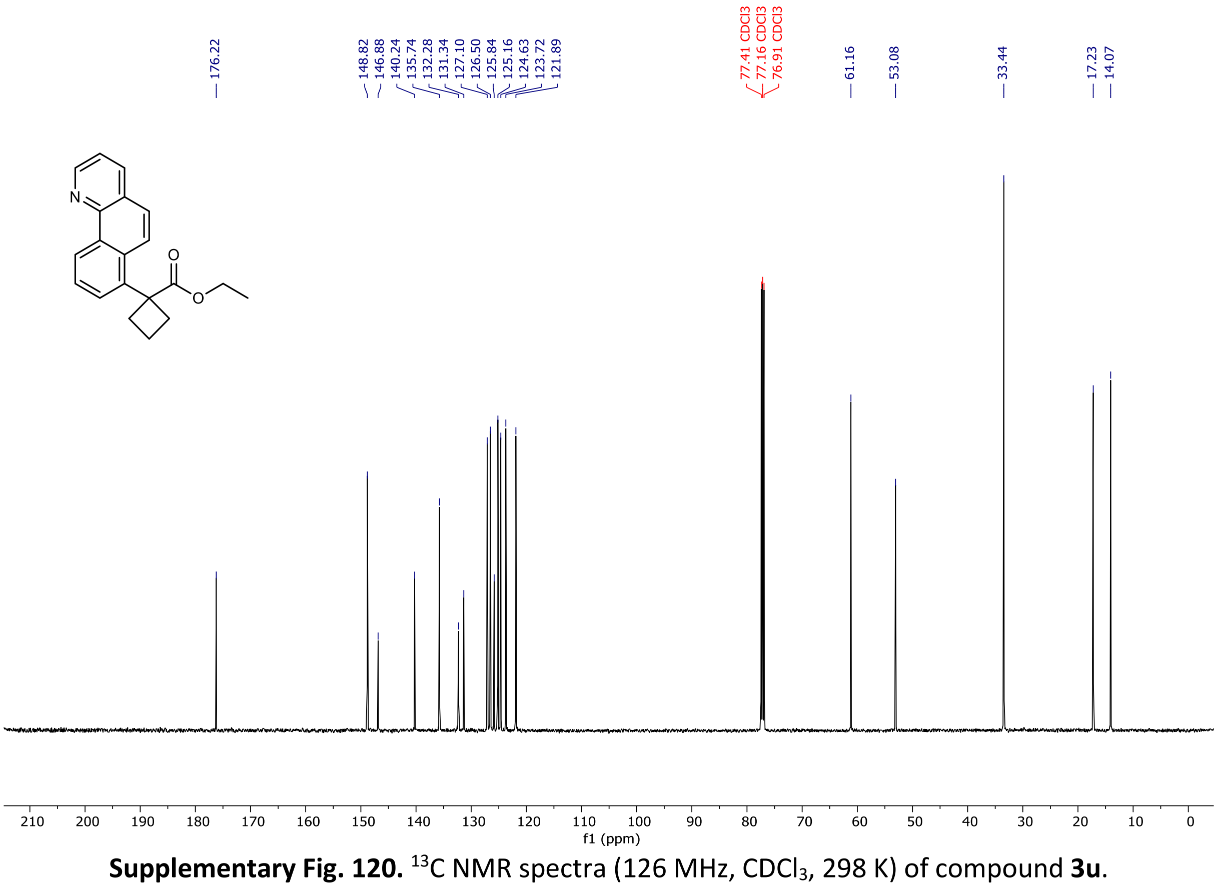

Supplementary Fig. 120. ^{13}C NMR spectra (126 MHz, $CDCl_3$, 298 K) of compound **3u**.

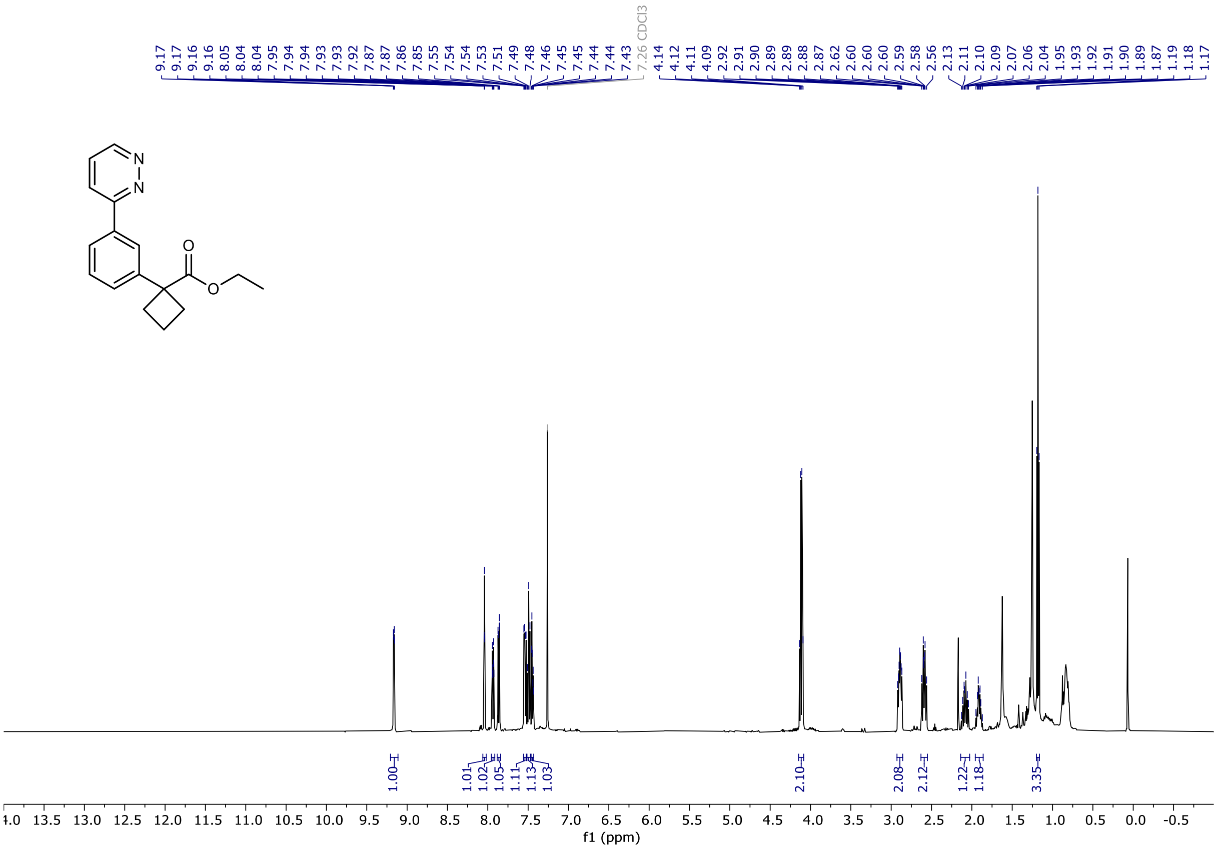

Supplementary Fig. 121. ^{1}H NMR spectra (500 MHz, $CDCl_3$, 298 K) of compound **3v**.

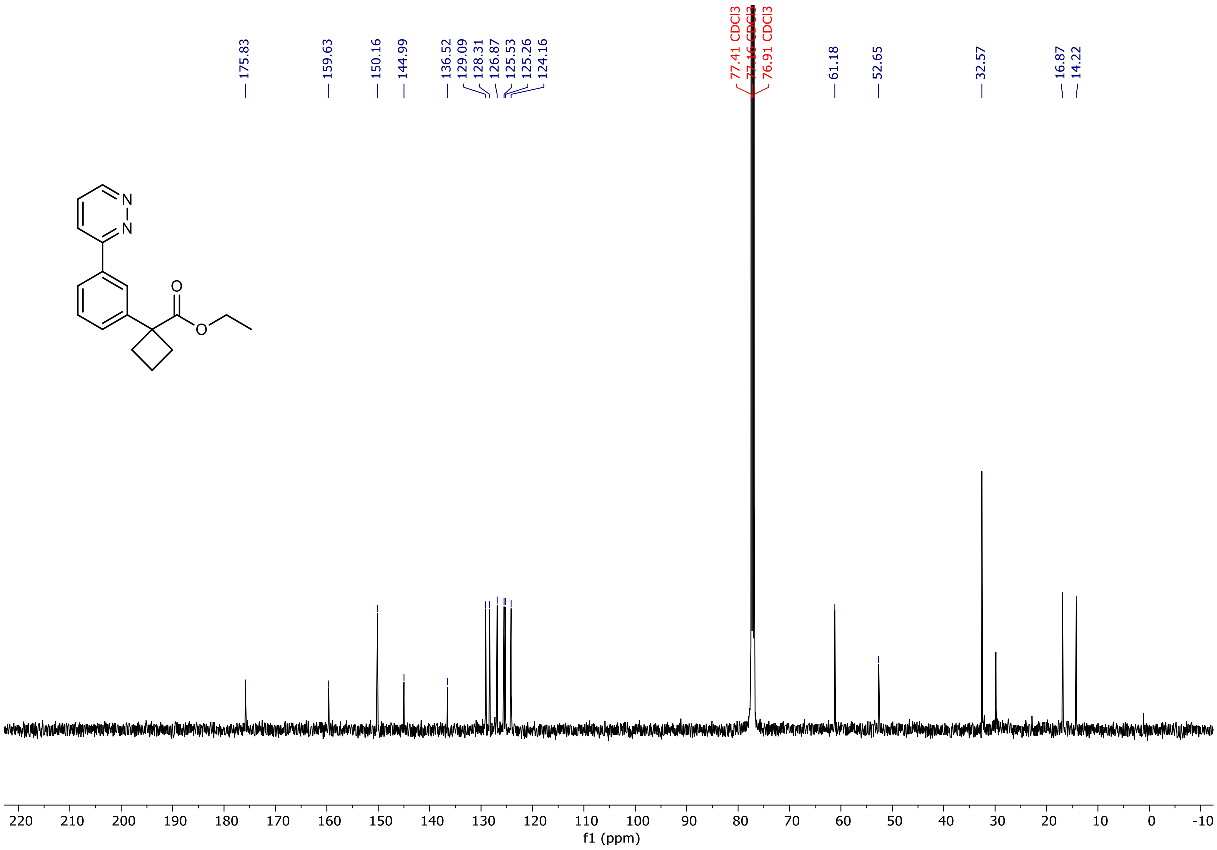

Supplementary Fig. 122. ^{13}C NMR spectra (126 MHz, $CDCl_3$, 298 K) of compound **3v**.

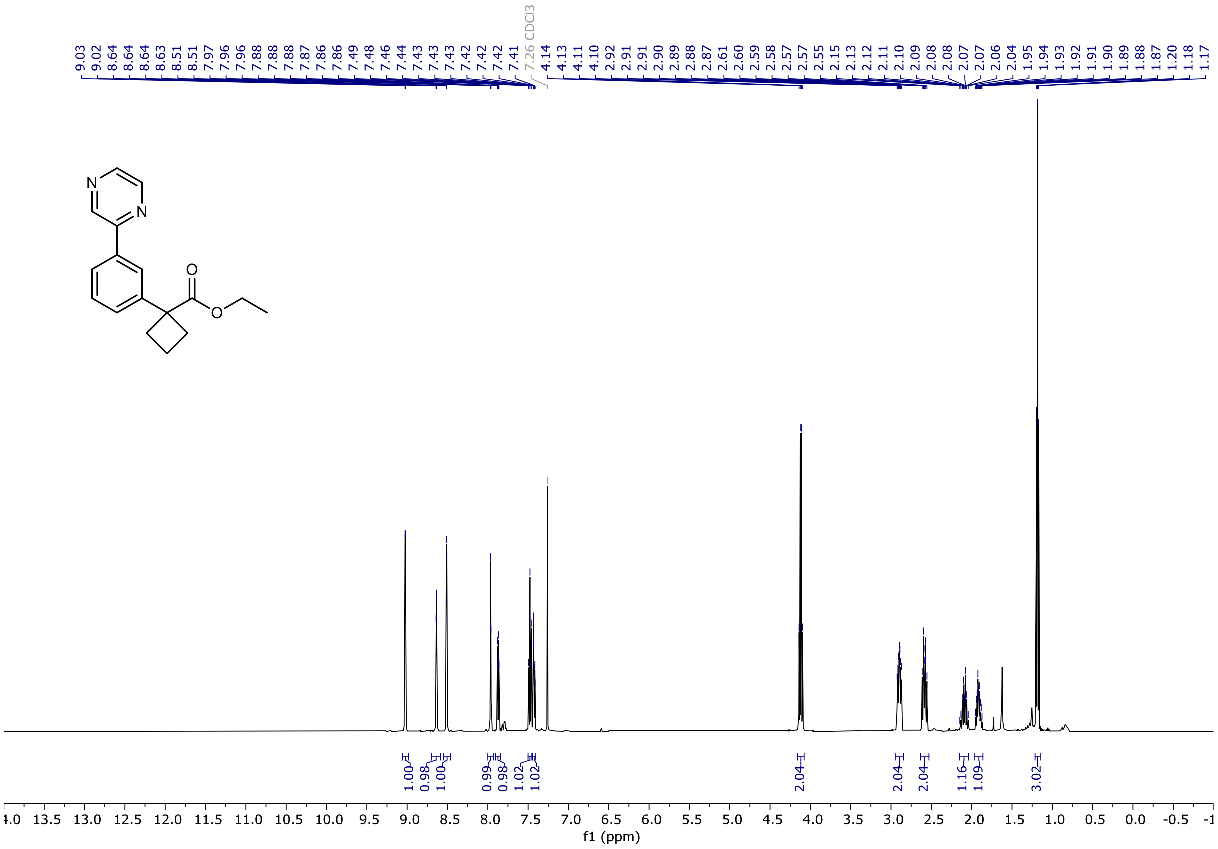

Supplementary Fig. 123. ^{1}H NMR spectra (500 MHz, $CDCl_3$, 298 K) of compound **3w**.

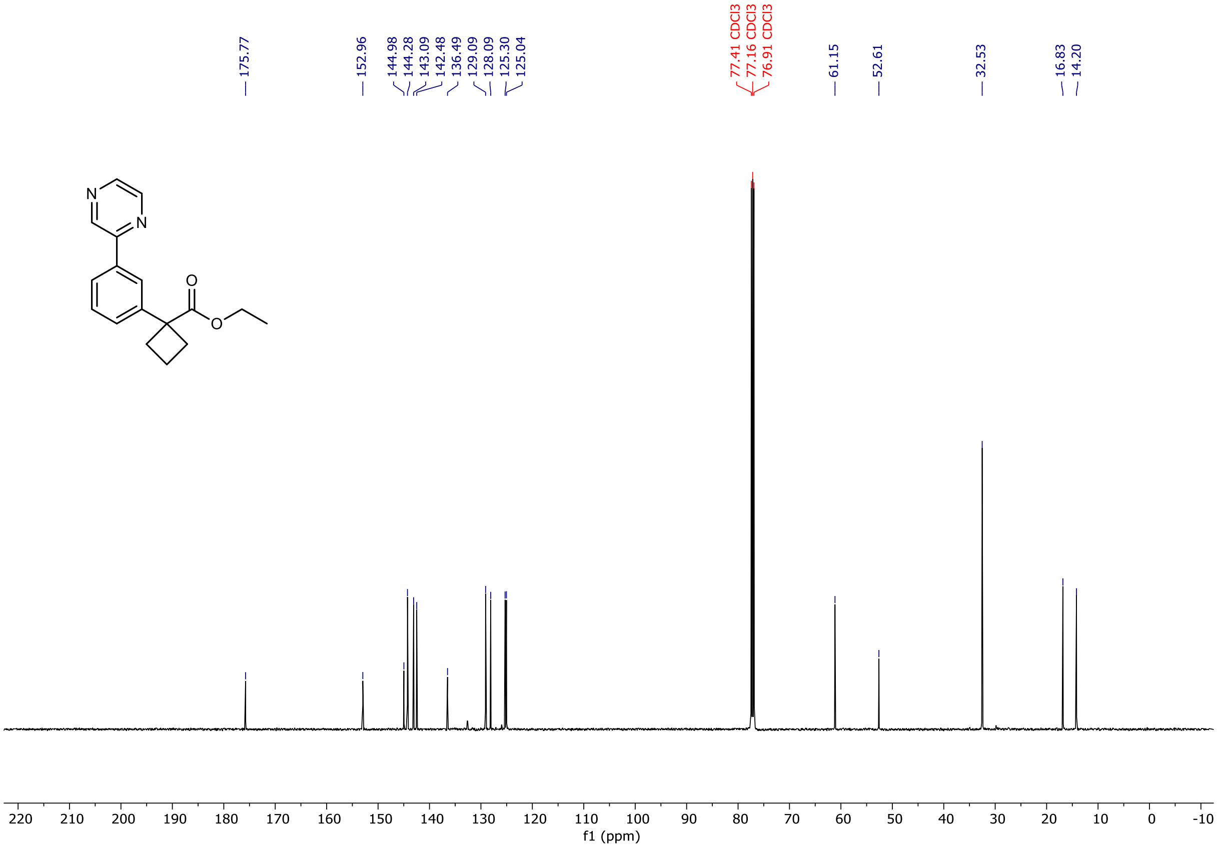

Supplementary Fig. 124. ^{13}C NMR spectra (126 MHz, $CDCl_3$, 298 K) of compound **3w**.

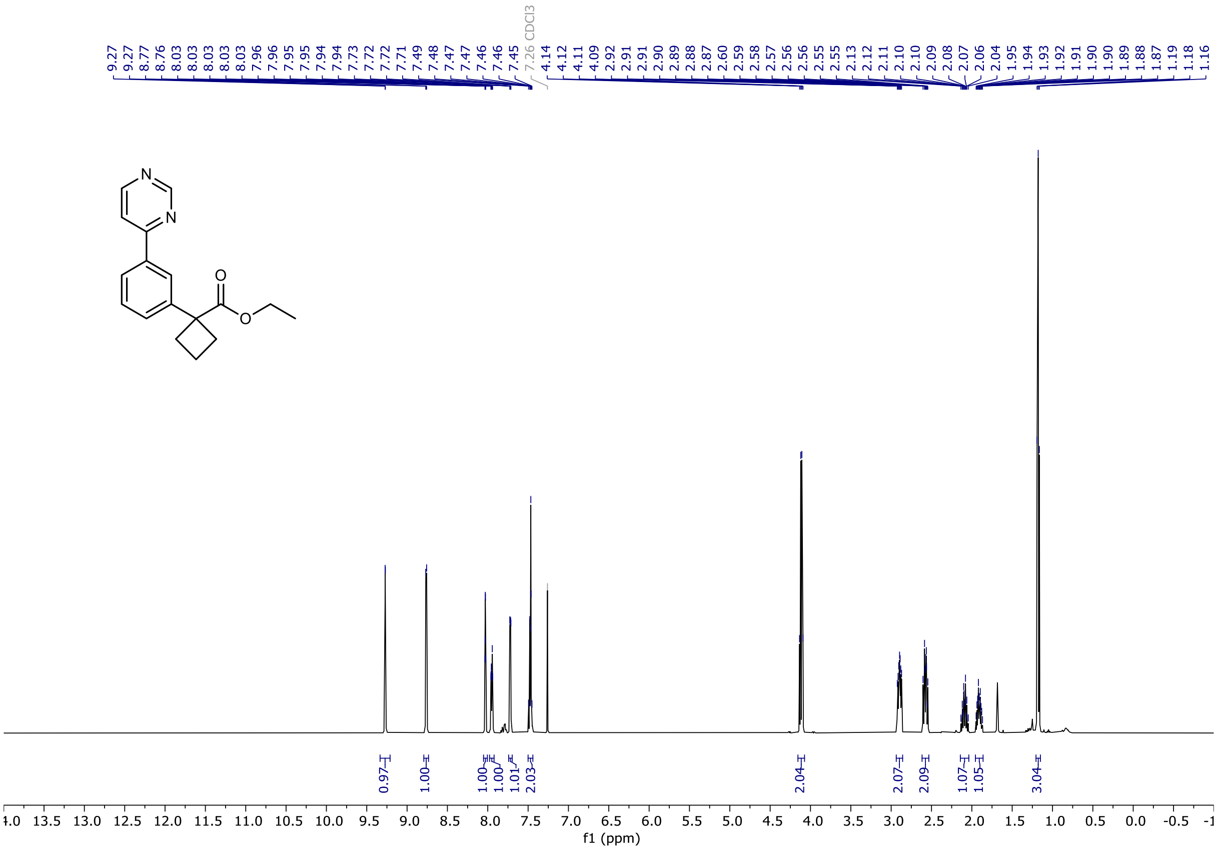

Supplementary Fig. 125. ^{1}H NMR spectra (500 MHz, $CDCl_3$, 298 K) of compound **3x**.

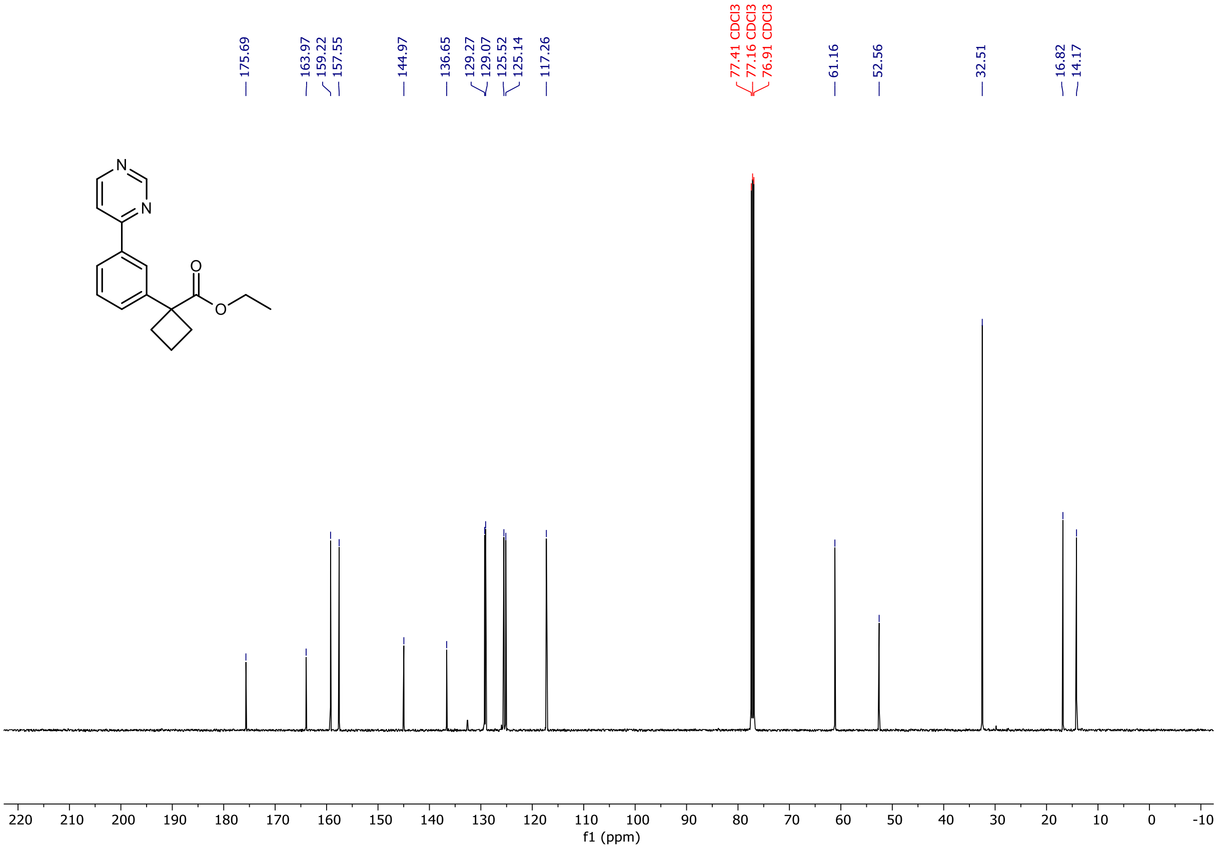

Supplementary Fig. 126. ^{13}C NMR spectra (126 MHz, $CDCl_3$, 298 K) of compound **3x**.

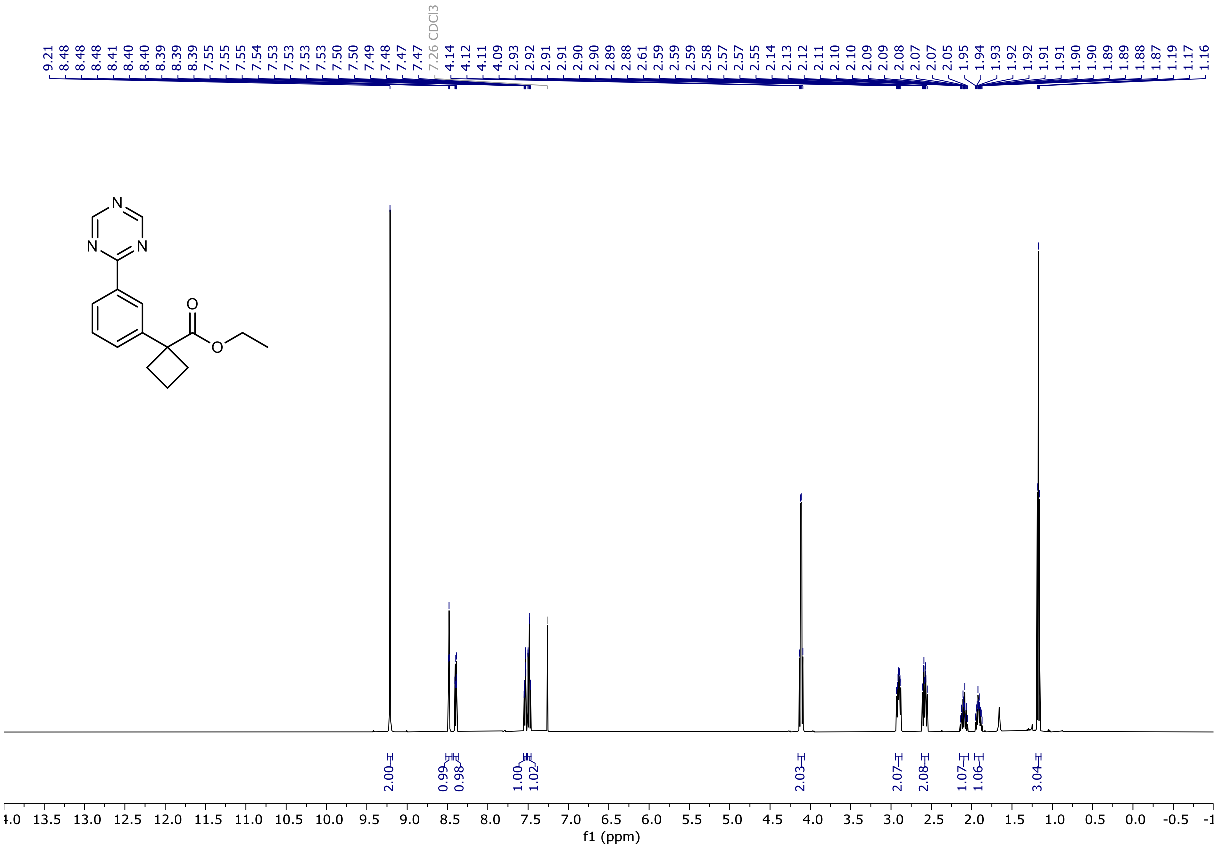

Supplementary Fig. 127. ^{1}H NMR spectra (500 MHz, $CDCl_3$, 298 K) of compound **3y**.

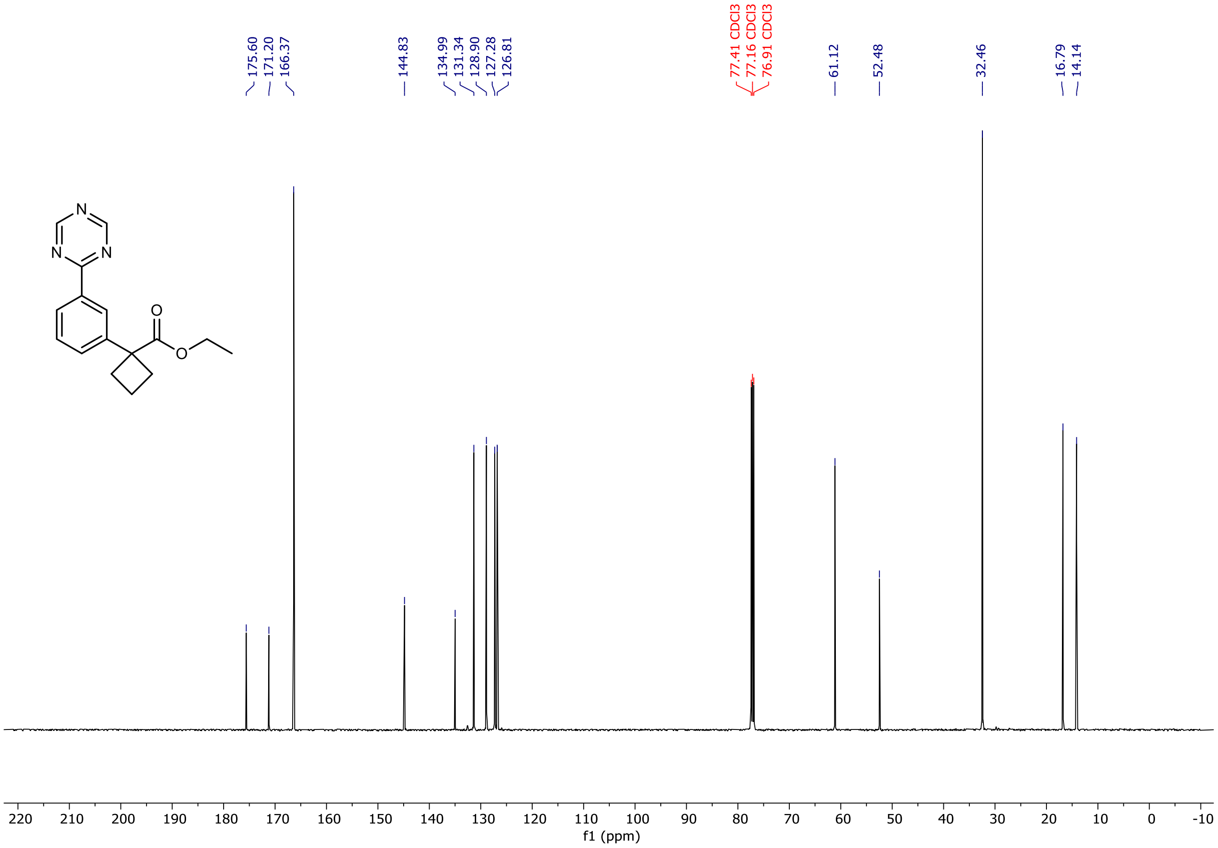

Supplementary Fig. 128. ^{13}C NMR spectra (126 MHz, $CDCl_3$, 298 K) of compound **3y**.

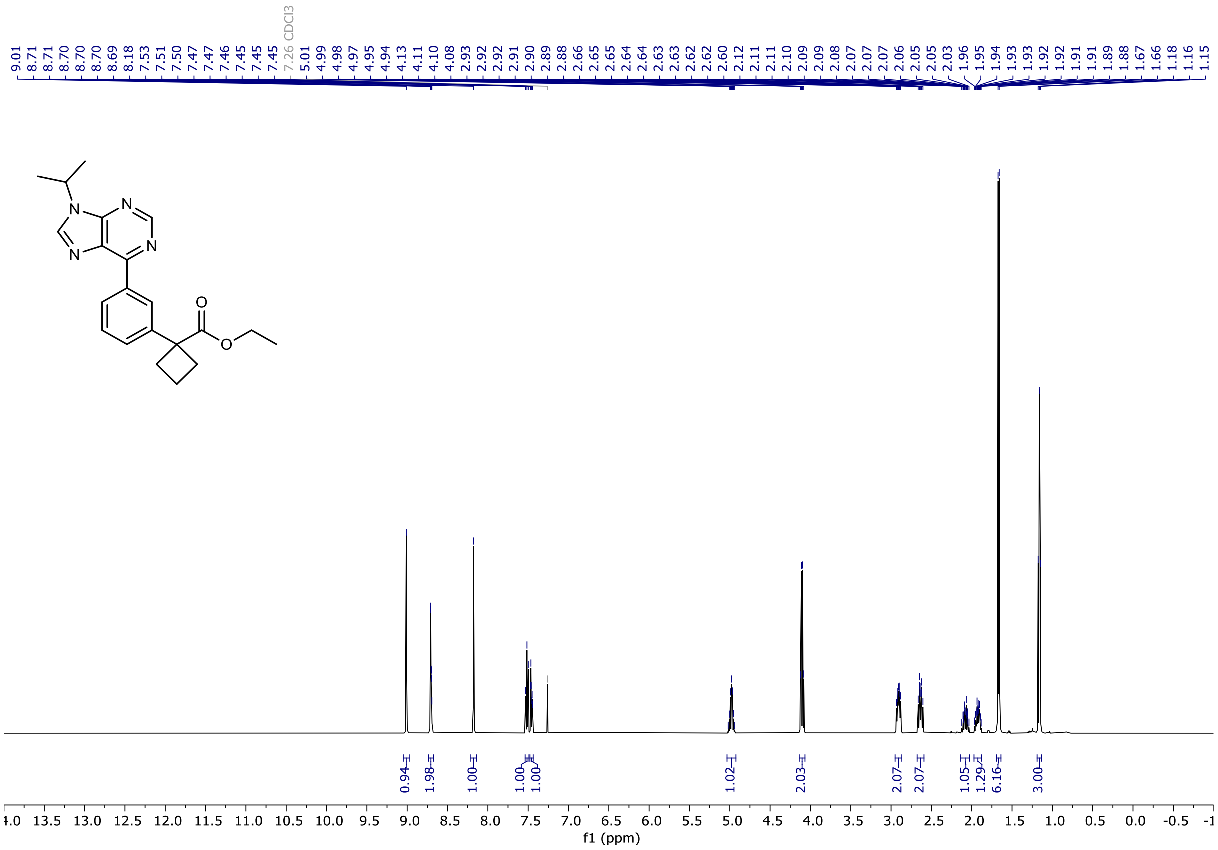

Supplementary Fig. 129. ^{1}H NMR spectra (500 MHz, $CDCl_3$, 298 K) of compound **3z**.

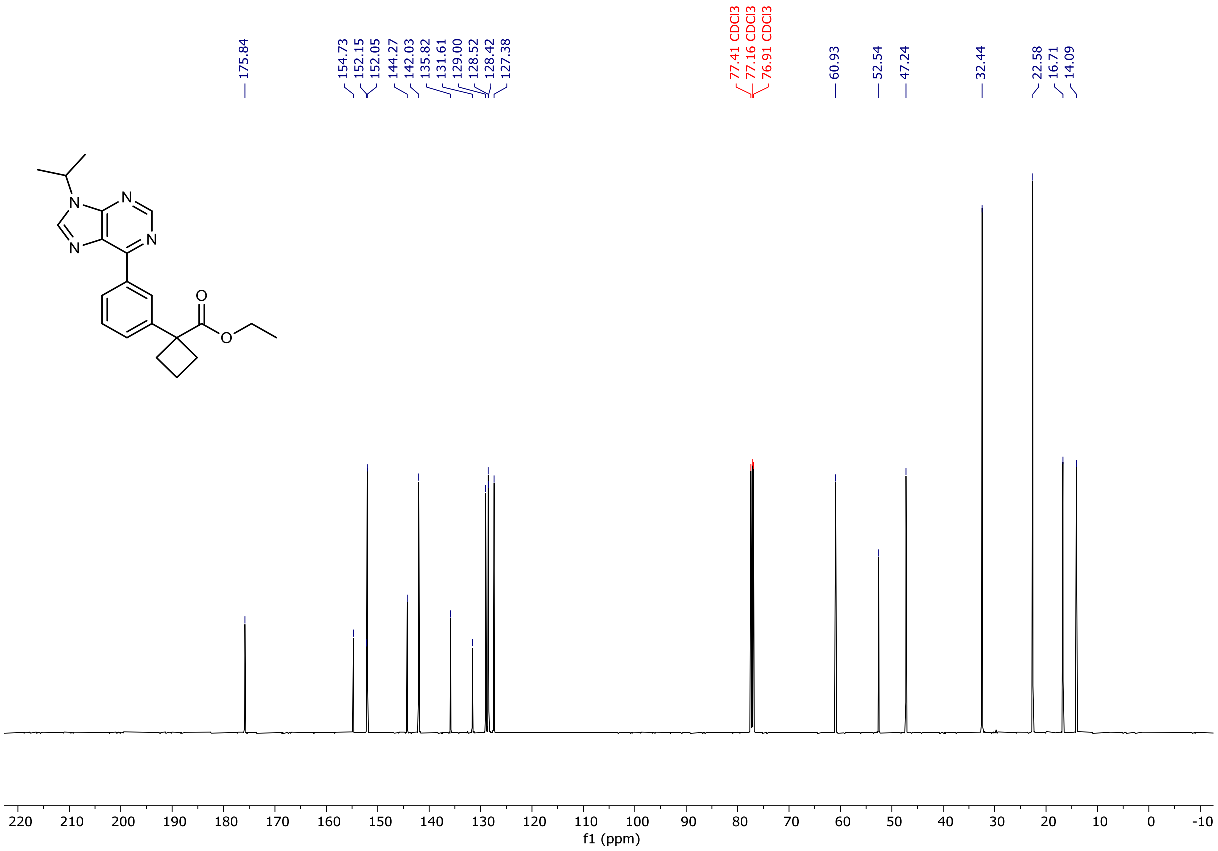

Supplementary Fig. 130. ^{13}C NMR spectra (126 MHz, $CDCl_3$, 298 K) of compound **3z**.

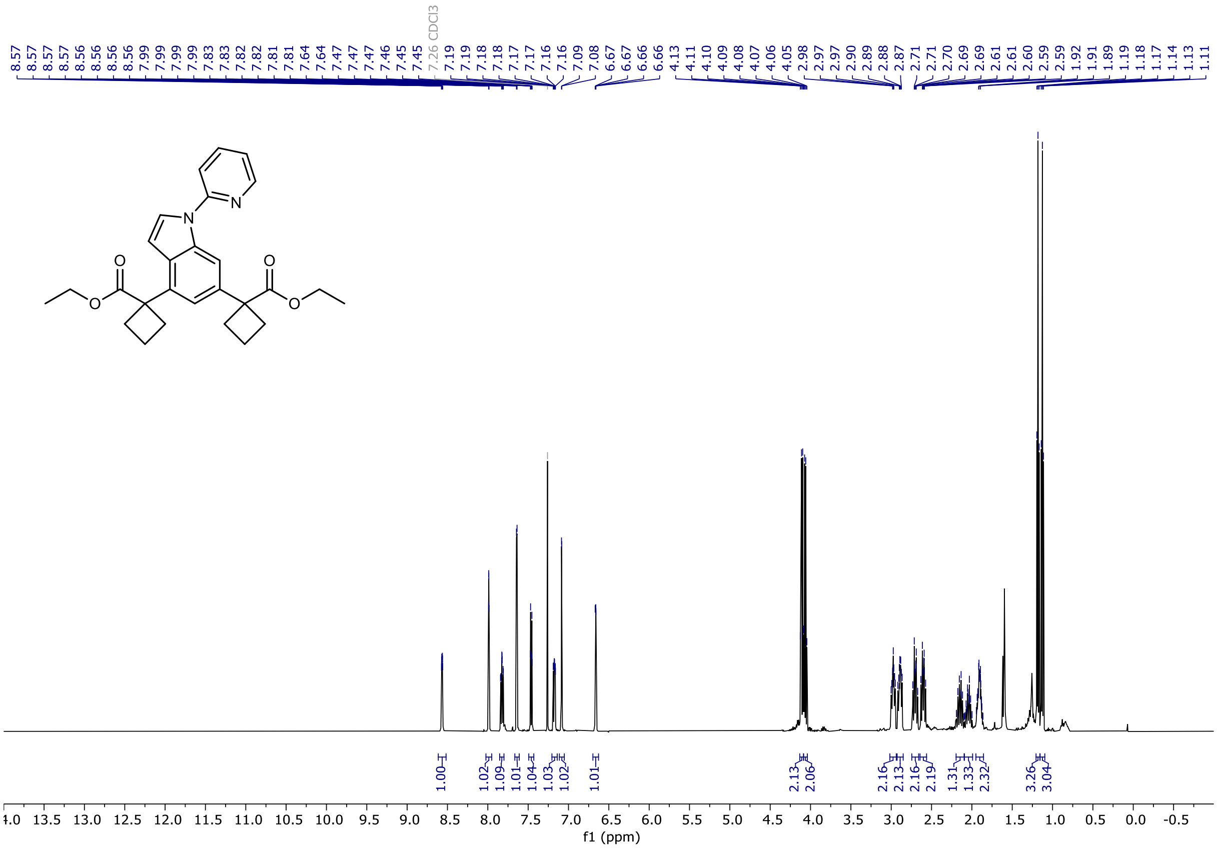

Supplementary Fig. 131. ^{1}H NMR spectra (500 MHz, $CDCl_3$, 298 K) of compound **3za**.

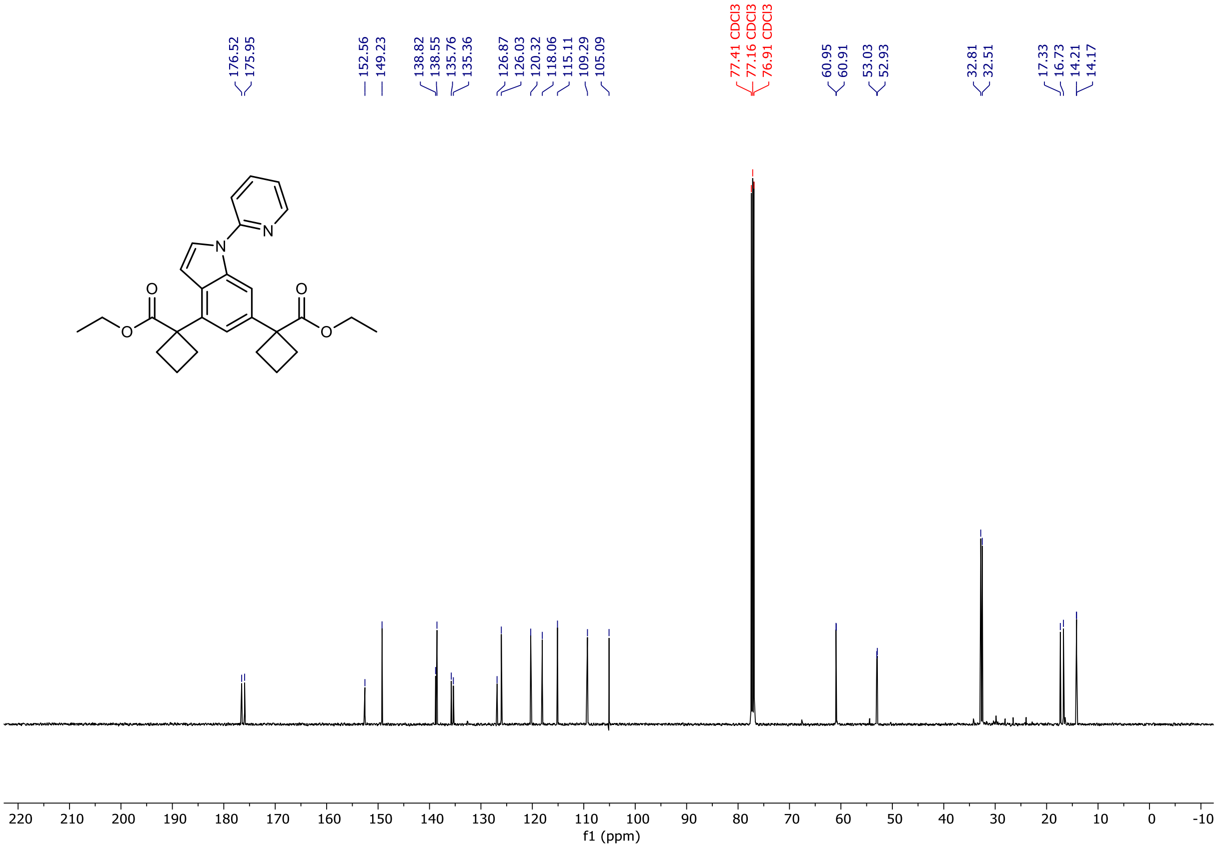

Supplementary Fig. 132. ^{13}C NMR spectra (126 MHz, $CDCl_3$, 298 K) of compound **3za**.

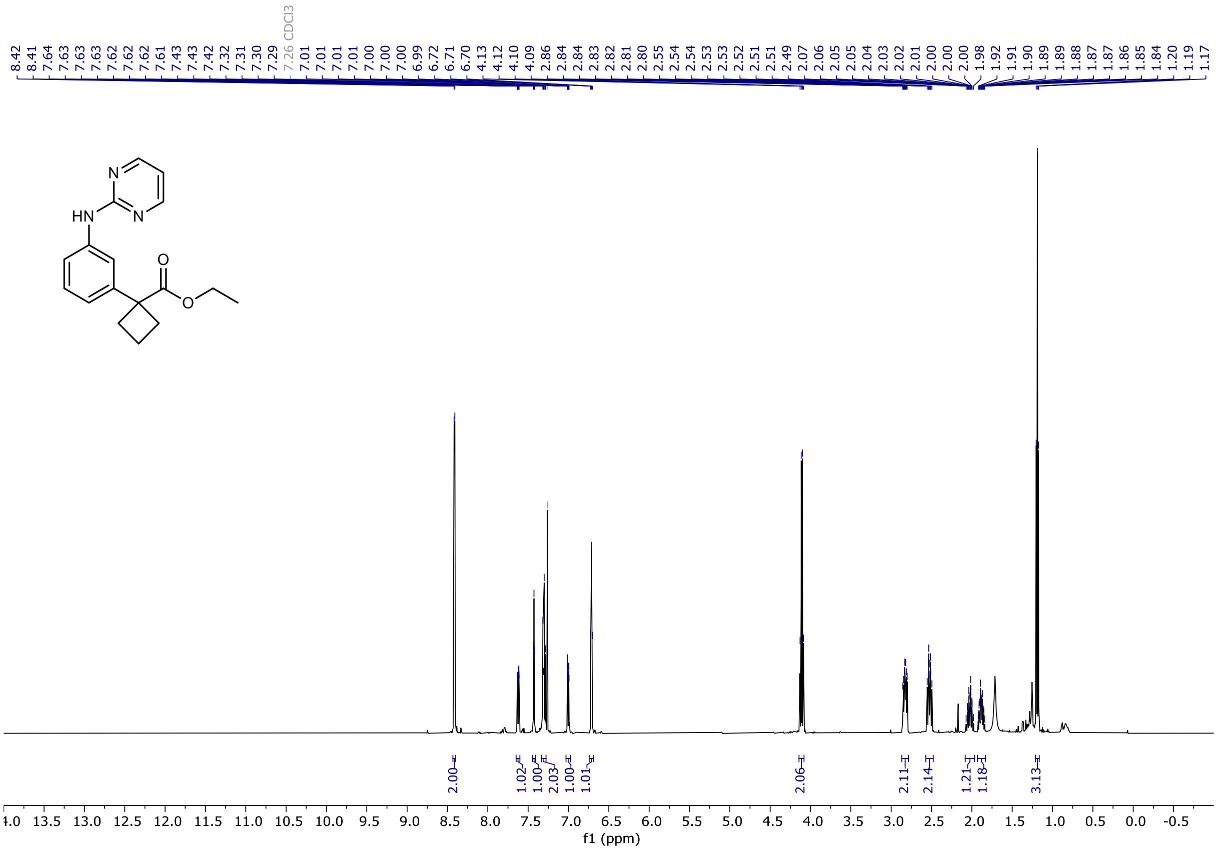

Supplementary Fig. 133. ^{1}H NMR spectra (500 MHz, $CDCl_3$, 298 K) of compound **3zb**.

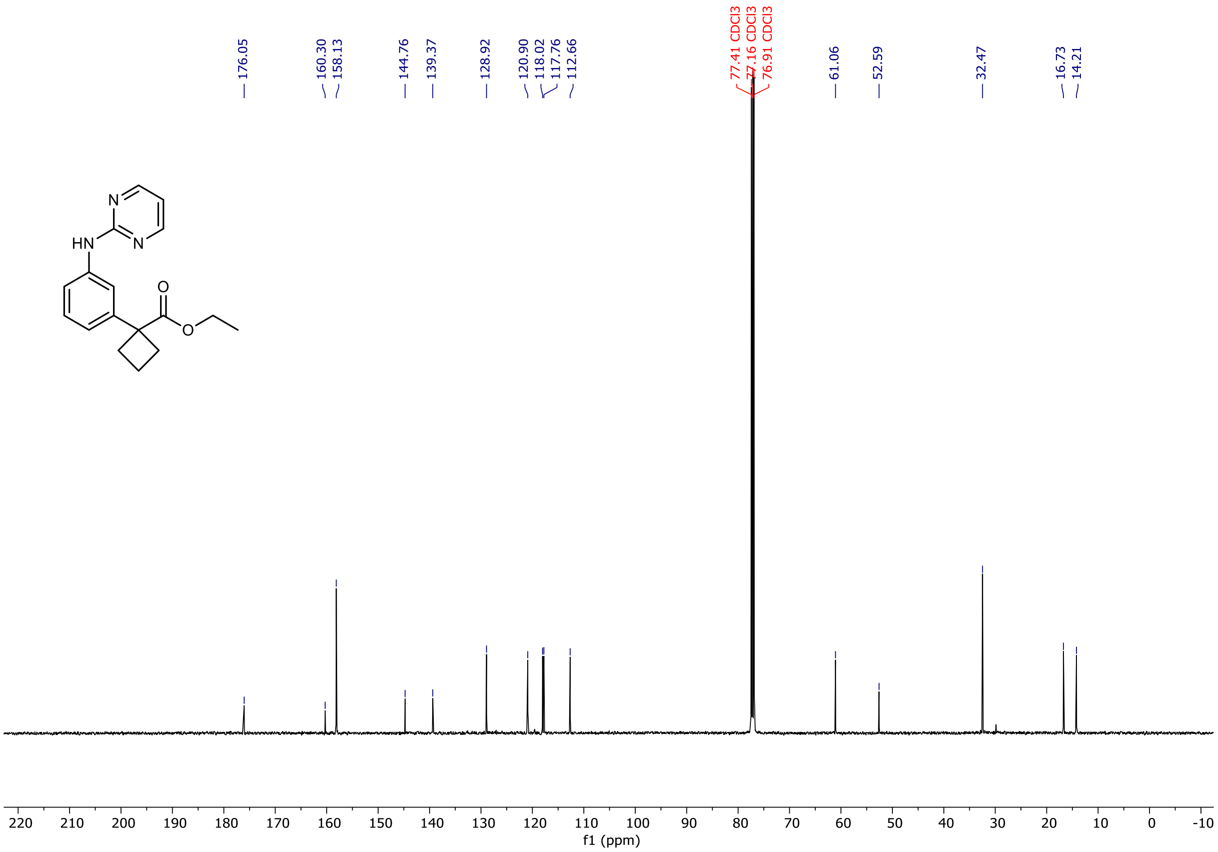

Supplementary Fig. 134. ^{13}C NMR spectra (126 MHz, $CDCl_3$, 298 K) of compound **3zb**.

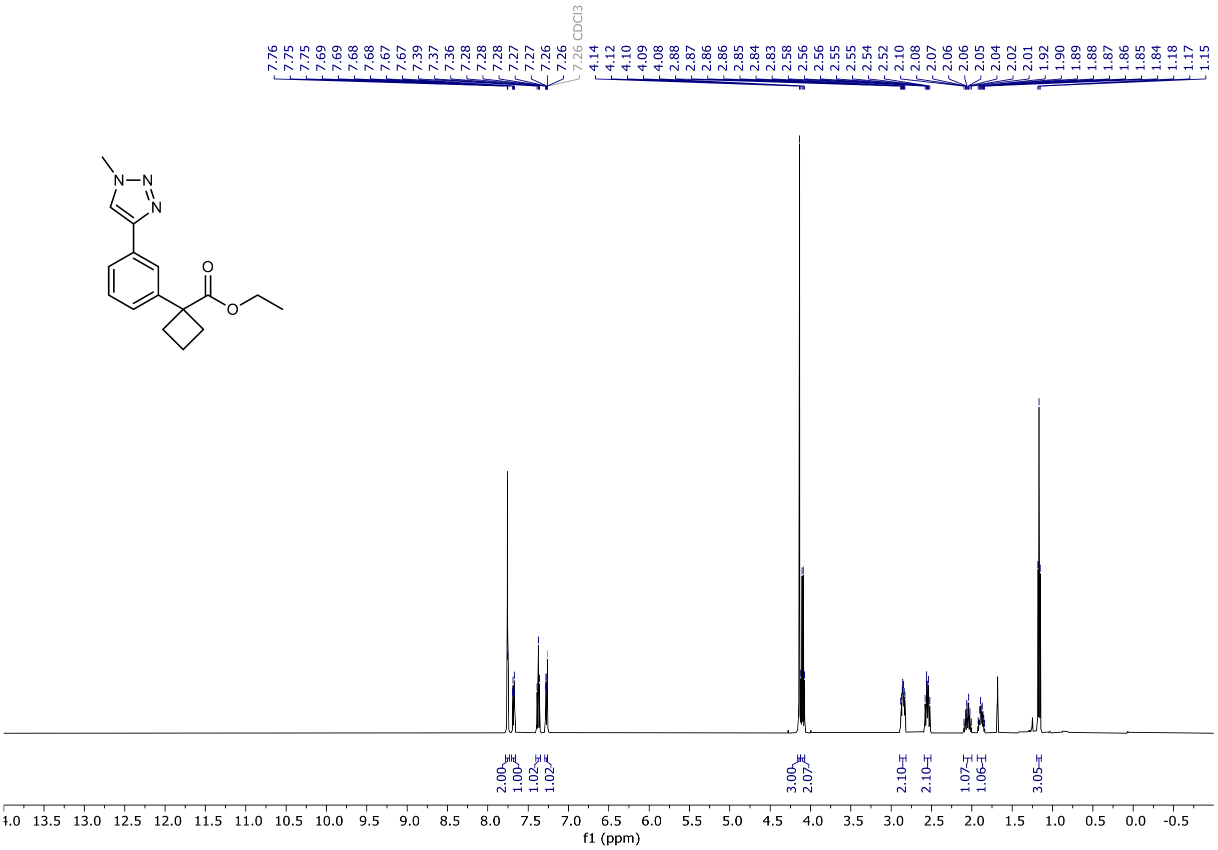

Supplementary Fig. 135. ^{1}H NMR spectra (500 MHz, $CDCl_3$, 298 K) of compound **3zc**.

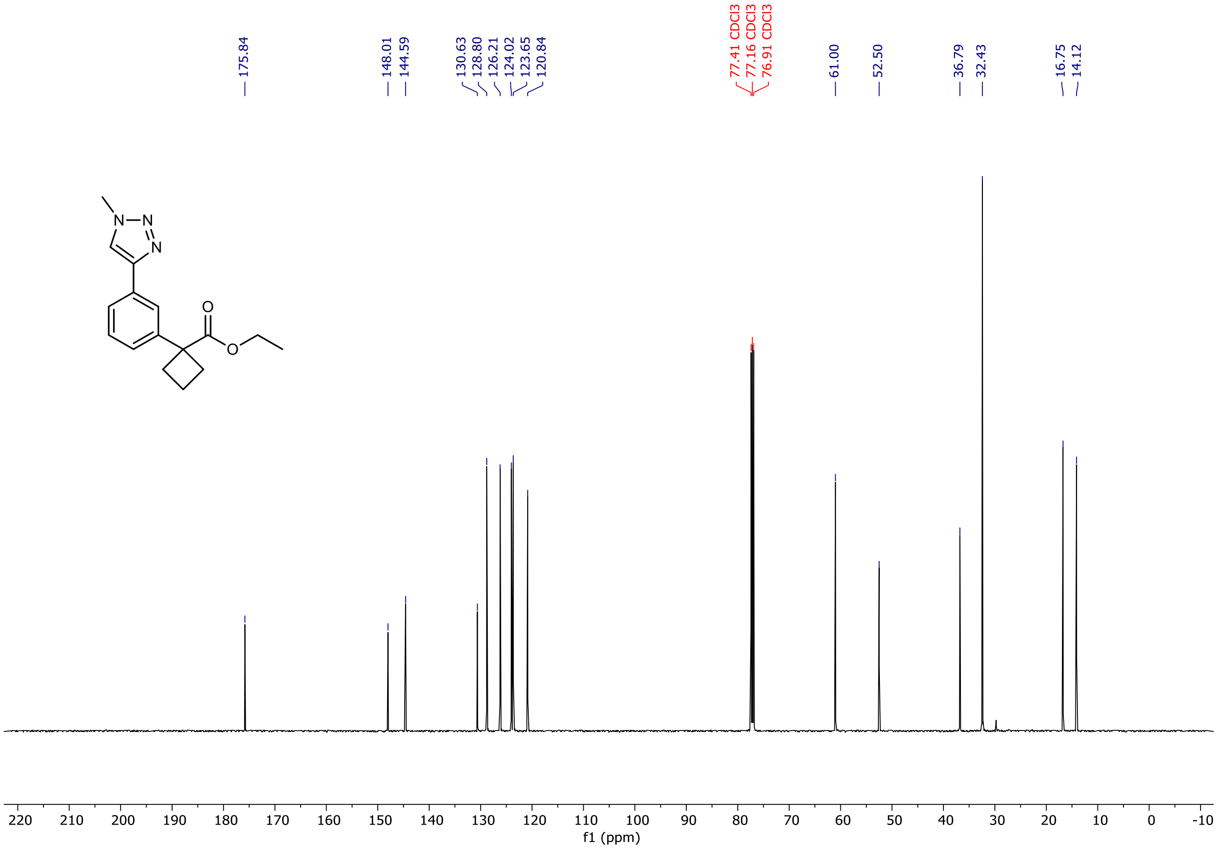

Supplementary Fig. 136. ^{13}C NMR spectra (126 MHz, $CDCl_3$, 298 K) of compound **3zc**.

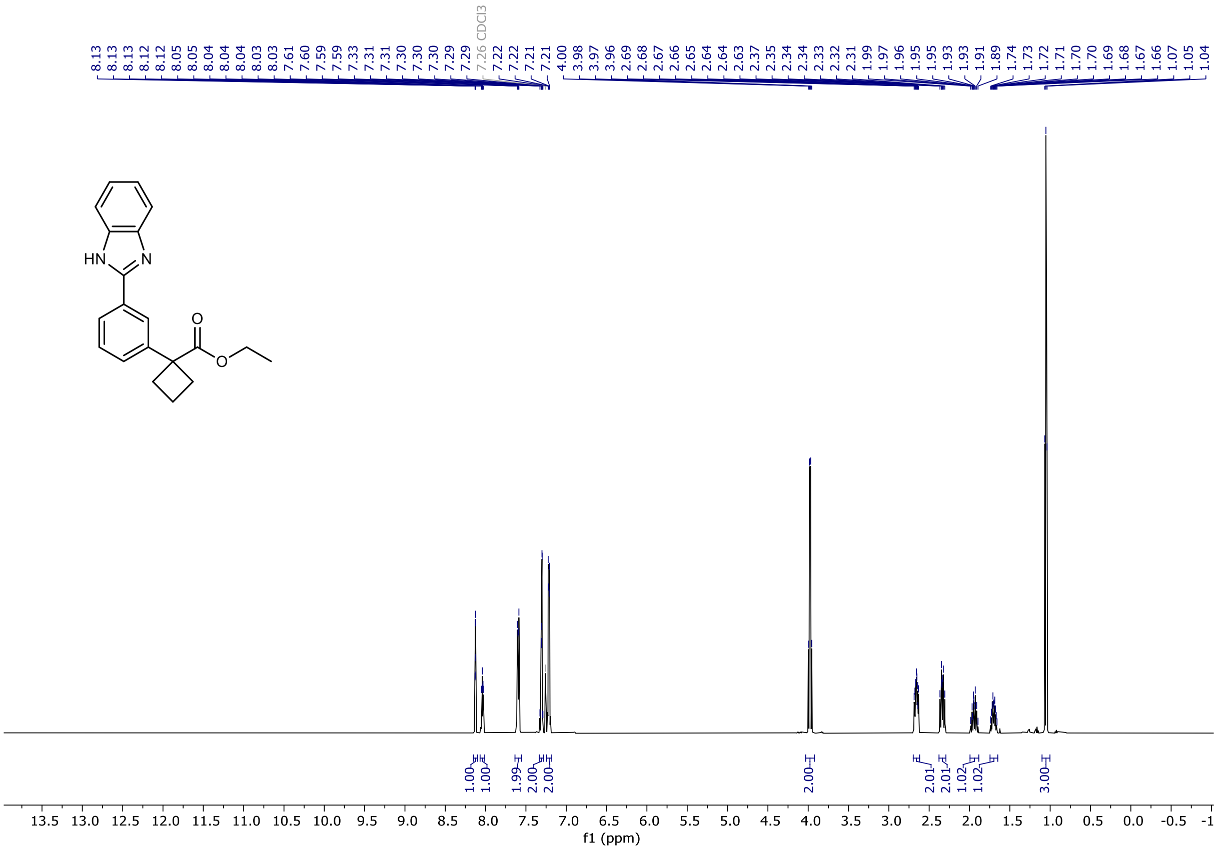

Supplementary Fig. 137. ^{1}H NMR spectra (500 MHz, $CDCl_3$, 298 K) of compound **3zd**.

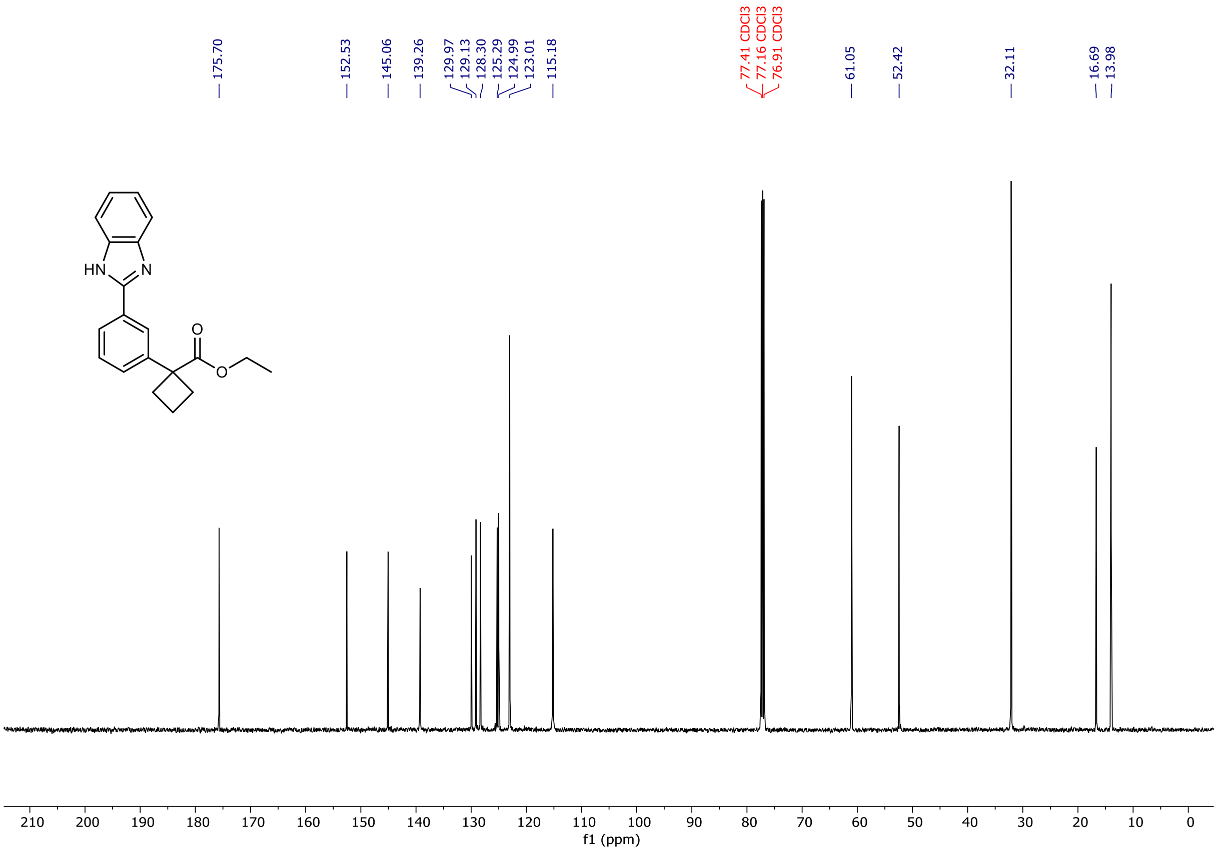

Supplementary Fig. 138. ^{13}C NMR spectra (126 MHz, $CDCl_3$, 298 K) of compound **3zd**.

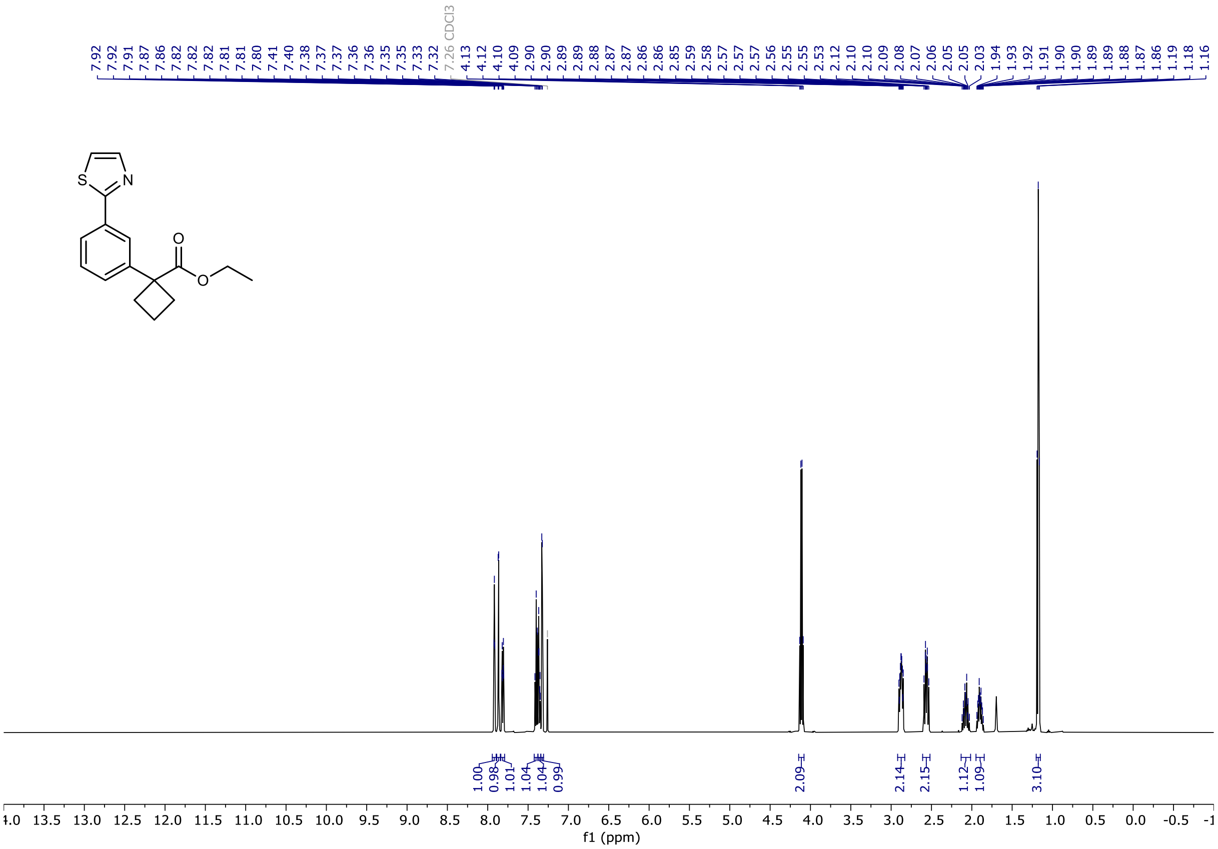

Supplementary Fig. 139. ^{1}H NMR spectra (500 MHz, $CDCl_3$, 298 K) of compound **3ze**.

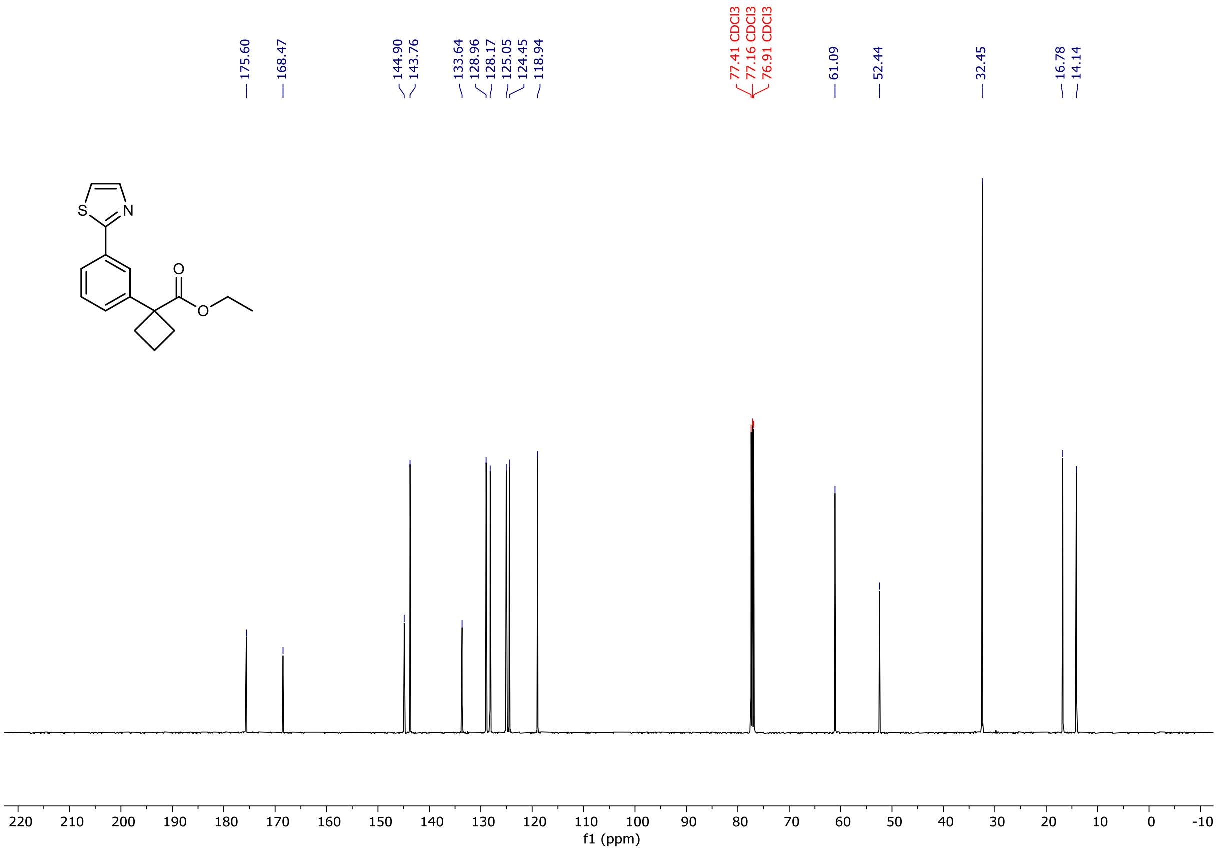

Supplementary Fig. 140. ^{13}C NMR spectra (126 MHz, $CDCl_3$, 298 K) of compound **3ze**.

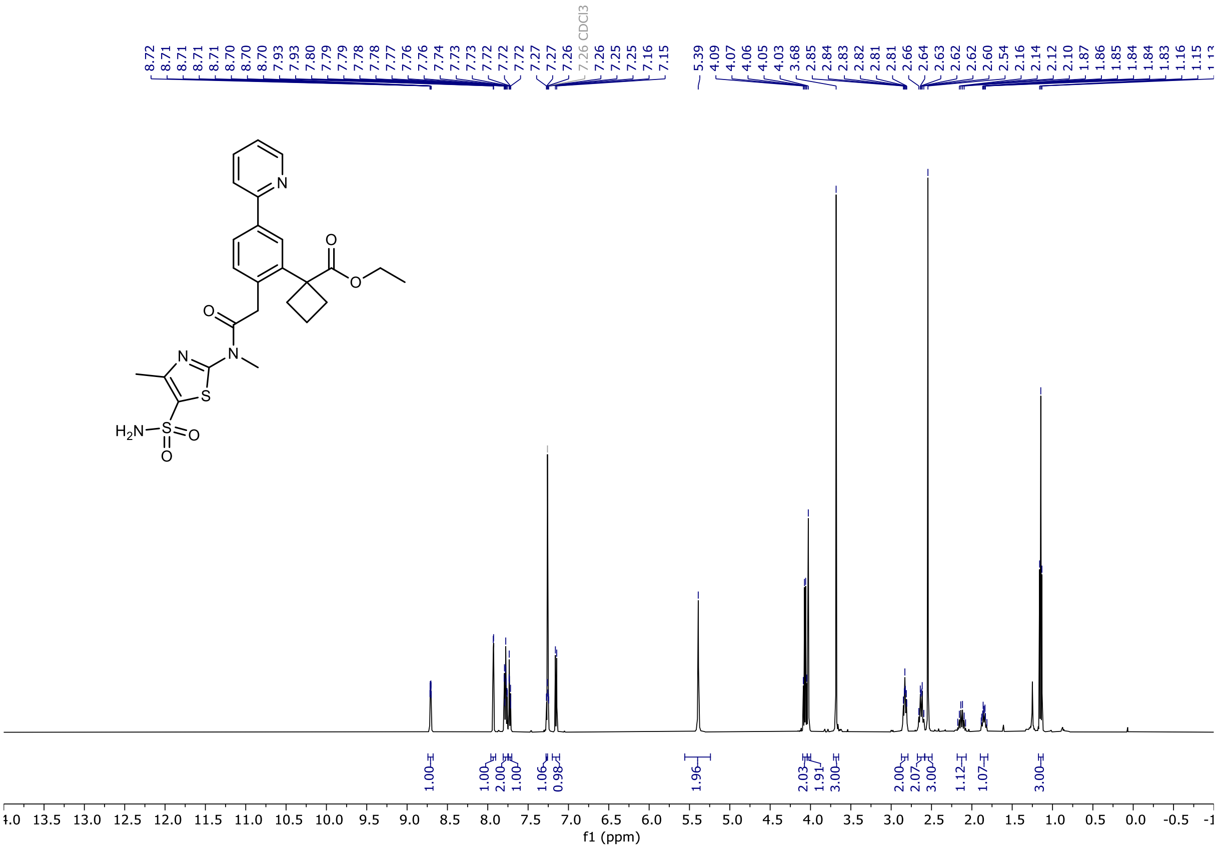

Supplementary Fig. 141. ^{1}H NMR spectra (500 MHz, $CDCl_3$, 298 K) of compound **5a**.

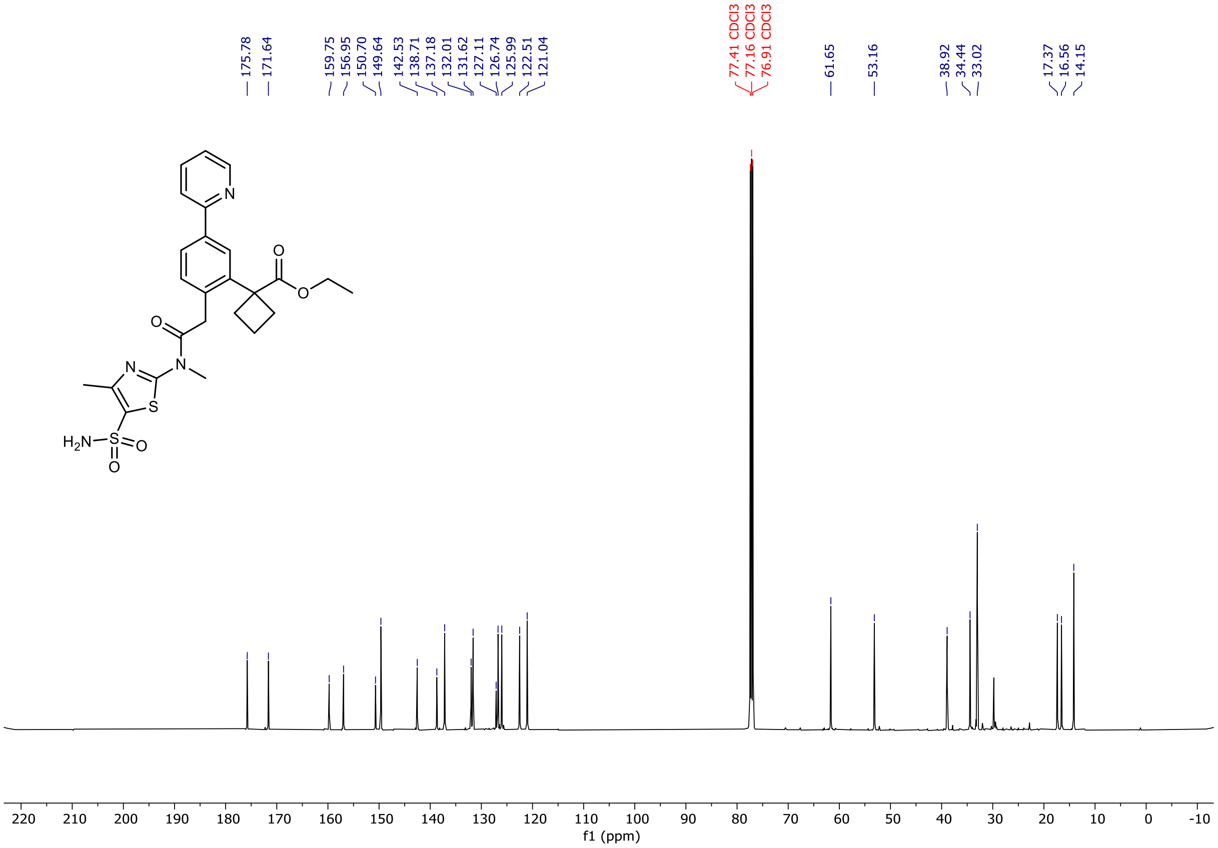

Supplementary Fig. 142. ^{13}C NMR spectra (126 MHz, $CDCl_3$, 298 K) of compound **5a**.

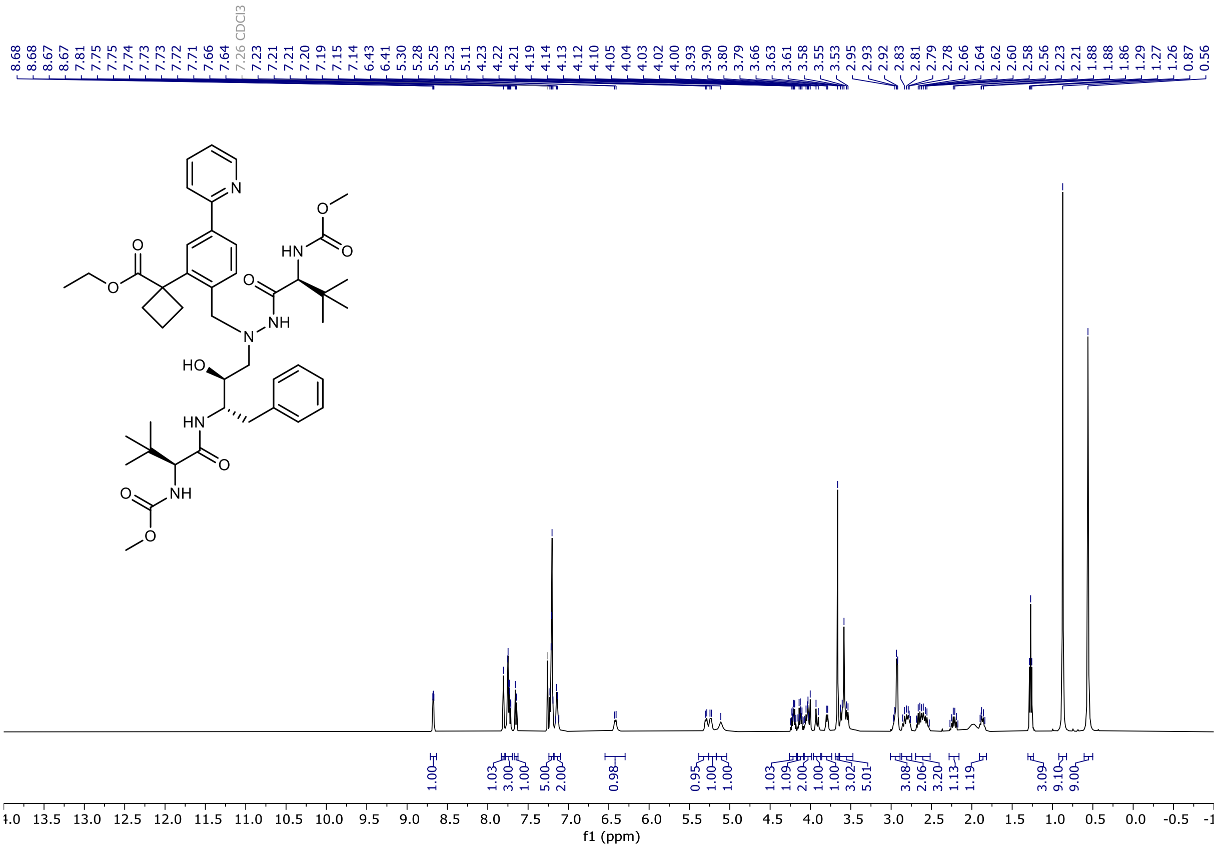

Supplementary Fig. 143. ^{1}H NMR spectra (500 MHz, $CDCl_3$, 298 K) of compound **5b**.

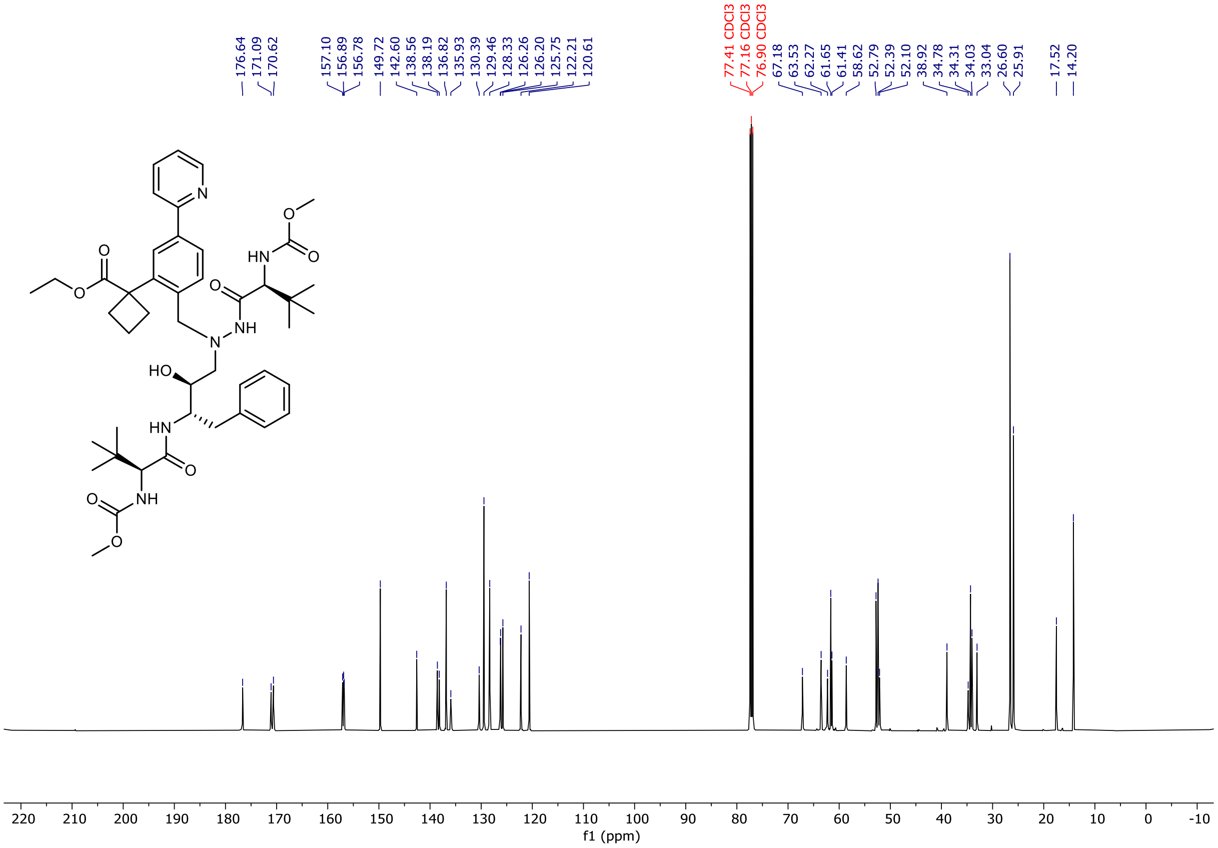

Supplementary Fig. 144. ^{13}C NMR spectra (126 MHz, $CDCl_3$, 298 K) of compound **5b**.

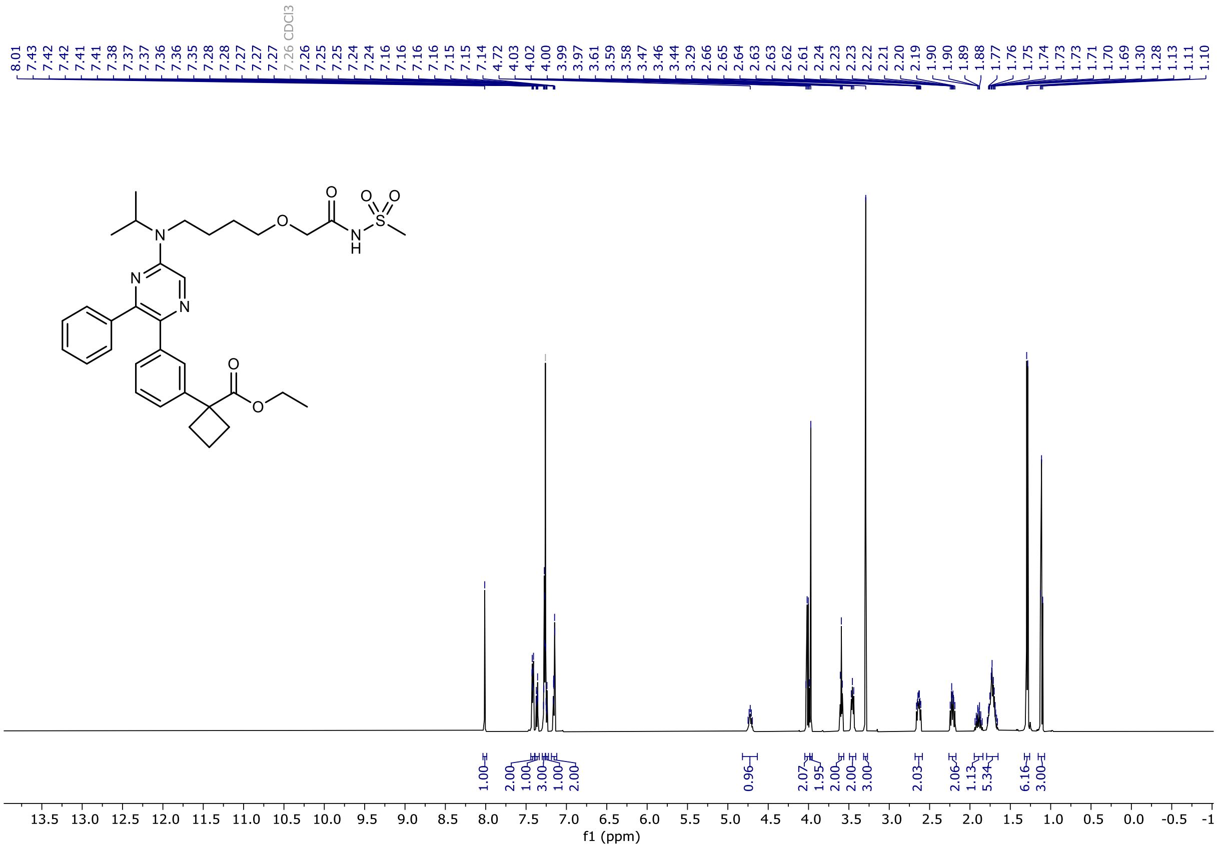

Supplementary Fig. 145. ^{1}H NMR spectra (500 MHz, $CDCl_3$, 298 K) of compound **5c**.

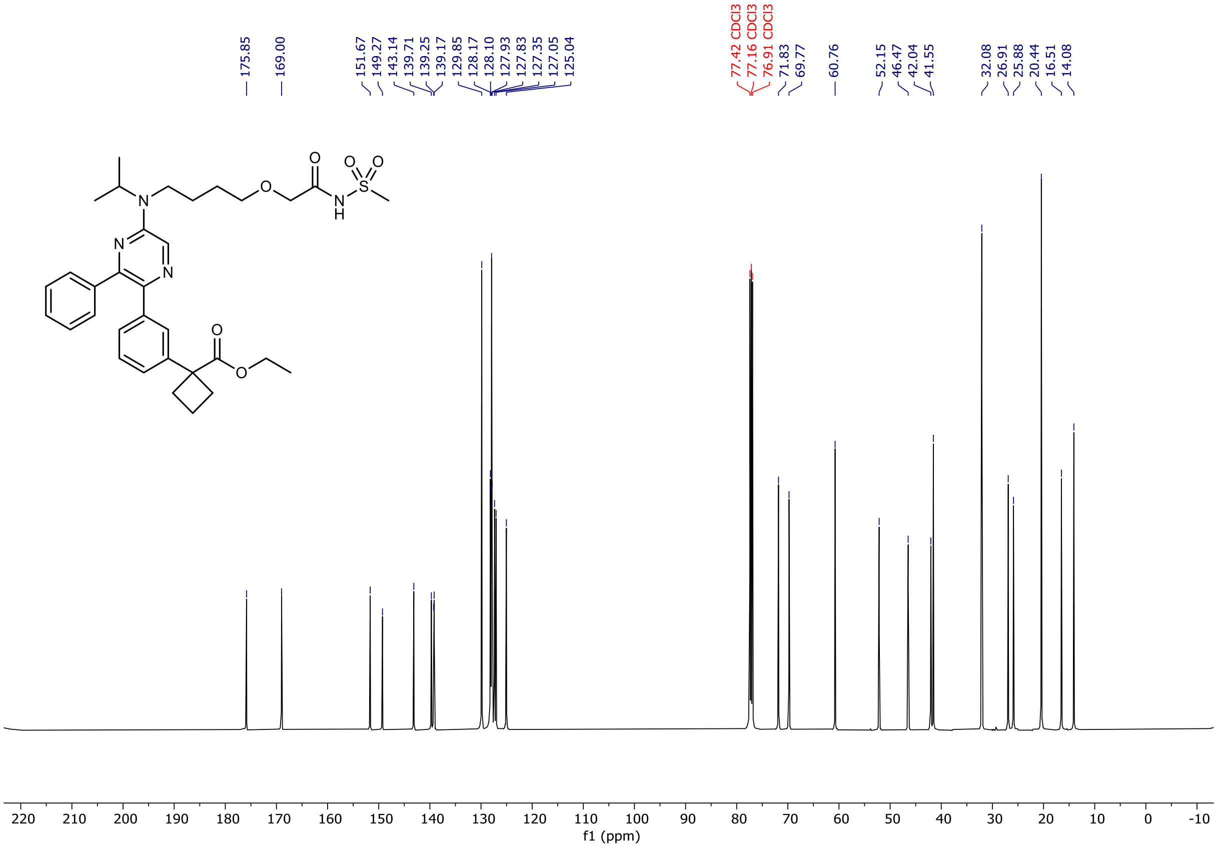

Supplementary Fig. 146. ^{13}C NMR spectra (126 MHz, $CDCl_3$, 298 K) of compound **5c**.

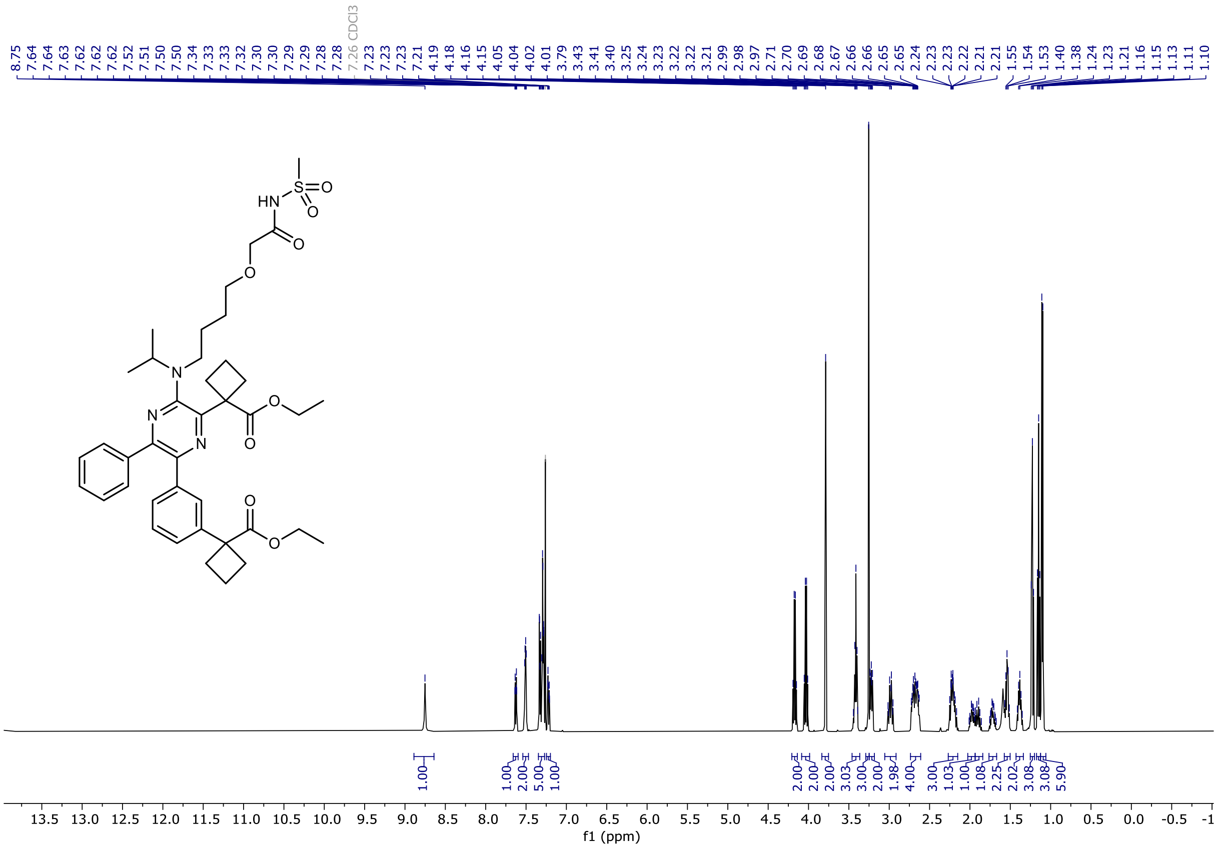

Supplementary Fig. 147. ^{1}H NMR spectra (500 MHz, $CDCl_3$, 298 K) of compound **5c2**.

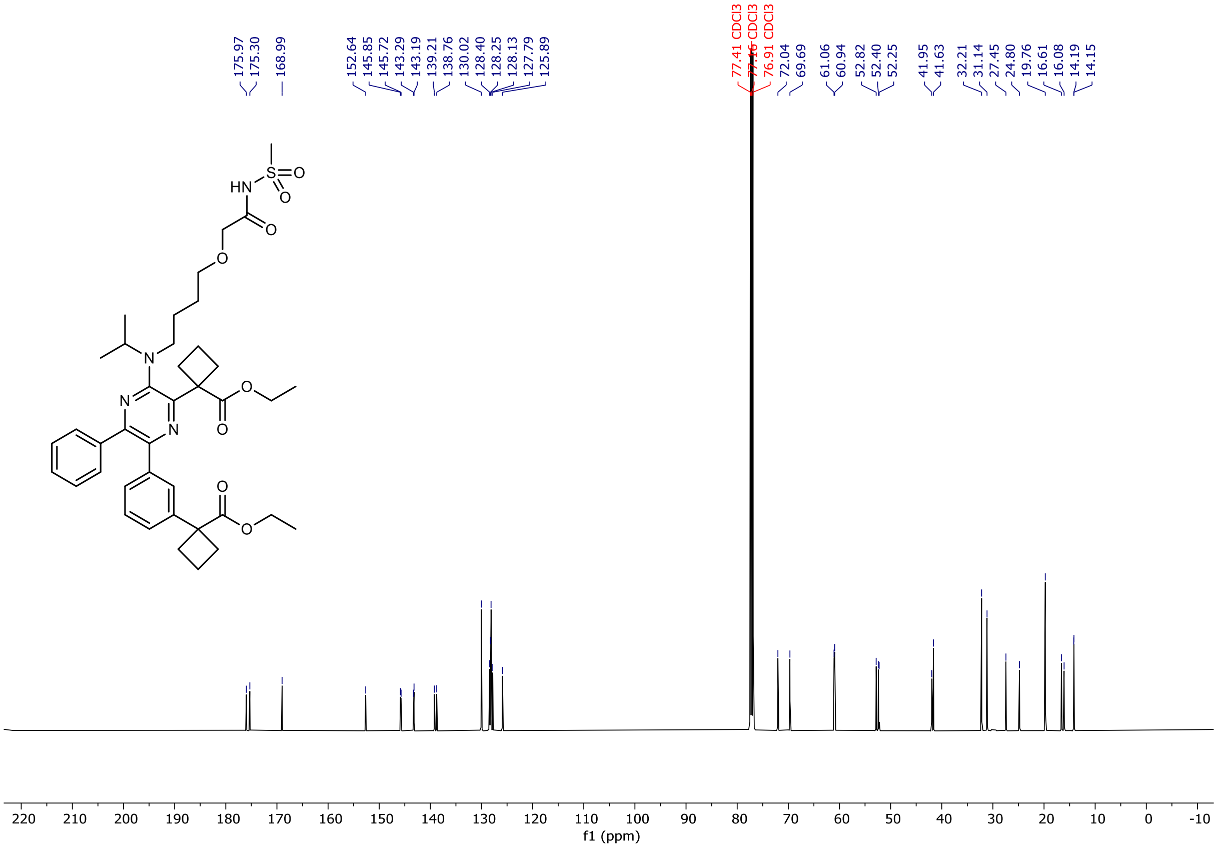

Supplementary Fig. 148. ^{13}C NMR spectra (126 MHz, $CDCl_3$, 298 K) of compound **5c2**.

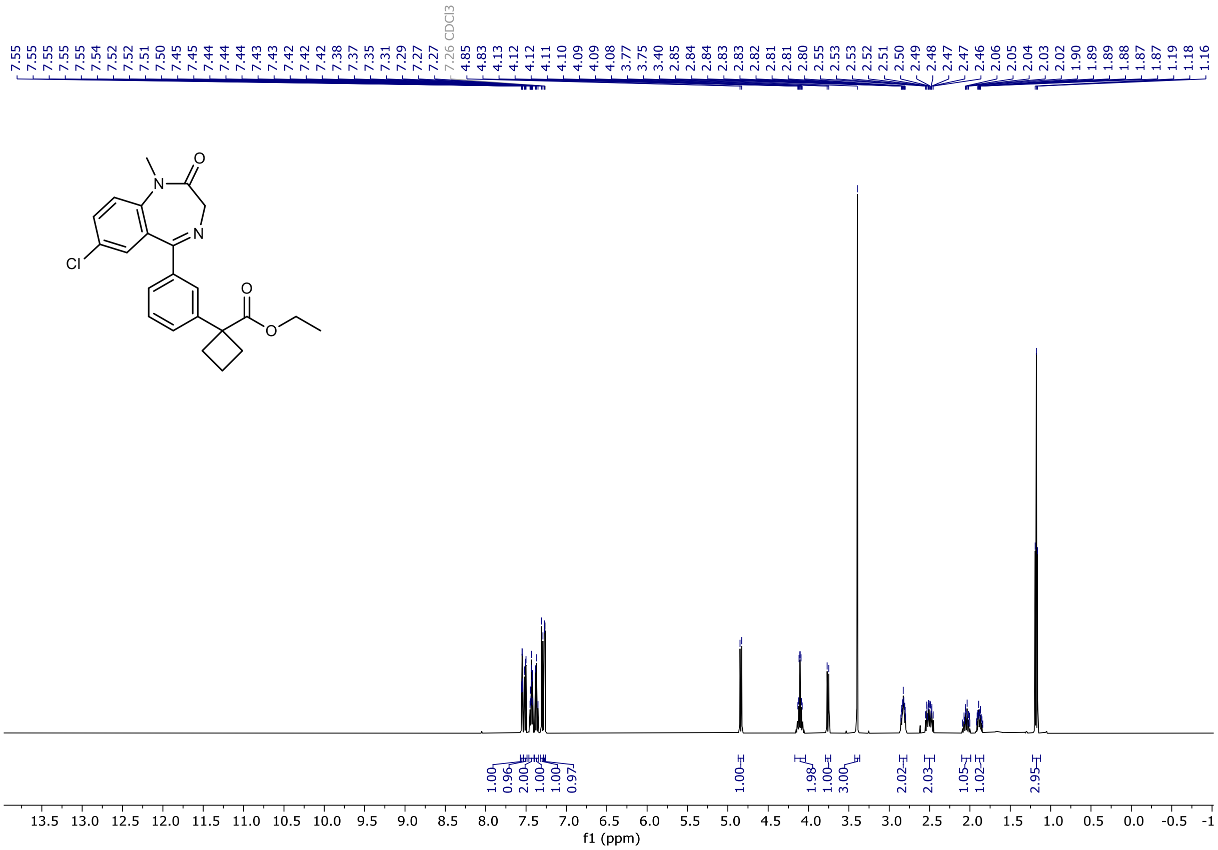

Supplementary Fig. 149. ^{1}H NMR spectra (500 MHz, $CDCl_3$, 298 K) of compound **5d**.

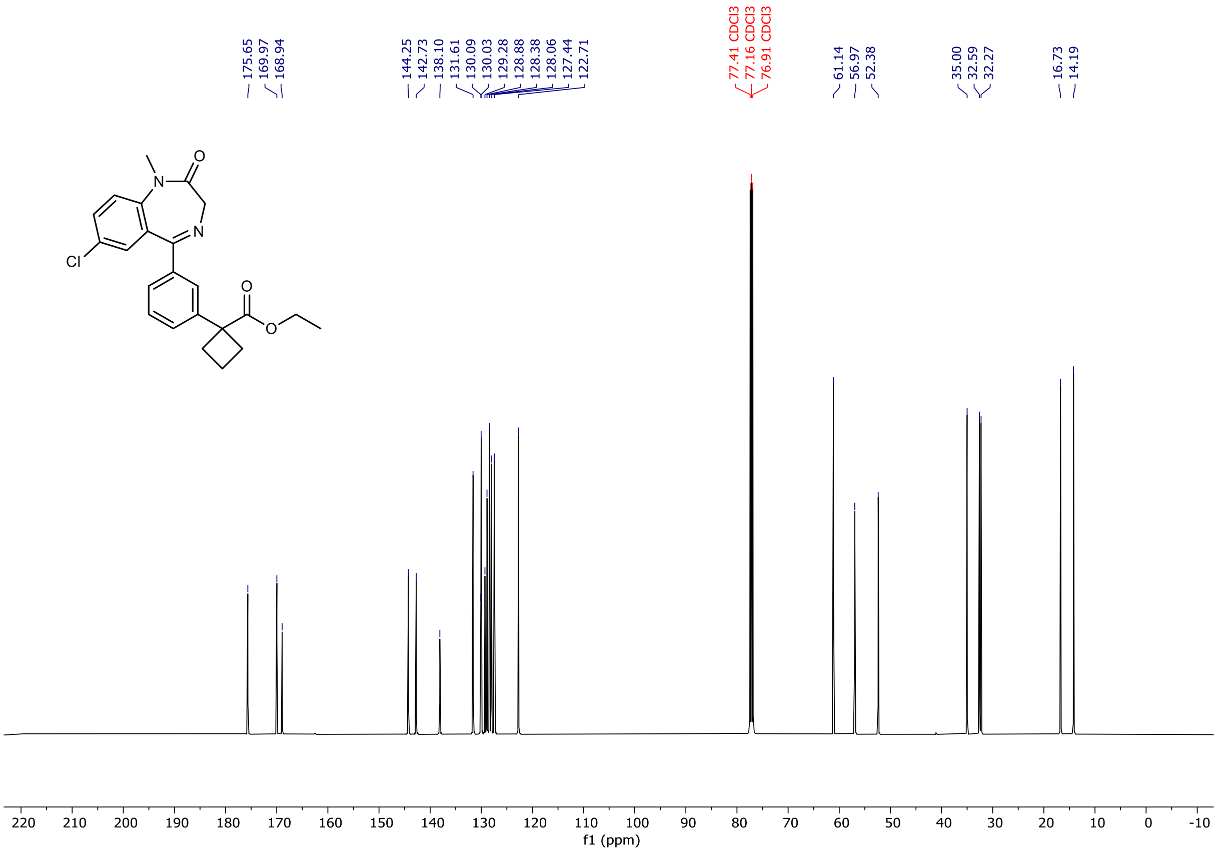

Supplementary Fig. 150. ^{13}C NMR spectra (126 MHz, $CDCl_3$, 298 K) of compound **5d**.

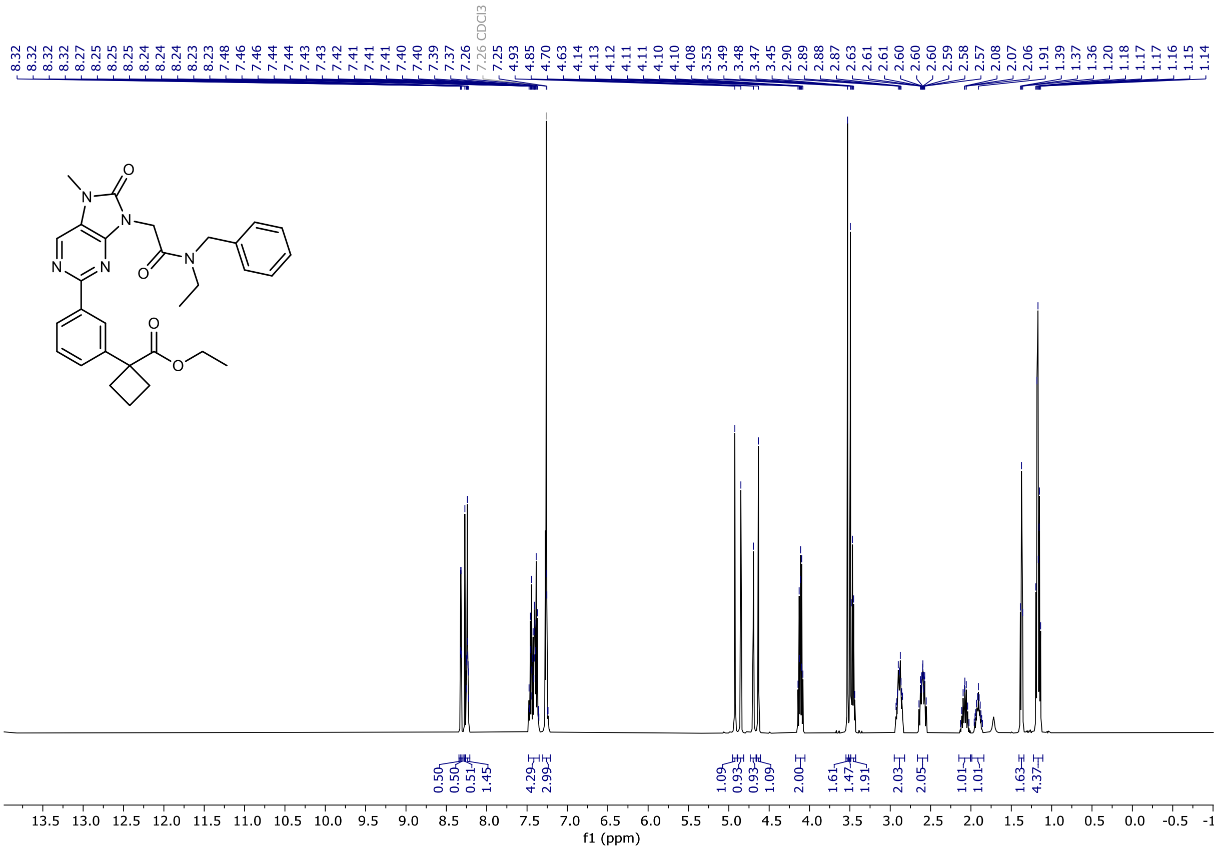

Supplementary Fig. 151. ^{1}H NMR spectra (500 MHz, $CDCl_3$, 298 K) of compound **5e**.

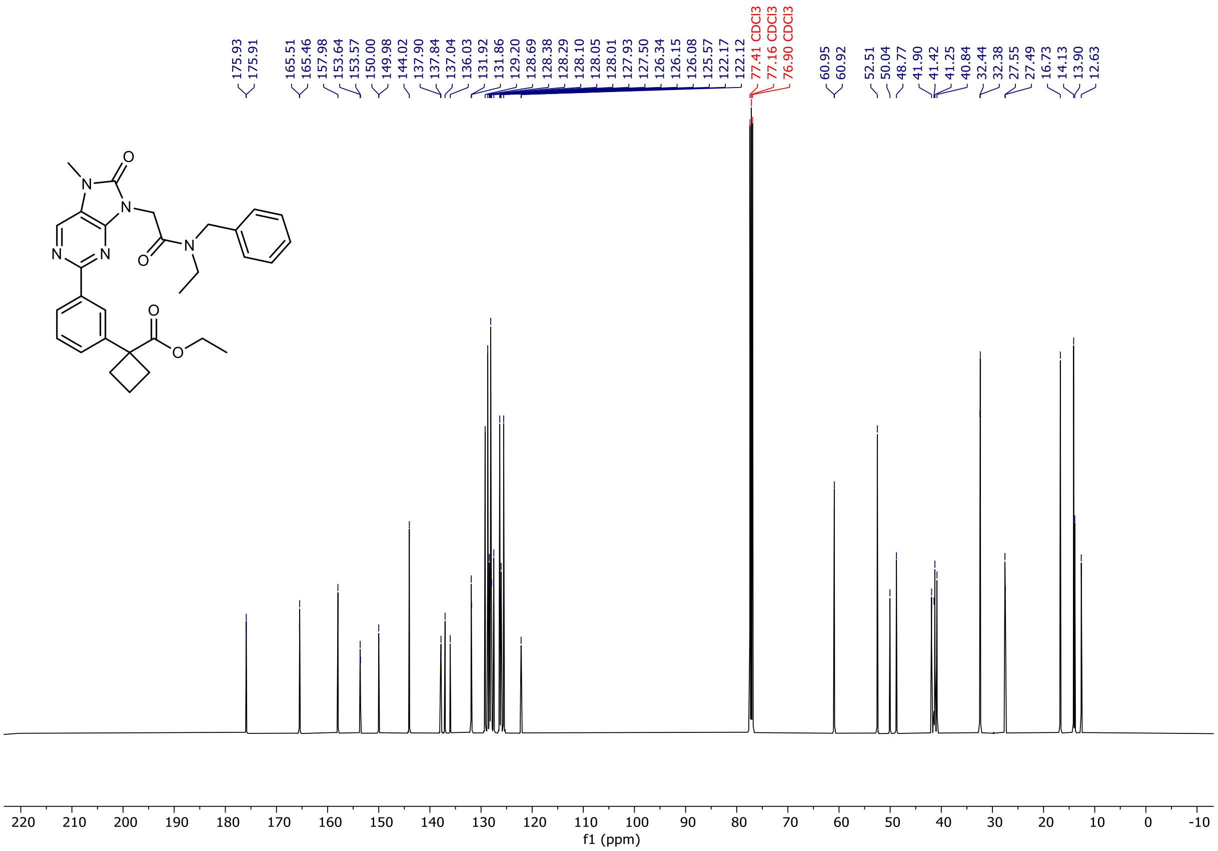

Supplementary Fig. 152. ^{13}C NMR spectra (126 MHz, $CDCl_3$, 298 K) of compound **5e**.

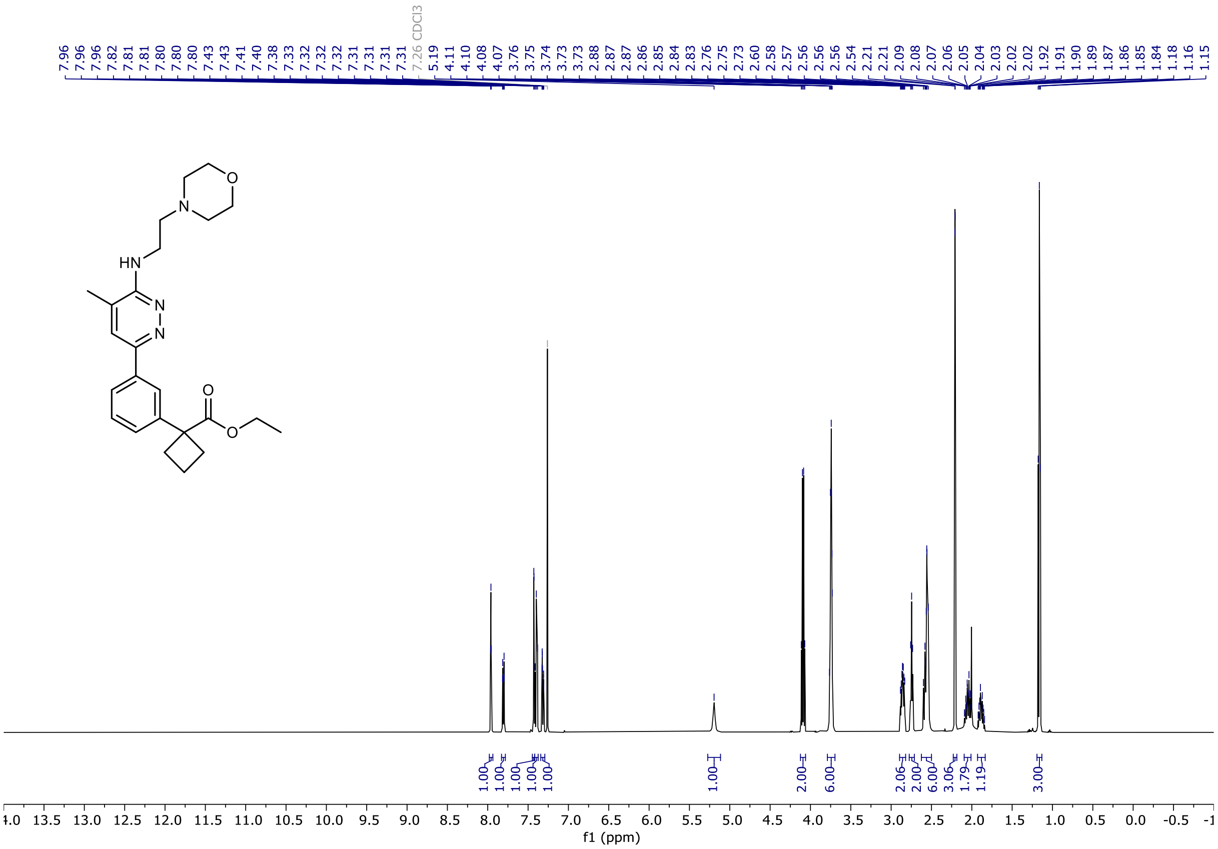

Supplementary Fig. 153. ^{1}H NMR spectra (500 MHz, $CDCl_3$, 298 K) of compound **5f**.

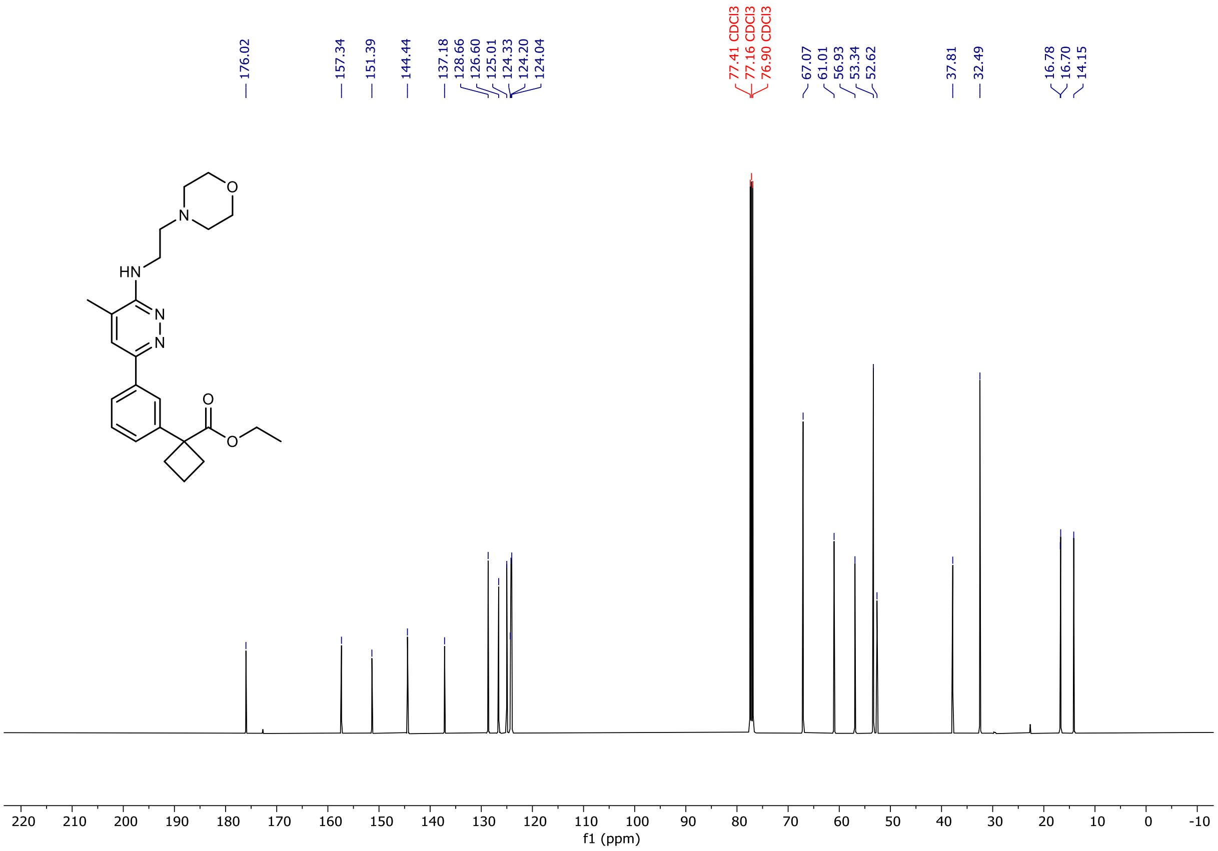

Supplementary Fig. 154. ^{13}C NMR spectra (126 MHz, $CDCl_3$, 298 K) of compound **5f**.

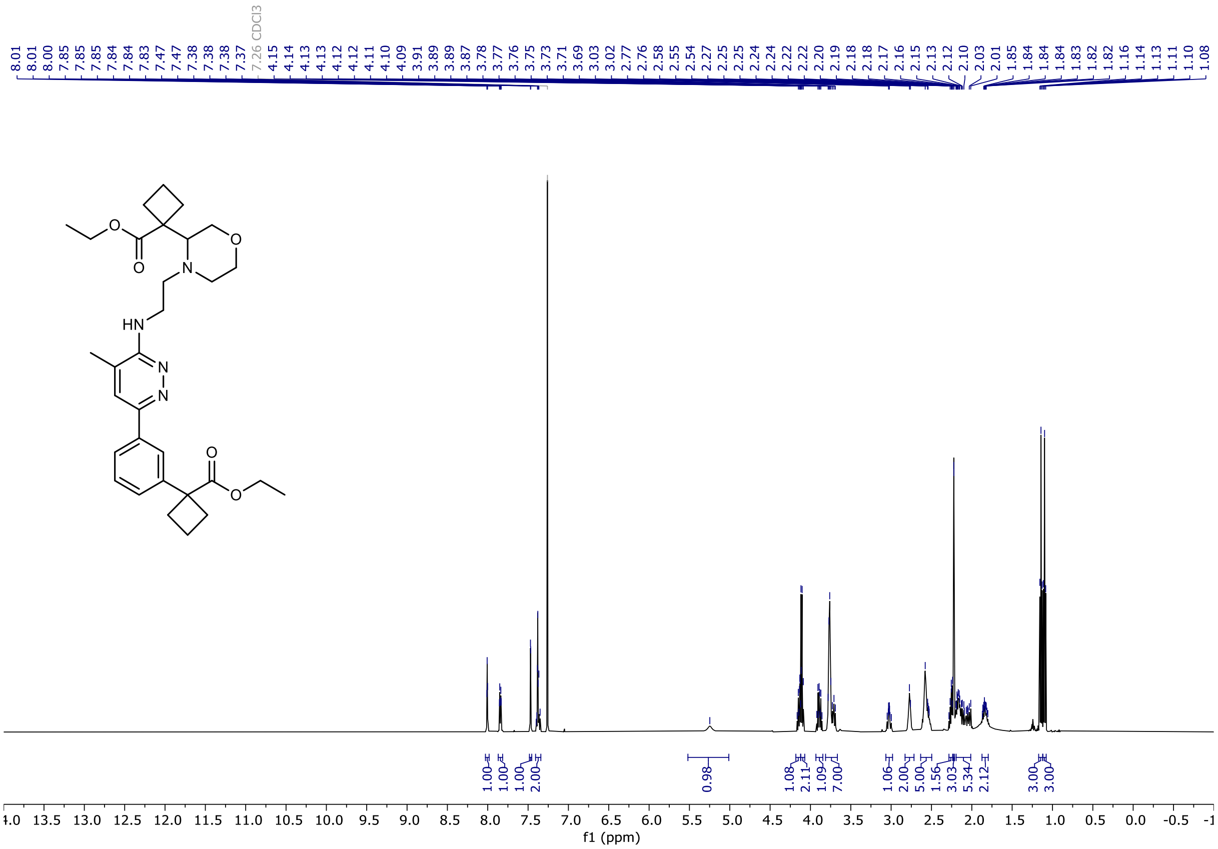

Supplementary Fig. 155. ^{1}H NMR spectra (500 MHz, $CDCl_3$, 298 K) of compound **5f2**.

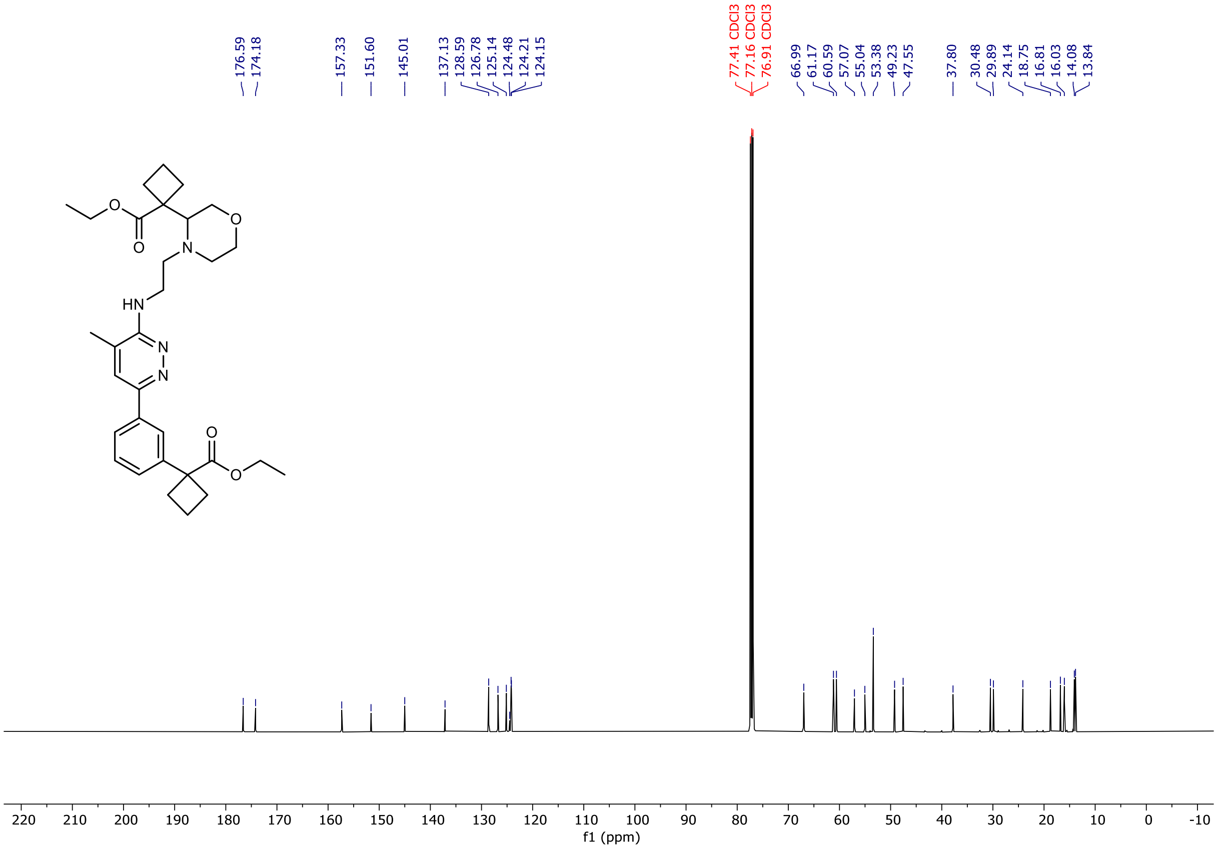

Supplementary Fig. 156. ^{13}C NMR spectra (126 MHz, $CDCl_3$, 298 K) of compound **5f2**.

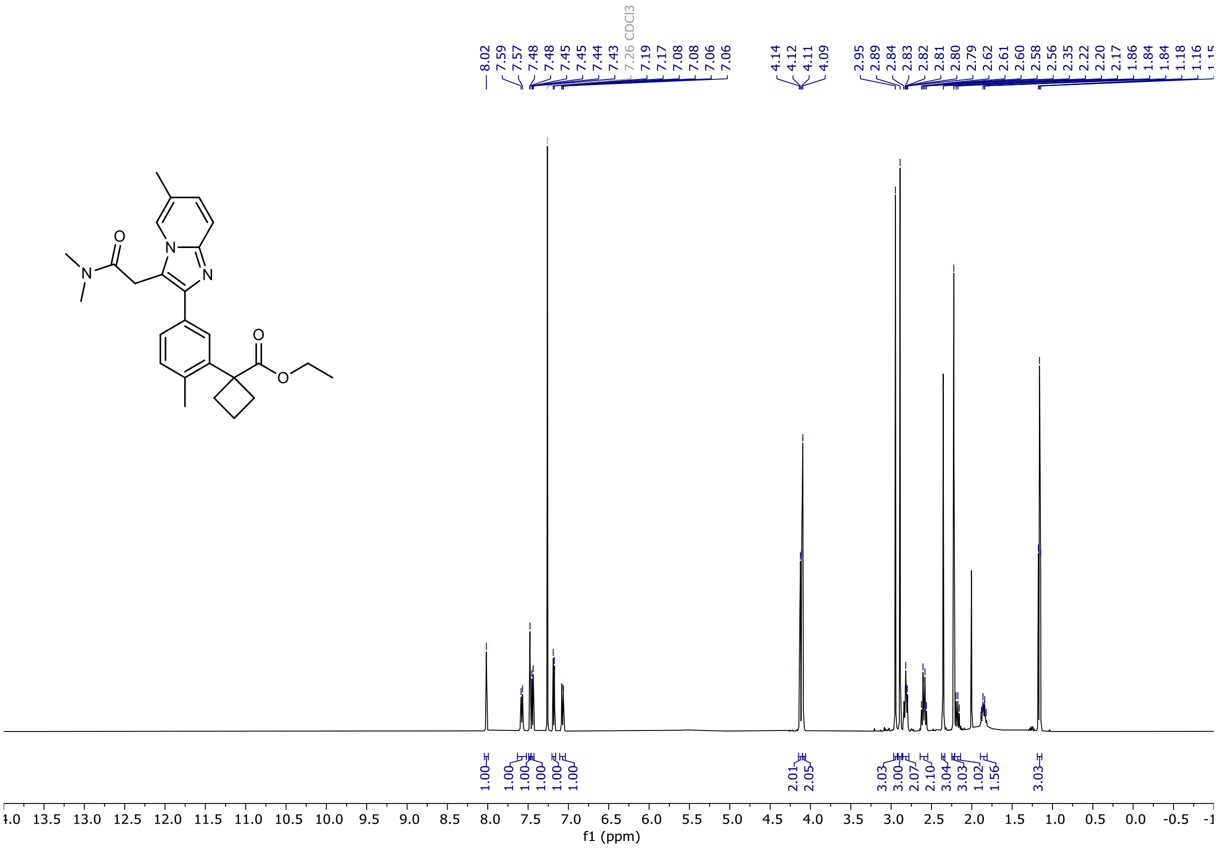

Supplementary Fig. 157. ^{1}H NMR spectra (500 MHz, $CDCl_3$, 298 K) of compound **5g**.

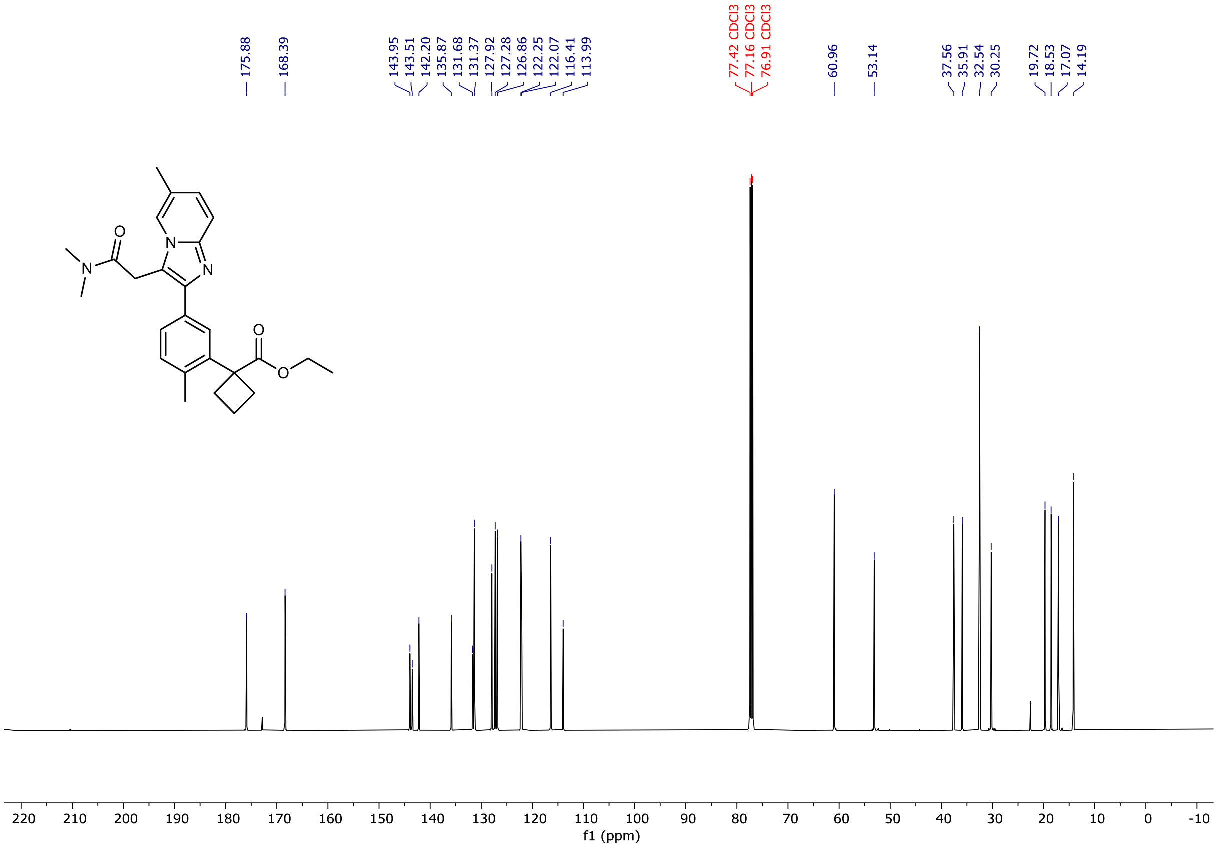

Supplementary Fig. 158. ^{13}C NMR spectra (126 MHz, $CDCl_3$, 298 K) of compound **5g**.

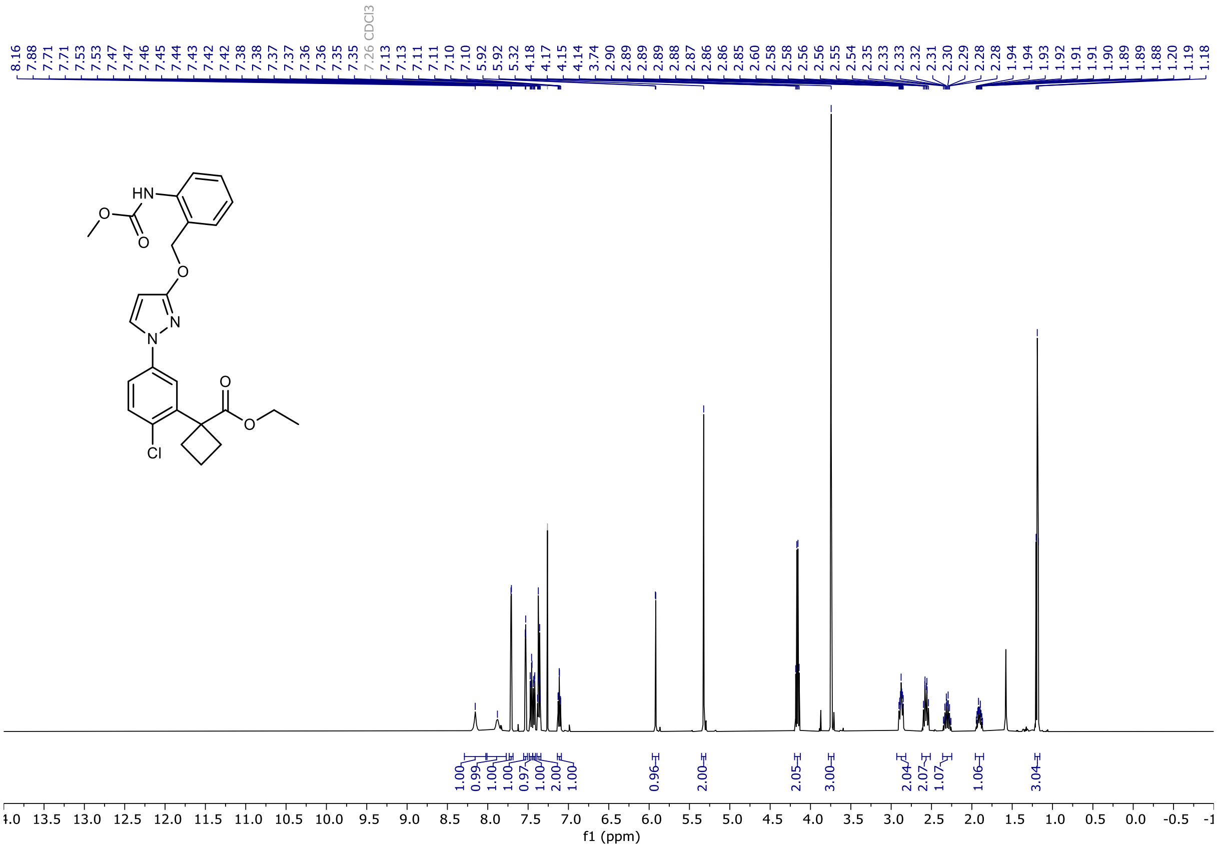

Supplementary Fig. 159. ^{1}H NMR spectra (500 MHz, $CDCl_3$, 298 K) of compound **5h**.

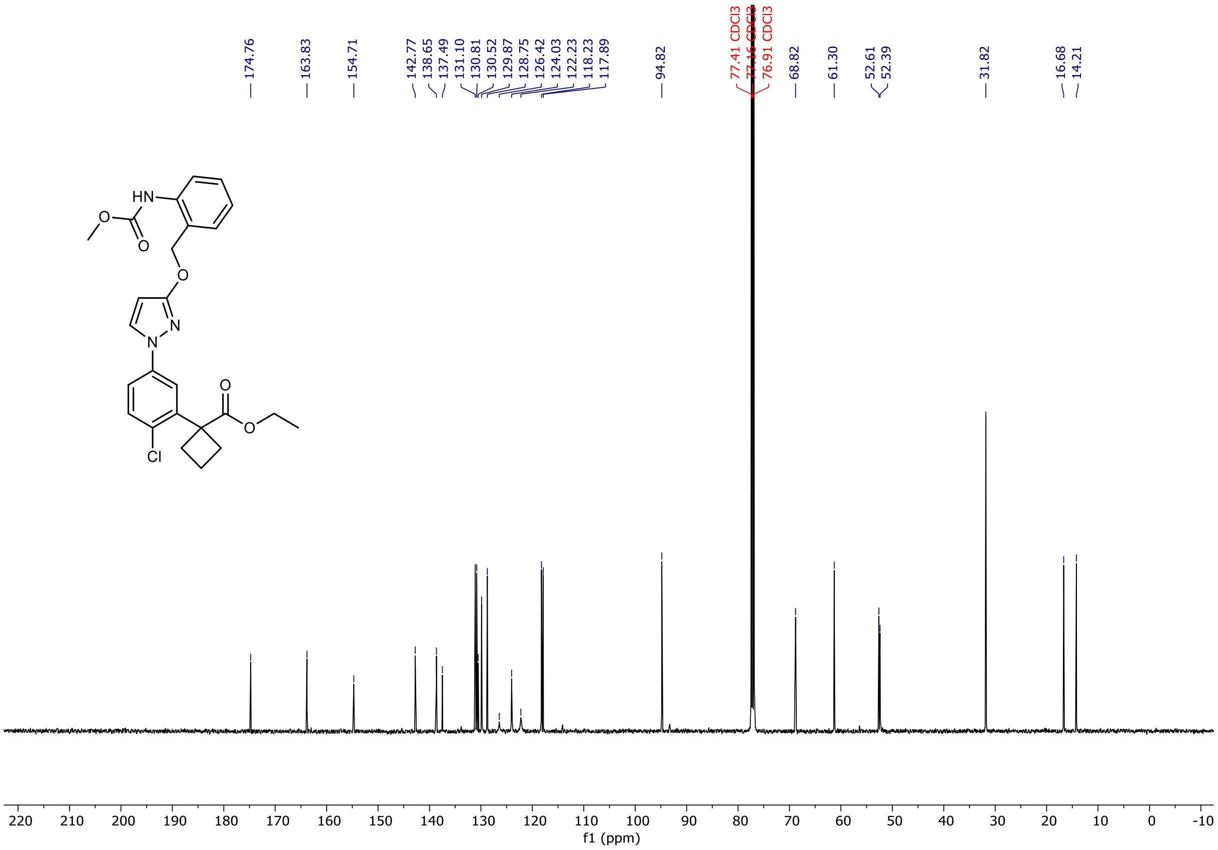

Supplementary Fig. 160. ^{13}C NMR spectra (126 MHz, $CDCl_3$, 298 K) of compound **5h**.

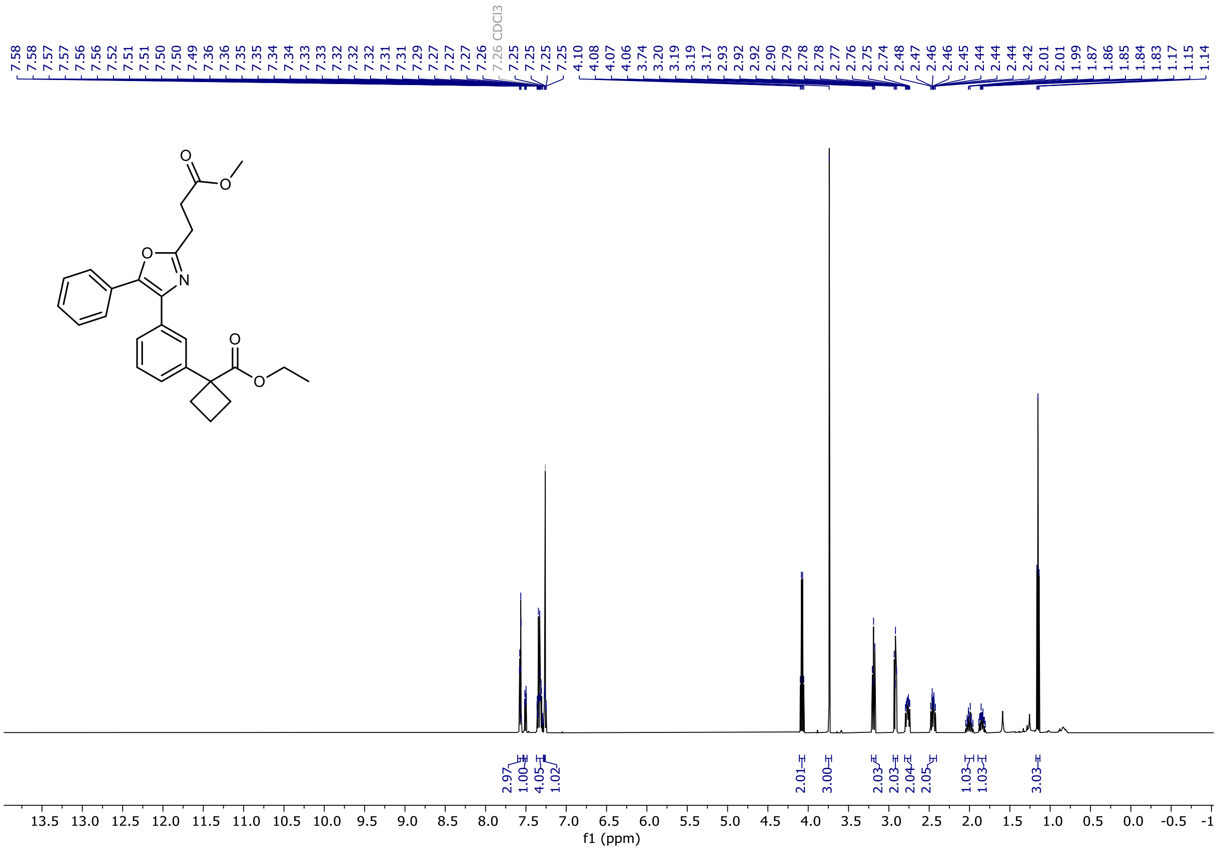

Supplementary Fig. 161. ^{1}H NMR spectra (500 MHz, $CDCl_3$, 298 K) of compound **5i**.

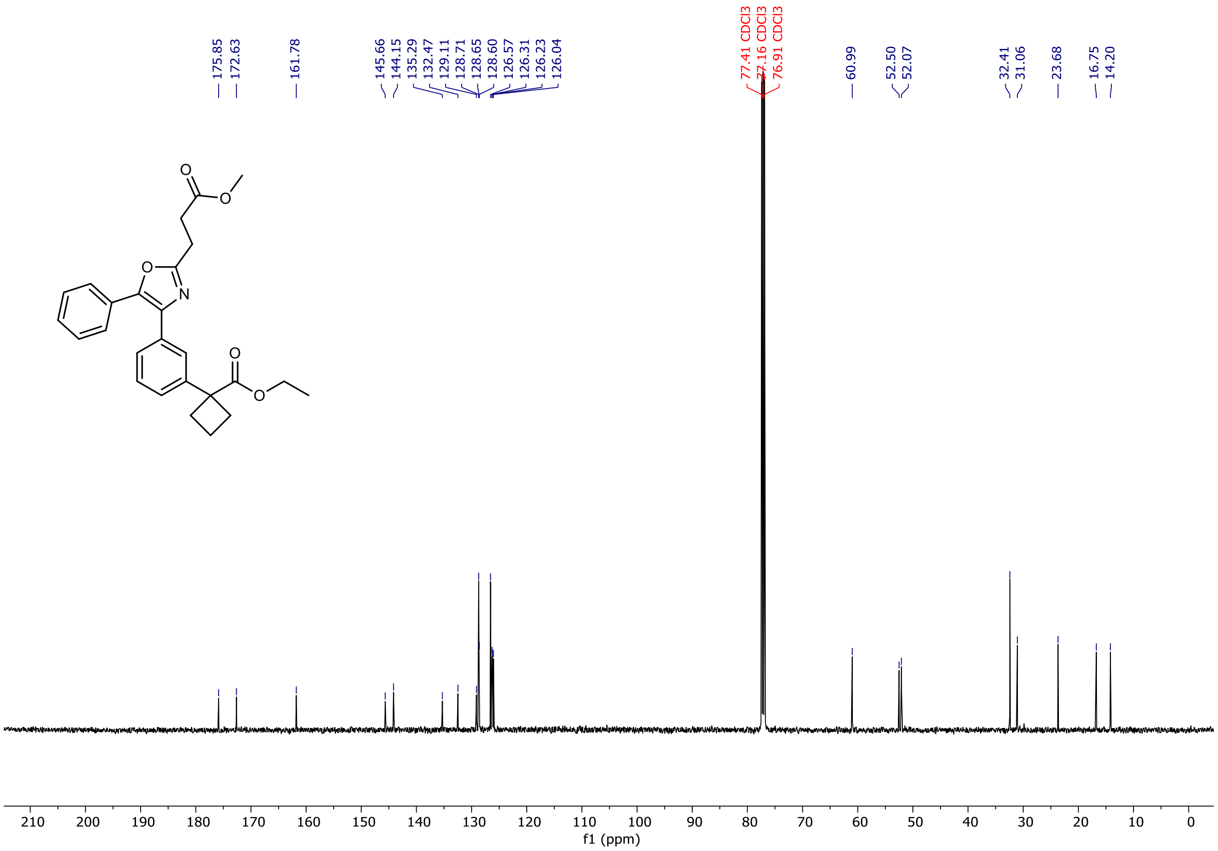

Supplementary Fig. 162. ^{13}C NMR spectra (126 MHz, $CDCl_3$, 298 K) of compound **5i**.

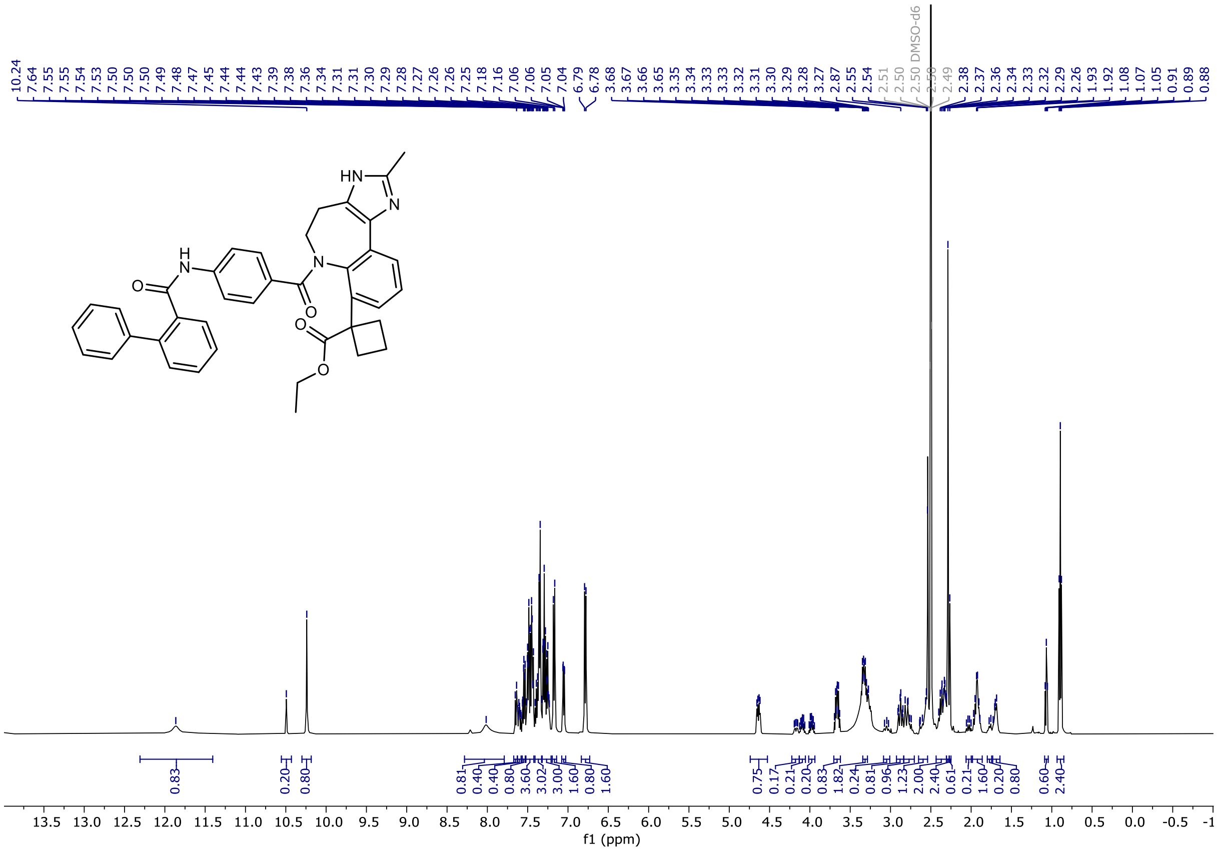

Supplementary Fig. 163. ^{1}H NMR spectra (500 MHz, DMSO-d_6, 298 K) of compound **5j**.

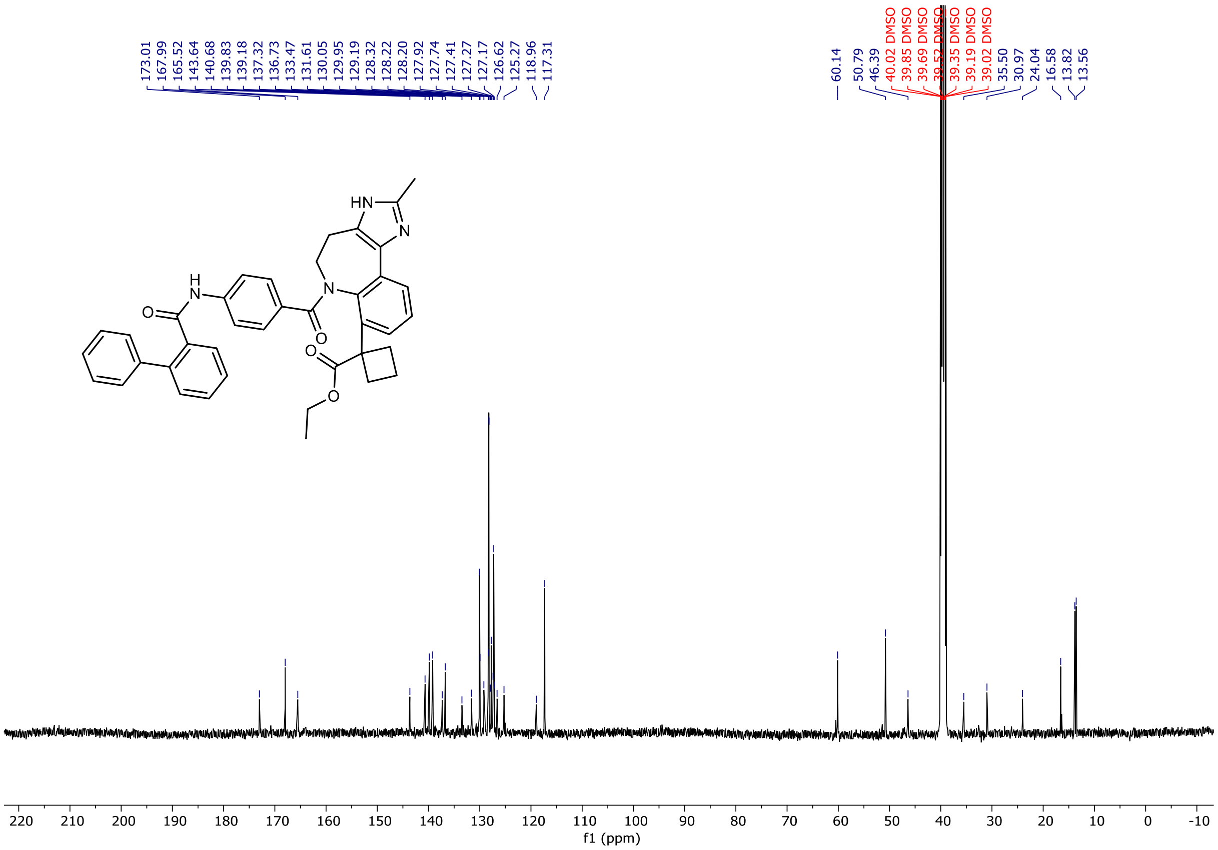

Supplementary Fig. 164. ^{13}C NMR spectra (126 MHz, DMSO-d_6, 298 K) of compound **5j**.

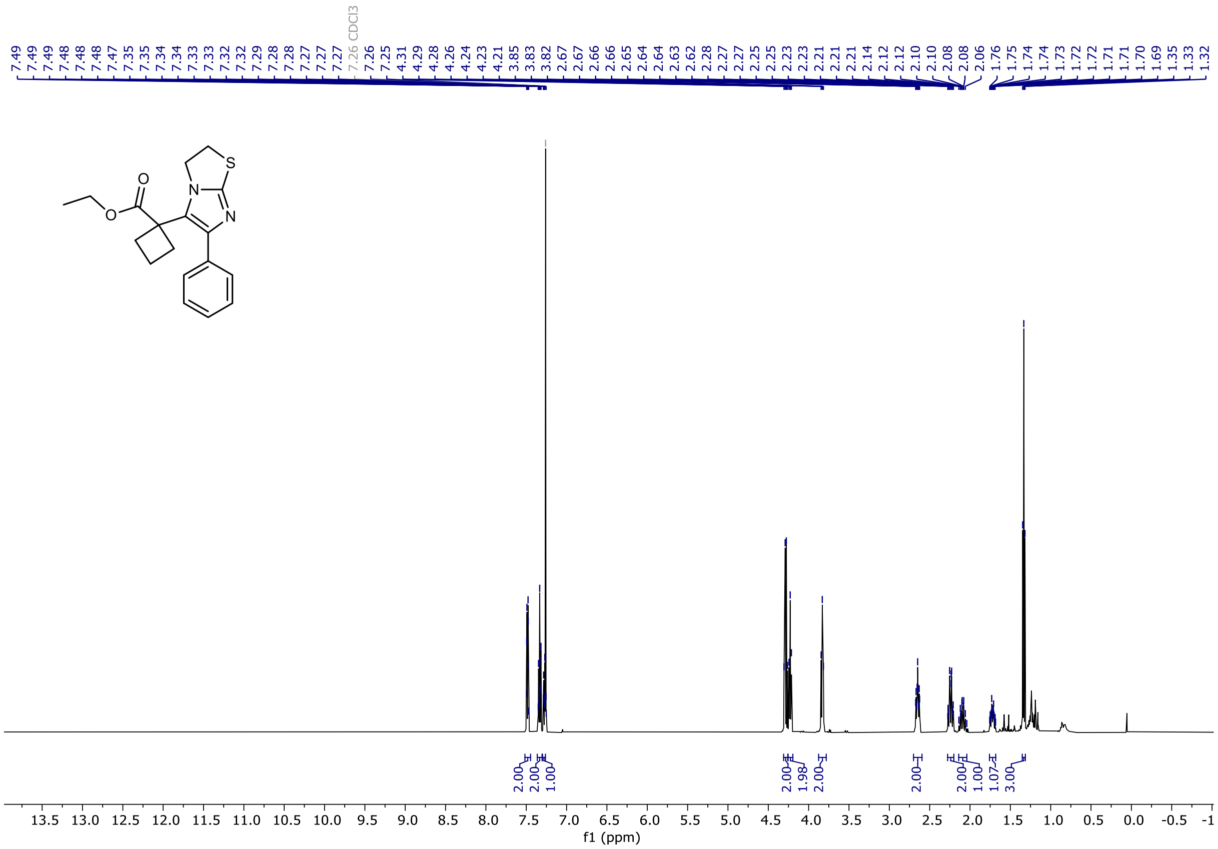

Supplementary Fig. 165. ^{1}H NMR spectra (500 MHz, $CDCl_3$, 298 K) of compound **5k**.

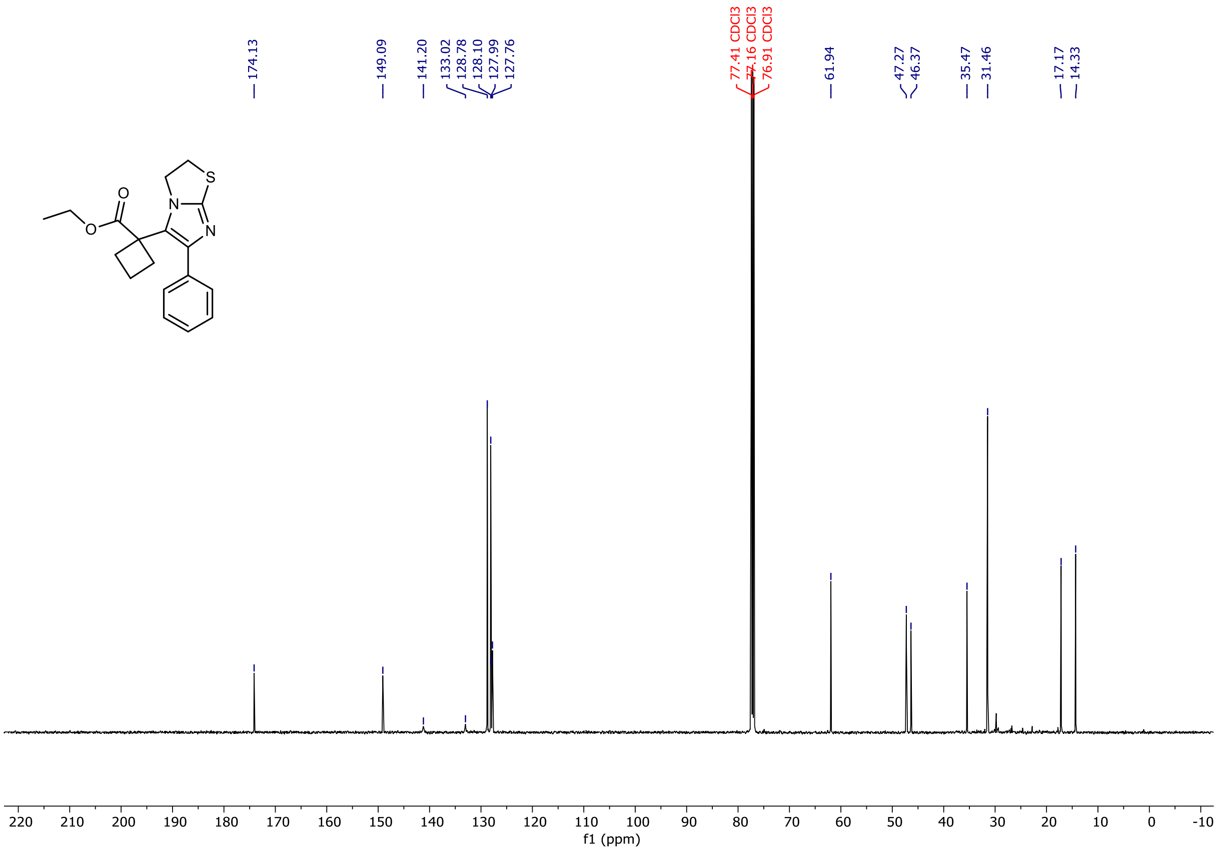

Supplementary Fig. 166. ^{13}C NMR spectra (126 MHz, $CDCl_3$, 298 K) of compound **5k**.

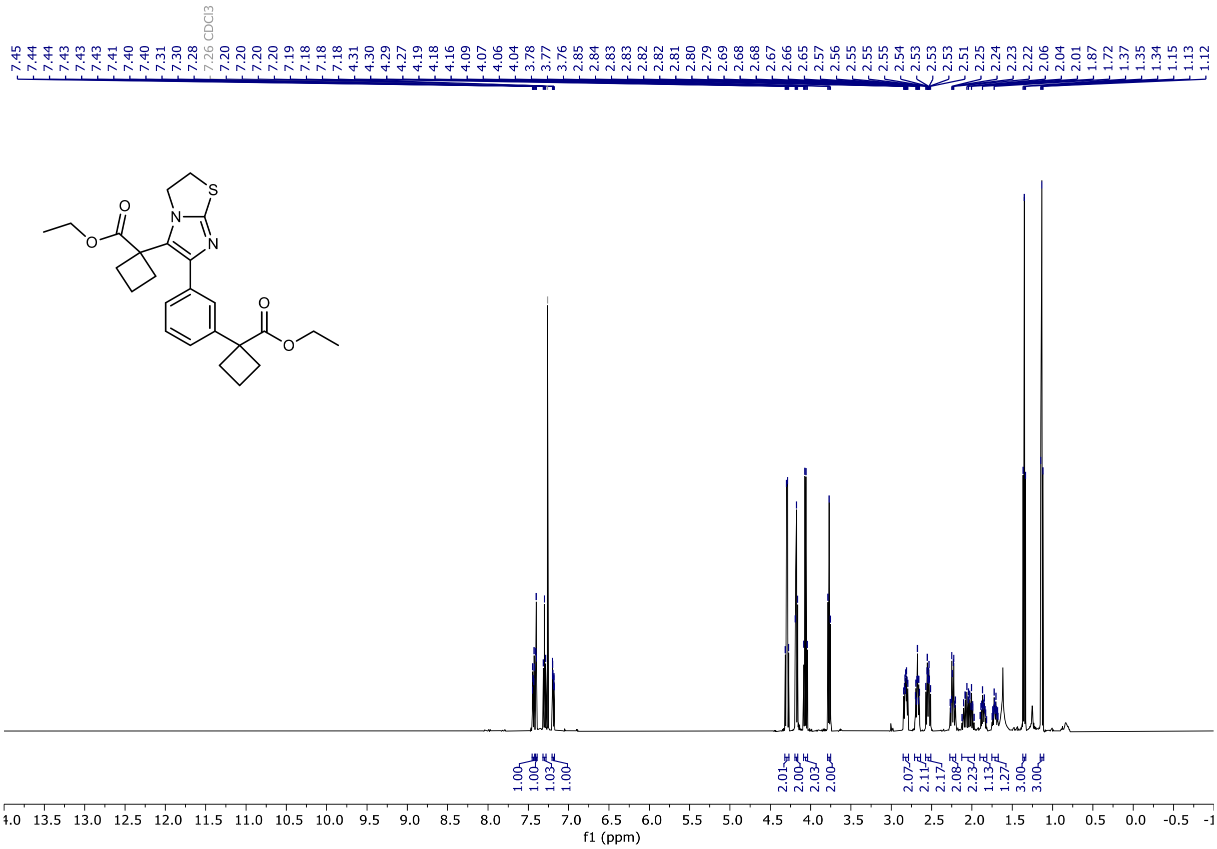

Supplementary Fig. 167. ^{1}H NMR spectra (500 MHz, $CDCl_3$, 298 K) of compound **5k2**.

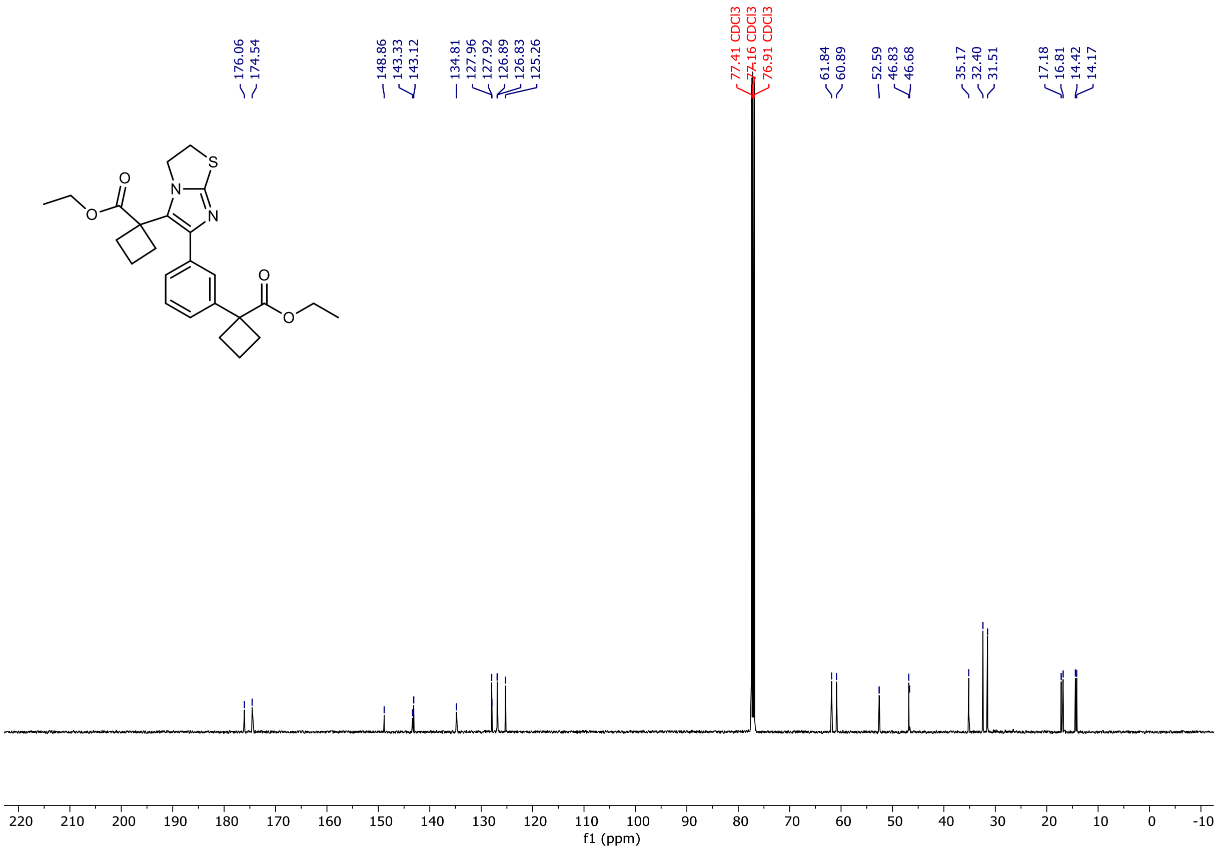

Supplementary Fig. 168. ^{13}C NMR spectra (126 MHz, $CDCl_3$, 298 K) of compound **5k2**.

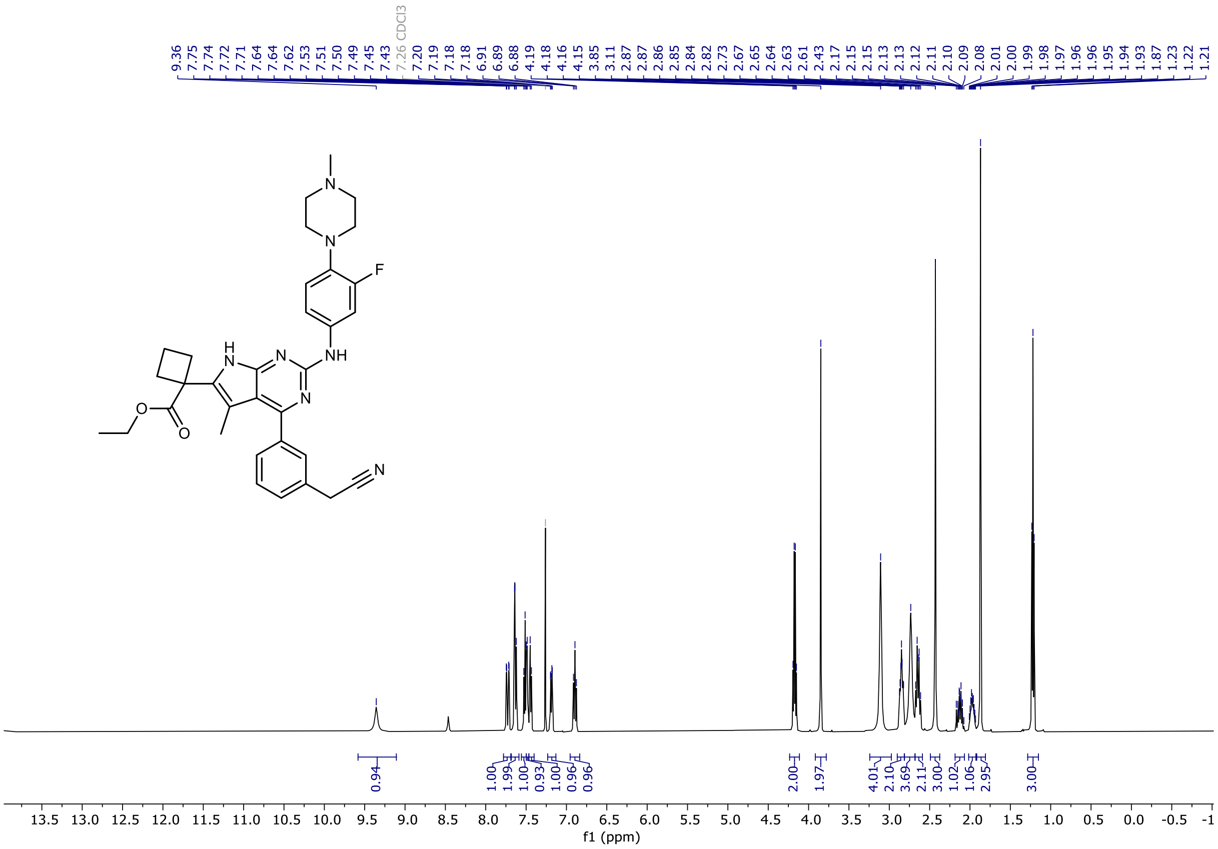

Supplementary Fig. 169. ^{1}H NMR spectra (500 MHz, $CDCl_3$, 298 K) of compound **5l**.

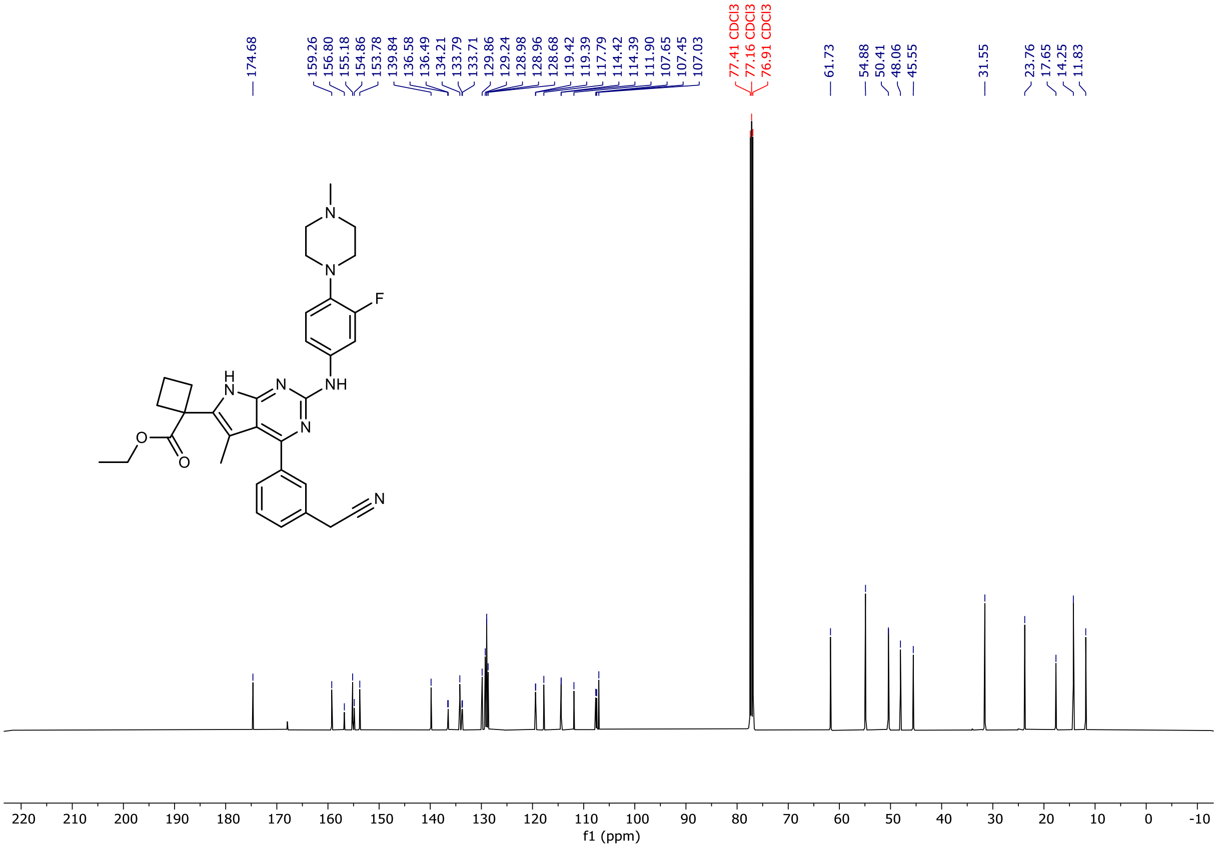

Supplementary Fig. 170. ^{13}C NMR spectra (126 MHz, $CDCl_3$, 298 K) of compound **5l**.

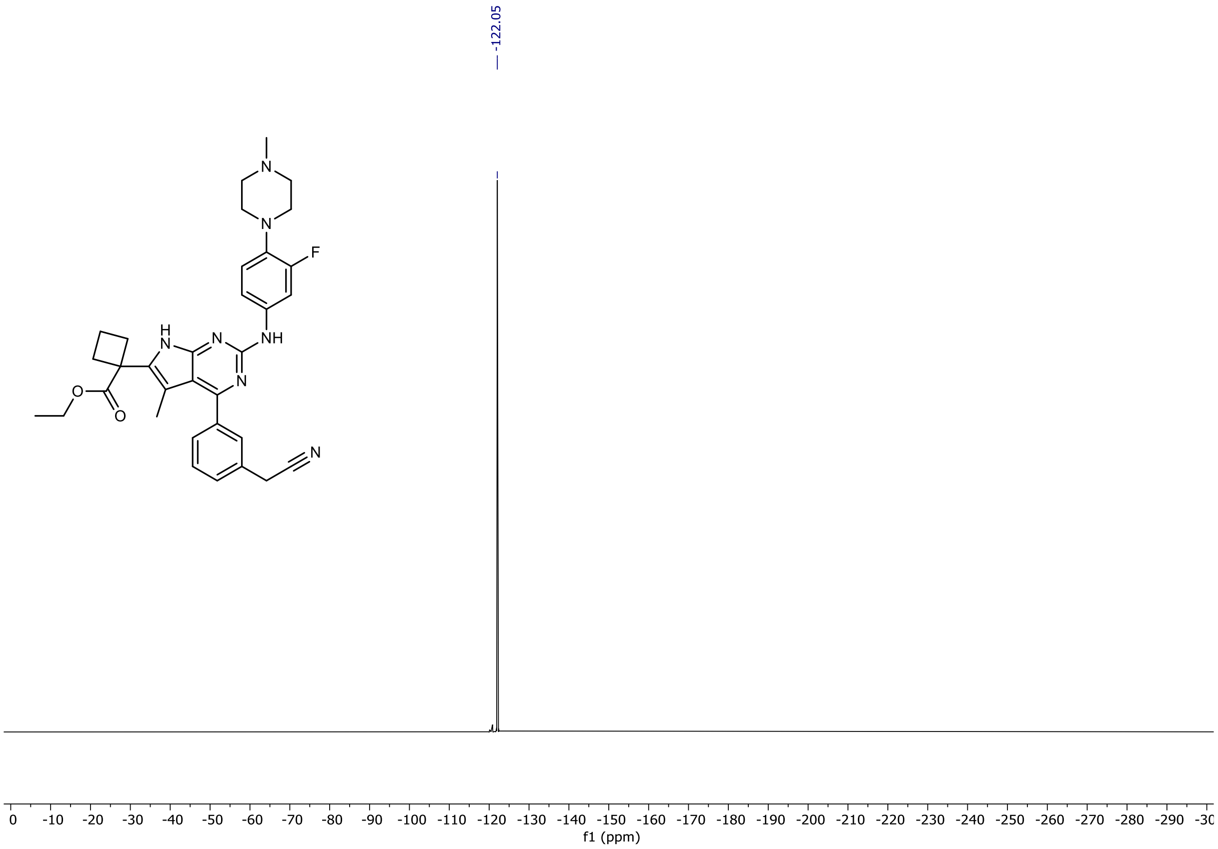

Supplementary Fig. 171. ^{19}F NMR spectra (471 MHz, $CDCl_3$, 298 K) of compound **5l**.

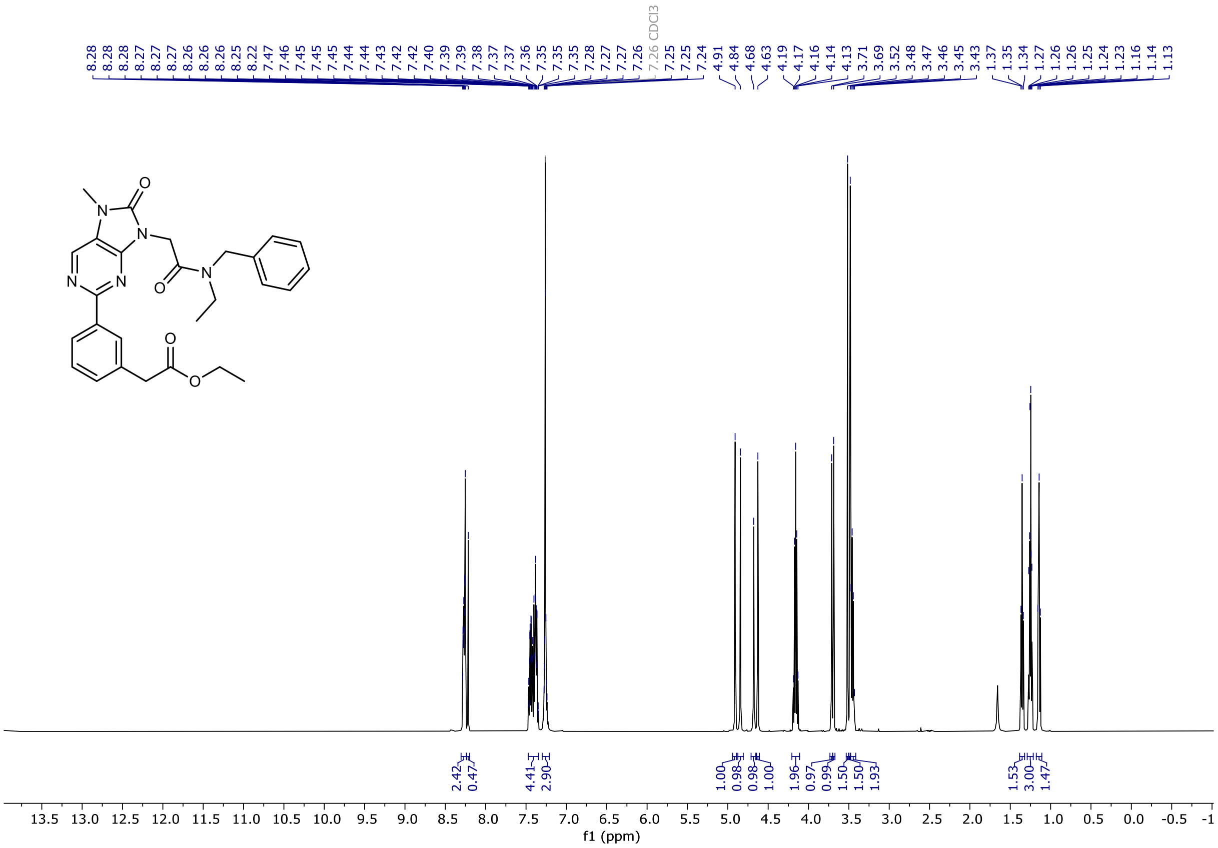

Supplementary Fig. 172. ^{1}H NMR spectra (500 MHz, $CDCl_3$, 298 K) of compound **6a**.

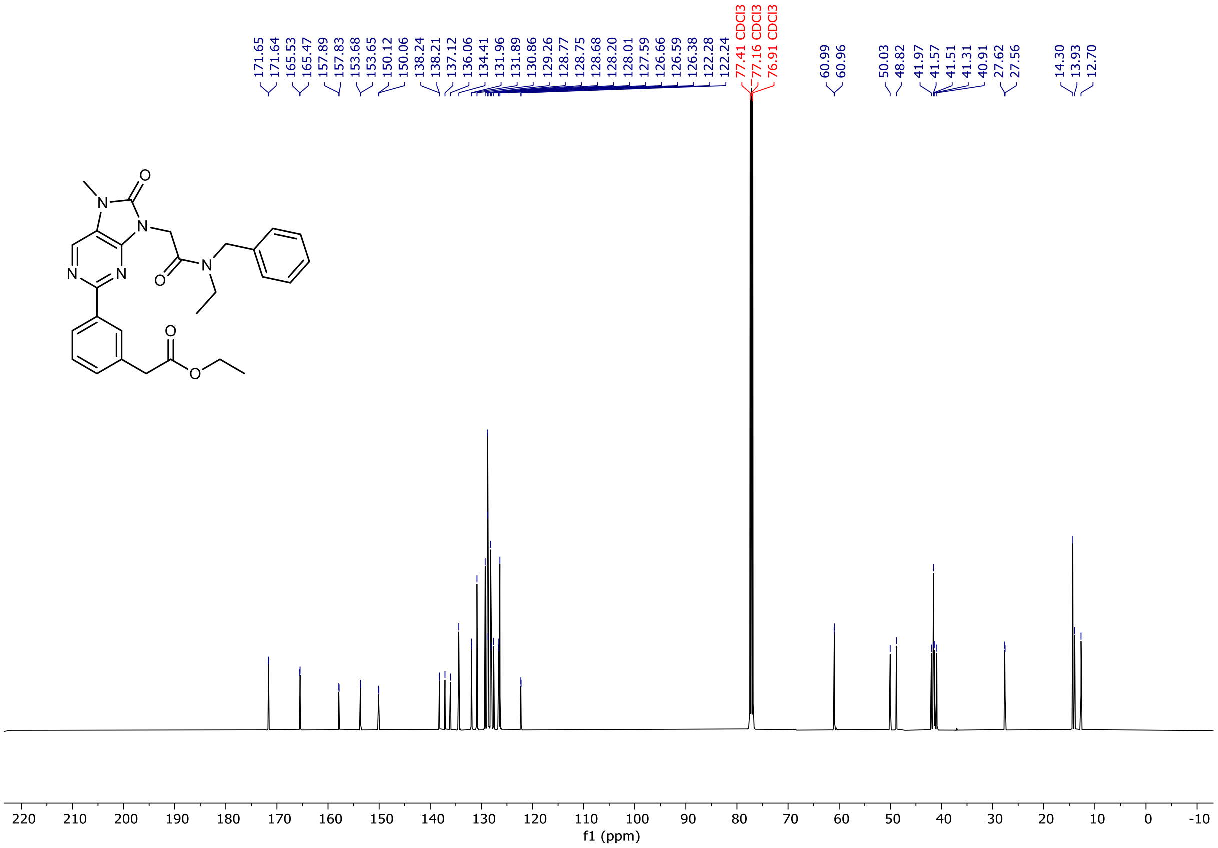

Supplementary Fig. 173. ^{13}C NMR spectra (126 MHz, $CDCl_3$, 298 K) of compound **6a**.

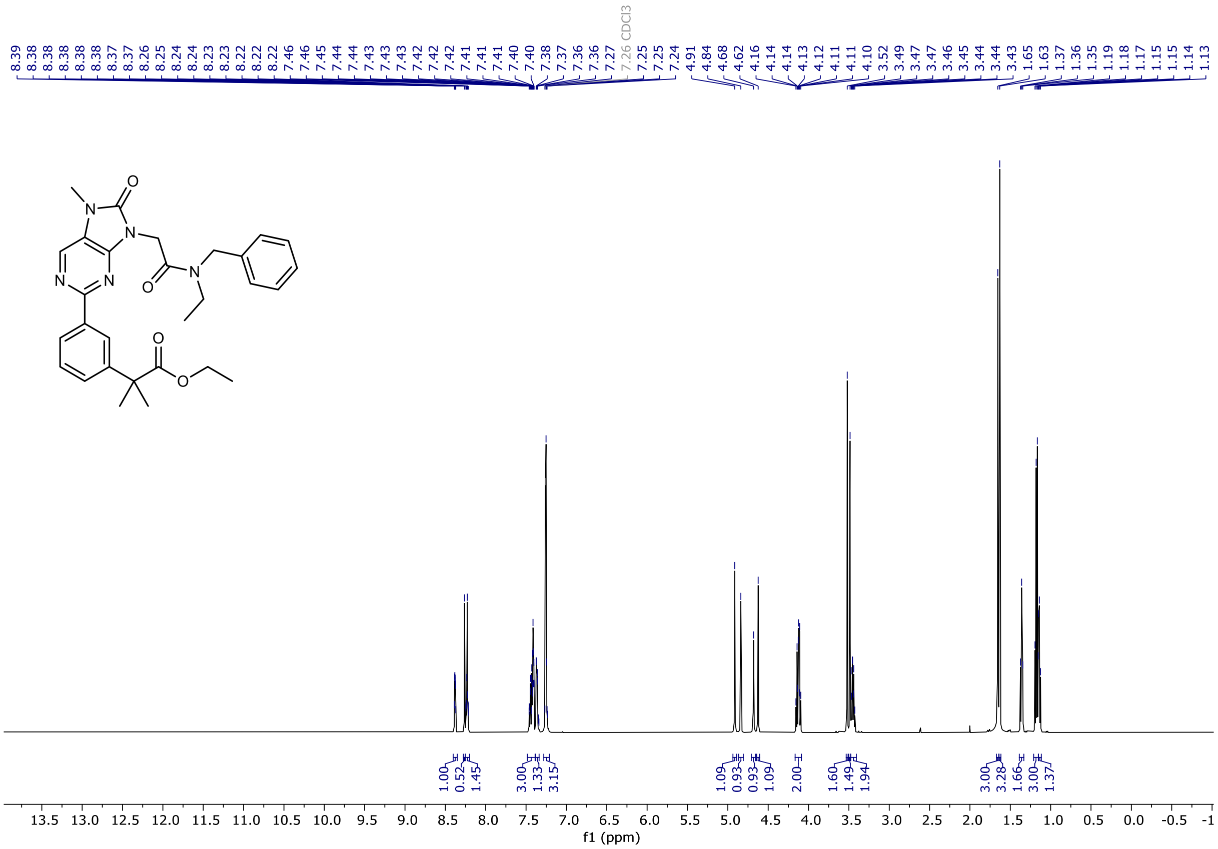

Supplementary Fig. 174. ^{1}H NMR spectra (500 MHz, $CDCl_3$, 298 K) of compound **6b**.

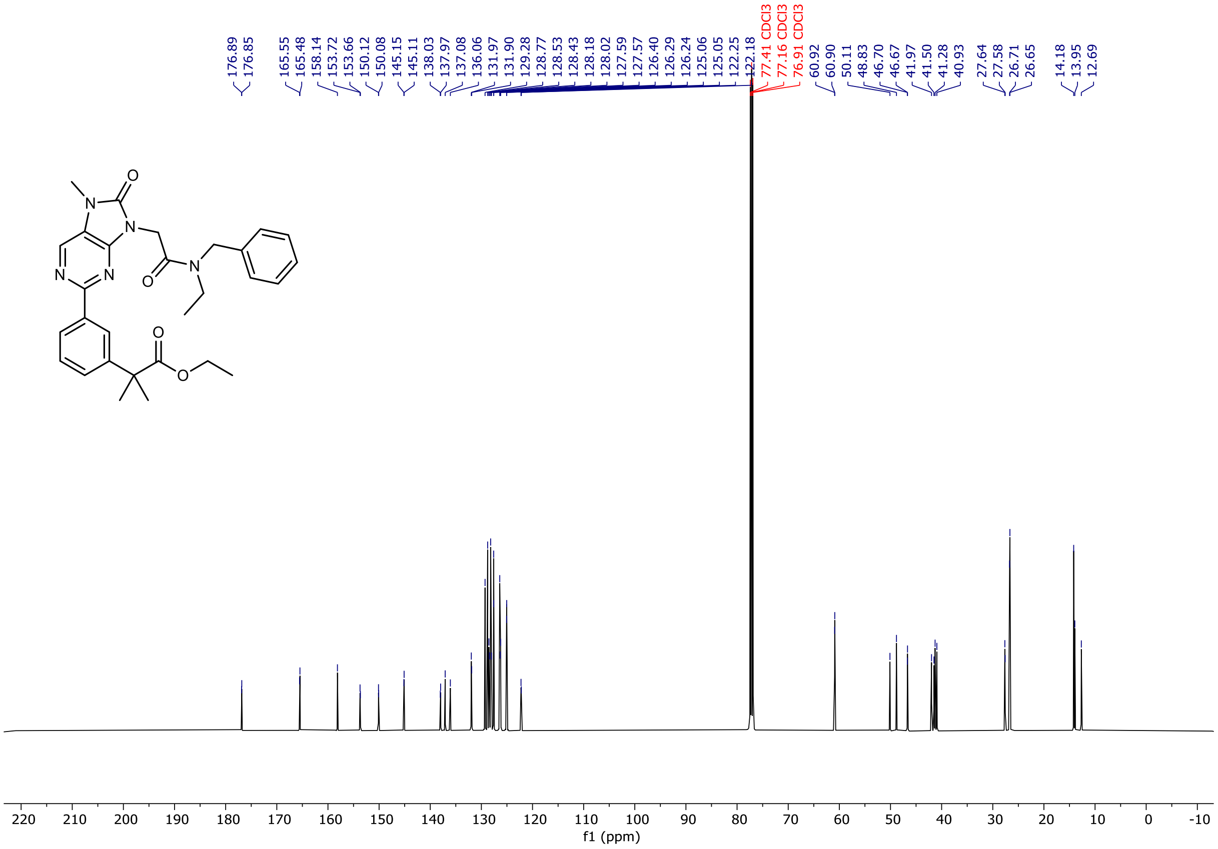

Supplementary Fig. 175. ^{13}C NMR spectra (126 MHz, $CDCl_3$, 298 K) of compound **6b**.

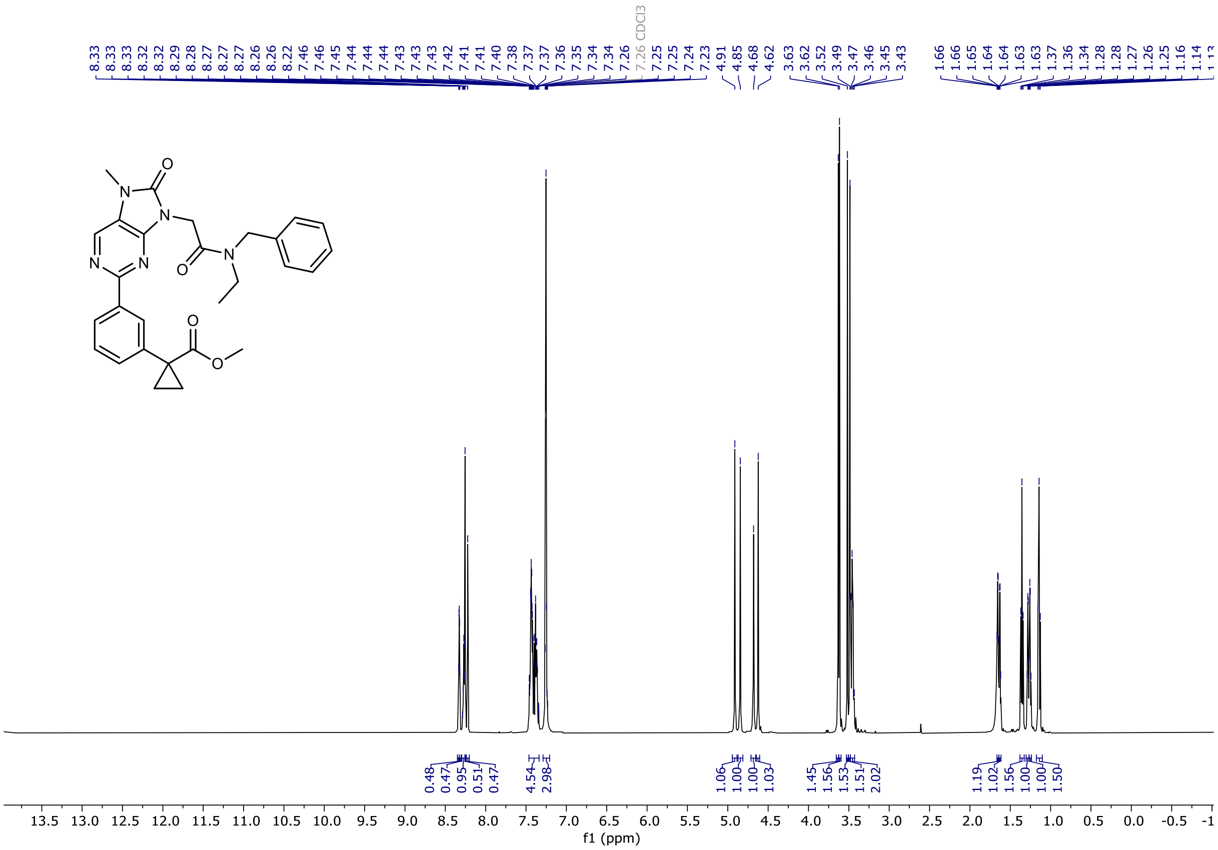

Supplementary Fig. 176. ^{1}H NMR spectra (500 MHz, $CDCl_3$, 298 K) of compound **6c**.

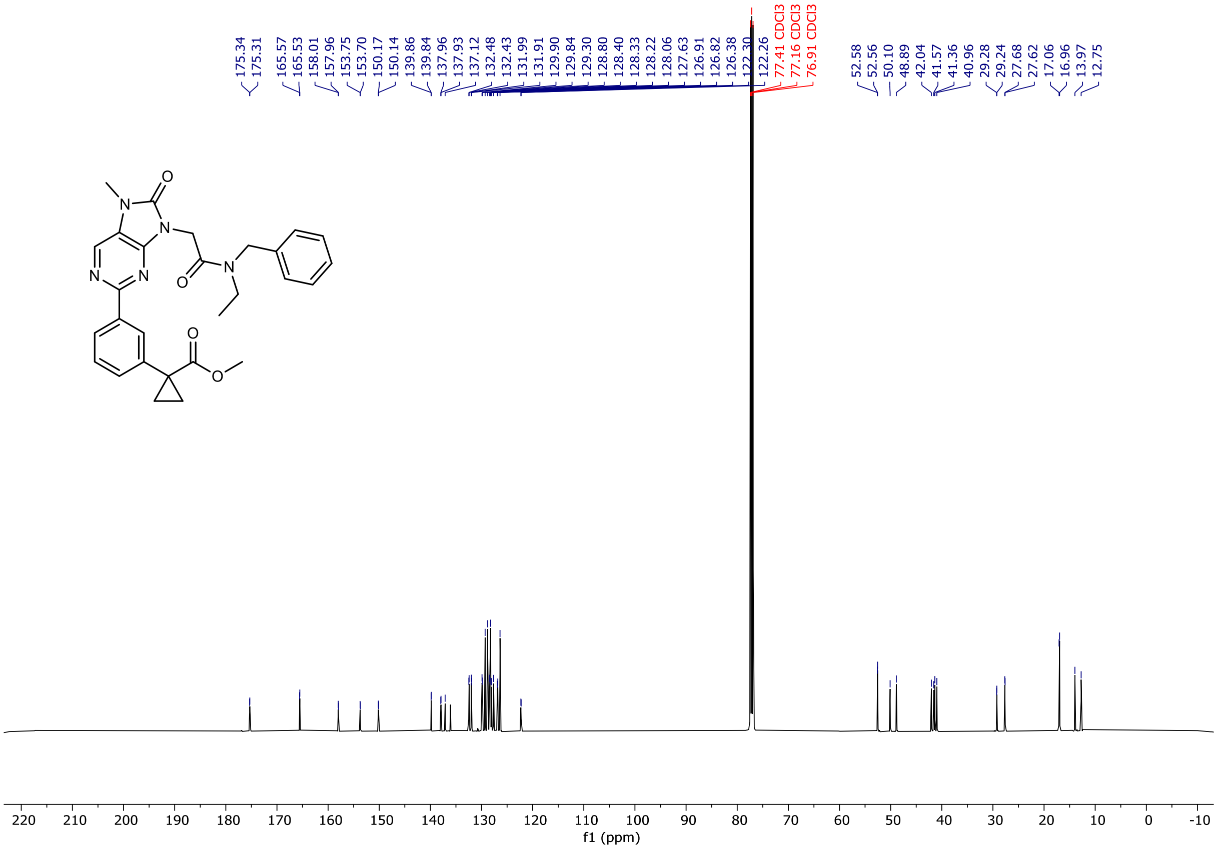

Supplementary Fig. 177. ^{13}C NMR spectra (126 MHz, $CDCl_3$, 298 K) of compound **6c**.

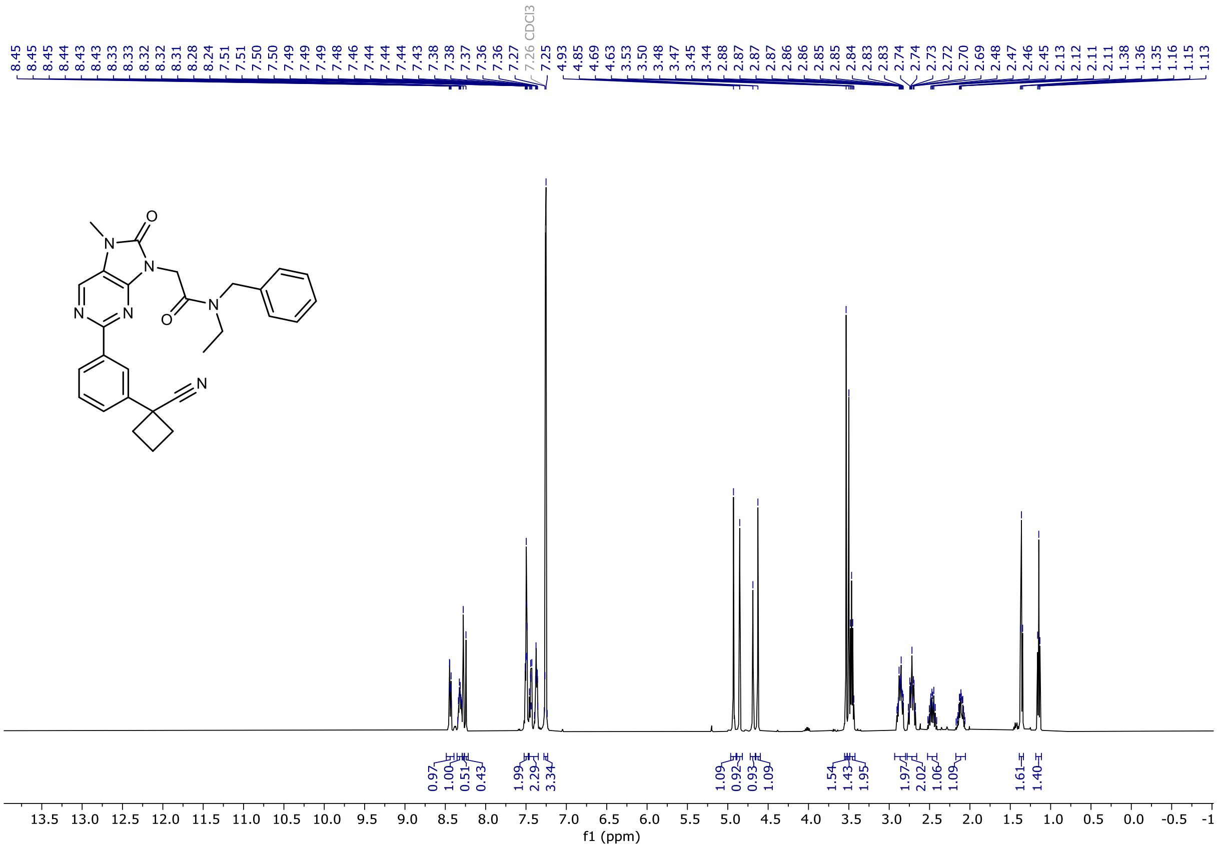

Supplementary Fig. 178. ^{1}H NMR spectra (500 MHz, $CDCl_3$, 298 K) of compound **6d**.

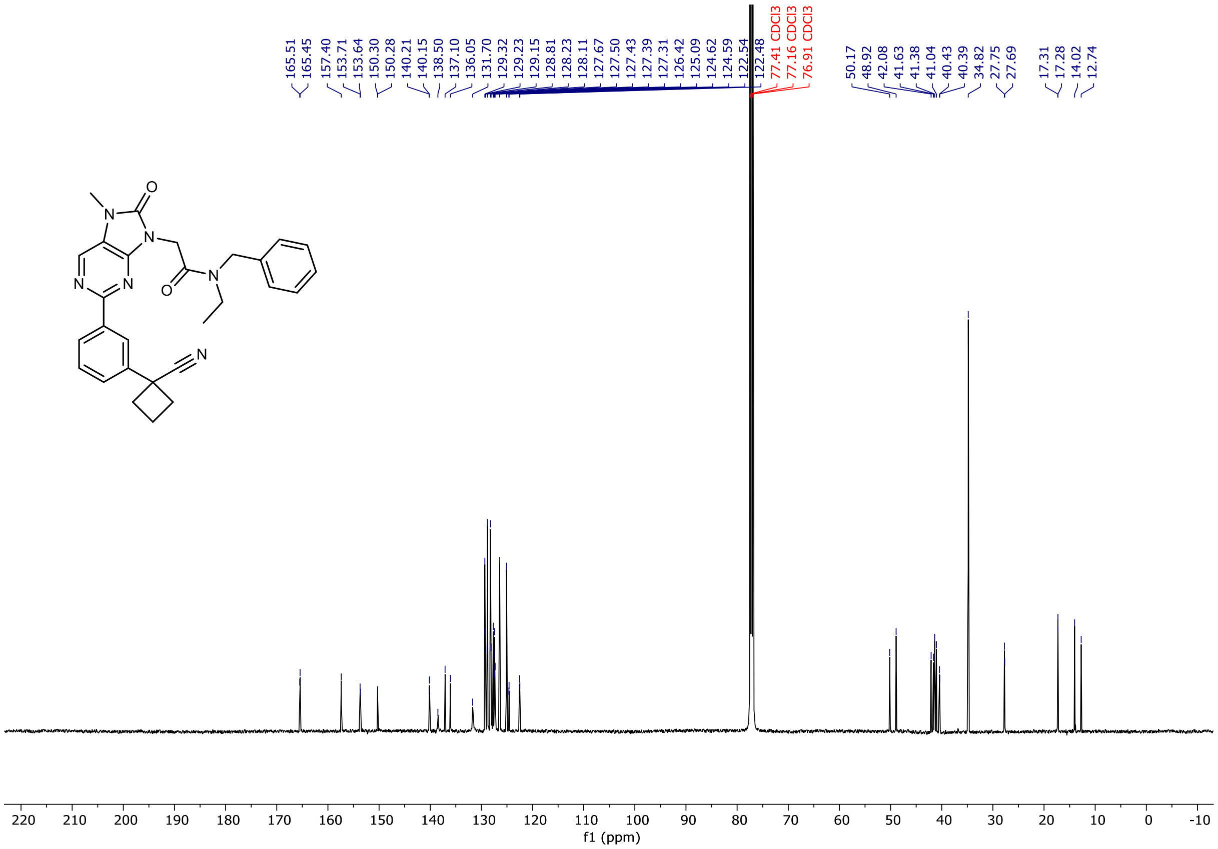

Supplementary Fig. 179. ^{13}C NMR spectra (126 MHz, $CDCl_3$, 298 K) of compound **6d**.

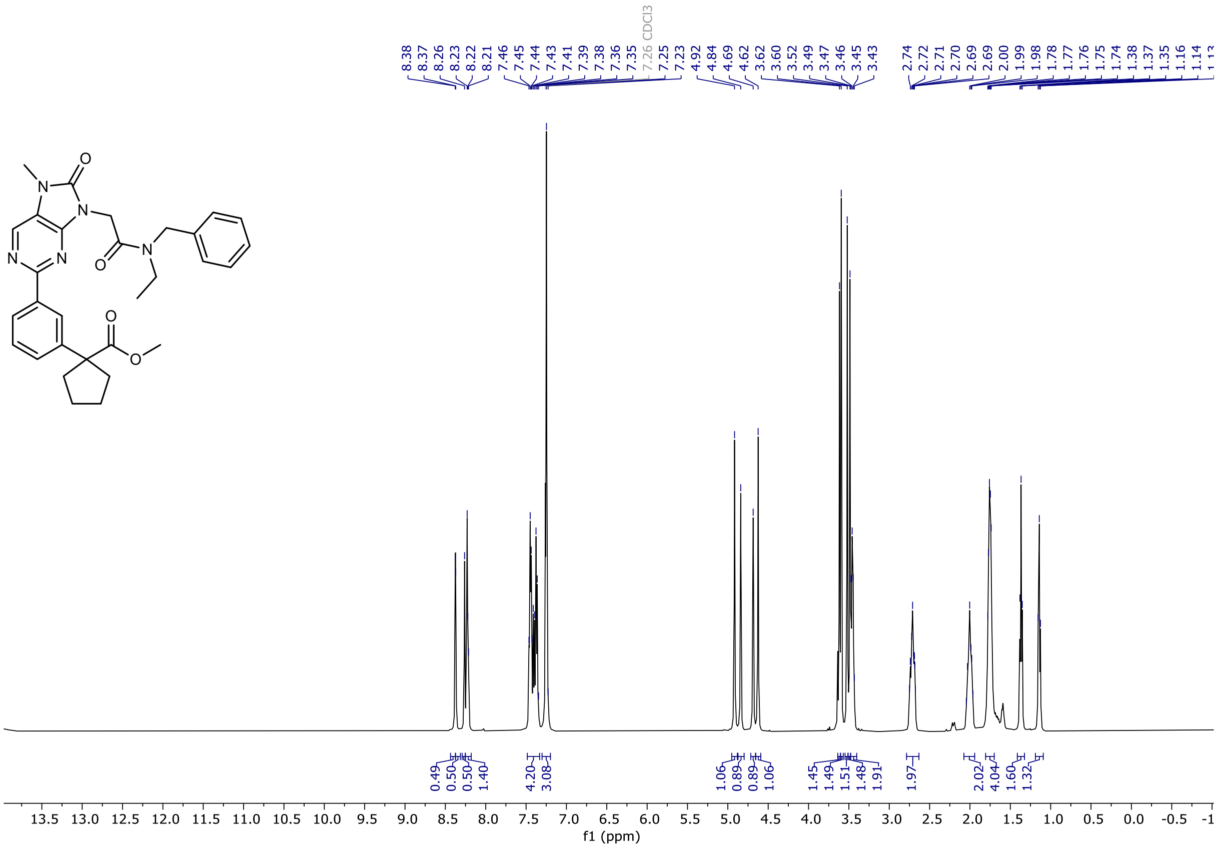

Supplementary Fig. 180. ^{1}H NMR spectra (500 MHz, $CDCl_3$, 298 K) of compound **6e**.

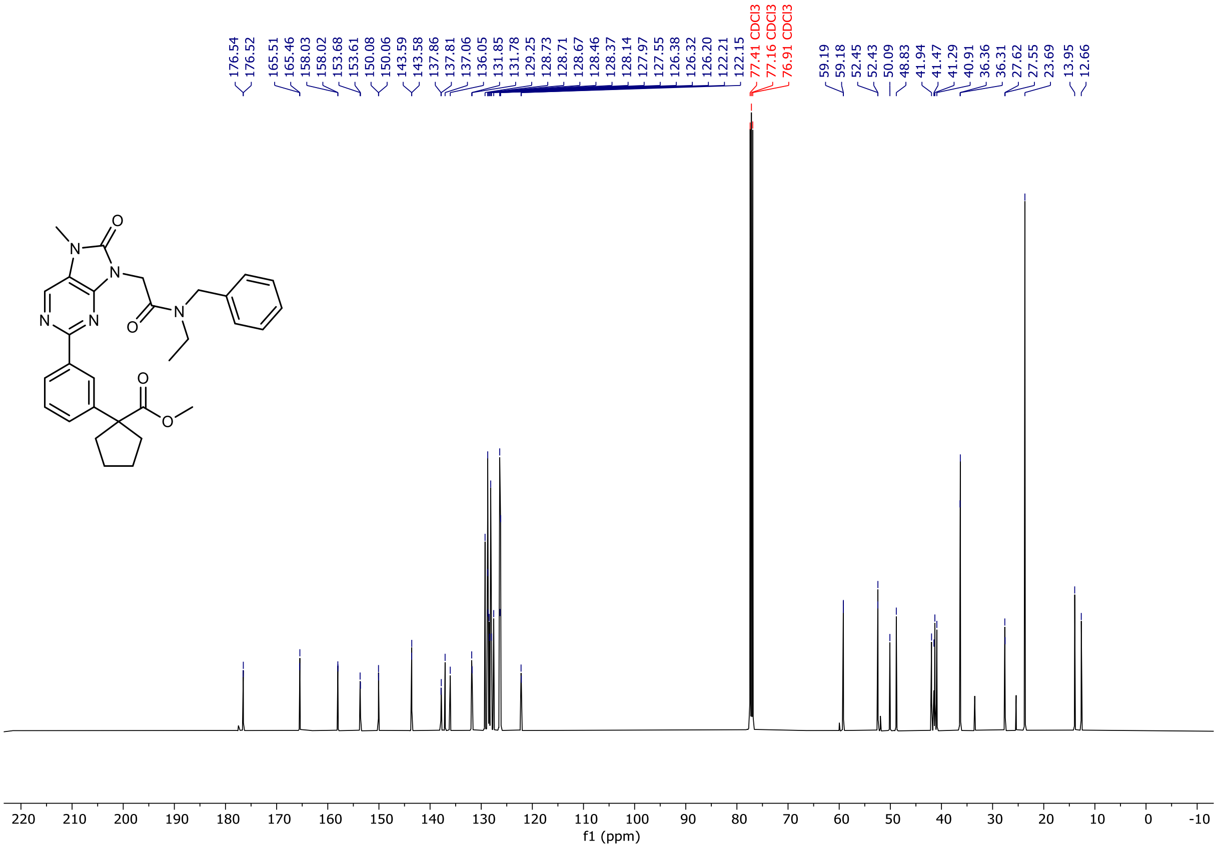

Supplementary Fig. 181. ^{13}C NMR spectra (126 MHz, $CDCl_3$, 298 K) of compound **6e**.

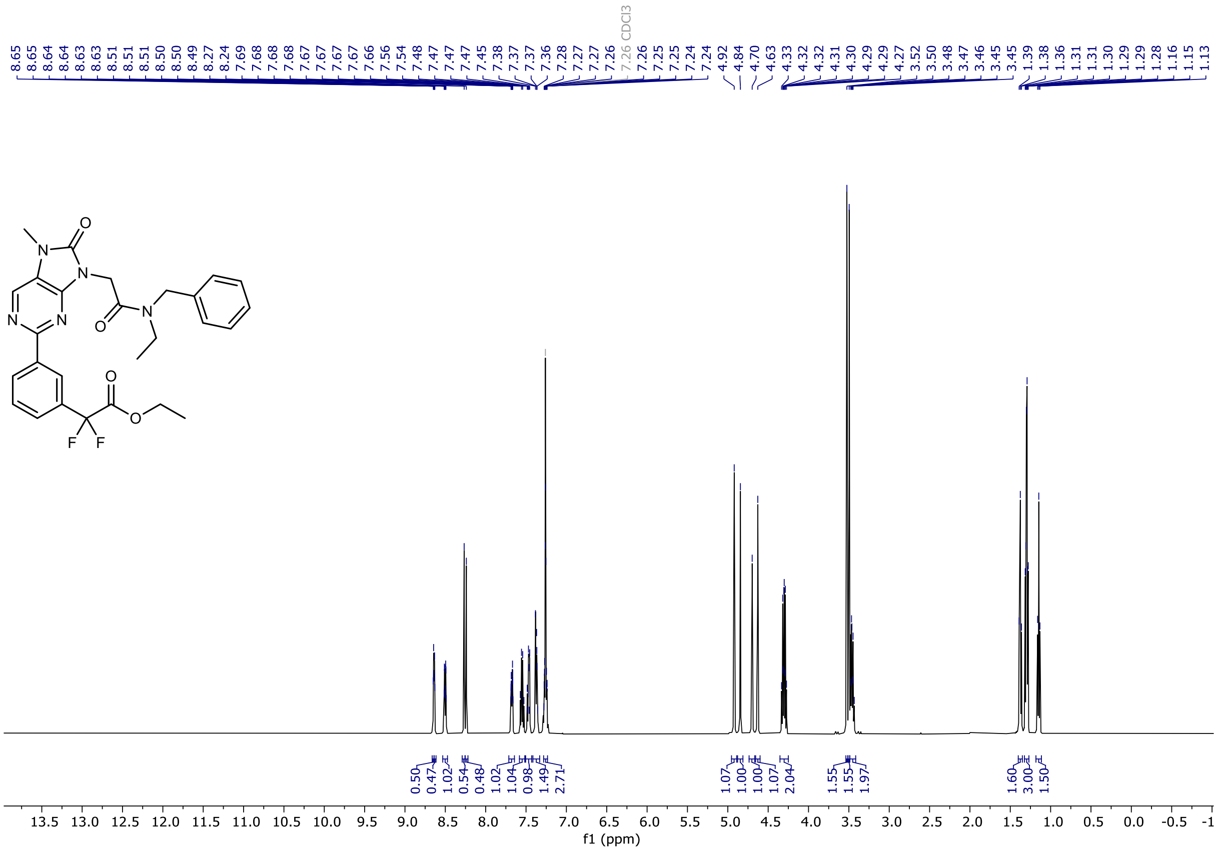

Supplementary Fig. 182. ^{1}H NMR spectra (500 MHz, $CDCl_3$, 298 K) of compound **6f**.

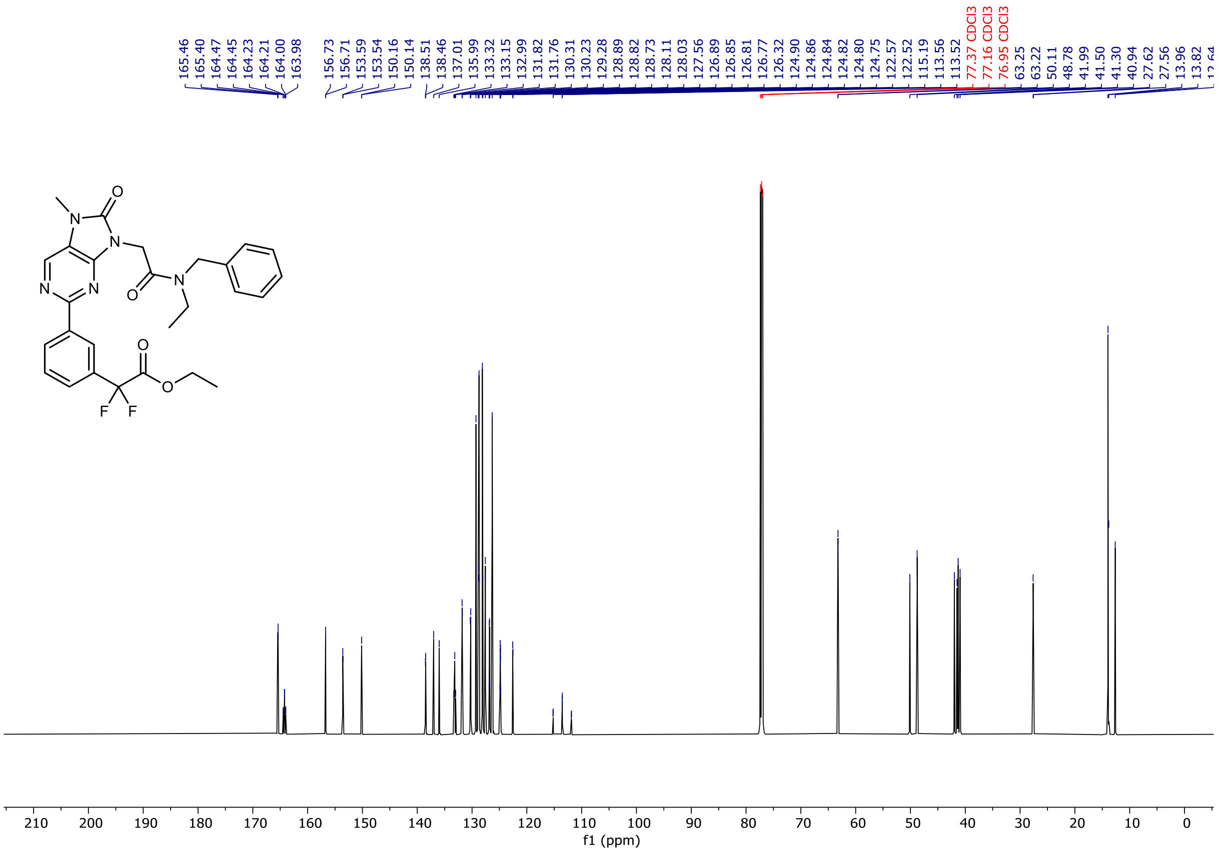

Supplementary Fig. 183. ^{13}C NMR spectra (126 MHz, $CDCl_3$, 298 K) of compound **6f**.

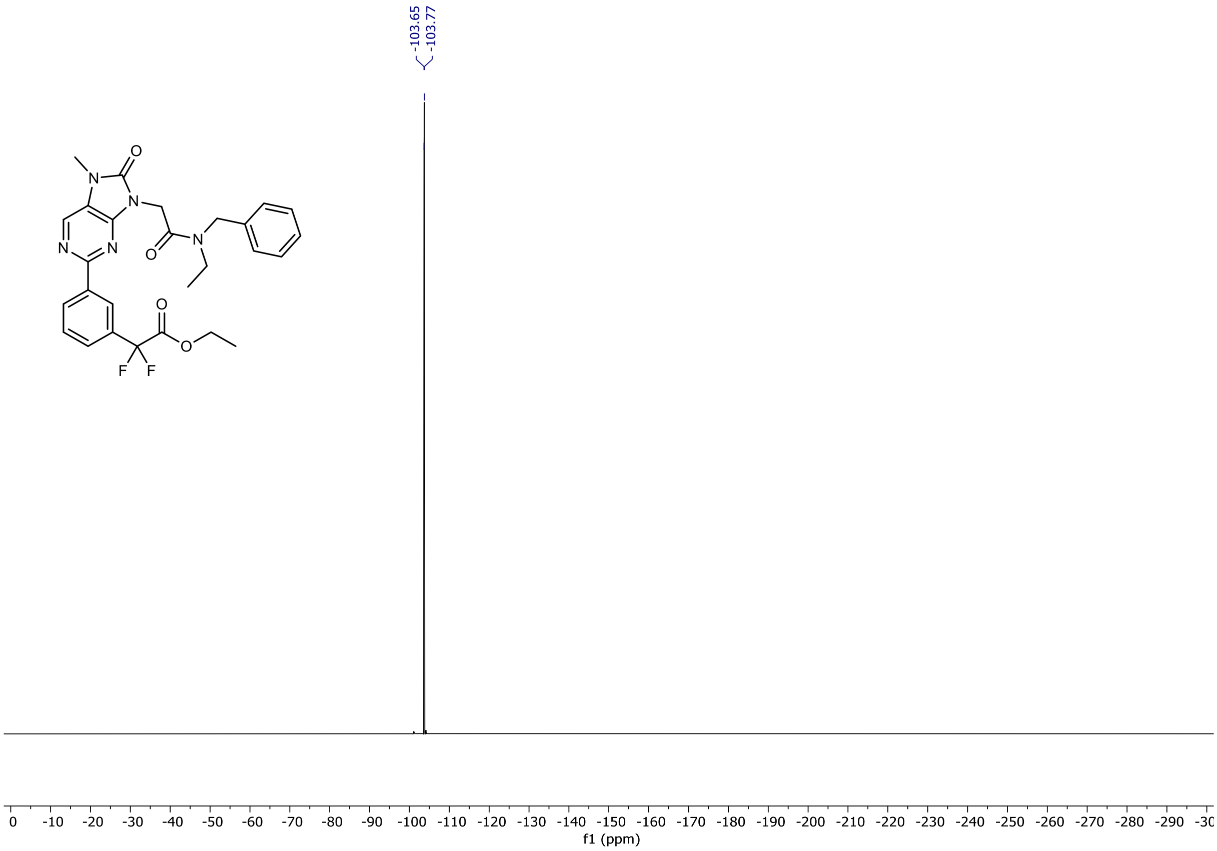

Supplementary Fig. 184. ^{19}F NMR spectra (471 MHz, $CDCl_3$, 298 K) of compound **6f**.

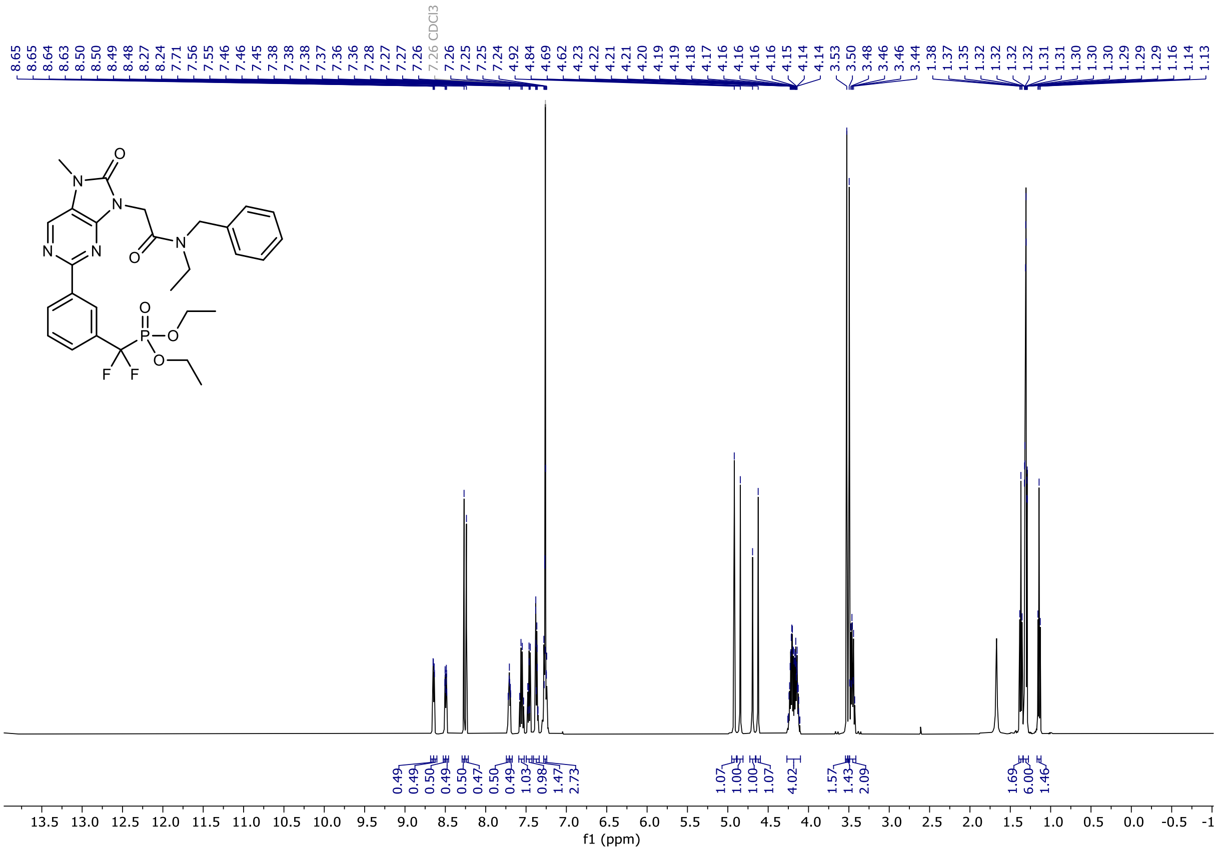

Supplementary Fig. 185. ^{1}H NMR spectra (500 MHz, $CDCl_3$, 298 K) of compound **6g**.

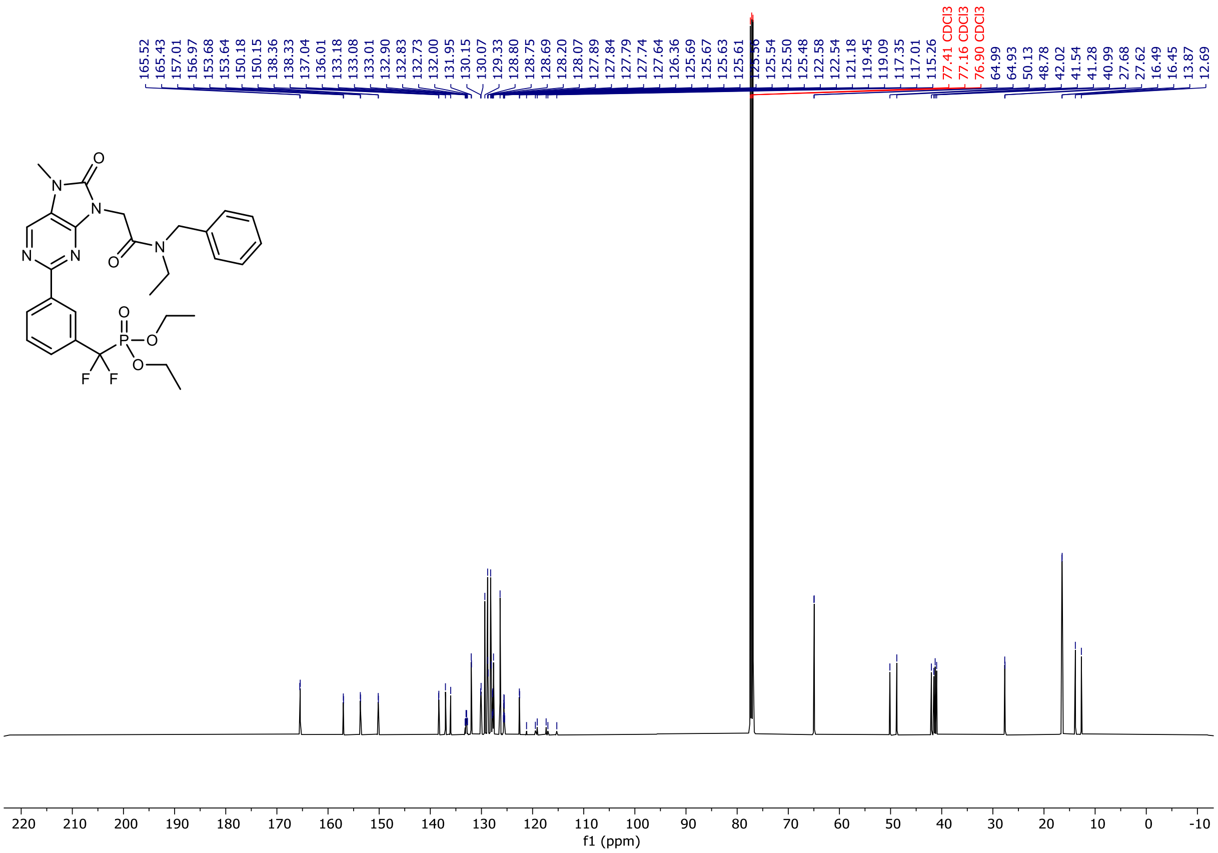

Supplementary Fig. 186. ^{13}C NMR spectra (126 MHz, $CDCl_3$, 298 K) of compound **6g**.

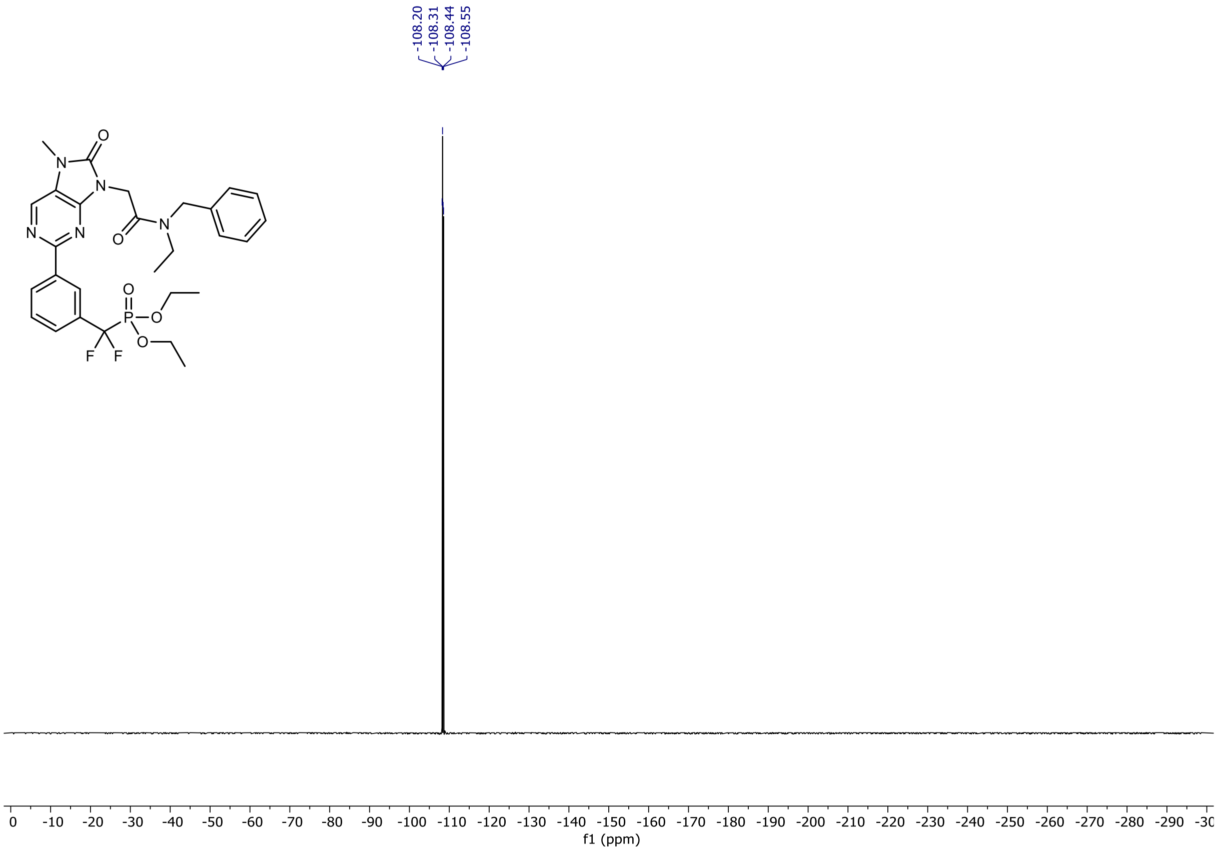

Supplementary Fig. 187. ^{19}F NMR spectra (471 MHz, $CDCl_3$, 298 K) of compound **6g**.

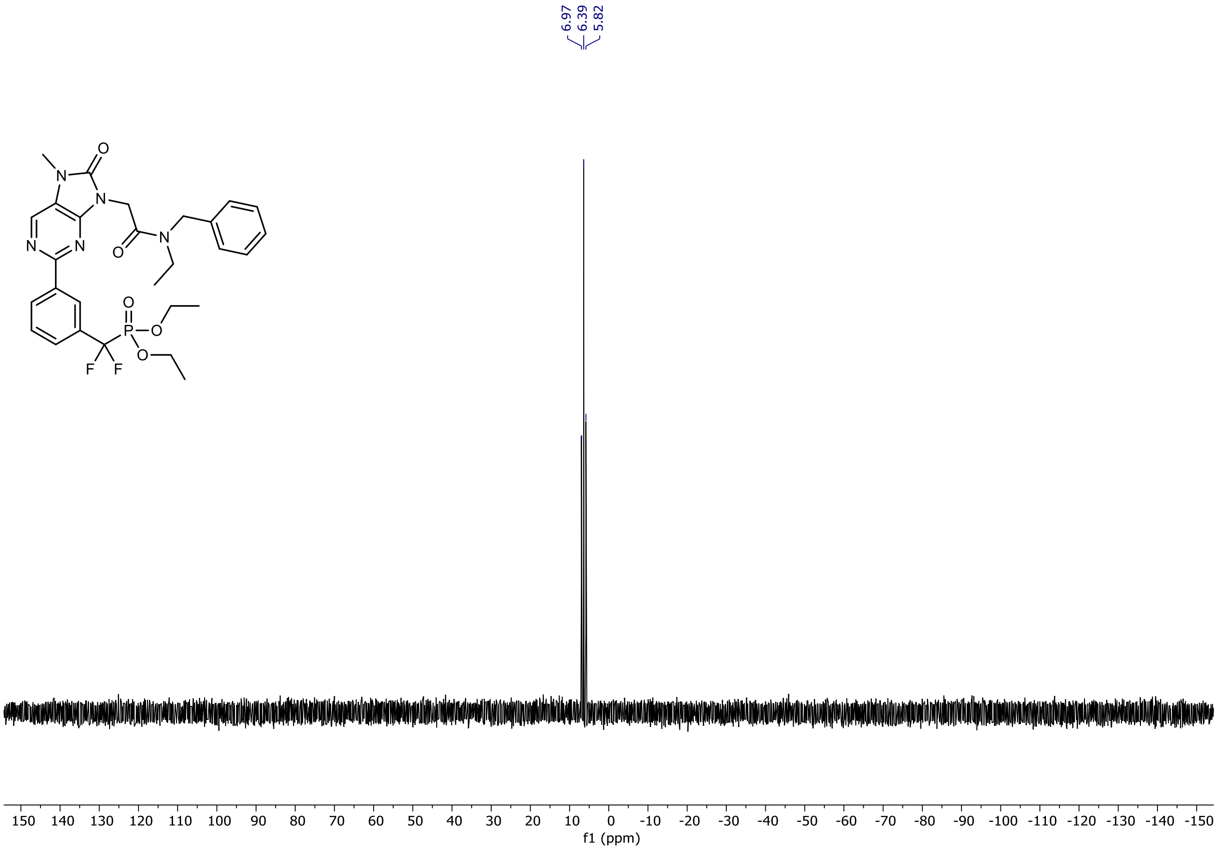

Supplementary Fig. 188. ^{31}P NMR spectra (203 MHz, $CDCl_3$, 298 K) of compound **6g**.

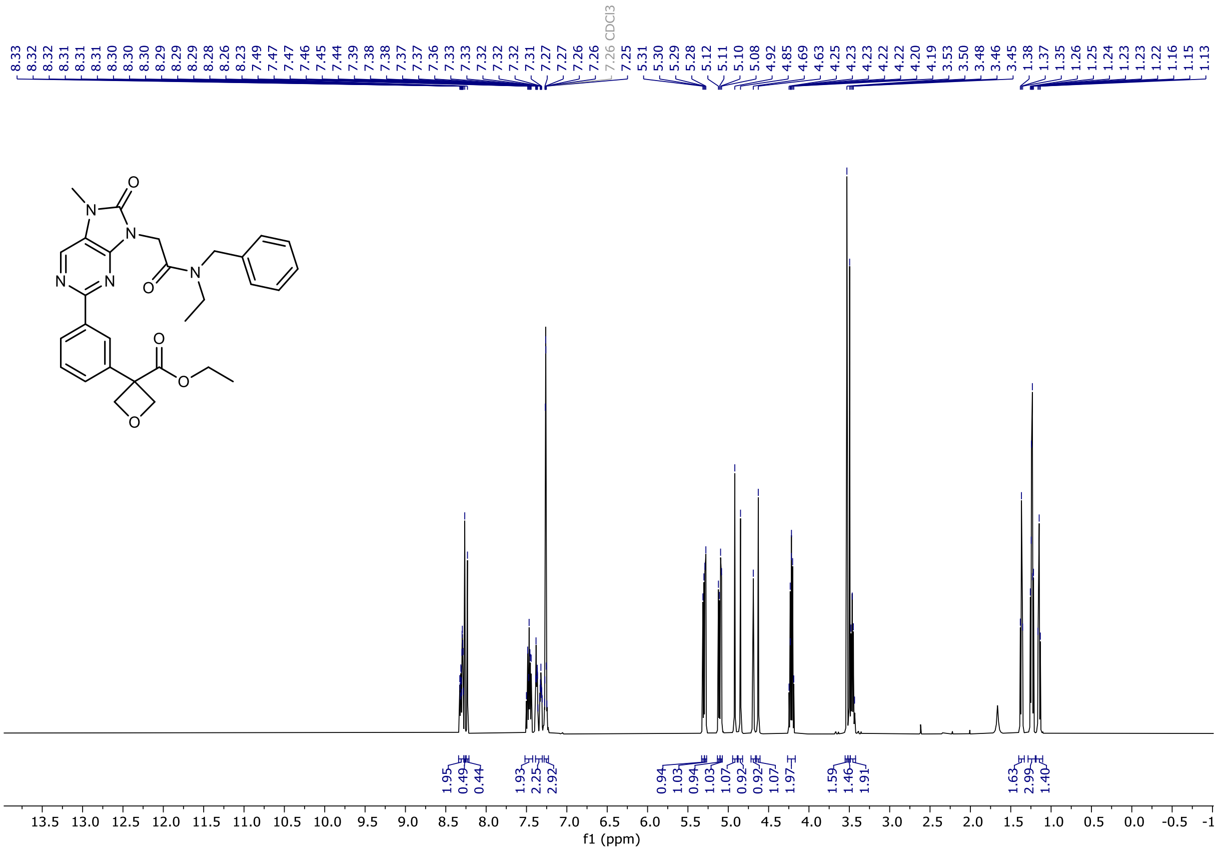

Supplementary Fig. 189. ^{1}H NMR spectra (500 MHz, $CDCl_3$, 298 K) of compound **6h**.

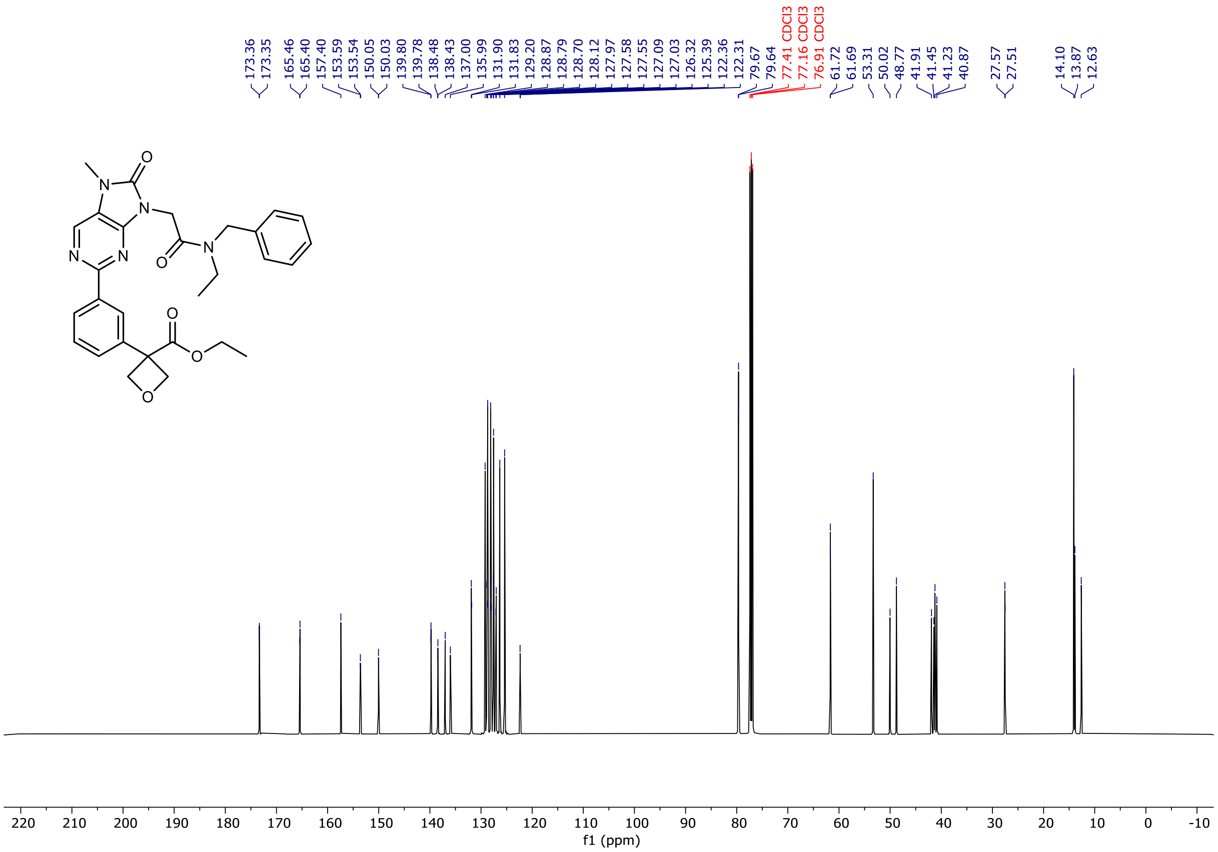

Supplementary Fig. 190. ^{13}C NMR spectra (126 MHz, $CDCl_3$, 298 K) of compound **6h**.

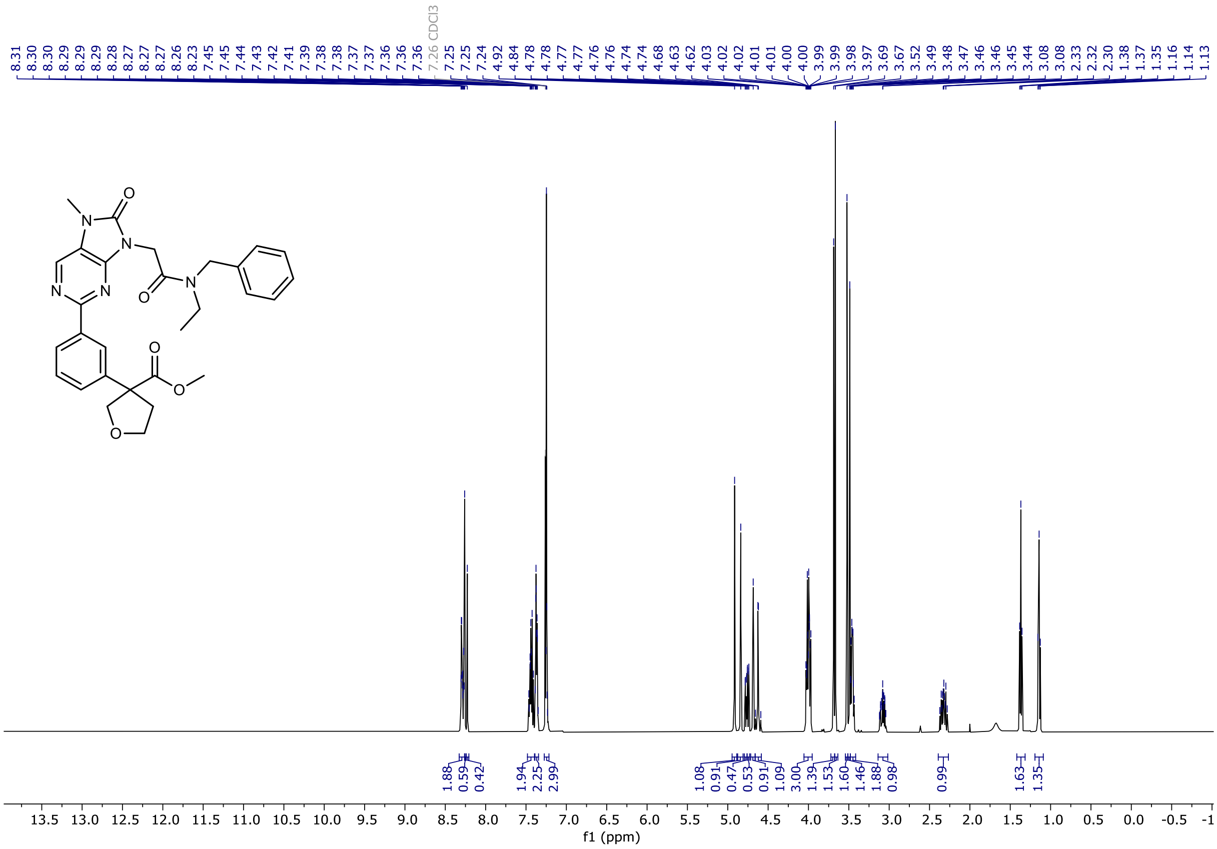

Supplementary Fig. 191. ^{1}H NMR spectra (500 MHz, $CDCl_3$, 298 K) of compound **6i**.

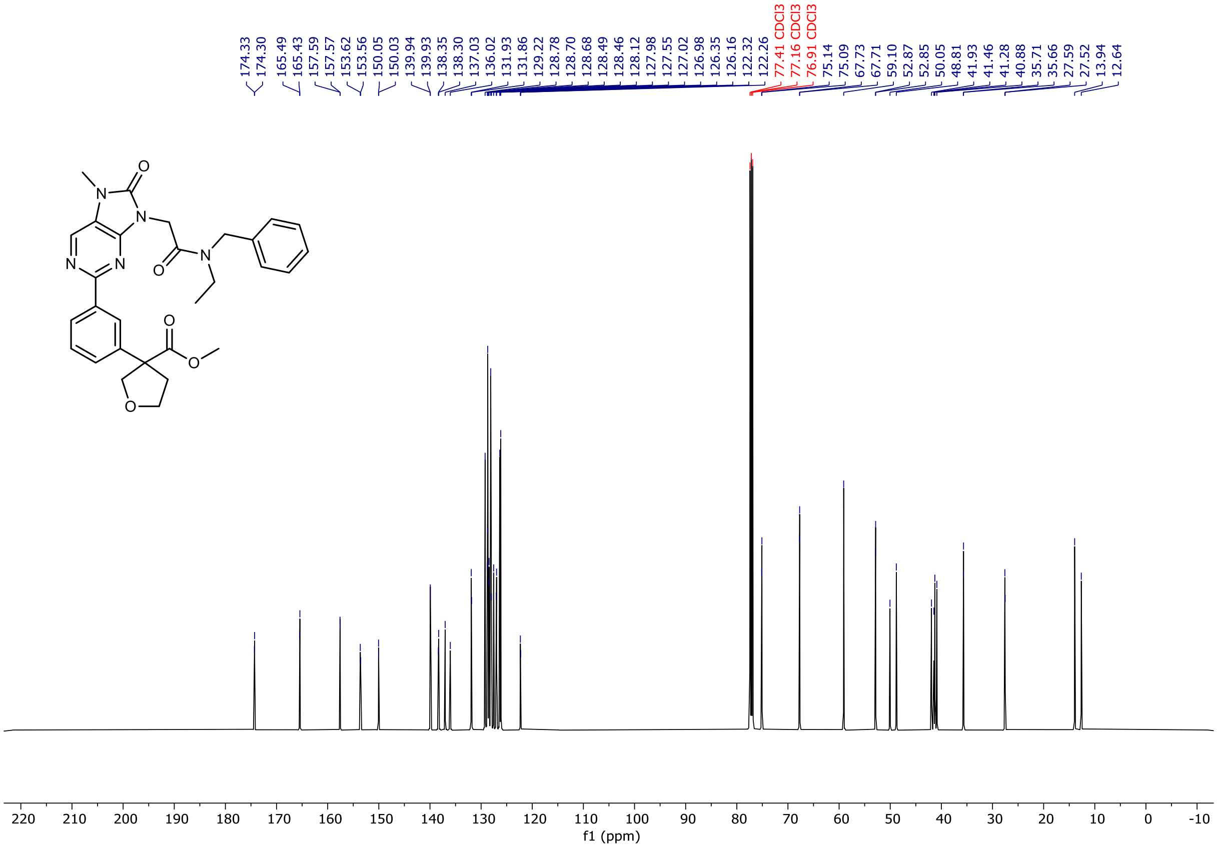

Supplementary Fig. 192. ^{13}C NMR spectra (126 MHz, $CDCl_3$, 298 K) of compound **6i**.

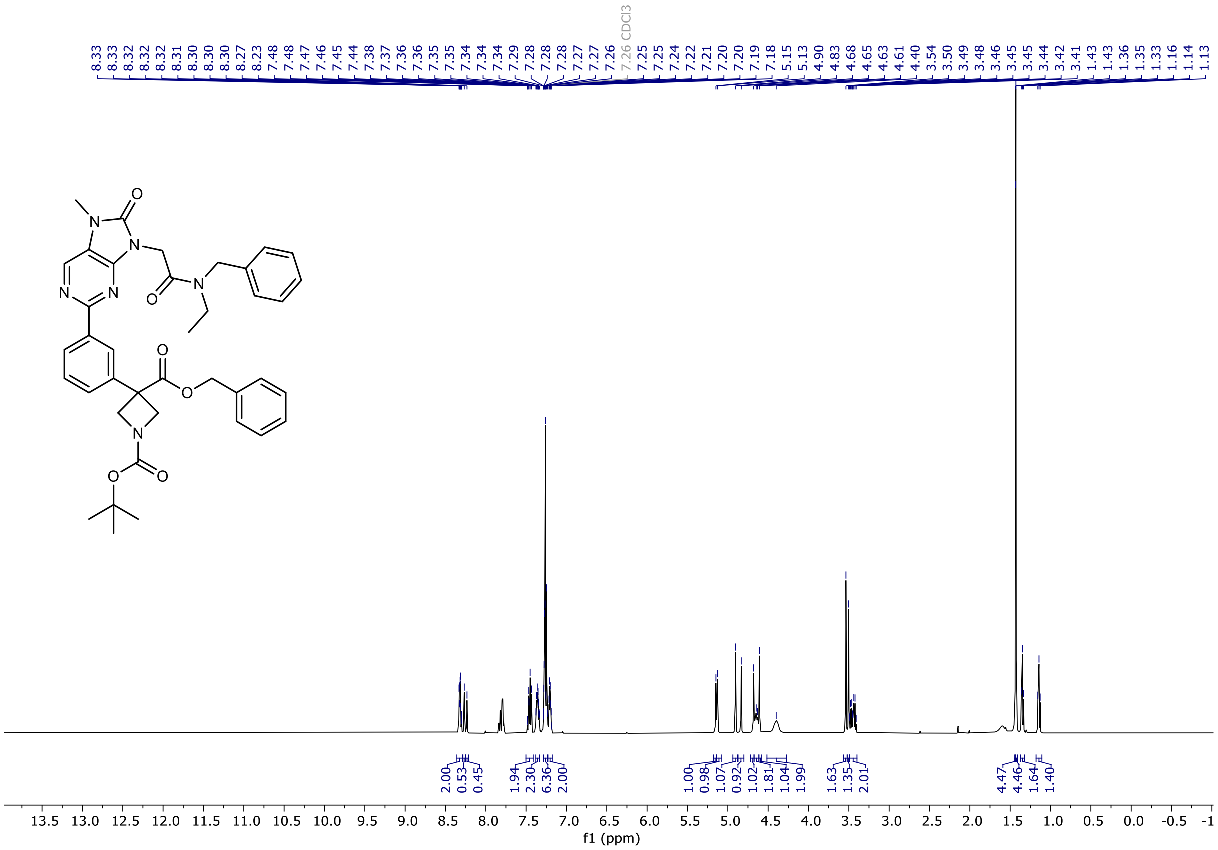

Supplementary Fig. 193. ^{1}H NMR spectra (500 MHz, $CDCl_3$, 298 K) of compound **6j**.

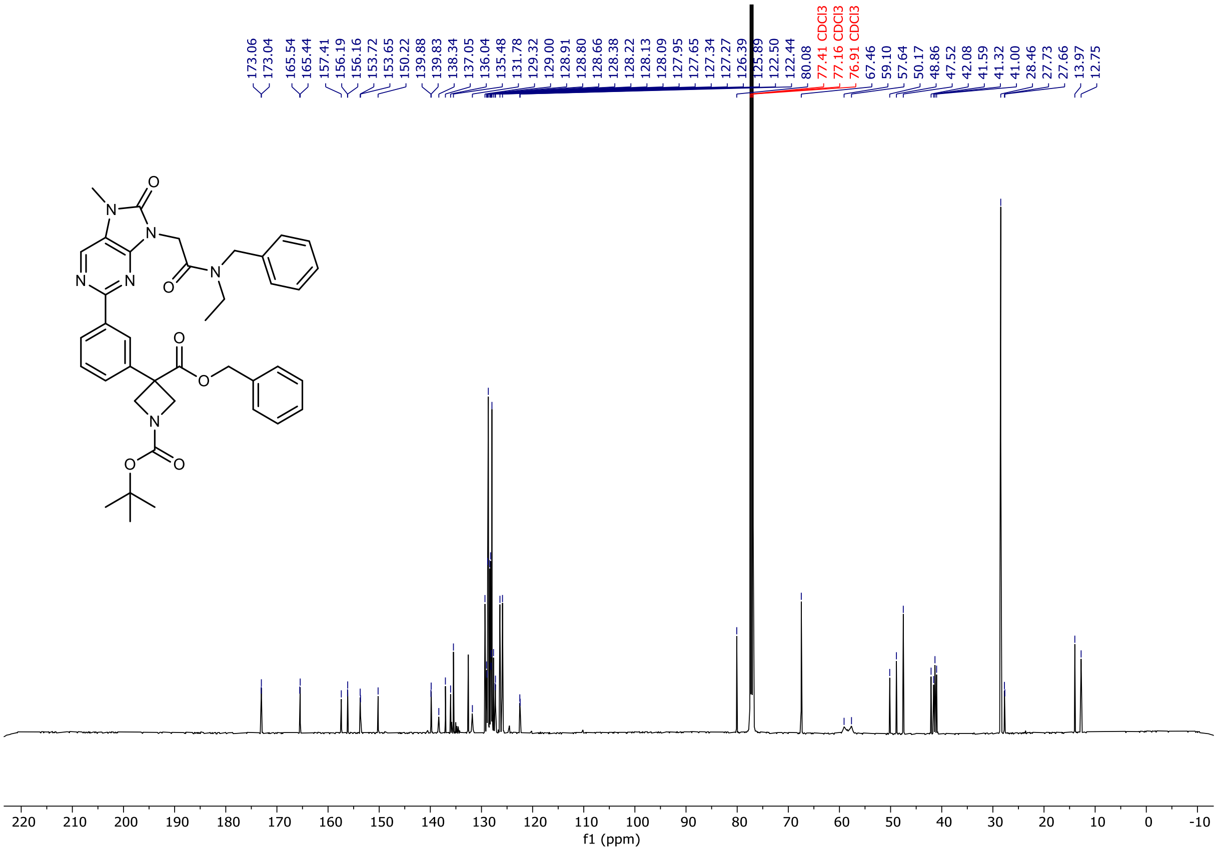

Supplementary Fig. 194. ^{13}C NMR spectra (126 MHz, $CDCl_3$, 298 K) of compound **6j**.

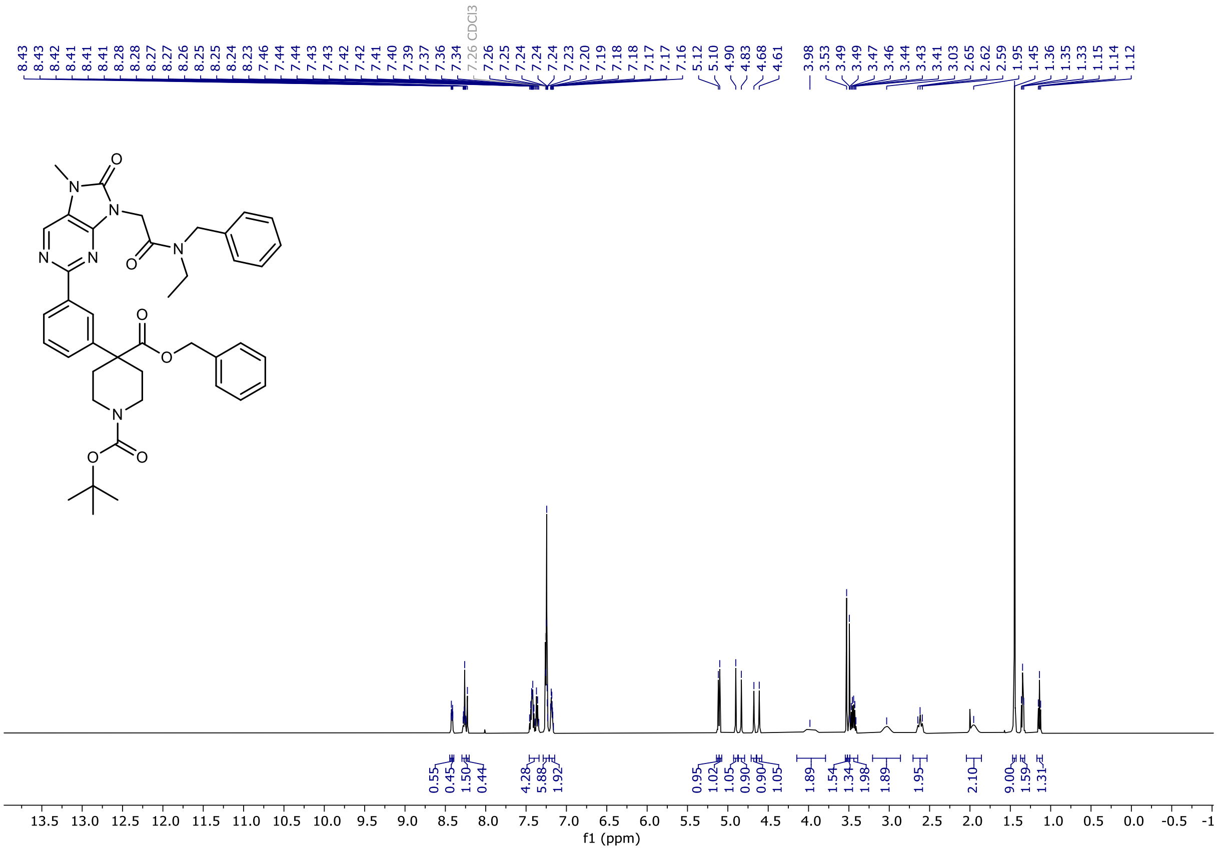

Supplementary Fig. 195. ^{1}H NMR spectra (500 MHz, $CDCl_3$, 298 K) of compound **6k**.

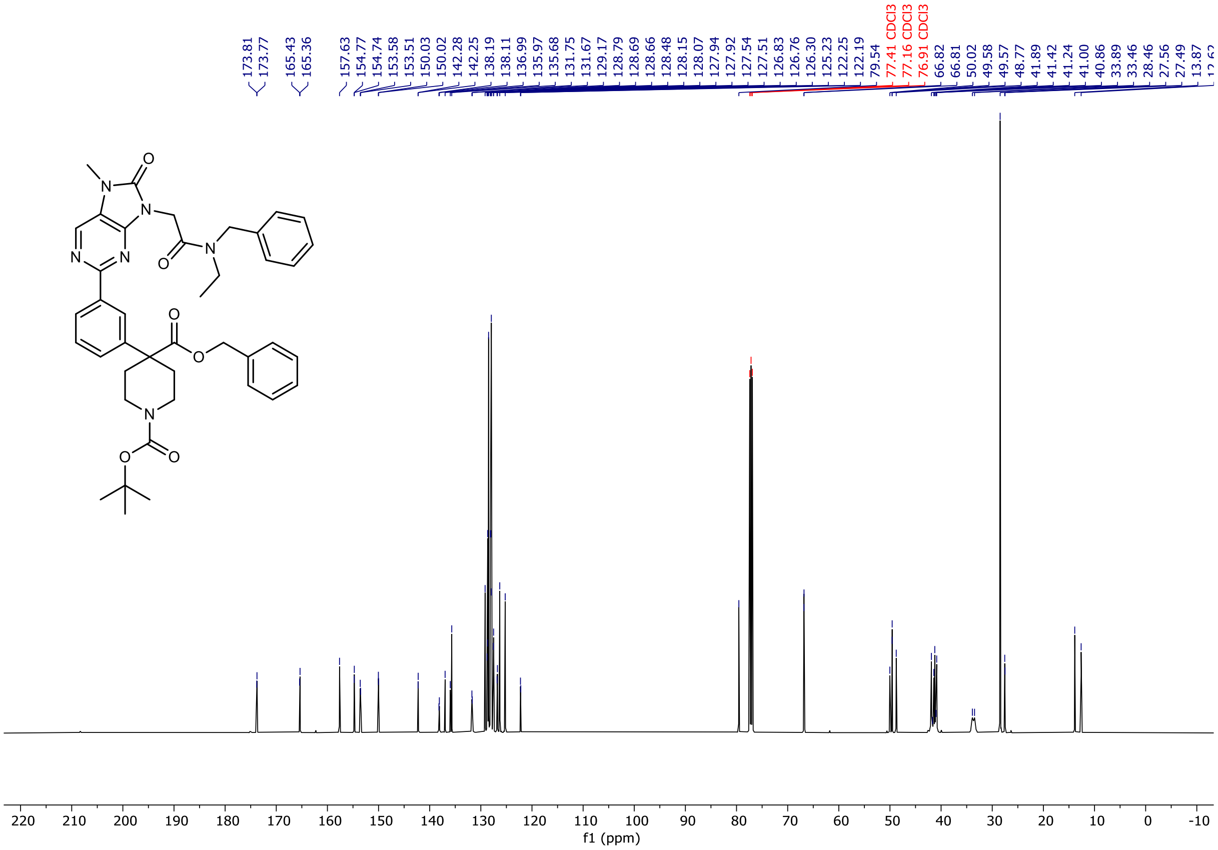

Supplementary Fig. 196. ^{13}C NMR spectra (126 MHz, $CDCl_3$, 298 K) of compound **6k**.

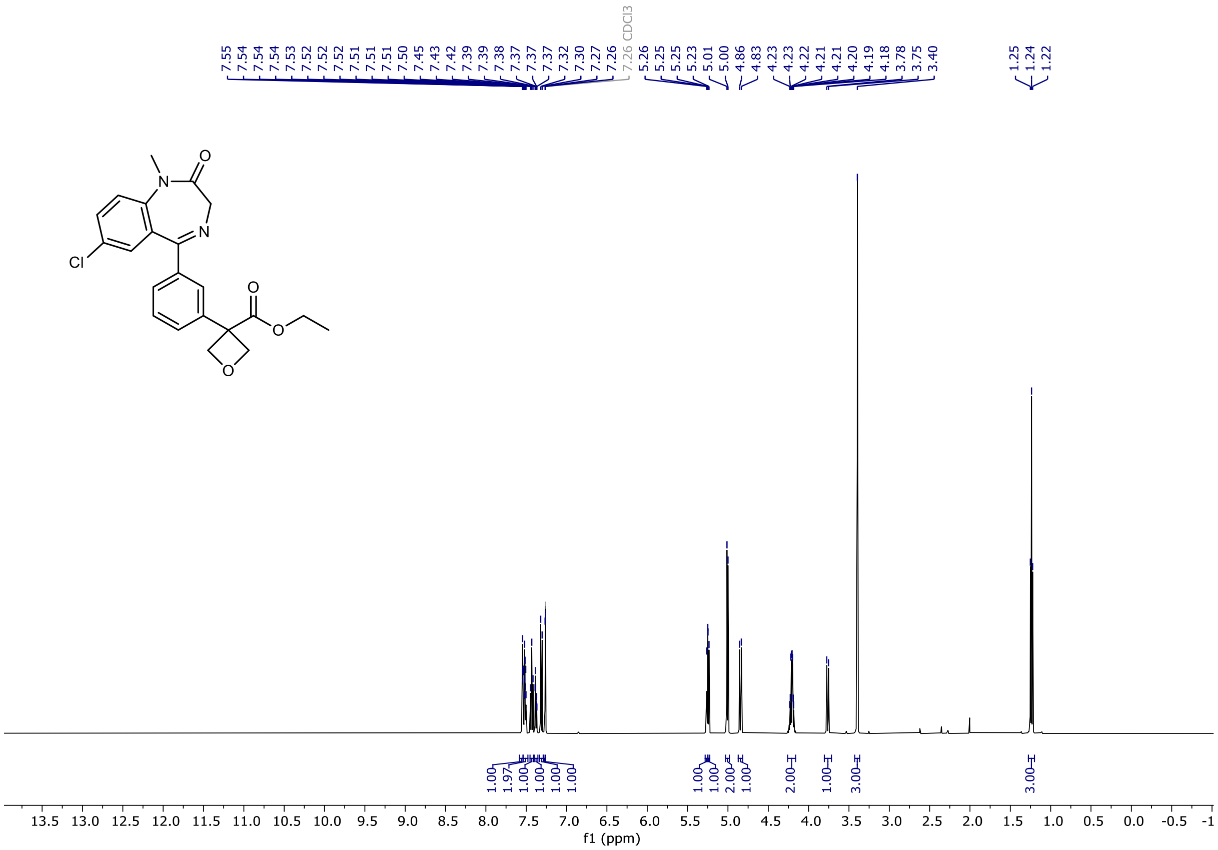

Supplementary Fig. 197. ^{1}H NMR spectra (500 MHz, $CDCl_3$, 298 K) of compound **7**.

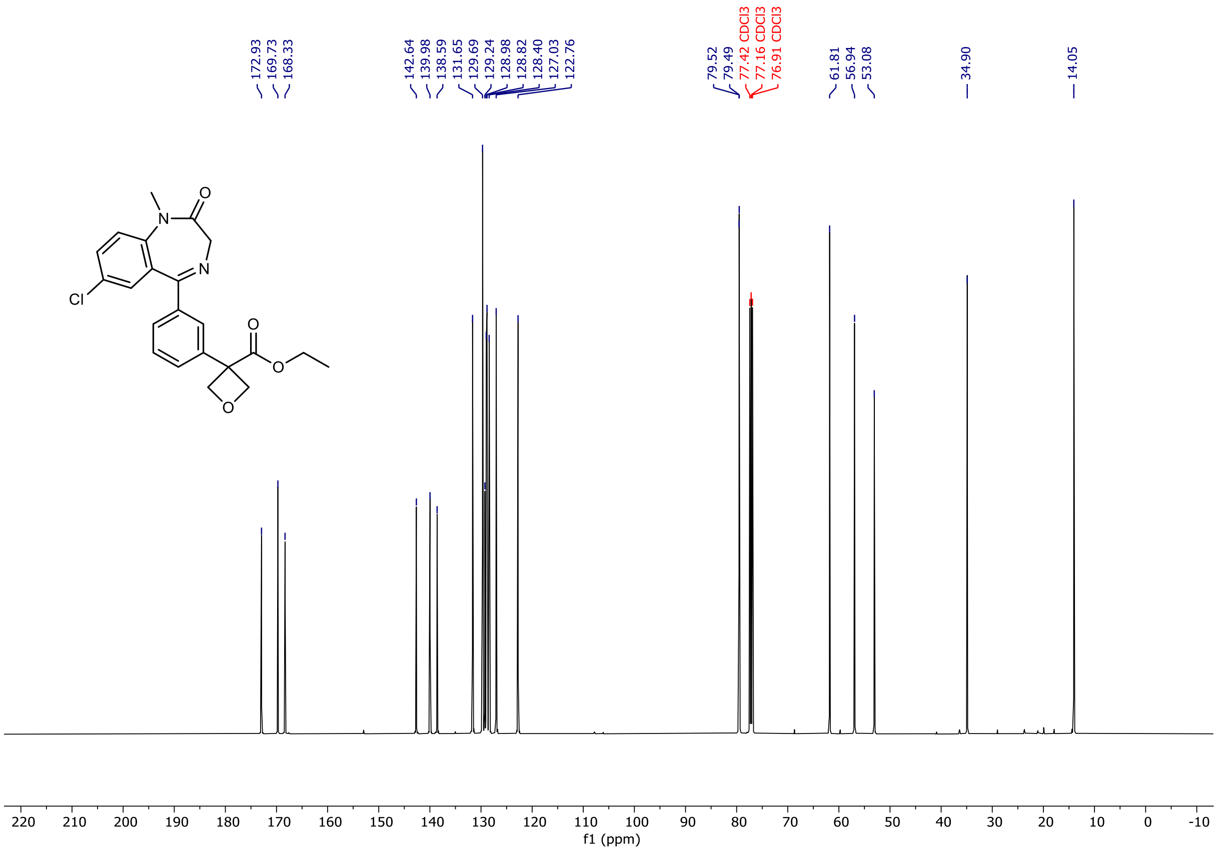

Supplementary Fig. 198. ^{13}C NMR spectra (126 MHz, $CDCl_3$, 298 K) of compound **7**.

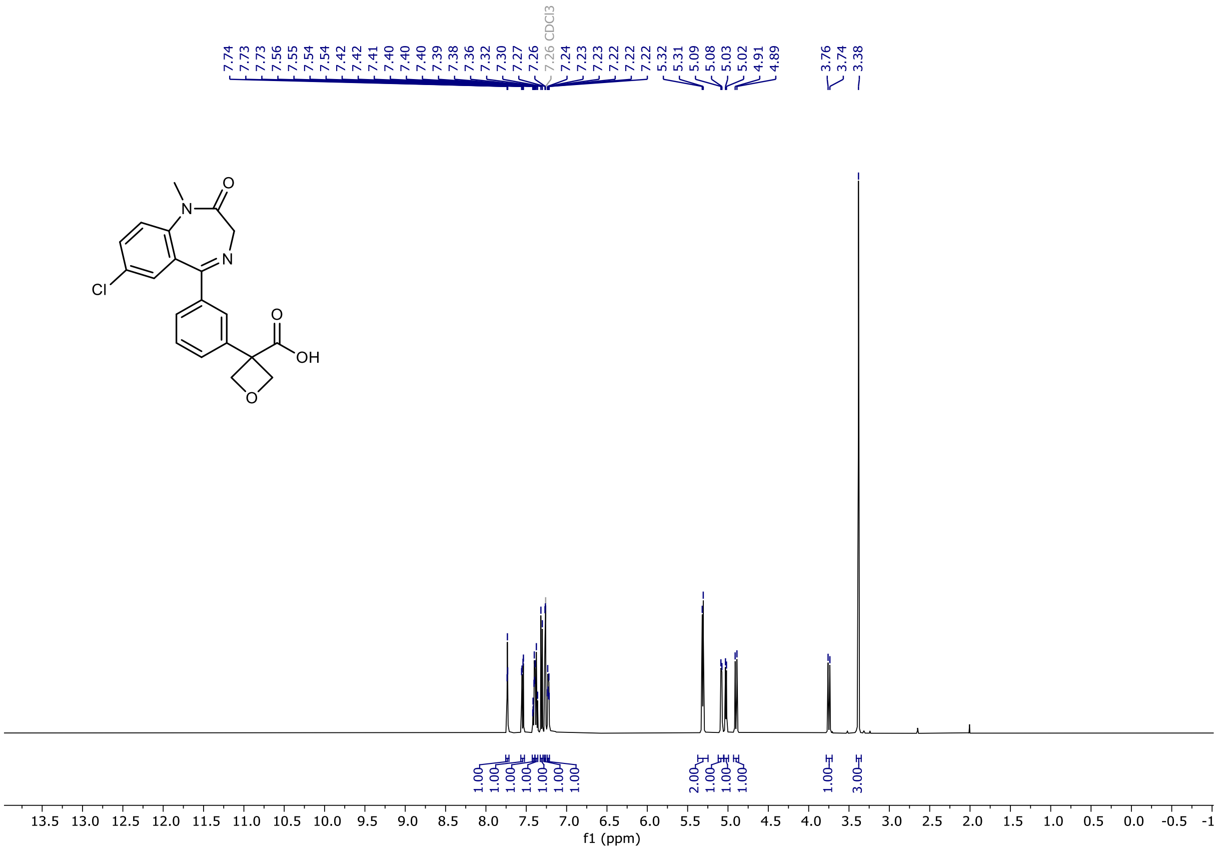

Supplementary Fig. 199. ^{1}H NMR spectra (500 MHz, $CDCl_3$, 298 K) of compound **8a**.

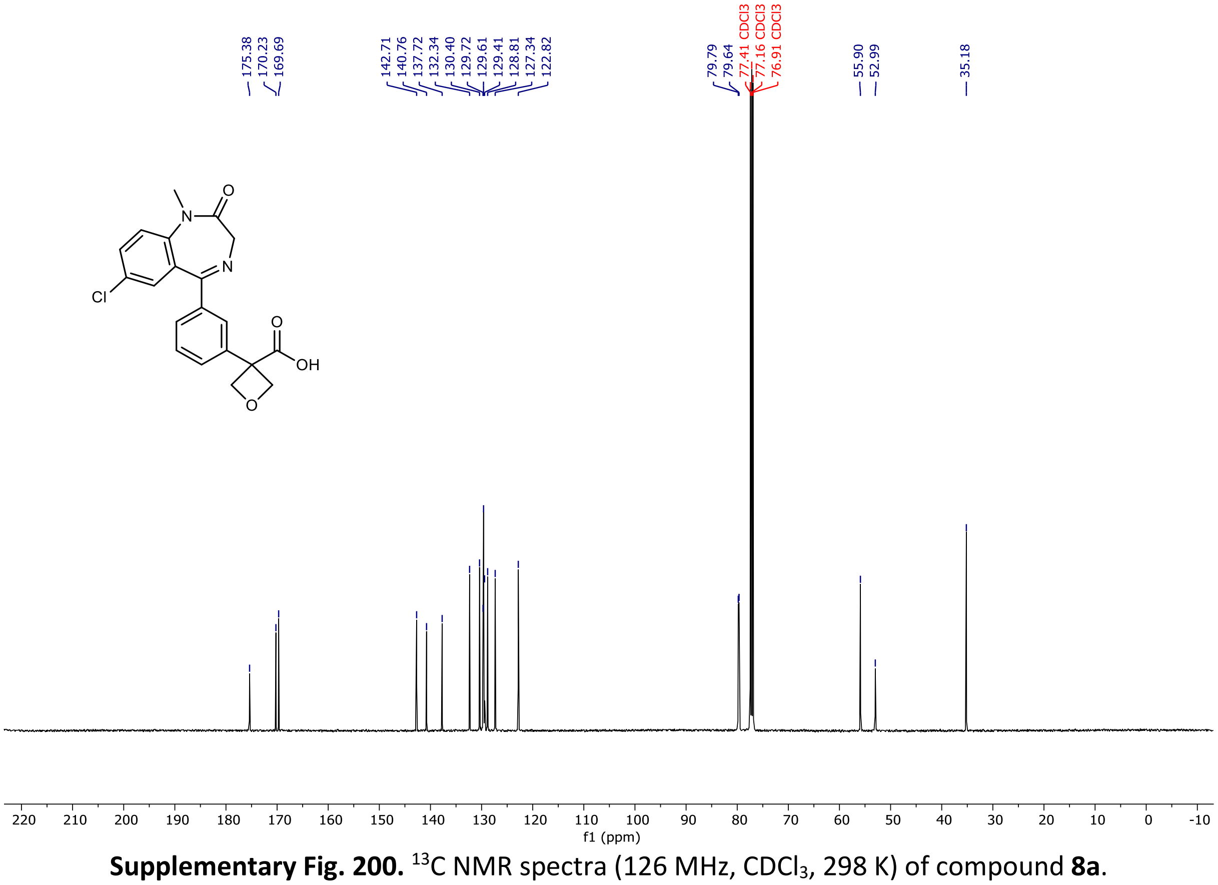

Supplementary Fig. 200. ^{13}C NMR spectra (126 MHz, $CDCl_3$, 298 K) of compound **8a**.

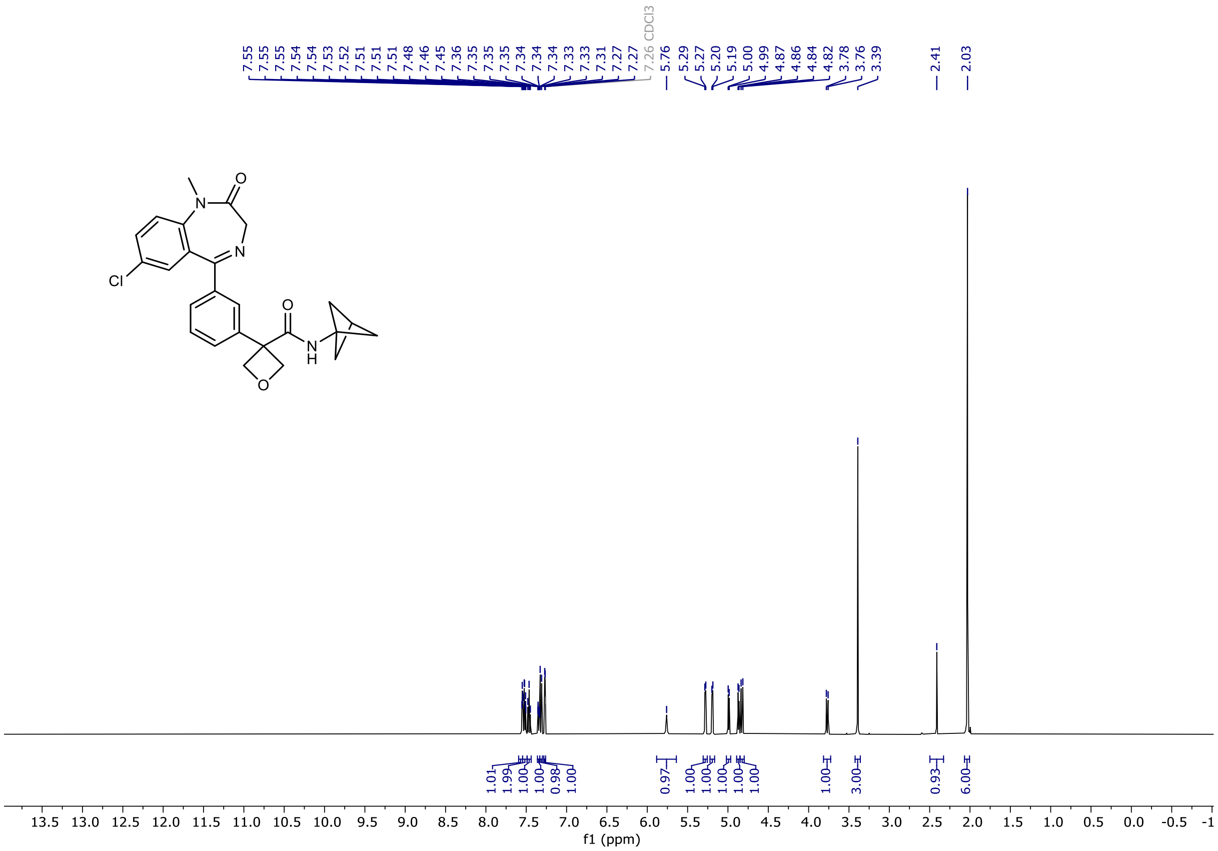

Supplementary Fig. 201. ^{1}H NMR spectra (500 MHz, $CDCl_3$, 298 K) of compound **8b**.

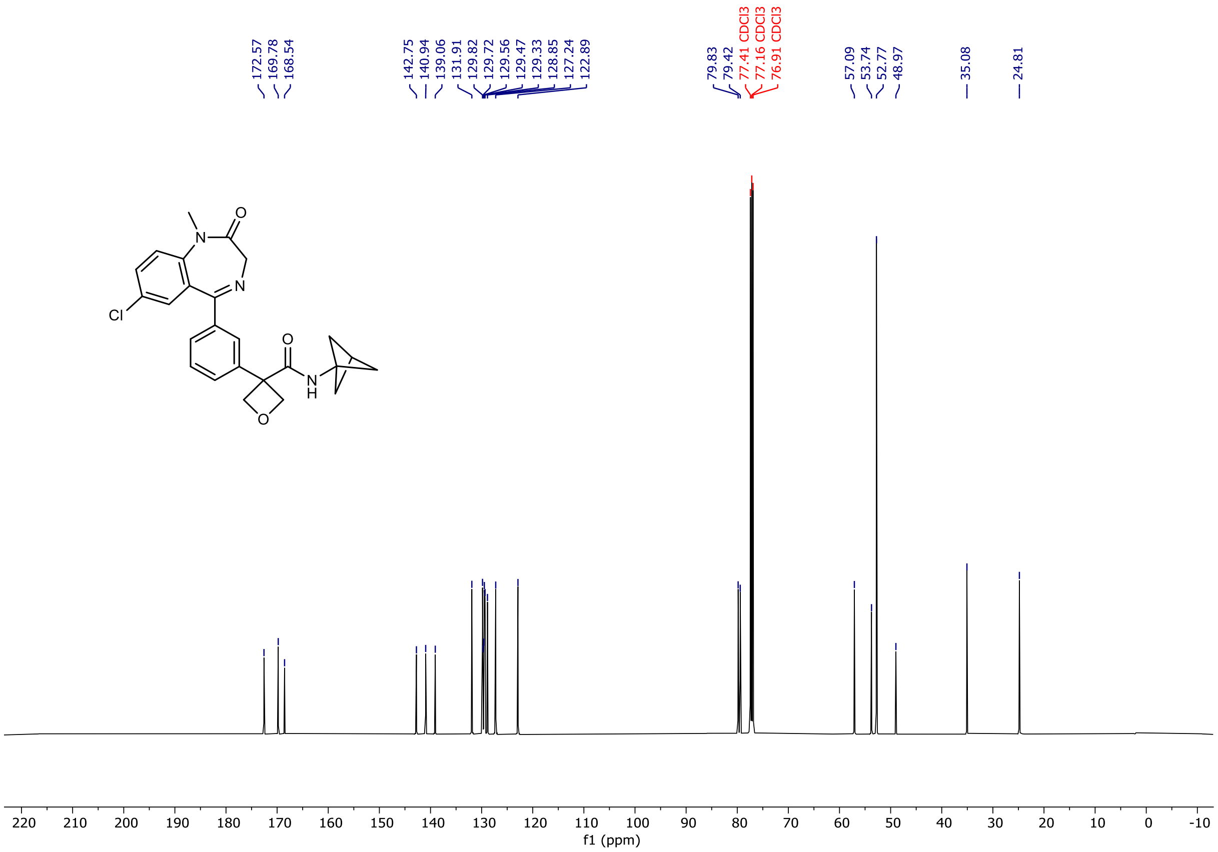

Supplementary Fig. 202. ^{13}C NMR spectra (126 MHz, $CDCl_3$, 298 K) of compound **8b**.

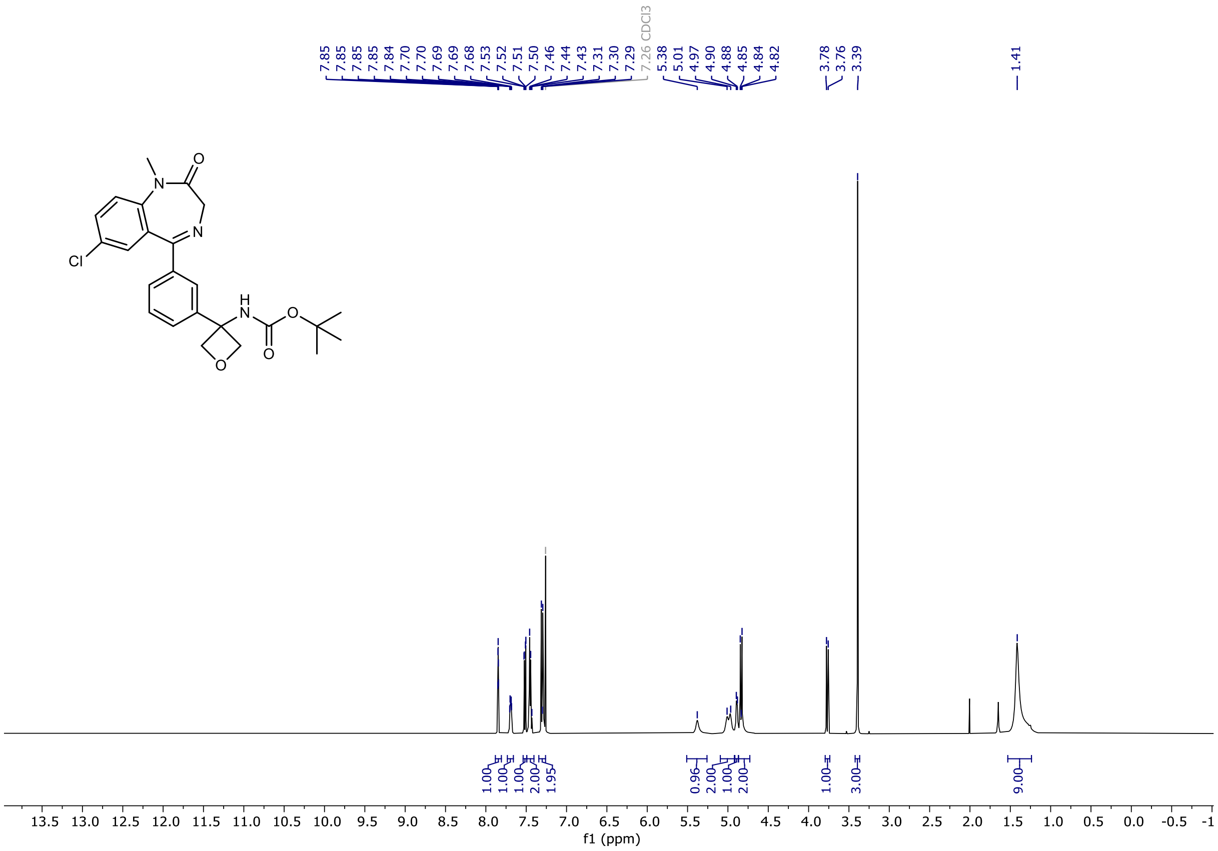

Supplementary Fig. 203. ^{1}H NMR spectra (500 MHz, $CDCl_3$, 298 K) of compound **8c**.

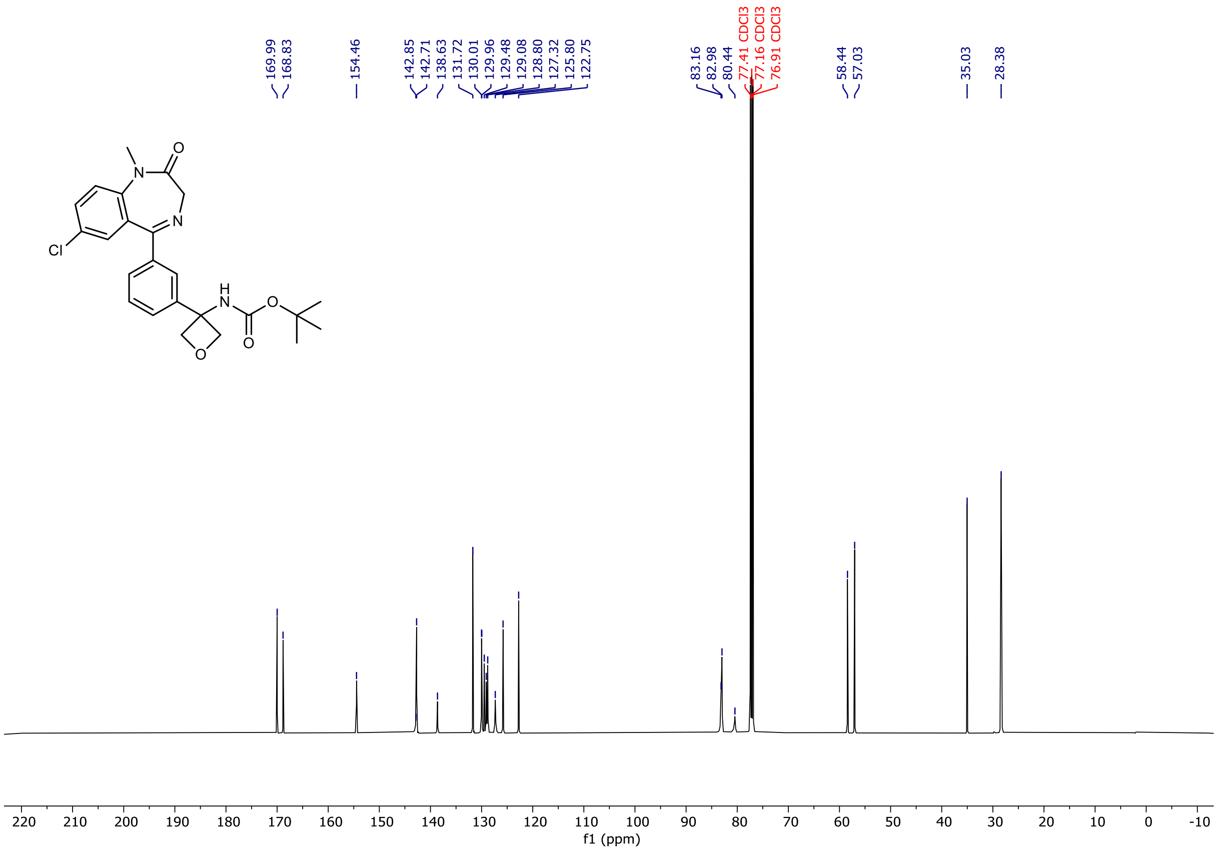

Supplementary Fig. 204. ^{13}C NMR spectra (126 MHz, $CDCl_3$, 298 K) of compound **8c**.

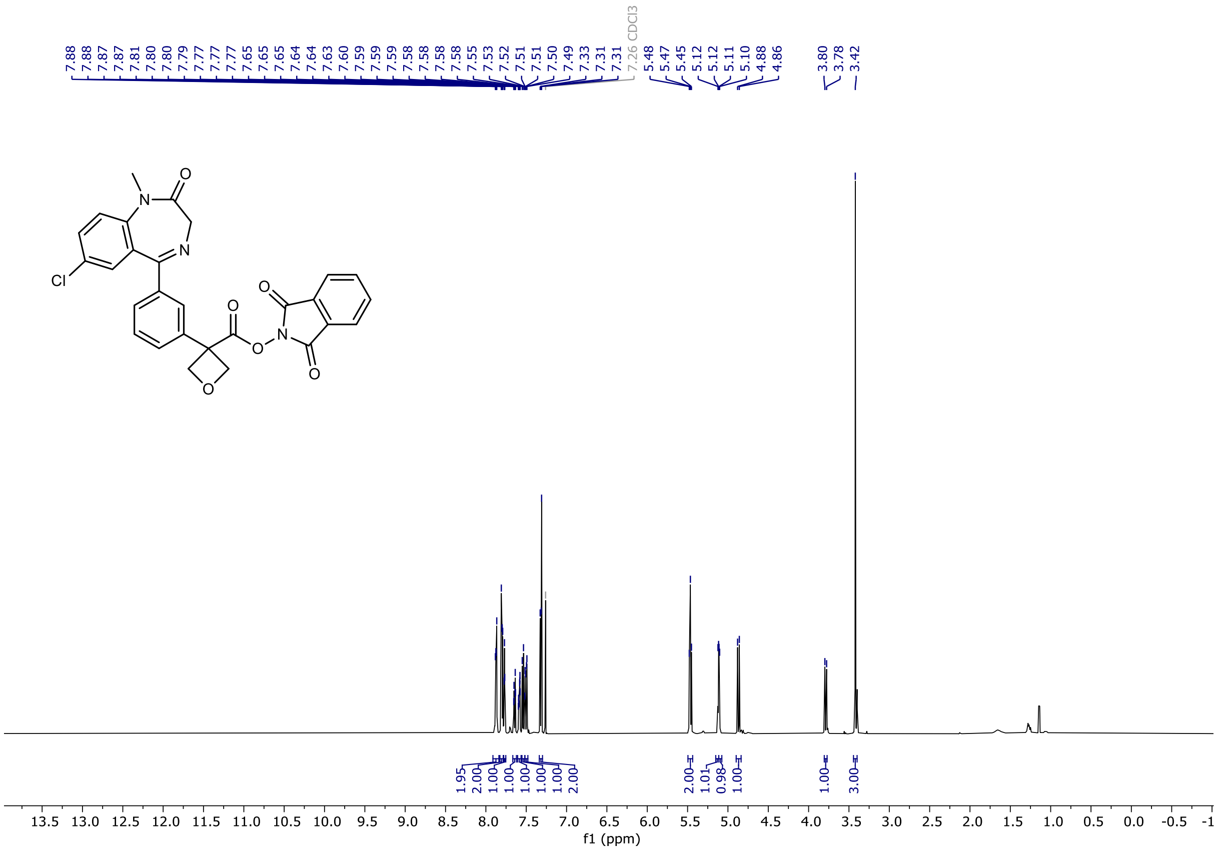

Supplementary Fig. 205. ^{1}H NMR spectra (500 MHz, $CDCl_3$, 298 K) of compound **8d**.

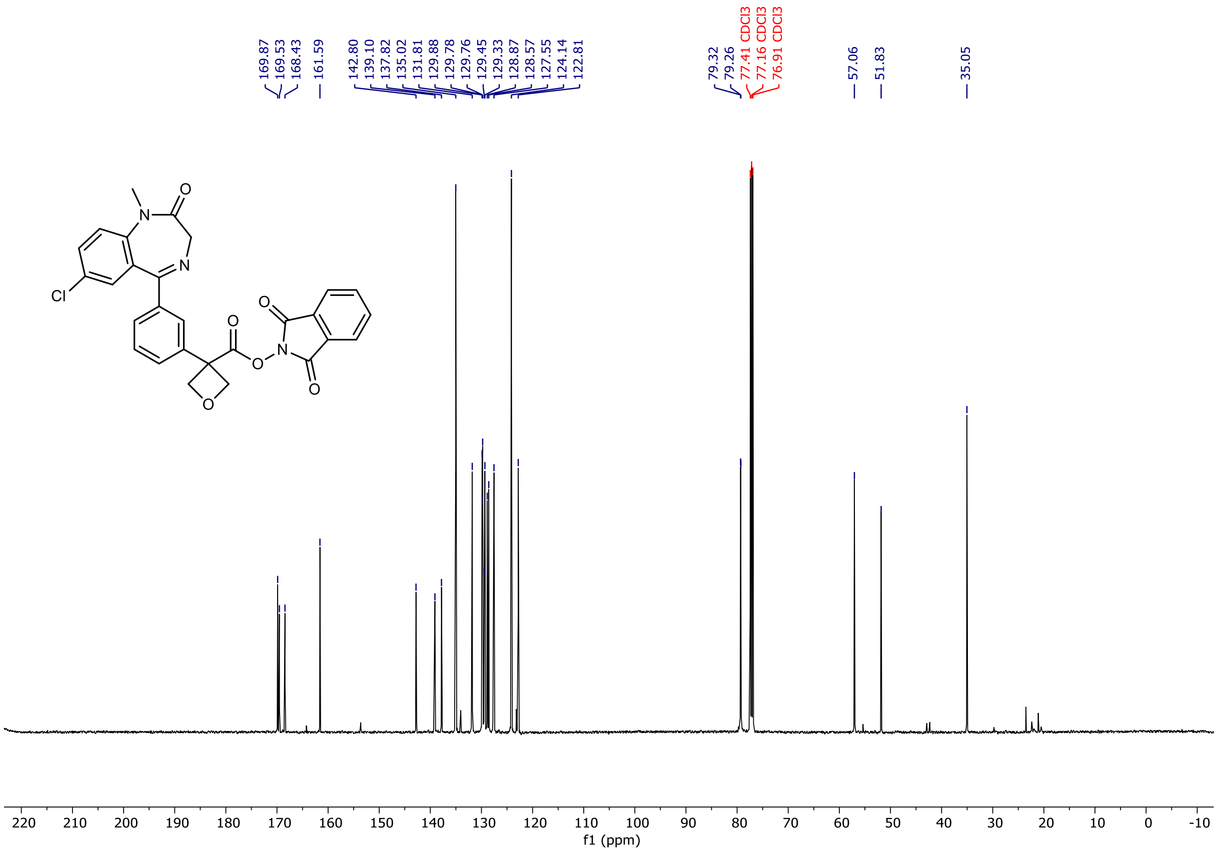

Supplementary Fig. 206. ^{13}C NMR spectra (126 MHz, $CDCl_3$, 298 K) of compound **8d**.

Supplementary References

1. Ackermann, L. & Lygin, A. V. Cationic ruthenium(II) catalysts for oxidative C–H/N–H bond functionalizations of anilines with removable directing group: synthesis of indoles in water. *Org. Lett.* **14**, 764–767 (2012).
2. Ackermann, L., Vicente, R., Potukuchi, H. K. & Pirovano, V. Mechanistic insight into direct arylations with ruthenium(II) carboxylate catalysts. *Org. Lett.* **12**, 5032–5035 (2010).
3. Vitnik, V. D. *et al.* One-step conversion of ketones to conjugated acids using bromoform. *Synth. Commun.* **39**, 1457–1471 (2009).
4. Palao, E. *et al.* Formation of quaternary carbons through cobalt-catalyzed $C(sp^3)–C(sp^3)$ Negishi cross-coupling. *Chem. Commun.* **56**, 8210–8213 (2020).
5. Gou, Q. *et al.* Pd^{II}-catalyzed purine-directed *ortho* nitration of 6-arylpurines by $C(sp^2)–H$ activation: a practical approach to synthesize 6-(2-nitroaryl)-purine derivatives. *Eur. J. Org. Chem.* **2018**, 4089–4094 (2018).
6. Simonetti, M., Cannas, D. M., Just-Baringo, X., Vitorica-Yrezabal, I. J. & Larrosa, I. Cyclometallated ruthenium catalyst enables late-stage directed arylation of pharmaceuticals. *Nat. Chem.* **10**, 724–731 (2018).
7. Ruan, Z. *et al.* Ruthenium(II)-catalyzed *meta* C−H mono- and difluoromethylations by phosphine/carboxylate cooperation. *Angew. Chem. Int. Ed.* **56**, 2045–2049 (2017).
8. Sagadevan, A. & Greaney, M. F. *meta*-Selective C−H activation of arenes at room temperature using visible light: dual-function ruthenium catalysis. *Angew. Chem. Int. Ed.* **58**, 9826–9830 (2019).
9. Paterson, A. J. *et al.* α-Halo carbonyls enable *meta* selective primary, secondary and tertiary C–H alkylations by ruthenium catalysis. *Org. Biomol. Chem.* **15**, 5993–6000 (2017).
10. Li, Z.-Y. *et al.* Ruthenium-catalyzed *meta*-selective C−H mono- and difluoromethylation of arenes through *ortho*-metalation strategy. *Chem. Eur. J.* **23**, 3285–3290 (2017).
11. Inghardt, T. *et al.* Discovery of AZD4831, a mechanism-based irreversible inhibitor of myeloperoxidase, as a potential treatment for heart failure with preserved ejection fraction. *J. Med. Chem.* **65**, 11485–11496 (2022).
12. Wan, H. & Holmen, A. G. High throughput screening of physicochemical properties and in vitro ADME profiling in drug discovery. *Comb. Chem. High Throughput Screen.* **12**, 315–329 (2009).
13. Leo, A., Hansch, C. & Elkins, D. Partition coefficients and their uses. *Chem. Rev.* **71**, 525–616 (1971).
14. Jacobson, L. *et al.* An Optimized automated assay for determination of metabolic stability using hepatocytes: assay validation, variance component analysis, and in vivo relevance. *ASSAY Drug Dev. Technol.* **5**, 403–416 (2007).
