# Supplementary material for: Late-stage meta-C–H alkylation of pharmaceuticals to modulate biological properties and expedite molecular optimisation in a single step
Source: Nat Commun. 2024 Apr 18;15:3349. doi: 10.1038/s41467-024-46697-8 (PMC11026381; doi:10.1038/s41467-024-46697-8)
Supplement: Supplementary file 3 — Reporting Summary [file 41467_2024_46697_MOESM3_ESM.pdf]

nature portfolio

Corresponding author(s): Lutz Ackermann and Magnus J. Johansson

Last updated by author(s): Feb 13, 2024

Reporting Summary

Nature Portfolio wishes to improve the reproducibility of the work that we publish. This form provides structure for consistency and transparency in reporting. For further information on Nature Portfolio policies, see our Editorial Policies and the Editorial Policy Checklist.

Statistics

For all statistical analyses, confirm that the following items are present in the figure legend, table legend, main text, or Methods section.

n/a	Confirmed	
☐	☒	The exact sample size (*n*) for each experimental group/condition, given as a discrete number and unit of measurement
☒	☐	A statement on whether measurements were taken from distinct samples or whether the same sample was measured repeatedly
☒	☐	The statistical test(s) used AND whether they are one- or two-sided<br>*Only common tests should be described solely by name; describe more complex techniques in the Methods section.*
☒	☐	A description of all covariates tested
☒	☐	A description of any assumptions or corrections, such as tests of normality and adjustment for multiple comparisons
☒	☐	A full description of the statistical parameters including central tendency (e.g. means) or other basic estimates (e.g. regression coefficient) AND variation (e.g. standard deviation) or associated estimates of uncertainty (e.g. confidence intervals)
☒	☐	For null hypothesis testing, the test statistic (e.g. *F*, *t*, *r*) with confidence intervals, effect sizes, degrees of freedom and *P* value noted<br>*Give P values as exact values whenever suitable.*
☒	☐	For Bayesian analysis, information on the choice of priors and Markov chain Monte Carlo settings
☒	☐	For hierarchical and complex designs, identification of the appropriate level for tests and full reporting of outcomes
☒	☐	Estimates of effect sizes (e.g. Cohen's *d*, Pearson's *r*), indicating how they were calculated

Our web collection on statistics for biologists contains articles on many of the points above.

Software and code

Policy information about availability of computer code

Data collection	TopSpin v3 and IconNMR v5 for collection of NMR data, MassLynx v4 for collection of UPLC-MS data.
Data analysis	MestReNova v14 for analysis of NMR data, MassLynx v4 for analysis of UPLC-MS data, TIBCO Spotfire v11 for generation of heat maps and data visualisation.

For manuscripts utilizing custom algorithms or software that are central to the research but not yet described in published literature, software must be made available to editors and reviewers. We strongly encourage code deposition in a community repository (e.g. GitHub). See the Nature Portfolio guidelines for submitting code & software for further information.

Data

Policy information about availability of data

All manuscripts must include a data availability statement. This statement should provide the following information, where applicable:

- Accession codes, unique identifiers, or web links for publicly available datasets
- A description of any restrictions on data availability
- For clinical datasets or third party data, please ensure that the statement adheres to our policy

The data generated in this study are provided within the paper and its Supplementary Information files. This includes general information; extended HTE data for the reaction optimisation and LSF substrates screening; experimental procedures and analytical details with characterisation data for all products; further physicochemical and DMPK data with details on the pharmaceutically relevant properties modulation of emapunil analogues; NMR spectra for all compounds. These data are also available from the corresponding authors upon request.

Research involving human participants, their data, or biological material

Policy information about studies with human participants or human data. See also policy information about sex, gender (identity/presentation), and sexual orientation and race, ethnicity and racism.

Reporting on sex and gender	n/a
Reporting on race, ethnicity, or other socially relevant groupings	n/a
Population characteristics	n/a
Recruitment	n/a
Ethics oversight	n/a

Note that full information on the approval of the study protocol must also be provided in the manuscript.

Field-specific reporting

Please select the one below that is the best fit for your research. If you are not sure, read the appropriate sections before making your selection.

☒ Life sciences ☐ Behavioural & social sciences ☐ Ecological, evolutionary & environmental sciences

For a reference copy of the document with all sections, see nature.com/documents/nr-reporting-summary-flat.pdf

Life sciences study design

All studies must disclose on these points even when the disclosure is negative.

Sample size	Emapunil (4e) and all the synthesised analogues (5e, 6a-6k) in Fig. 5a were subjected to fundamental in vitro drug discovery assays, for collecting standard physicochemical and DMPK data: solubility, LogD, human plasma protein binding (Prot. Bind.) and intrinsic clearance (CLint) in human liver microsomes (HLM) or rat hepatocytes (Rat Hep.).<br>As such the sample size was determined by the number and diversity of synthesised compounds, which provided a good understanding of the reaction scope and the modulation of biological properties upon late-stage C–H alkylation. The sample size enabled a satisfactory comparison of biologically relevant properties between the meta-alkylated analogues (including the corresponding free acid derivatives for metabolic stability data) and the parent molecule as reference. Other drug-like molecules synthesised were not included since any data generated would not be meaningful for the direct comparison of pharmaceutical properties between diverse bioactive compounds.
Data exclusions	No data was excluded.
Replication	For all data, n = 1 unless otherwise noted. All assays are thoroughly validated in-house or by a third party CRO to reliably provide high quality data sufficient for our drug discovery effort, ensuring the integrity of our screens.
Randomization	This is not relevant, as all assays are thoroughly validated in-house or by a third party CRO to reliably provide high quality data sufficient for our drug discovery efforts. While the samples of the study were technically not randomised, they were tested alongside many unrelated samples in high-throughput physicochemical, DMPK and in vitro pharmacological assays, ensuring the integrity of our screens.
Blinding	This is not relevant, as all assays are thoroughly validated in-house or by a third party CRO to reliably provide high quality data sufficient for our drug discovery efforts. While the samples of the study were technically not blinded, they were tested alongside many unrelated samples in high-throughput physicochemical, DMPK, and in vitro pharmacological assays, ensuring the integrity of our screens.

Reporting for specific materials, systems and methods

We require information from authors about some types of materials, experimental systems and methods used in many studies. Here, indicate whether each material, system or method listed is relevant to your study. If you are not sure if a list item applies to your research, read the appropriate section before selecting a response.

Materials & experimental systems

n/a	Involved in the study
☒	☐ Antibodies
☐	☒ Eukaryotic cell lines
☒	☐ Palaeontology and archaeology
☒	☐ Animals and other organisms
☒	☐ Clinical data
☒	☐ Dual use research of concern
☒	☐ Plants

Methods

n/a	Involved in the study
☒	☐ ChIP-seq
☒	☐ Flow cytometry
☒	☐ MRI-based neuroimaging

Eukaryotic cell lines

Policy information about cell lines and Sex and Gender in Research

Cell line source(s)	HLM (CLint): Human Liver Microsomes - InVitroCYP 150-Donor, Mixed Gender (BioIVT: Product No. X008070, Lot No. QQY).<br>Rat Hep. (CLint): Rat Hepatocytes - Cryosuspension, Male Wistar Hannover (BioIVT: Product No. M00065, Lot No. DVO).
Authentication	Human Liver Microsomes (HLM) & Han Wistar Rat Hepatocytes (Rat Hep.): Harvested and Supplied by BioIVT.<br>Not kept in continuous culture. Authentication not relevant.
Mycoplasma contamination	Human Liver Microsomes (HLM) & Han Wistar Rat Hepatocytes (Rat Hep.): Harvested and Supplied by BioIVT.<br>Not kept in continuous culture. Mycoplasma testing not relevant.
Commonly misidentified lines<br>(See ICLAC register)	No commonly misidentified cell lines were used.

Plants

Seed stocks	n/a
Novel plant genotypes	n/a
Authentication	n/a
